# Indoloindolizines: The Complete Story of a Polycyclic Aromatic Scaffold from Theoretical Design to Organic Field-Effect Transistor Applications

**DOI:** 10.1021/jacs.4c16189

**Published:** 2025-01-14

**Authors:** Abhishek Pareek, Muhammad Yasir Mehboob, Maciej Cieplak, Maciej Majdecki, Hubert Szabat, Krzysztof Noworyta, Piotr Połczyński, Maja Morawiak, Piyush Sindhu Sharma, Cina Foroutan-Nejad, Przemysław Gaweł

**Affiliations:** † Institute of Organic Chemistry, Polish Academy of Sciences, Kasprzaka 44/52, 01-224 Warsaw, Poland; ‡ Institute of Physical Chemistry, Polish Academy of Sciences, Kasprzaka 44/52, 01-224 Warsaw, Poland; § Department of Chemistry, Laboratory of Electroanalytical Chemistry, Biological and Chemical Research Centre, University of Warsaw, Żwirki i Wigury 101, 02-093 Warsaw, Poland

## Abstract

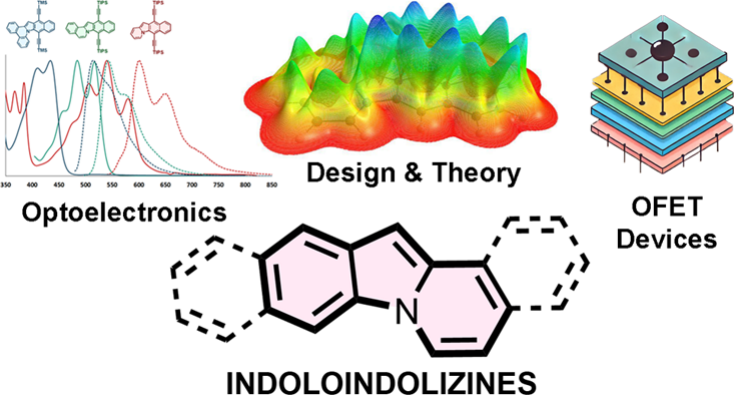

The development of stable and tunable polycyclic aromatic compounds (PACs) is crucial for the advancement of organic optoelectronics. Conventional PACs, such as acenes, often suffer from poor stability due to photooxidation and oligomerization, which are linked to their frontier molecular orbital energy levels. To address these limitations, we designed and synthesized a new class of π-expanded indoloindolizines by merging indole and indolizine moieties into a single polycyclic framework. We developed a scalable synthetic protocol to produce a wide range of π-expanded derivatives. The structural, electronic, and optical properties of these compounds were extensively characterized. We achieved precise modulation of the electronic structure by controlling the aromaticity of specific rings. Benzannulation at targeted positions allowed fine-tuning of the HOMO–LUMO gap, leading to distinct shifts in the optoelectronic properties. Single-crystal X-ray diffraction confirmed their molecular structures, while theoretical calculations provided insights into the observed experimental trends. These indoloindolizines exhibit vivid colors and fluorescence across the visible spectrum and enhanced stability against photooxidation. Reactivity studies demonstrated high regioselectivity in electrophilic substitutions, highlighting the indole-like behavior of these compounds and opening avenues for further functionalization. To showcase the practical utility of our design rules, we fabricated organic field-effect transistors (OFETs) using the newly developed indoloindolizines, which revealed remarkable performance with ambipolar charge transport properties. Overall, our work establishes indoloindolizines as a promising platform for the development of stable, tunable organic materials for optoelectronic applications. Through rational molecular design, we have provided a new pathway for molecular innovation in organic electronics.

## Introduction

The advancement of organic optoelectronics relies heavily on polycyclic aromatic compounds (PACs), which act as fundamental components in these devices.^1−3^ Unfortunately, many PAC representatives, particularly acenes, suffer from poor stability. Major decomposition pathways, such as photooxidation with ^1^O_2_ and oligomerization, are closely linked to the HOMO and LUMO energy levels of acenes.^4,5^ Therefore, finding ideal systems for organic optoelectronic applications with tunable energy levels of frontier molecular orbitals, alongside maintaining good stability and solubility, is a highly challenging task.

One of the most effective strategies for enhancing the stability of PACs involves substituting some of their carbon atoms with main group element heteroatoms, such as sulfur (S), nitrogen (N), boron (B), and phosphorus (P).^6,7^ Such heteroatom substitution not only significantly improves their stability but also endows the resulting polycyclic heteroaromatic compounds with a broad spectrum of properties that cannot be attainable by their purely hydrocarbon analogues.

**Figure 1:**
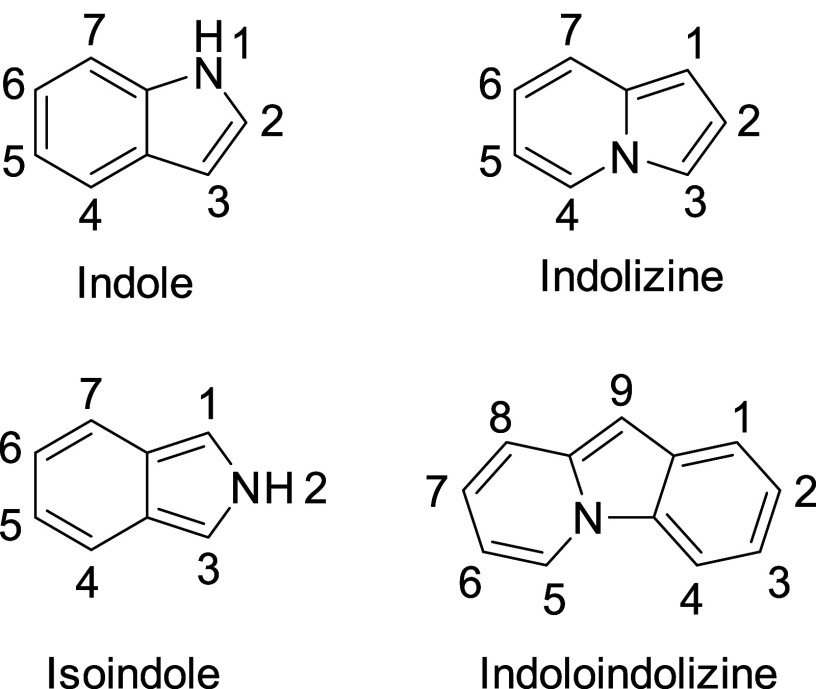
Schematic structures of indole, indolizine, isoindole, and indoloindolizine together with the IUPAC numbers of ring positions. The HOMO–LUMO gaps of indolizine and isoindole are 0.88 and 0.89 eV lower than those of indole, respectively (Section S6).

Besides fine-tuning the electronic structure, heteroatoms can improve molecular packing and processability, opening new avenues for material design and application. Thiophenes and azaacenes, in particular, have been the subject of extensive research due to their remarkable potential in organic electronic devices for their transformative impact on the performance and functionality of these materials.^8−11^ Derivatives of indole and its isomers, isoindole and indolizine, are among the less studied PACs for organic optoelectronics (Figure 1). A recent theoretical investigation by Pino-Rios and Solà has cast these molecules in a new light, revealing significant insights into their relative stability.^12^ Their research uncovered a pronounced disparity in the distribution of local aromaticity within these structures, which is influenced by the positioning of the nitrogen atom. The 0.88 eV reduction in the HOMO–LUMO energy gap of indolizine compared to that of indole is attributed solely to the variations in nitrogen position within their frameworks. Pino-Rios and Solà concluded that the relatively lower HOMO–LUMO energy gaps and higher energies of isoindole and indolizine result from the reduction of the aromaticity in the six-membered ring.^13^

Such striking differences between indole and its isomers offer a unique avenue for tailoring the optoelectronic properties of polycyclic aromatic compounds through the strategic incorporation and substitution of these heterocycles. While π-expanded derivatives of indole have been the subject of considerable study,^14−16^ the integration of indolizines into polycyclic aromatic frameworks remains surprisingly sparse.^17,18^ In this context, we have embarked on a quest to merge indole and indolizine within a single polycyclic molecular architecture, which we coined indoloindolizine (Figure 1), aiming to enable the precise modulation of the electronic structure for optoelectronic applications, in particular, stable organic field-effect transistors (OFETs). We elucidate molecular design principles aimed at adjusting the HOMO–LUMO gap by the interplay between indole- and indolizine-type character. We developed a straightforward and efficient synthesis methodology for a diverse array of π-expanded derivatives and conducted a thorough investigation of their optoelectronic properties. The study is further enriched by extensive theoretical analyses that shed light on the seemingly counterintuitive phenomenon of an increasing HOMO–LUMO gap with an expanding conjugated π-system.

## Results and Discussion

### Design Strategy

In a recent study, Pino-Rios and Solà demonstrated that the relative stabilities of isoindole and indolizine decrease because the aromaticity of the six-membered rings in these compounds is reduced compared to that of indole. The greater aromaticity of indole originates from a unique resonance structure that allows both the five- and six-membered rings to maintain six π-electrons each. Apart from energetic stability, aromaticity is known to be connected to hardness, which is defined by the HOMO–LUMO energy gap of molecules. Indole’s HOMO–LUMO gap is indeed 0.88 eV higher than that of indolizine, aligning with the hardness criterion of aromaticity.^19,13^ Ideally, by adjusting the indole- or indolizine-like characteristics, one could tune the HOMO–LUMO energy gap in molecules that incorporate both systems.

Here, we focus on polycyclic aromatic derivatives of indoloindolizine, where both indole and indolizine moieties are embedded in the core structure. We propose that by intensifying the aromaticity of the indolizine moiety, hence increasing the indolizine-likeness, in the molecule, we can reduce the HOMO–LUMO gap. On the other hand, by weakening the aromaticity of the indolizine framework, thus increasing the indole-likeness, we can deliberately increase the HOMO–LUMO gap. Following the Pino-Rios and Solà findings, we particularly focused on dearomatizing the six-membered ring in the indolizine moiety through benzannulation to achieve highly conductive yet stable organic semiconductors suitable for application in electronic devices. In OFET applications, a lower HOMO–LUMO gap can facilitate more efficient charge injection from the electrodes into the semiconductor layer, thereby improving carrier mobility and reducing the operating voltage needed for effective current flow.^20^

By the combined retrosynthetic and computational analysis, we selected a group of potentially interesting and synthetically accessible laterally π-expanded fused indoloindolizines for further exploration of their optoelectronic properties. Our synthetic protocol (*vide infra*) allows benzannulation on positions 2,3 (on the indole part of the molecule), 5,6, and 7,8 on the indolizine moiety. Therefore, further theoretical analysis is focused on derivatives that are synthetically attainable. To examine our hypothesis, we computed HOMO–LUMO gaps and assessed the aromaticity of all rings utilizing magnetically induced current density (MICD), nucleus-independent chemical shifts (NICS),^21−23^ and isomerization stabilization energy (ISE)^24^ at the DFT (B3LYP^25−28^/def2-TZVPP^29,30^) computational level. As a rule of thumb, chemists expect that the HOMO–LUMO gap in conjugated molecules shrinks as the size of the conjugated π-system increases. This principle generally holds true for linear cata-condensed PACs. Indeed, a recent large-scale computational study by Gershoni-Poranne and co-workers confirmed a direct correlation between increasing π-system size and a reduction in the HOMO–LUMO energy gap.^31^ However, their work also revealed that the number of rings is not the sole factor determining the HOMO–LUMO gap. The distribution of Clar sextets within the π-system plays an equally important role. Indeed, in a series of heptacatafusenes—structural isomers of heptacene—the HOMO–LUMO gap varies dramatically, between 328 and 840 nm, depending on the number and arrangement of Clar sextets.^32^ s-Indacenes and pentalenes are additional remarkable cases where the benzannulation of inherently antiaromatic frameworks both enhances the stability and increases the HOMO–LUMO gap.^33−37^ Hence, not only the size but also the electronic structure and aromaticity patterns of the π-system must be considered when rationalizing and predicting electronic properties in PACs.

**Figure 2:**
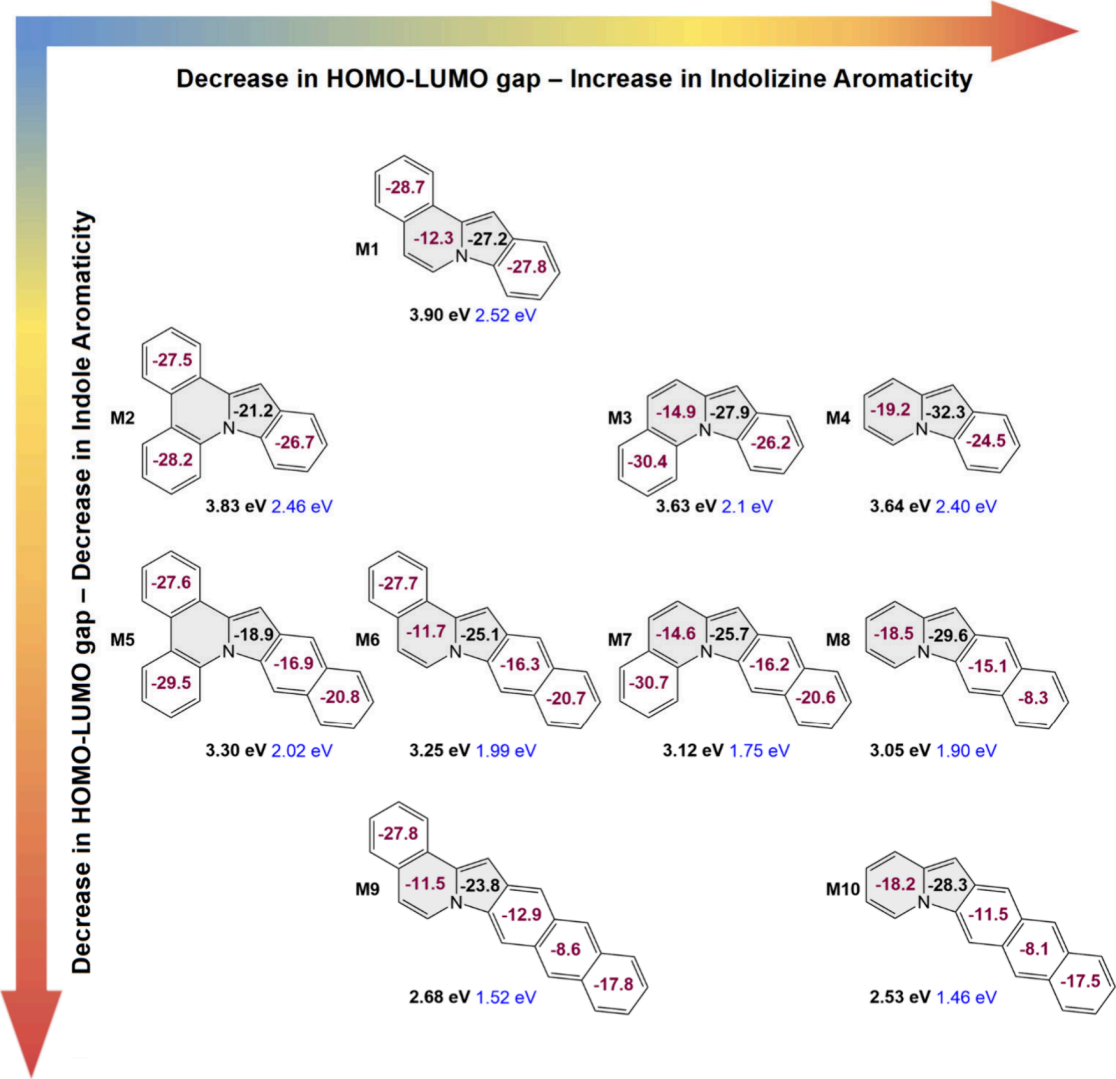
Periodic variation of the HOMO–LUMO gap in the indoloindolizine derivatives. The model systems **M** are numbered from **M1** to **M10**, generally as their HOMO–LUMO gap decreases. The purple numbers in each ring denote the ISE of the ring. The black number in the five-membered ring provides the ISE for the whole indolizine moiety. Below each molecule, their HOMO–LUMO gaps computed at the DFT (black) and extended Hückel (blue) levels are presented.

The intrinsic difference between indole and indolizine makes the trends in the variation of the HOMO–LUMO gap complicated in the case of indoloindolizine derivatives. Nevertheless, listing the studied molecules according to their HOMO–LUMO gap reveals a structure-dependent periodic pattern in model compounds **M** (Figure 2; for a more comprehensive table, see Figure S63). To construct a periodic table for indoloindolizines, we followed four simple rules. (**1**) From the top left to the bottom right the HOMO–LUMO gap of molecules decreases. The only exceptions to this rule are **M3** and **M4** where DFT predicts the gap of **M4** to be 0.01 eV higher than that of **M3**, but for the sake of the fourth rule, we place **M3** before **M4** in the table. (**2**) We keep in the same row all molecules that have an equal or smaller number of rings than the first molecule in a row of the periodic table. (**3**) If a subsequent molecule has more rings than the first molecule on the left side of a row, then it should be placed in a lower row. (**4**) All molecules that have similar substitution patterns on their indolizine moiety are listed in a group below each other.

Inspecting the variation of the HOMO–LUMO gaps with respect to structure and aromaticity reveals an interesting trend. Within each row of our periodic table, we observe that as the HOMO–LUMO gap decreases, the isomerization stabilization energy (ISE) of the indolizine moiety increases. Notably, in each row, the ISE of the six-membered ring of the indole moiety remains almost constant. From the top to bottom of each group, where the number of attached benzene rings on the six-membered ring of the indolizine moiety is conserved, the addition of more benzene rings to the other side of the molecule has two effects. First, the aromaticity of the indolizine moiety decreases, as it is reflected in the decrease of the ISE of the indolizine fragment. Interestingly, the ISE of the six-membered ring of indole also decreases more rapidly than that of the six-membered ring of the indolizine moiety. These two simultaneous changes point to the loss of the aromatic stabilization energy in the indole unit in each group from top to bottom. Concomitantly, in each group, the HOMO–LUMO gaps decrease from top to bottom. Akin to the periodic table of elements, one may fill the gaps in the periodic table presented in Figure 2. A more comprehensive version of this periodic table with substituted indoloindolizines is presented in Figure S63. Inspecting the current density passing through the five-membered ring of these systems reveals a similar trend but with less sensitivity to the HOMO–LUMO gap variation trend. The current density values for all studied systems are collected in Figure S64 for comparison with those of the ISEs. Our initial analysis suggests that double benzannulation of positions 5,6 and 7,8 on the indolizine moiety is the most efficient method to increase the HOMO–LUMO gap, as **M2** has the largest gap in its row. Monobenzannulation in position 7,8 gives a slightly higher HOMO–LUMO gap than in the corresponding isomers with a benzene ring added to the 5,6 position of the indoloindolizine moiety. Finally, benzannulation of the indole moiety increases the ISE of the indolizine part, which results in lowering the HOMO–LUMO gap, as seen on the right side of our indoloindolizine periodic table (Figure 2).

### Synthesis

The synthetic strategies for π-expanded indolizines and indoles flourished in recent years.^18^ Despite this advancement, the lateral expansion of their polycyclic frameworks remains a relatively underexplored avenue, with only a few reported methods of limited scalability.^17,38−40^ To map the largely unexplored chemical space of linear polycyclic indoloindolizines, our objective was to establish a straightforward and scalable synthetic approach for these promising molecular architectures. Drawing inspiration from Dalcanale’s pioneering synthesis of naphtho[2,3-*b*]indolizine-6,11-dione,^41^ we devised a solution-based protocol for a reaction between methylquinones and pyridine derivatives (Table 1). Among the tested reagents and polar aprotic solvents needed to stabilize the postulated highly polar intermediates, DMSO at elevated temperatures, along with 2 equiv of elemental iodine, yielded the best results in a test reaction between pyridine and 2-methylnaphthoquinone. This protocol enables the use of solid substrates, including π-expanded variants, which significantly broadens the reaction scope compared to Dalcanale’s protocol. Applying these conditions to other methylquinones and pyridines, we obtained a series of π-expanded indolizinequinones **IQ** in fair to good yields (Table 1). Interestingly, while isoquinoline reacted readily, forming systems benzannulated at positions 7 and 8 of indoloindolizine, quinoline, which would result in benzannulation at positions 6 and 7, did not react at all. This lack of reactivity is likely due to the poor reactivity of the C–H positions adjacent to the nitrogen in quinoline, which prevents ring closure and forming indoloindolizine.

**Table 1:**
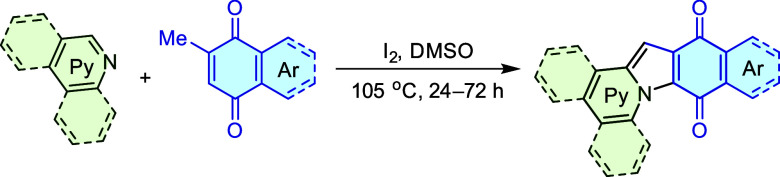
Synthesis of Indolizinequinones **IQ**^a^

Py	Ar	Product Code	Time	Yield
IsoQuinoline	2,3-Dimethyl	IQ1	36 h	31%
Pyridine	2,3-Dimethyl	IQ4	36 h	18%
Phenanthridine	Benzene	IQ5	72 h	17%
IsoQuinoline	Benzene	IQ6	24 h	52%
Pyridine	Benzene	IQ8a	48 h	67%
3-Br-Pyridine	Benzene	IQ8b	36 h	70%
3-F-Pyridine	Benzene	IQ8c	48 h	30%
4-CF_3_–Pyridine	Benzene	IQ8d	36 h	46%
IsoQuinoline	Naphthalene	IQ9	48 h	47%
Pyridine	Naphthalene	IQ10	48 h	82%

a Numbering corresponds to the position in the periodic table in Figure 2.

Indolizine-containing quinones **IQ** were further functionalized with trialkylsilylacetylenes using a streamlined one-pot protocol. The process commenced with the butyllithium-mediated addition of acetylene derivatives to the carbonyl groups of the quinones. The adducts generated from this reaction exhibited relative instability during purification attempts. Consequently, to circumvent this instability, a reductive elimination step was performed in the same reaction vessel. This was accomplished by introducing SnCl_2_ along with a 10% aqueous HCl solution to give the desired π-expanded indoloindolizines **I** in fair to good yields. For **I5** and **I10**, the additions of TIPS-acetylene did not give the desired bisadduct, presumably because of the steric clash between the large TIPS group and the rest of the molecule. TMS-acetylene was used instead to give the desired products in acceptable yields (Table 2).

**Table 2:**
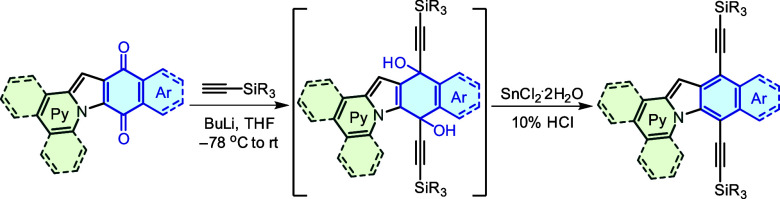
Synthesis of Indoloindolizines **I**^a^

IQ	Py	Ar	SiR3	Product Code	Yield
IQ1	Isoquinoline	2,3-dimethyl	TIPS	I1	38%
IQ4	Pyridine	2,3-dimethyl	TMS	I4	34%
IQ5	Phenanthridine	Benzene	TMS	I5	41%
IQ6	Isoquinoline	Benzene	TIPS	I6	55%
IQ8a	Pyridine	Benzene	TIPS	I8a	30%
IQ8b	3-Br-Pyridine	Benzene	TIPS	I8b	83%
IQ8c	3-F-Pyridine	Benzene	TIPS	I8c	60%
IQ8d	4-CF_3_-Pyridine	Benzene	TIPS	I8d	61%
IQ9	Isoquinoline	Naphthalene	TIPS	I9	61%
IQ10	Pyridine	Naphthalene	TMS	I10	30%

a Numbering corresponds to the position in the periodic table in Figure 2. TMS = trimethylsilyl; TIPS = triisopropylsilyl.

### Structural Characterization

**Figure 3:**
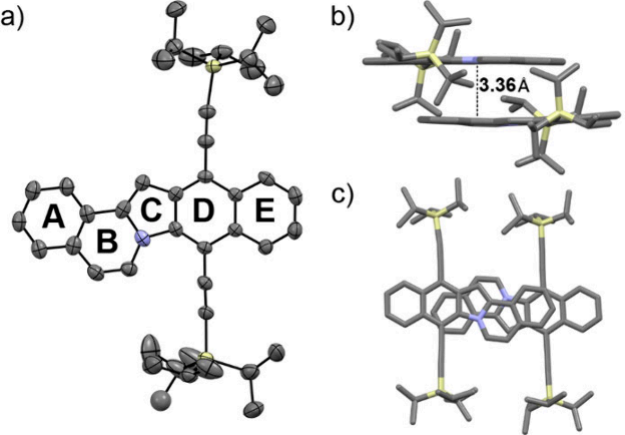
a) ORTEP plot for the single crystal structure of **I6**. Atomic displacement parameters at 160 K are drawn at the 50% probability level. b) Side and c) top views of antiparallel packing within dimers of **I6**. H atoms have been omitted for the sake of clarity. Atom colors: gray, C; blue, N; and yellow, Si.

The structure of indoloindolizine **I6** was unambiguously confirmed with a single-crystal X-ray diffraction analysis (Figure 3). In the crystal, **I6** forms dimeric structures in an antiparallel configuration, which are organized within the crystalline matrix into two distinct domains oriented at an angle of approximately 65° relative to each other. The polycyclic cores of these dimers are situated a mere 3.36 Å apart. Such a compact intermolecular distance is presumably a consequence of antiparallel dipole–dipole interactions, driven by the intrinsic dipole moments inherent in the indoloindolizine’s asymmetric polycyclic framework. A detailed examination of bond lengths within the polycyclic core of **I6** reveals a pronounced delocalization, evidenced by a small bond-length alternation (BLA), within ring A (Figure 3). Conversely, rings B–E exhibit more localized electronic environments, as indicated by increased BLA values (Figure S22). These differences suggest the presence of distinct aromatic substructures within these segments (*vide infra*). It is worth noting that the observed BLA in **I6** is consistent with the computed ISE values in **M6**, where ring A has the highest ISE value among all of the rings (Figure 2).

### Optoelectronic Properties

**Figure 4:**
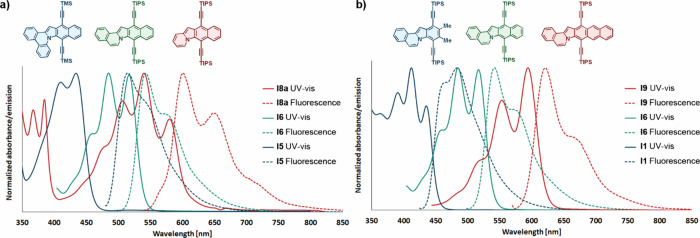
Normalized UV–vis and fluorescence spectra of a) **I5 I6 I8a** and b) **I1 I6 I9** showing trends in the spectral shift upon structural alterations of the indoloindolizine core.

Indoloindolizines, as representatives of a new class of polycyclic heteroaromatic dyes, exhibit vivid colors and fluorescence across the entire visible spectrum (Figure 4; see also Section S3 for spectra of all obtained derivatives). The observed trends in spectral changes caused by structural modifications align closely with the design principles described above. Specifically, linear benzannulation of the indoloindolizine core at the 2,3-positions leads to a significant bathochromic shift of the lowest energy absorption, reaching 150 nm when comparing compound **I1** to **I9** (as shown in Figure 4b). This phenomenon is readily explained by Hückel’s molecular orbital theory, which posits that expanding the conjugated π-system reduces the HOMO–LUMO energy gap. A recent analysis of the COMPAS-2 database of over half a million molecules shows clearly that this trend is general for cata-condensed heteroaromatic polycyclic compounds.^31^ In contrast, adding phenyl rings at the 5,6 and 7,8 positions of indoloindolizine results in a notable hypsochromic shift of up to 150 nm when moving from compound **I8a** to compound **I5** (Figure 4a). This effect is less intuitive since expanding the conjugated system typically reduces the HOMO–LUMO gap. Nonetheless, extended Hückel molecular orbital theory accurately predicts this trend, showcasing its exceptional utility in molecular design (Figure 2). Time-dependent density functional theory (TD-DFT) computations have confirmed that the lowest-energy absorption bands in all studied compounds originate from transitions from HOMO to LUMO (Section S6). Additionally, visualization of frontier molecular orbitals shows a rather uniform distribution of electron density over the indoloindolizine core for both HOMO and LUMO, which indicates that these are π–π* transitions and together with TD-DFT results suggests that the shift is not related to the presence of symmetry-forbidden transitions (Section S6). Despite the good prediction of spectral trends, the TD-DFT results did not explain the reason behind such behavior.

**Figure 5:**
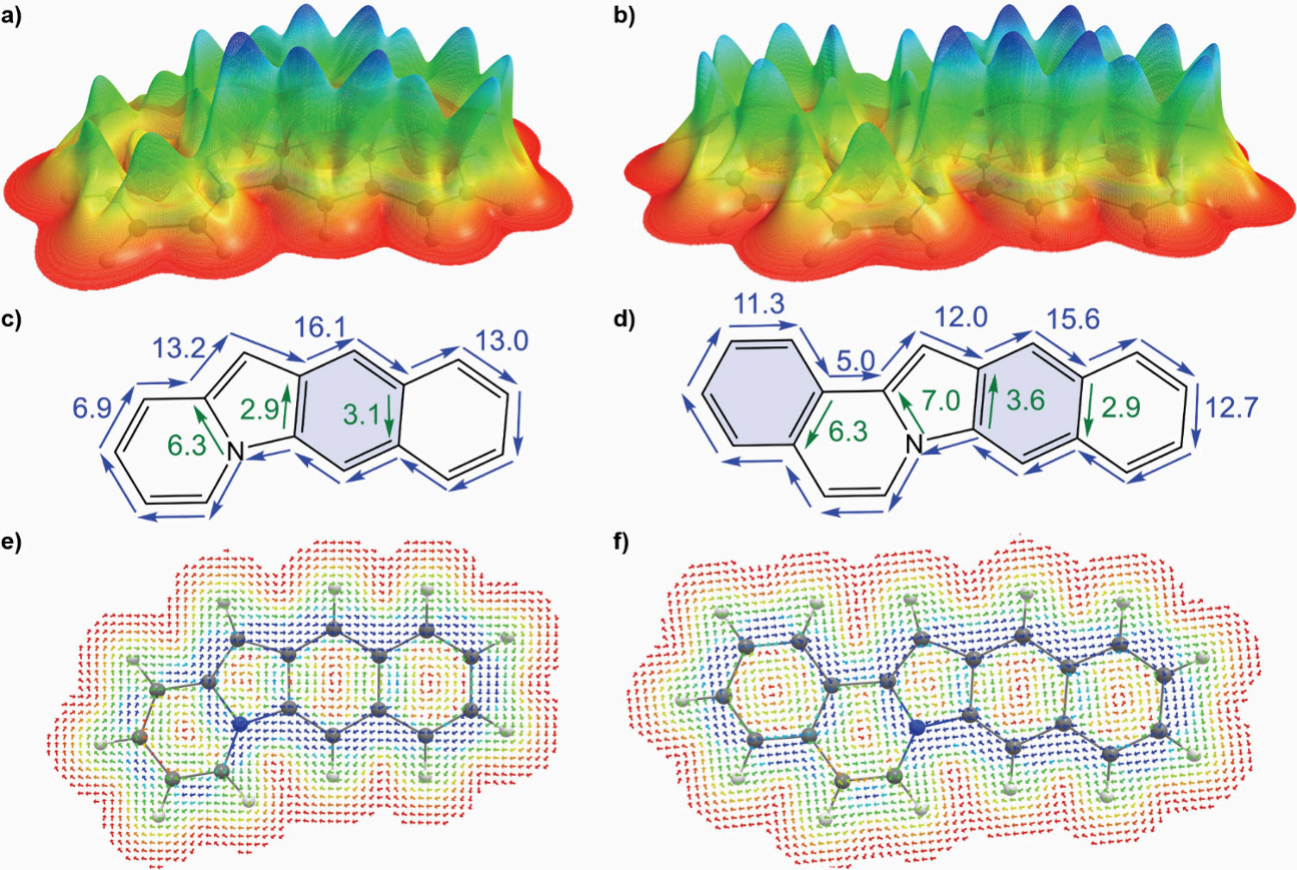
Current density profiles of two model systems (**M6** and **M8**). (a and b) Relief maps present the distribution of the current density computed for the plane 1 Bohr (approximately 0.53 Å) above the molecular plane, where the π ring current is most intense. The height and colors are proportional to the current intensity at each point. (c, d) Integrated current density values for planes perpendicular to the bonds and the direction of the current in nA/T. (e and f) Vector map of the current density 1 Bohr (approximately 0.53 Å) above the molecular plane above the ring plane of the molecules. The colors are selected to resemble those in a and b. In **M8** (a, c, e) and **M6** (b, d, f), the six-membered ring in the indolizine moiety has a lower current density than its counterpart in indole. Benzannulation on position 7,8 in indoloindolizine (b, d, f) introduces a ring with local current, e.g., a Clar sextet, which is separated from the indole moiety within the framework of indoloindolizine.

To elucidate the foundation of the observed structure–property relationships, we conducted ring current analysis on the polycyclic cores of these compounds (detailed in Section S6). These calculations demonstrated that the addition of phenyl rings at the 2,3-positions integrates them into the global polycyclic aromatic ring current, akin to the benzannulation process observed in acenes. On the other hand, attaching phenyl rings to indoloindolizine at the 5,6 and/or 7,8 positions results in the formation of distinct localized ring current loops. Additionally, it separates the six-membered ring of indolizine from the main polycyclic system’s global ring current, promoting overall indol character. Consequently, this isolation gives rise to smaller aromatic subsystems (chromophores), which in turn leads to a hypsochromic shift in the electronic absorption spectra (Figure 5).

The fluorescence spectra of studied indoloindolizines follow similar trends to their absorption spectra (Figure 4; see also Section S3 for spectra of all derivatives). However, the vibronic fine structures of the absorption bands are not perfectly reflected in the emission spectra of most derivatives. This suggests a difference in the vibronic coupling between ground and excited states.^42^ On the other side, the Stokes shift of most derivatives is relatively low (approximately 25 nm), which indicates high structural rigidity of the polycyclic aromatic core. A higher Stokes shift for **I5** is presumably the result of its slightly bent structure caused by the steric clash between the phenanthridine moiety and the TMS-acetylene substituent. Fluorescence quantum yields vary significantly depending on the structure of the polycyclic core. Compound **I8a** and its derivatives, where one can identify only one Clar’s sextet and one global delocalized aromatic ring current loop, exhibit low to very low quantum yields (Table 3). On the other side, derivatives expanded on the 5,6 and 7,8 positions of indoloindolizine, with more localized ring current loops, show significantly higher quantum yields. Such a change in the photoluminescence quantum efficiency indicates the presence of nonradiative relaxation pathways in the former ones, which will be a topic of investigation in our future studies.

**Table 3:** Summary of the Optoelectronic Properties of Selected Indoloindolizines

	UV–vis		Fluorescence				DFT	Electrochemistry (DPV)		
Compound	λ_abs_ nm	eV	λ_em_ nm	eV	Stokes Shift eV	Φ_PL_ %	Δ*E* TDDFT	Eox V vs Fc/Fc^+^	Ered V vs Fc/Fc^+^	Δ*E* V vs Fc/Fc^+^
**I8a**	580	**2.13**	600	2.07	0.06	3	**2.58**	–0.21	–2.18	**1.97**
**I6**	517	**2.39**	542	2.29	0.10	16	**2.76**	0.37	–1.94	**2.31**
**I5**	434	**2.84**	515	2.41	0.43	75	**2.92**	0.38	–2.11	**2.49**
**I1**	435	**2.82**	464	2.67	0.15	16	**3.38**	0.35	–1.76	**2.78 (CV)**
**I9**	594	**2.07**	621	2.00	0.07	17	**2.37**	0.13	–1.94	**2.07**

To thoroughly evaluate the redox properties of the newly synthesized indoloindolizines, we performed electrochemical measurements using both cyclic voltammetry and differential pulse voltammetry (Table 3, Section S4). These complementary techniques provided detailed insights into the oxidation and reduction behavior of the chromophores, allowing for a precise determination of their redox potentials. The estimated HOMO–LUMO energy gaps, calculated from these redox potentials, followed the trends established by optical spectroscopy and theoretical investigations (Figure S29). This consistency further validates our chromophore design strategy.

### Reactivity

The integration of the indoloindolizine framework into polycyclic systems significantly influences the reactivity of these compounds. The most remarkable attribute observed, particularly compared with acenes, is the enhanced stability against photooxidation. The majority of the compounds we investigated exhibited notable resilience, remaining stable in solution under exposure to sunlight and air for days. In a controlled experiment, where solutions of these compounds were aerated and subjected to 254 nm UV light irradiation, the indoloindolizines demonstrated stability that surpassed traditional organic electronic benchmarks—such as for TIPS-tetracene and TIPS-pentacene—by at least an order of magnitude (Section S3). This shows the potential of indoloindolizine-based materials in extending the durability and performance of organic electronic devices.

**Figure 6:**
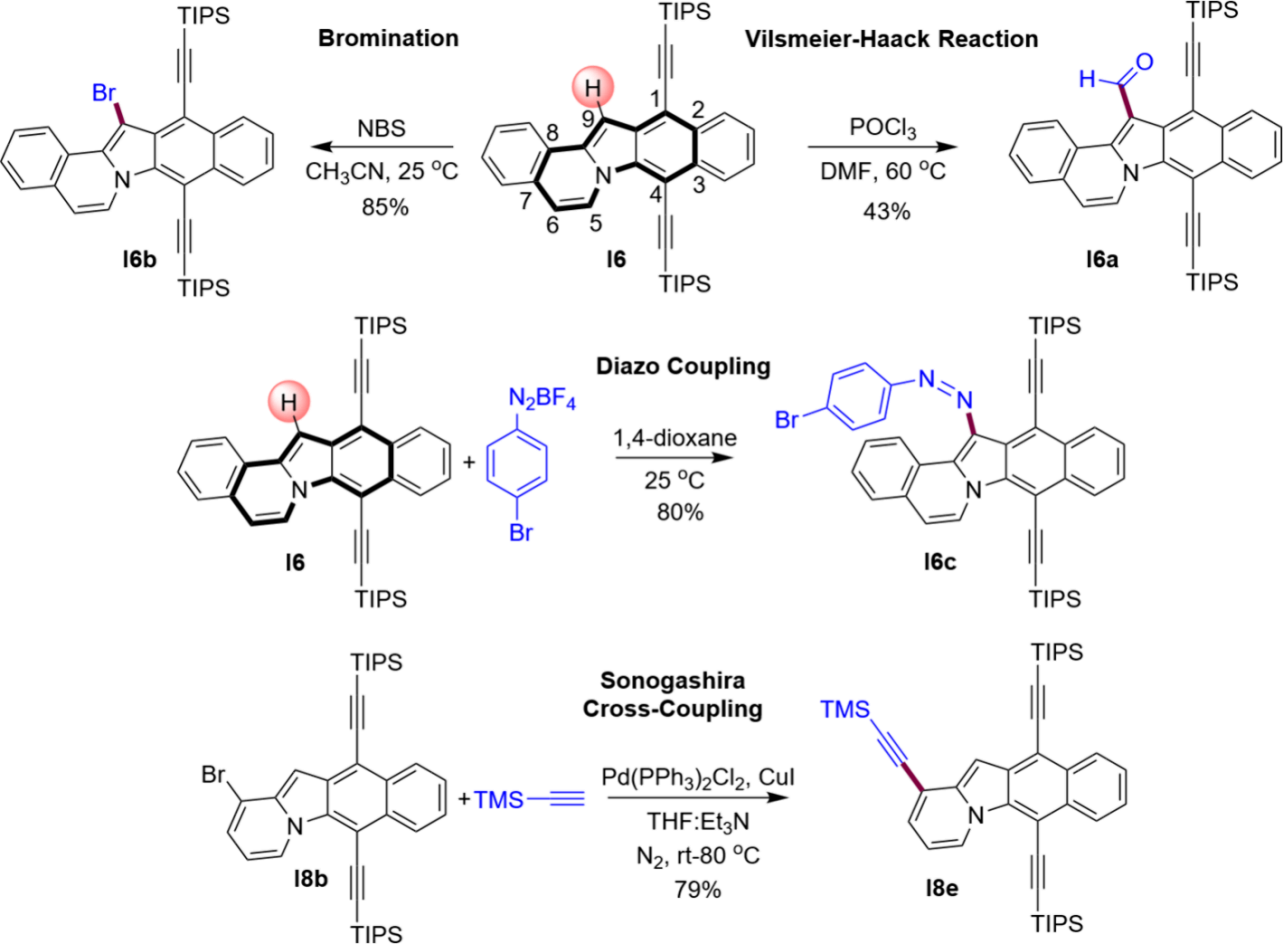
Examples of indoloindolizines’ reactivity.

Furthermore, the indole-type nature of indoloindolizines is manifested in their reactivity. In electrophilic substitution, **I6** reacts exclusively at the pyrrole C–H position (position 9 of the indoloindolizine moiety, Figure 6). Such high regioselectivity of these reactions can also be explained by aromatic stabilization energy where the positive charge localizes in the transition state on the ring that is already less aromatic, which in this case is the pyrrole ring. Hence, compound **I6** gave the corresponding aldehyde **I6a** under the Vilsmeier–Haack reaction conditions. Additionally, **I6** undergoes bromination with N-bromosuccinimide, producing C9-brominated indoloindolizine **I6b** in a high yield.

Expanding the scope of our reactivity investigation, **I6** was also subjected to an azo coupling reaction with 4-bromobenzenediazonium tetrafluoroborate salt, affording *cis*-azo-indoloindolizine **I6c**. Interestingly, **I6c** does not switch under light irradiation, presumably because of the large steric hindrance imposed by the adjacent TIPS-acetylene group. Furthermore, we expanded the synthetic scope of this new class of compounds by attaching TMS-acetylene to **I8b** under Sonogashira cross-coupling conditions giving **I8e**, which shows that **I8b** can readily be functionalized at this position with a broad range of substituents via cross-coupling chemistry.

### Organic Field-Effect Transistors

Based on their optoelectronic properties and solid-state structures, we selected compounds **I6**, **I8a**, and **I9** to construct organic field-effect transistors (OFETs). These devices were fabricated by drop-casting their solutions onto platinum- or pentafluorobenzenethiol (PFBT)-functionalized gold-coated platinum structures on SiO_2_/Si substrates (details provided in Section S7).^43^ Scanning electron microscopy (SEM) images revealed a uniform and continuous distribution of the organic films within the gate channel (Figure S68), indicating effective film formation. The films exhibited smooth surfaces without noticeable defects or aggregation. Additionally, energy-dispersive X-ray spectroscopy (EDX) confirmed the electrode morphology by verifying its elemental composition.

**Figure 7:**
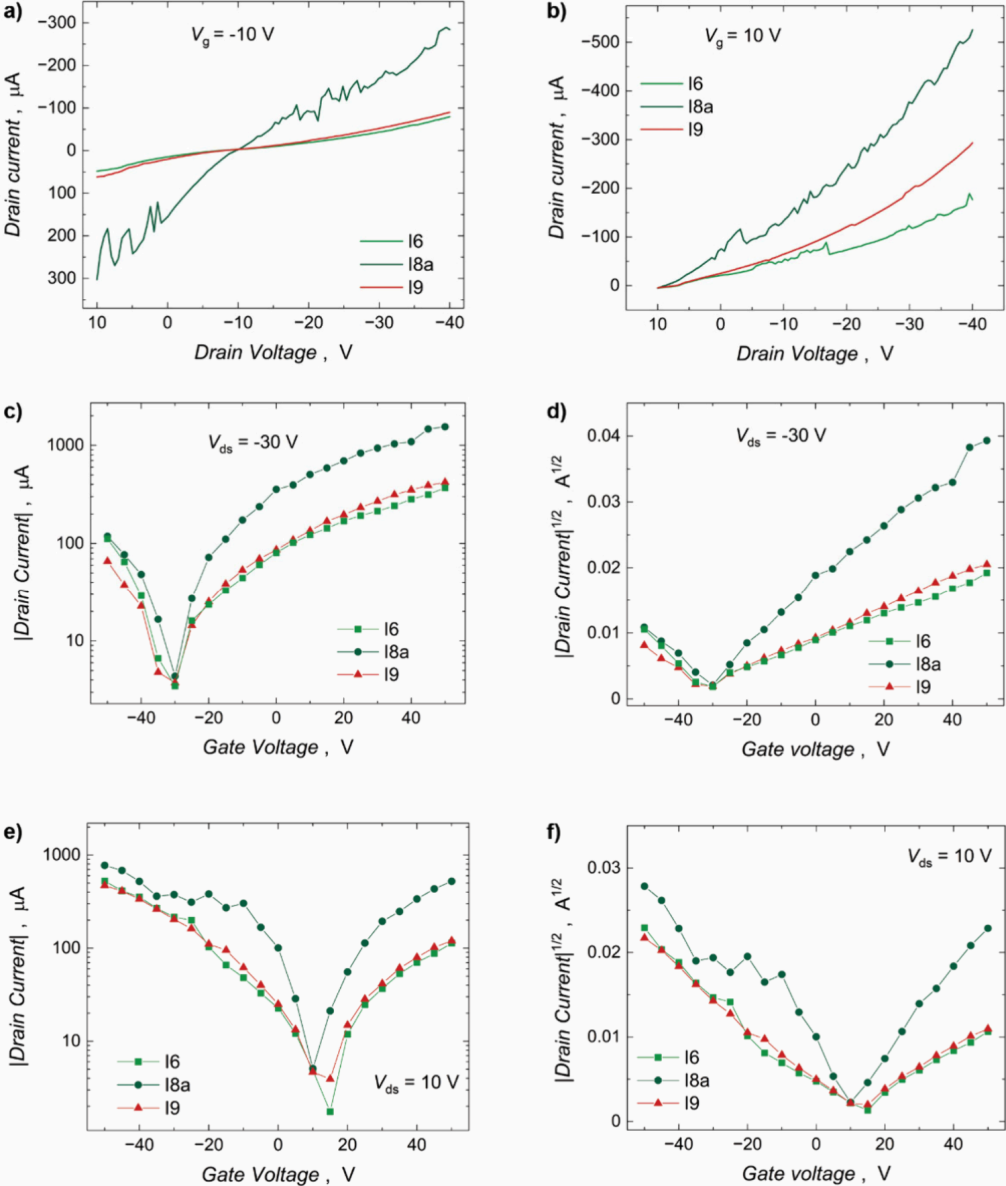
(a, b) Examples of the *I–V* characteristics recorded for the studied transistors at *V*_g_ values of (a) −10 and (b) 10 V. (c–f) Transfer characteristics of the studied compounds recorded at −30 and 10 V *V*_ds_ voltages. The gate width for the devices based on **I6** and **I9** was 10 μm, while that for **I8a** was 6 μm.

To evaluate the electrical performance of OFETs based on these indoloindolizine derivatives, we recorded current–voltage (I–V) characteristics and transfer curves at various gate voltages (Figure 7a,b, Section S7). The output characteristics (Figure 7a) demonstrate the ambipolar behavior of all measured compounds with the drain current modulated by the gate voltage. At low drain voltages, the nonlinear shape of the curves indicates contact resistance and suggests the presence of charge trap states in the materials.^44,45^ Additionally, the transfer characteristics for both negative and positive drain voltages (Figure 7c,d) are symmetric, indicating balanced electron and hole populations within the devices. The threshold voltages for p-type operation are approximately −30 V and are similar across all compounds, whereas for n-type operation, the threshold voltages range from 10 to 15 V, depending on the specific compound. These relatively high threshold voltages for both n- and p-channel operations may indicate charge injection limitations and strong charge carrier trapping at the semiconductor–dielectric interface,^45^ with hole trapping being more pronounced. Notably, the transfer characteristics of **I8a** reveal weaker electron trapping than those of the other derivatives (Figure 7c). However, further optimization of the silyl side groups and deposition processes is needed to reduce charge trapping, which will be the focus of future studies.

Comparing the transfer characteristics of OFETs prepared with the three studied indoloindolizine derivatives (Figure 7d and 7f), it is clear that devices based on **I6** and **I9** exhibit quite similar behavior. Based on the dependence of the square root of the source-drain current on the gate voltage (Figure 7d and 7e), charge carrier mobilities for both holes and electrons were calculated (Table S4). The obtained values for hole mobility range from 0.21 to 0.49 cm^2^ V^–1^ s^–1^, while electron mobilities range from 0.11 to 0.29 cm^2^ V^–1^ s^–1^. These mobility values are comparable to those reported for current small-molecule organic semiconductors, suggesting that indoloindolizine derivatives are suitable candidates for application in electronic devices.^20,46−48^ Interestingly, for both **I6** and **I9**, the hole mobility is higher than the electron mobility, with the electron mobility being identical for both compounds. The hole mobility for **I6** is the highest among those of the studied compounds. In contrast, the device based on **I8a** shows nearly identical hole and electron mobilities. This may stem from variations in charge trapping among the studied compounds. The hole trapping effect, as inferred from the threshold voltage variation, is similar for all three compounds, while electron trapping is less pronounced for **I8a** than for the other two compounds, thus less affecting the overall electron mobility in this compound. Notably, the HOMO and LUMO energy levels for **I8a**, as determined from electrochemical experiments, are less negative compared to those of the other two compounds, which affect electron and hole formation.

## Conclusions

We have successfully designed, synthesized, and thoroughly characterized a new class of chromophores based on an indoloindolizine framework. We strategically merged indole and indolizine moieties into a single polycyclic aromatic system. Through targeted benzannulation, we deliberately adjusted the aromatic character of the indolizine moiety, fine-tuning the HOMO–LUMO gap by intensifying or diminishing the aromaticity of specific rings. This approach allowed us to precisely control the electronic and optical properties of the chromophores.

Our straightforward and scalable synthetic methodology enabled the efficient production of a diverse array of π-expanded derivatives. Systematic investigation of their optoelectronic properties revealed intense colors and fluorescence throughout the visible range. The observed spectral trends closely aligned with our design principles and were corroborated by electrochemical measurements and theoretical calculations, including time-dependent density functional theory (TD-DFT) and ring current analyses. Reactivity studies highlighted the indole-like behavior of indoloindolizines, demonstrating high regioselectivity in electrophilic substitutions and opening avenues for further functionalization.

Furthermore, the enhanced stability of these compounds against photooxidation compared to that of acenes underscores their potential for practical applications. We validated this potential by integrating them into organic field-effect transistor (OFET) devices, where they exhibited ambipolar semiconducting behavior with good hole and electron mobilities, ultimately showcasing the utility of our design approach. Overall, our findings establish indoloindolizines as a promising platform for the development of stable and tunable organic materials. By establishing rational molecular design guidelines, we have advanced the field of organic optoelectronics and opened new opportunities for device applications.

## Supporting Information Available

General experimental methods, synthetic protocols, electronic spectra, stability experiments, electrochemical data, details of the X-ray crystallographic analysis, descriptions of quantum chemical calculations, extended theoretical results on optoelectronic properties, aromatic ring currents, aromatic stabilization energies, OFET device fabrication and characterization procedures, and ^1^H and ^13^C NMR spectra (PDF)
Cartesian coordinates of all theoretically studied molecules in xyz format (ZIP)
