# Supplementary material for: Indoloindolizines: The Complete Story of a Polycyclic Aromatic Scaffold from Theoretical Design to Organic Field-Effect Transistor Applications
Source: J Am Chem Soc. 2025 Jan 14;147(7):5996–6005. doi: 10.1021/jacs.4c16189 (PMC11848911; doi:10.1021/jacs.4c16189)
Supplement: Supplementary file 1 — ja4c16189_si_001.pdf [file ja4c16189_si_001.pdf]

Supporting Information

Indoloindolizines: A Complete Story of Polycyclic Aromatic Scaffold from Theoretical Design to Organic Field-Effect Transistor Applications.

Abhishek Pareek,[a] Muhammad Yasir Mehboob,[a] Maciej Cieplak,[b] Maciej Majdecki,[a] Hubert Szabat,[c] Krzysztof Noworyta,[b] Piotr Połczyński,[c] Maja Morawiak,[a] Piyush Sindhu Sharma,[b] Cina Foroutan-Nejad,[a] Przemysław Gaweł[a]**

[a] Institute of Organic Chemistry, Polish Academy of Sciences, Kasprzaka 44/52, 01-224 Warsaw, Poland

[b] Institute of Physical Chemistry, Polish Academy of Sciences, Kasprzaka 44/52, 01-224 Warsaw, Poland

[c] Department of Chemistry, Laboratory of Electroanalytical Chemistry, Biological and Chemical Research Centre, University of Warsaw, Zwirki i Wigury 101, PL-02-093 Warsaw, Poland

Table of Contents

S1. Materials and General Methods 2

S2. Synthetic Protocols 4

S3. Photophysical Measurements and Stability Studies 21

S4. Electrochemistry 24

S5. X-Ray Crystallographic Analysis 33

S6. Quantum Chemical Calculations 35

Calculations of optoelectronic properties 35

Calculations of Aromatic Ring Currents and Nucleus Independent Chemical Shifts (NICS) 38

Calculations of aromatic stabilization energy 49

S7. Organic Field Effect Transistor (OFET) device fabrication and characterization 70

S8. ^{1}H and ^{13}C NMR Spectra 74

S9. References 120

S1. Materials and General Methods

Reagents (Acros, Aldrich, ABCR, and TCI) were purchased as reagent grade and used without further purification.

Solvents for extraction or column chromatography were used analytical grade.

Dry solvents (THF, CH_2Cl_2, diethyl ether, and toluene) for reactions were purified by a solvent drying system from MBraun under nitrogen atmosphere (H_2O content < 10 ppm as determined by Karl-Fischer titration). All other solvents were purchased in p.a. quality.

Reactions in the absence of air and moisture were performed in oven-dried glassware under Ar atmosphere.

Flash column chromatography (FC) was performed using Biotage® Selekt apparatus at 25 °C with a head pressure of 0.0–30 bar and Flow Rate (50-250 mL/min). SiO_2 (60 Å, 230-400 mesh, particle size 0.040-0.063 mm, Fluka). The used solvent compositions are reported in synthetic procedures.

Analytical thin layer chromatography (TLC) was performed on aluminium sheets coated with silica gel 60 F254 (Merck, Macherey-Nagel). Visualization was achieved using UV light (254 or 365 nm).

Evaporation in vacuo was performed at 25–60 °C and 800–10 mbar.

Reported yields refer to spectroscopically and chromatographically pure compounds that were dried under high vacuum (0.5–0.1 mbar) before analytical characterization.

^{1}H and ^{13}C nuclear magnetic resonance (NMR) spectra were recorded on Bruker 400 (Avance III HD), Bruker DRX 500, Varian-Agilent 500 and Varian-Agilent 600 spectrometers at 400 MHz, 500 MHz or 600 MHz (^{1}H) and 75 MHz, 126 MHz or 150 MHz (^{13}C), respectively. Temperatures of measurements are indicated in the procedures and on the spectra. Chemical shifts δ are reported in ppm downfield from tetramethylsilane using the residual deuterated solvent signals as an internal reference ($CDCl_3$: δH = 7.26 ppm, δC = 77.0 ppm). For ^{1}H NMR, coupling constants *J* are given in Hz and the resonance multiplicity is described as s (singlet), d (doublet), t (triplet), q (quartet), m (multiplet).

High-resolution mass spectrometry (HR-MS) was performed by the Laboratory for Analysis of Bioactive Compoundsat the Institute of Organic Chemistry PAS on a AutoSpec Premier spectrometer (EI), on a 4000 Q-TRAP spectrometer (ESI) and (ACPI).

Melting Points All melting points for crystalline products were measured with automated melting point apparatus EZ-MELT and were given without correction.

Photophysical measurements in solution were performed at 298 K and conducted using a 10-mm square cell. UV–visible absorption spectra were recorded on a UV-visible spectrophotometer Shimadzu UV-1900i. The steady-state emission spectra and excitation spectra were measured with a spectrofluorometer (Edinburgh FL 980). Photoluminescence quantum yield (Φ_{Fl}) was determined using a comparative method[1] using 9,10-diphenylanthracene as a reference (Φ_{Fl} = 97%).[2]

General Procedures: All reactions were performed in glassware that had been flame-dried under vacuum or oven-dried overnight. All reaction temperatures are noted as the oil bath temperature, the internal temperature as monitored by a Teflon-coated thermocouple), or as the room temperature (approximately 23 °C). Solvents used for extraction were reagent grade, and chromatography solvents

were technical grade. Column chromatography was conducted using 230-400 mesh silica gel. Visualizations were accomplished by UV light, aqueous $KMnO_4$, ceric ammonium molybdate (CAM) solution, or iodine adsorbed on SiO_2.

X-Ray Crystallographic Analysis was performed on a Bruker X8 APEXII diffractometer using CuKα (λ = 1.54178 Å) radiation. Frames were integrated with the Bruker SAINT[3] package using a narrow-frame algorithm. The structure was solved and refined using the Bruker SHELXTL Software Package.[4] Data were corrected for absorption effects using the face-indexed numerical method (SADABS).[5] The structure was solved by direct methods SHELXS-2014 and refined with full-matrix least-squares calculations on F^2 using SHELX-2014.[6]

Crystallographic data have been deposited at the Cambridge Crystallographic Data Centre, 12 Union Road, 129 Cambridge CB21EZ, UK, and copies can be obtained on request, free of charge, by quoting the publication citation and the deposition number. CCDC number for **I6:** 2332189

Electrochemical measurements were carried out under inert atmosphere (Ar gas) in a glass cell in a three electrode setup. 50mM tetrabutylammonium perchlorate (TBAP) (≥98.0%, Aldrich; dried in a vacuum at 100°C for 12 hours) dissolved in CH_2Cl_2 was used as a supporting electrolyte. As working and counter electrode a Pt sheet was used. Before each experiment surface of platinum electrodes was purified by immersion in fresh "piranha solution" and thorough rinsing with mQ water. Subsequently Pt electrodes were dried in Ar atmosphere, and annealed in hydrogen-air flame and cooled down under Ar gas. As a reference electrode silver wire immersed in 10 mM silver perchlorate (Sigma, dried in a vacuum at 80°C) and 50 mM TBAP dissolved in acetonitrile (ACN) (HPLC grade, additionally three times distilled) was used. A two sintered glass bridge, filled with 50mM TBAP/ CH_2Cl_2, was used to avoid mixing of ACN and CH_2Cl_2. Reference electrode potential was determined after each experiment by addition of ferrocenium. All the potentials are given in reference to the ferrocene/ferrocenium couple redox potential.

Electrochemical experiments were measured on BioLogic SP-300 potentiostat with IR compensation. Cyclic voltammetry (CV) experiments were recorded starting from open circuit potential with sweep rate v = 100 mV/s. In differential pulse voltammetry (DPV) measurements working electrode potential was set and scanned towards desired value with pulses hight (P_H) and width equal (P_W) to ±2.5 mV and 100 ms respectively, and step time (S_t) and hight (S_H) 500 ms and ±5 mV, depending on anodic or cathodic polarisation, thus resulting in sweep rate of 10 mV/s. All electrochemical measurements were recorded at controlled temperature of –5°C in order to slow down dichloromethane evaporation.

Commercially available dichloromethane (CH_2Cl_2) (Roth, ROTISOLV® for HPLC) was purified according to the following procedure: CH_2Cl_2 was shaken several times with small (10% v/v) portions of concentrated, triply distilled, sulphuric acid until the aqueous layer remained colourless. Then DCM was shaken two times with saturated $Na_2CO_{3(aq)}$ and subsequently three times with ultrapure water (Milli-Q). Organic layer was stirred overnight with anhydrous $CaCl_2$. Afterwards, CH_2Cl_2 was heated to reflux with P_2O_5 for three hours under inert atmosphere (Ar BIP+), and then distilled at slow rate of 0.5 mL/min, discarding the first 20% and last 30% as forerunning and residue respectively. Such obtained solvent was stored under Ar gas in a sealed bottle.

All used glassware was purified using freshly prepared "piranha solution" (9:1 v/v mixture of concentrated sulphuric acid and 30% hydrogen peroxide respectively), then washed thoroughly with ultrapure mQ water, and finally dried in a high vacuum at 200°C for at least 48 hours.

S2. Synthetic Protocols

9,10-dimethylindolo[2,1-a]isoquinoline-8,11-dione (IQ1)

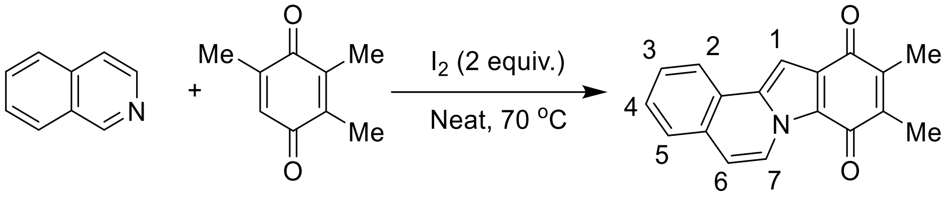

2a **1a** **IQ1**

Isoquinoline **2a** (645 mg, 0.58 mL, 4.99 mmol) and I_2, (507 mg, 2 mmol) were added to the 2,3,5-trimethylcyclohexa-2,5-diene-1,4-dione **1a** (150 mg, 1 mmol) in solvent free condition. The mixture was heated at 75 °C under stirring for 24 h than the minimum amount of CH_3CN was added, and the mixture was stirred for an additional 12 hours. Subsequently, the reaction mixture was diluted with $CHCl_3$, (10 mL) and washed with 10% HCl (10 mL), water (10 mL) and 5 % NaS_2O_3 solution (15 mL), organic layer dried over Na_2SO_4, and concentrated. The residue was purified using flash silica gel chromatography (hexanes:ethyl acetate 8:2) to **IQ1** (86 mg, 31%) as a reddish solid.

Rf: 0.6 (Hexane/EtOAc 8:2)

^{1}H NMR: (600 MHz, $CDCl_3$) δ 9.14 (d, J = 7.1 Hz, 1H; H–C (7)), 8.15 (d, J = 7.9 Hz, 1H; H–C (2)), 7.74-7.67 (m, 1H; H–C (1)), 7.61 (t, J = 7.5 Hz, 1H; H–C (3)), 7.59-7.53 (m, 1H; H–C (4)), 7.37 (s, 1H; H–C (5)), 7.17 (d, J = 7.4 Hz, 1H; H–C (6), 2.14 (s, 3H; Me), 2.11 ppm (s, 3H; Me)

^{13}C NMR: (151 MHz, $CDCl_3$) δ 184.40, 175.81, 144.66, 143.66, 140.29, 137.90, 128.82, 127.64, 124.36, 124.35, 120.99, 116.51, 116.50, 101.22, 12.60, 12.56 ppm.

HRMS: (APCI$^+$) Calcd for $C_{18}H_{13}NO_2$ $[M+H]^+$: 276.1025; found: 276.1024

mp: 273 °C

2,3-dimethylpyrido[1,2-a]indole-1,4-dione (IQ4)

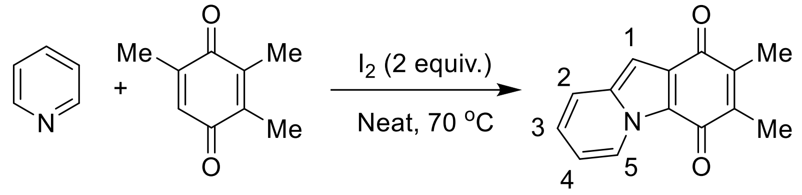

2b **1a** **IQ4**

Pyridine **2b** (395 mg, 4.99 mmol) and I_2, (507 mg, 2 mmol) were added to the 2,3,5-trimethylcyclohexa-2,5-diene-1,4-dione **1a** (150 mg, 1 mmol) in solvent free condition. The mixture was heated at 75 °C under stirring for 36 h. Then, the reaction mixture was diluted with $CHCl_3$, (10-15 mL) and washed with 10 % HCl (10 mL), water (10 mL) and 5 % NaS_2O_3 solution (10 mL), organic layer dried over Na_2SO_4, and concentrated. The residue was purified using flash silica gel chromatography (hexanes:ethyl acetate 8:2) to **IQ4** (40 mg, 18%) as a maroon solid.

Rf: 0.6 (Hexane/EtOAc 8:2)

^{1}H NMR: (600 MHz, $CDCl_3$) δ 9.39 (d, J = 7.1 Hz, 1H; H–C (5)), 7.59 (d, J = 8.9 Hz, 1H; H–C (2)), 7.18-7.08 (m, 1H; H–C (3)), 7.02-6.92 (m, 1H; H–C (4)), 6.88 (d, J = 0.8 Hz, 1H; H–C (1)), 2.13 (s, 3H; Me), 2.09 ppm (s, 3H; Me)

^{13}C NMR: (151 MHz, $CDCl_3$) δ 184.40, 175.81, 143.66, 140.29, 137.90, 128.82, 127.64, 124.36, 120.99, 116.51, 116.50, 101.22, 12.60, 12.56 ppm.

HRMS: ($APCI^+$) Calcd for $C_{14}H_{11}NO_2$ $[M+H]^+$: 226.0868; found: 226.0871

mp: 154 °C

Benzo[5,6]indolo[1,2-f]phenanthridine-10,15-dione (IQ5)

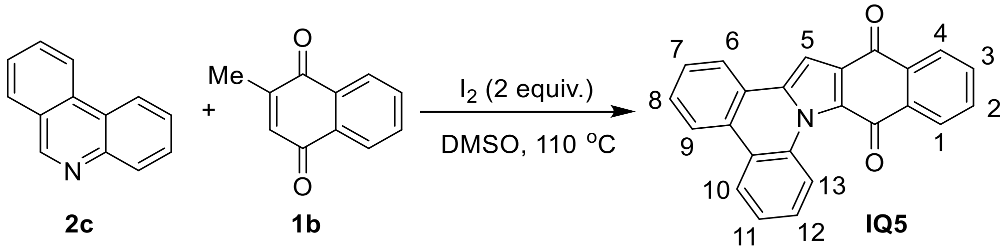

2c **1b** **IQ5**

Phenanthridine **2c** (625 mg, 3.48 mmol) and I_2, (590 mg, 2.32 mmol) were added to the solution of 2-methylnaphthalene-1,4-dione **1b** (200 mg, 1.16 mmol) in DMSO (3 mL). The mixture was heated at 110 °C under stirring for 72 h. Then, the reaction mixture was diluted with $CHCl_3$, (25-30 mL) and washed with 10 % HCl (10 mL), water (10 mL) and 5 % NaS_2O_3 solution (15 mL), dried over Na_2SO_4, and concentrated. The residue was purified using flash silica gel chromatography (hexanes:ethyl acetate 8:2) to **IQ5** (70 mg, 17%) as a brownish solid.

Rf: 0.5 (Hexane/EtOAc 8:2)

^{1}H NMR: (600 MHz, $CDCl_3$) δ 9.01 (d, J = 8.7 Hz, 1H; H–C (1)), 8.37 (dd, J = 15.6, 8.0 Hz, 2H; H–C (2, 3)), 8.29 (d, J = 7.6 Hz, 1H; H–C (4)), 8.22-8.14 (m, 2H; H–C (10, 13)), 7.76-7.57 ppm (m, 7H; H–C (5, 6, 7, 8, 9, 11, 12))

^{13}C NMR: (151 MHz, $CDCl_3$) δ 182.22, 173.93, 139.23, 136.45, 134.04, 133.55, 132.86, 132.79, 132.75, 129.47, 128.91, 128.24, 127.97, 127.73, 126.93, 126.33, 124.85, 124.47, 123.97, 123.59, 122.72, 122.03, 102.91 ppm.

HRMS: ($APCI^+$) Calcd for $C_{24}H_{13}NO_2$ $[M+H]^+$: 348.1025; found: 348.1019

mp: 249 °C

Benzo[5,6]indolo[2,1-a]isoquinoline-8,13-dione (IQ6):

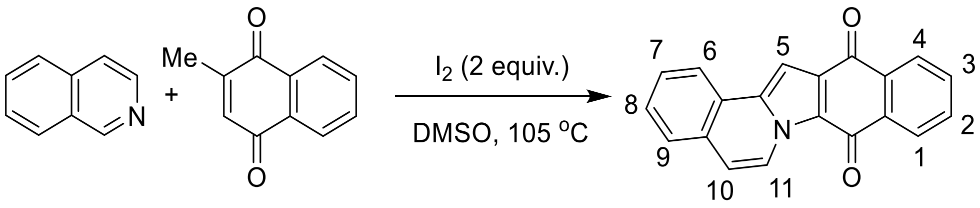

2a **1b** **IQ6**

Isoquinoline **2a** (1.13 g, 1.02 mL, 8.71 mmol) and I_2, (1.47 g, 5.81 mmol) were added to the solution of 2-methylnaphthalene-1,4-dione **1b** (500 mg, 2.90 mmol) in DMSO (5 mL). The mixture was heated at 105 °C under stirring for 24 h. Then, the reaction mixture was diluted with $CHCl_3$, (20-30 mL) and washed with 10 % HCl (15 mL), water (15 mL) and 5 % NaS_2O_3 solution (30 mL), dried over Na_2SO_4, and concentrated. The residue was purified using flash silica gel chromatography (hexanes:ethyl acetate 8:2) to **IQ6** (450 mg, 52%) as a brownish solid.

Rf: 0.5 (Hexane/EtOAc 8:2)

^{1}H NMR: (500 MHz, DMSO-d_6) δ 9.27 (d, J = 7.4 Hz, 1H; H–C (11)), 8.53 (d, J = 7.4 Hz, 1H; H–C (4)), 8.14 (dd, J = 7.5, 1.5 Hz, 1H; H–C (1)), 8.09 (dd, J = 7.3, 1.5 Hz, 1H; H–C (6)), 7.92-7.88 (m, 1H; H–C (10)), 7.87-7.78 (m, 3H; H–C (2, 3, 8)), 7.73-7.65 (m, 2H; H–C (7, 9)), 7.54 ppm (d, J = 7.5 Hz, 1H; H–C (5))

^{13}C NMR: (126 MHz, DMSO-d_6) δ 181.03, 173.81, 136.16, 134.42, 134.06, 133.28, 133.07, 129.22, 129.08, 128.88, 128.36, 127.51, 126.32, 126.13, 124.60, 124.42, 124.16, 123.08, 117.12, 101.43 ppm.

HRMS: (EI$^+$) Calcd for $C_{20}H_{11}NO_2$ [M]$^+$: 298.0868; found: 298.0864

Mp: 302 °C

Benzo[f]pyrido[1,2-a]indole-6,11-dione (IQ8a):

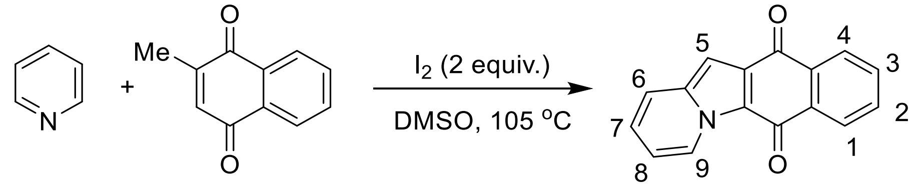

Pyridine **2b** (689.11 mg, 0.7 mL, 8.71 mmol) and I_2, (1.47 g, 5.81 mmol) were added to the solution of 2-methylnaphthalene-1,4-dione **1b** (500 mg, 2.90 mmol) in DMSO (5 mL). The mixture was heated at 105 °C under stirring for 48 h. Then, the reaction mixture was diluted with $CHCl_3$, (20-30 mL) and washed with 10 % HCl (15 mL), water (15 mL) and 5 % NaS_2O_3 solution (30 mL), dried over Na_2SO_4, and concentrated.

Rf: 0.5 (Hexane/EtOAc 8:2)

^{1}H NMR: (500 MHz, $CDCl_3$) δ 9.60 (d, J = 7.0 Hz, 1H; H–C (9)), 8.24-8.19 (m, 2H; H–C (2, 3)), 7.75-7.67 (m, 2H; H–C (1, 4)), 7.42 (d, J = 7.3 Hz, 1H; H–C (8)), 7.24 (s, 1H; H–C (5)), 6.90 ppm (t, J = 7.1 Hz, 1H; H–C (7))

^{13}C NMR: (126 MHz, $CDCl_3$) δ 181.88, 174.47, 137.90, 135.13, 133.95, 133.74, 133.07, 130.32, 127.49, 127.17, 127.14, 127.09, 126.65, 121.31, 116.41, 103.04 ppm.

HRMS: (EI$^+$) Calcd for $C_{16}H_9NO_2$ [M]$^+$: 247.0633; found: 247.0638

Mp: 240 °C

1-Bromobenzo[f]pyrido[1,2-a]indole-6,11-dione (IQ8b):

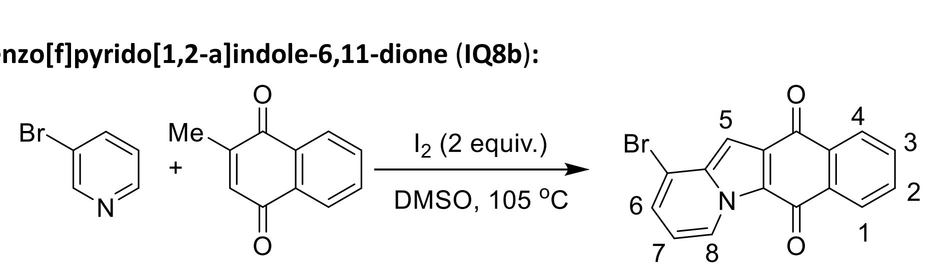

3-Bromopyridine **2d** (1.38 g, 0.84 mL, 8.71 mmol) and I_2, (1.47 g, 5.81 mmol) were added to the solution of 2-methylnaphthalene-1,4-dione **1b** (500 mg, 2.90 mmol) in DMSO (5 mL). The mixture was heated at 105 °C under stirring for 36 h. Then, the reaction mixture was diluted with $CHCl_3$, (20-30 mL) and washed with 10 % HCl (15 mL), water (15 mL) and 5 % NaS_2O_3 solution (30 mL), dried over Na_2SO_4, and concentrated. The residue was purified using flash silica gel chromatography (hexanes:ethyl acetate 8:2) to **IQ8b** (667 mg, 70%) as a maroon solid. An inseparable trace impurity was observed with **IQ8b**,

while the final product **I8b** was isolated as a pure compound. The yield of IQ8b has been calculated excluding the impurity's contribution.

Rf: 0.5 (Hexane/EtOAc 8:2)

^{1}H NMR: (500 MHz, $CDCl_3$) δ 9.60 (d, *J* = 7.0 Hz, 1H; H–C (8)), 8.24-8.19 (m, 2H; H–C (2, 3)), 7.75-7.67 (m, 2H; H–C (1, 4)), 7.42 (d, *J* = 7.3 Hz, 1H; H–C (6)), 7.24 (s, 1H; H–C (5)), 6.90 ppm (t, *J* = 7.1 Hz, 1H; H–C (7));

^{13}C NMR: (126 MHz, $CDCl_3$) δ 181.88, 174.47, 137.90, 135.13, 133.95, 133.74, 133.07, 130.32, 127.49, 127.17, 127.14, 127.09, 126.65, 121.31, 116.41, 103.04 ppm.

HRMS: (APCI$^{+)}$ Calcd for $C_{16}H_8BrNO_2$ $[M+H]^+$: 325.9817; found: 325.9822

Mp: 234 °C

1-Fluorobenzo[f]pyrido[1,2-a]indole-6,11-dione (IQ8c)

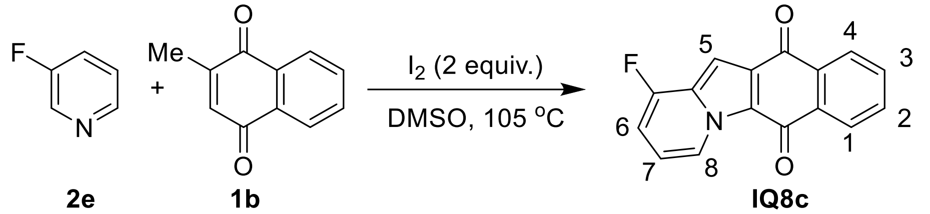

3-Fluoropyridine **2e** (507 mg, 0.45 mL, 5.23 mmol) and I_2, (884 mg, 3.48 mmol) were added to the solution of 2-methylnaphthalene-1,4-dione **1b** (300 mg, 1.74 mmol) in DMSO (3 mL). The mixture was heated at 105 °C under stirring for 48 h. Then, the reaction mixture was diluted with $CHCl_3$, (20-25 mL) and washed with 10 % HCl (10 mL), water (10 mL) and 5 % NaS_2O_3 solution (15 mL), dried over Na_2SO_4, and concentrated. The residue was purified using flash silica gel chromatography (hexanes:ethyl acetate 8:2) to **IQ8c** (140 mg, 30%) as a maroon solid. An inseparable trace impurity was observed along with IQ8c, while the aromatized product **I8c** was isolated as a pure compound. The yield of **IQ8c** has been calculated excluding the impurity's contribution.

Rf: 0.5 (Hexane/EtOAc 8:2)

^{1}H NMR: (500 MHz, $CDCl_3$) δ 9.68-9.64 (m, 1H; H–C (8)), 8.28-8.24 (m, 2H; H–C (2, 3)), 7.82-7.63 (m, 2H; H–C (1, 4)), 7.09-6.83 ppm (m, 2H; H–C (6, 7))

^{13}C NMR (126 MHz, $CDCl_3$) δ 181.22, 181.07, 174.35, 173.58, 156.62, 154.58, 134.47, 134.11, 134.03, 133.83, 133.81, 133.40, 133.29, 130.99, 129.24, 128.61, 127.29, 127.26, 126.50, 126.42, 125.14, 125.10, 124.83, 124.79, 124.61, 116.72, 116.66, 110.17, 109.64, 109.49, 109.27, 109.13 ppm.

HRMS: (APCI$^+$) Calcd for $C_{16}H_8FNO_2$ $[M+H]^+$: 266.0617; found: 266.0618

mp: 214 °C

2-(Trifluoromethyl)benzo[f]pyrido[1,2-a]indole-6,11-dione (IQ8d)

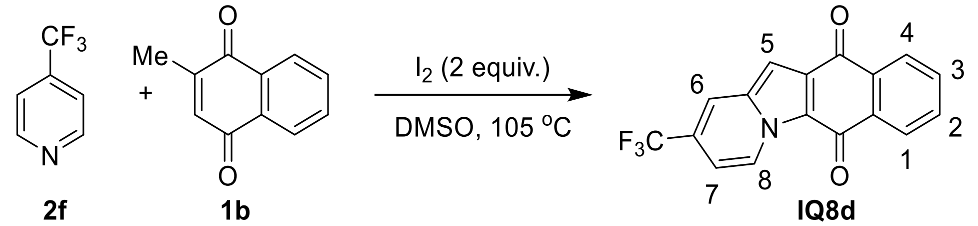

4-(Trifluoromethyl)pyridine **2f** (513 mg, 0.40 mL, 3.48 mmol) and I_2, (590 mg, 2.32 mmol) were added to the solution of 2-methylnaphthalene-1,4-dione **1b** (200 mg, 1.16 mmol) in DMSO (3 mL). The mixture was heated at 105 °C under stirring for 36 h. Then, the reaction mixture was diluted with $CHCl_3$, (25-30 mL) and washed with 10 % HCl (10 mL), water (15 mL) and 5 % NaS_2O_3 solution (15 mL), dried over Na_2SO_4, and concentrated. The residue was purified using flash silica gel chromatography (hexanes:ethyl acetate 8:2) to **IQ8d** (170 mg, 46%) as a maroon solid.

Rf: 0.55 (Hexane/EtOAc 8:2)

^{1}H NMR: (500 MHz, $CDCl_3$) δ 9.79 (d, *J* = 7.3 Hz, 1H; H–C (8)), 8.33-8.22 (m, 2H; H–C (2, 3)), 8.01 (s, 1H; H–C (6)), 7.82-7.67 (m, 2H; H–C (1, 4)), 7.22 ppm (dd, *J* = 7.4, 1.9 Hz, 1H; H–C (7))

^{13}C NMR: (126 MHz, $CDCl_3$) δ 180.78, 174.03, 138.00, 134.36, 134.22, 133.73, 133.67, 129.47, 129.26, 127.91, 127.63, 127.40, 126.66, 124.24, 119.29, 119.25, 113.14, 113.12 ppm.

HRMS: (APCI$^+$) Calcd for $C_{17}H_8F_3NO_2$ $[M+H]^+$: 316.0585; found: 316.0580

mp: 245 °C

Naphtho[2',3':5,6]indolo[2,1-a]isoquinoline-8,15-dione (IQ9)

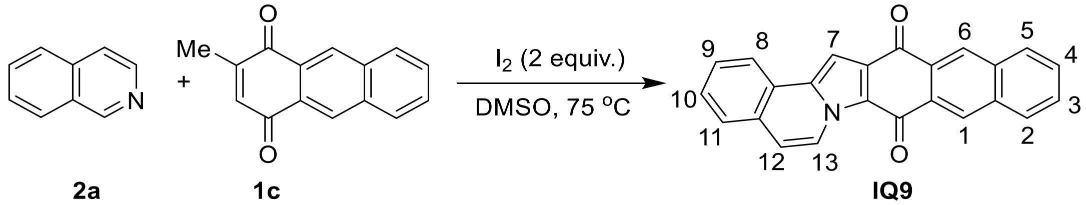

Isoquinoline **2a** (232 mg, 0.21 mL, 1.8 mmol) and I_2, (183 mg, 0.72 mmol) were added to the solution of 2-methylanthracene-1,4-dione **1c** (80 mg, 0.36 mmol) in DMSO (3 mL). The mixture was heated at 75 °C under stirring for 48 h. Then, the reaction mixture was diluted with $CHCl_3$, (5-10 mL) and washed with 10 % HCl (5 mL), water (5 mL) and 5 % NaS_2O_3 solution (10 mL), dried over Na_2SO_4, and concentrated. The residue was purified by multiple washing with methanol to obtain pure product **IQ9** (50 mg, 47%) as a brownish solid.

Rf: 0.6 (Hexane/EtOAc 8:2)

^{1}H NMR: (500 MHz, $CDCl_3$) δ 9.50 (d, *J* = 7.5 Hz, 1H; H–C (13)), 8.77 (d, *J* = 6.9 Hz, 2H; H–C (1, 6)), 8.25 (d, *J* = 7.9 Hz, 1H; H–C (8)) , 8.07 (d, *J* = 7.2 Hz, 2H; H–C (3, 4)), 7.76 (d, *J* = 7.7 Hz, 1H; H–C (2)), 7.70-7.59 (m, 5H; H–C (5, 9, 10, 11, 12)) , 7.28 ppm (s, 1H; H–C (7)). Due to the compound's poor solubility in conventional organic solvents, it was not possible to acquire satisfactory **^{13}C NMR** data.

HRMS: (APCI$^+$) Calcd for $C_{20}H_{11}NO_2$ $[M+H]^+$: 348.1025; found: 348.1028

mp: 294 °C

Naphtho[2,3-f]pyrido[1,2-a]indole-6,13-dione (IQ10)

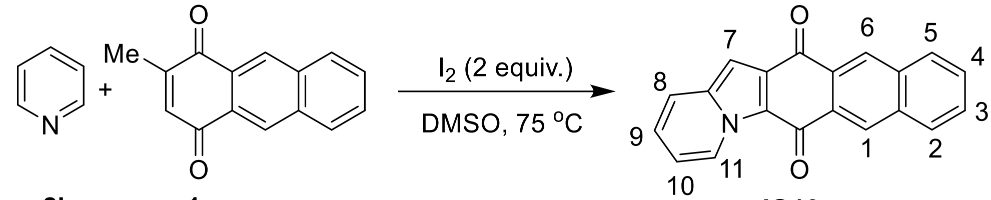

Pyridine **2b** (267 mg, 0.27 mL, 3.37 mmol) and I_2, (343 mg, 1.35 mmol) were added to the solution of 2-methylanthracene-1,4-dione **1c** (150 mg, 0.67 mmol) in DMSO (3 mL). The mixture was heated at 75 °C under stirring for 48 h. Then, the reaction mixture was diluted with $CHCl_3$, (20-25 mL) and washed with 10 % HCl (10 mL), water (10 mL) and 5 % NaS_2O_3 solution (10 mL), dried over Na_2SO_4, and concentrated. The residue was purified by multiple washing with methanol to obtain pure product **IQ10** (165 mg, 82%) as a brownish solid.

Rf: 0.6 (Hexane/EtOAc 8:2)

^{1}H NMR: (500 MHz, $CDCl_3$) δ 9.75 (d, *J* = 7.3 Hz, 1H; H–C (11)), 8.74 (d, *J* = 8.8 Hz, 2H; H–C (1, 6)), 8.05 (d, *J* = 7.4 Hz, 2H; H–C (2, 5)), 7.73-7.58 (m, 3H; H–C (3, 4, 10)), 7.25-7.20 (m, 1H; H–C (8)), 7.13 (s, 1H; H–C (7)), 7.10-7.00 ppm (m, 1H; H–C (9))

^{13}C NMR: (126 MHz, $CDCl_3$) δ 182.18, 173.70, 139.04, 135.33, 134.59, 132.10, 131.61, 130.77, 130.28, 130.01, 129.35, 129.32, 129.04, 128.60, 128.46, 125.46, 120.88, 116.57, 101.73 ppm.

HRMS: (APCI$^+$) Calcd for $C_{20}H_{11}NO_2$ $[M+H]^+$: 298.0868; found: 298.0870

mp: 285 °C

9,10-Dimethyl-8,11-bis((triisopropylsilyl)ethynyl)indolo[2,1-a]isoquinoline (I1)

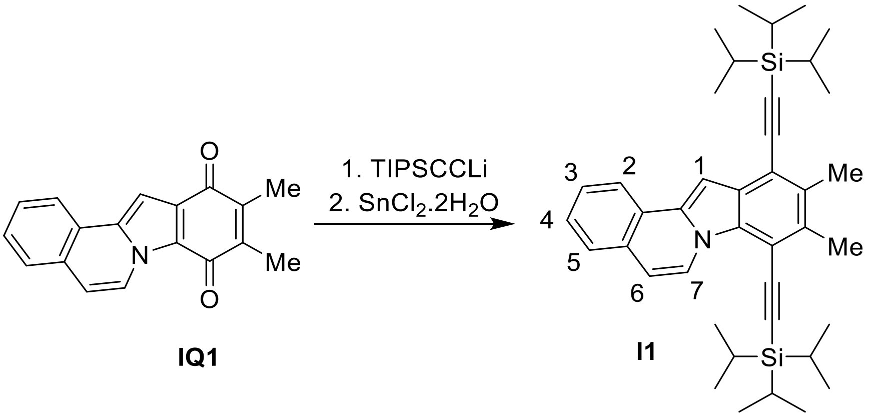

n-BuLi (0.59 mL, 1.47 mmol, 2.5 M in hexanes) was added dropwise to the solution of TIPS-acetylene (0.18 mL, 1.47 mmol) in dry THF (5 mL) at –78 °C. After 30 min. 8,9-dimethylbenzo[f]pyrido[1,2-a]indole-6,11-dione **IQ1** (45 mg , 0.16 mmol) was added in one portion. The reaction mixture was allowed to warm to 25 °C and stirred for 16 h. Afterwards, a solution of $SnCl_2.2H_2O$ (221 mg, 0.98 mmol) in 5 mL 10% aqueous HCl was added to the flask and stirring continued for 1 hr. Extraction with hexanes (15 mL), washing with water (3 x 10 mL), drying with $MgSO_4$, and flash column chromatography (SiO_2, hexanes:CH_2Cl_2 20:1) gave **I1** (38 mg, 38%) as a yellow solid.

Rf: 0.05 (Hexane/EtOAc 9.5:0.5)

^{1}H NMR: (600 MHz, $CDCl_3$) δ 9.71 (d, *J* = 7.6 Hz, 1H; H–C (7)), 8.14 (dd, *J* = 7.9, 1.2 Hz, 1H; H–C (2)), 7.55 (s, 1H; H–C (1)), 7.48 (dd, *J* = 7.7, 1.4 Hz, 1H; H–C (3)), 7.45 (dd, *J* = 7.5, 1.4 Hz, 1H; H–C (4)), 6.59 (d, *J* = 7.6 Hz, 1H; H–C (6)), 2.64 (s, 3H; Me), 2.63 (s, 3H; Me), 1.32-1.17 ppm (m, 42H; TIPS)

^{13}C NMR: (151 MHz, $CDCl_3$) δ 135.17, 134.60, 133.77, 129.68, 128.99, 128.10, 127.73, 127.21, 126.65, 125.94, 124.50, 123.82, 115.07, 107.81, 107.32, 105.19, 104.44, 103.52, 99.12, 93.26, 19.00, 18.91, 11.69, 11.63 ppm.

HRMS: ($EI^{+)}$ Calcd for $C_{30}H_{29}NSi_2$ $[M+H]^+$: 460.1917; found: 460.1915

mp: 174 °C

UV/Vis (CH_2Cl_2): λ_{max} (ε) = 435 (18090), 412 (24685), 391 (20380), 332 (48335), 303 (61880), 288 nm (84410 M^{-1} cm^{-1})

2,3-Dimethyl-1,4-bis((triisopropylsilyl)ethynyl)pyrido[1,2-a]indole (I4)

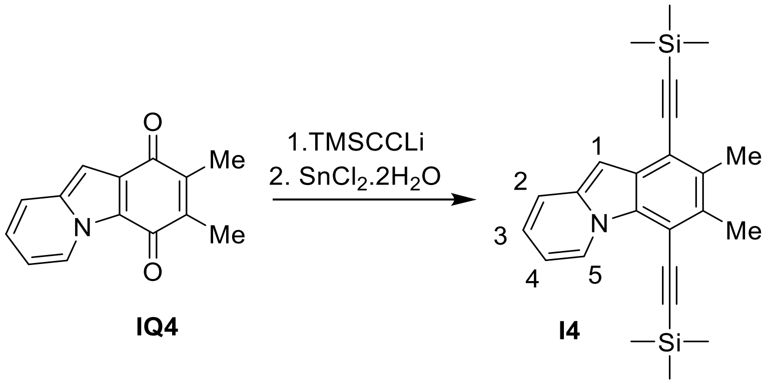

IQ4 **I4**

n-BuLi (0.72 mL, 1.80 mmol, 2.5 M in hexanes) was added dropwise to the solution of TMS-acetylene (0.26 mL, 1.80 mmol) in dry THF (5 mL) at –78 °C. After 30 min. 2,3-dimethylpyrido[1,2-a]indole-1,4-dione **IQ4** (45 mg , 0.20 mmol) was added in one portion. The reaction mixture was allowed to warm to 25 °C and stirred for 16 h. Afterwards, a solution of $SnCl_2.2H_2O$ (270 mg, 1.2 mmol) in 5 mL 10% aqueous HCl was added to the flask and stirring continued for 1 hr. Extraction with hexanes (10 mL), washing with water (3 x 10 mL), drying with $MgSO_4$, and flash column chromatography (SiO_2, hexanes:CH_2Cl_2 20:1) gave **I4** (26 mg, 34%) as a light-yellow solid.

Rf: 0.90 (Hexane/EtOAc 9.5:0.5)

^{1}H NMR: (500 MHz, Acetone-d_6) δ 9.80 (d, *J* = 7.2 Hz, 1H; H–C (5)), 7.56 (d, *J* = 9.3 Hz, 1H; H–C (2)), 7.02-6.95 (m, 1H; H–C (3)), 6.73 (s, 1H; H–C (1)), 6.61-6.55 (m, 1H; H–C (4)), 2.60 (s, 3H; Me), 2.59 (s, 3H; Me), 0.39 (s, 9H; TMS), 0.33 ppm (s, 9H; TMS)

^{13}C NMR: (126 MHz, Acetone-d_6) δ 138.17, 135.25, 133.85, 130.63, 126.61, 123.39, 120.17, 115.32, 108.69, 108.53, 107.88, 104.13, 103.38, 103.23, 92.64, 18.78, 18.22, 0.37, -0.10 ppm.

HRMS: ($APCI^+$) Calcd for $C_{24}H_{29}NSi_2$ $[M+H]^+$: 388.1917; found: 388.1922

mp: 146 °C

UV/Vis (CH_2Cl_2): λ_{max} (ε) = 481 (4745), 453 (7815), 428 (6960), 355 (8585), 340 (7895), 300 (22735), 283 nm (36215 M^{-1} cm^{-1})

10,15-Bis((trimethylsilyl)ethynyl)benzo[5,6]indolo[1,2-f]phenanthridine (I5)

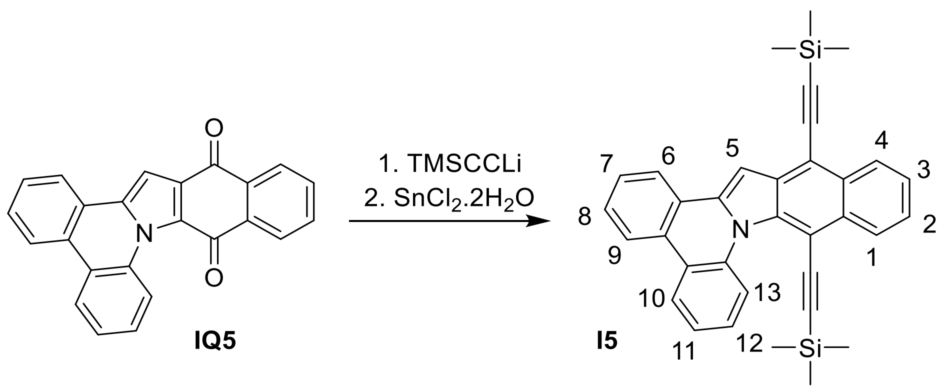

n-BuLi (0.40 mL, 1.01 mmol, 2.5 M in hexanes) was added dropwise to the solution of TMS-acetylene (0.23 mL, 1.01 mmol) in dry THF (5 mL) at –78 °C. After 30 min. benzo[5,6]indolo[1,2-f]phenanthridine-10,15-dione **IQ5** (50 mg , 0.14 mmol) was added in one portion. The reaction mixture was allowed to warm to 25 °C and stirred for 16 h. Afterwards, a solution of $SnCl_2.2H_2O$ (194 mg, 0.86 mmol) in 5 mL 10% aqueous HCl was added to the flask and stirring continued for 1 hr. Extraction with hexanes (15 mL), washing with water (3 x 10 mL), drying with $MgSO_4$, and flash column chromatography (SiO_2, hexanes:CH_2Cl_2 20:1) gave **I5** (30 mg, 41%) as a greenish yellow solid.

Rf: 0.95 (Hexane/EtOAc 9.5:0.5)

^{1}H NMR: (500 MHz, $CDCl_3$) δ 8.69-8.64 (m, 1H; H–C (13)), 8.54-8.49 (m, 1H; H–C (10, 12)), 8.28-8.24 (m, 1H; H–C (6)), 8.24-8.16 (m, 2H; H–C (1, 4)), 7.64-7.47 (m, 5H; H–C (2, 3, 8, 9, 11)), 7.47-7.43 (m, 1H; H–C (7)), 7.37-7.31 (m, 1H; H–C (5)), 0.44 (s, 9H; TMS), 0.18 ppm (s, 9H; TMS)

^{13}C NMR: (126 MHz, $CDCl_3$) δ 141.25, 135.39, 134.65, 134.48, 131.56, 130.54, 129.44, 129.35, 128.33, 127.03, 126.62, 126.49, 126.34, 125.64, 125.57, 125.46, 123.72, 123.43, 122.82, 122.73, 122.55, 114.45, 111.12, 105.53, 105.34, 102.10, 101.17, 96.67, 0.49, -0.07 ppm.

HRMS: (EI$^+$) Calcd for $C_{34}H_{31}NSi_2$ $[M+H]^+$ 509.1995; found: 509.1997

mp: 115 °C

UV/Vis (CH_2Cl_2): λ_{max} (ε) = 434 (12540), 410 (11725), 344 (7265), 325 (5775), 290 (21235), 261 nm (40990 M^{-1} cm^{-1})

8,13-Bis((triisopropylsilyl)ethynyl)benzo[5,6]indolo[2,1-a]isoquinoline (**I6**):

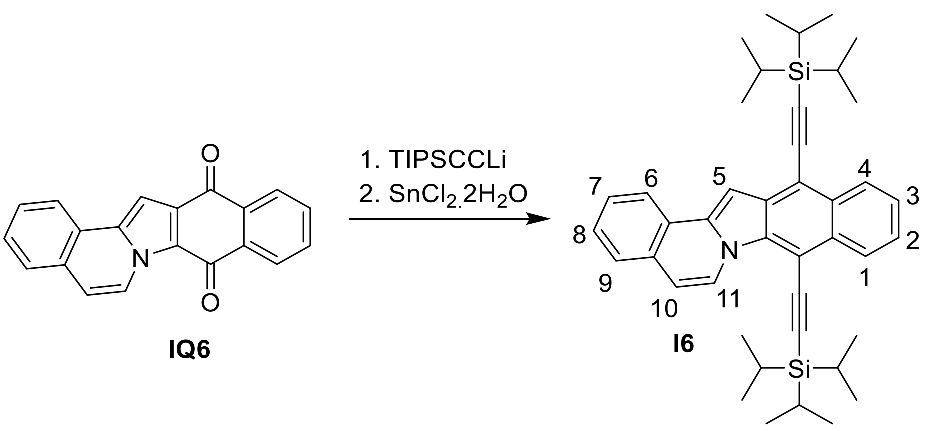

n-BuLi (2.83 mL, 7.06 mmol, 2.5 M in hexanes) was added dropwise to the solution of TIPS-acetylene (1.87 mL, 7.06 mmol) in dry THF (15 mL) at –78 °C. After 30 min. benzo[5,6]indolo[2,1-a]isoquinoline-8,13-dione **IQ6** (300 mg , 1.01 mmol) was added in one portion. The reaction mixture was allowed to warm to 25 °C and stirred for 16 h. Afterwards, a solution of $SnCl_2.2H_2O$ (1.37 g, 6.05 mmol) in 5 mL 10% aqueous HCl was added to the flask and stirring continued for 1 hr. Extraction with hexanes (25 mL), washing with water (3 x 15 mL), drying with $MgSO_4$, and flash column chromatography (SiO_2, hexanes:CH_2Cl_2 20:1) gave **I6** (350 mg, 55%) as an orange solid.

Rf: 0.95 (Hexane/EtOAc 9.5:0.5)

^{1}H NMR: δ 9.85 (d, J = 7.6 Hz, 1H; H–C (11)), 8.70-8.63 (m, 1H; H–C (4)), 8.63-8.57 (m, 1H; H–C (1)), 8.23-8.16 (m, 1H; H–C (6)), 7.63-7.55 (m, 3H; H–C (2, 3, 8)), 7.55-7.48 (m, 2H; H–C (7, 9)), 7.44 (s, 1H; H–C (5)), 6.61 (d, J = 7.6 Hz, 1H; H–C (10)), 1.44-1.12 ppm (m, 42H; TIPS)

^{13}C NMR: (126 MHz, $CDCl_3$) 139.27, 132.87, 131.24, 130.83, 130.38, 130.13, 129.04, 127.44, 126.77, 126.43, 126.21, 125.45, 125.21, 125.09, 125.04, 124.59, 111.25, 107.55, 106.20, 104.23, 103.16, 102.76, 101.92, 93.14, 19.09, 19.00, 11.78, 11.70 ppm.

HRMS: (APCI$^+$) Calcd for $C_{42}H_{53}NSi_2$ $[M+H]^+$: 628.3795; found: 628.3791

mp: 229 °C

UV/Vis (CH_2Cl_2): λ_{max} (ε) = 517 (17670), 485 (17890), 460 (10090), 381 (8780), 342 (43030), 301 nm (52255 M^{-1} cm^{-1})

8,13-Bis((triisopropylsilyl)ethynyl)benzo[5,6]indolo[2,1-a]isoquinoline-14-carbaldehyde (Vilsmeier Haack) (I6a):

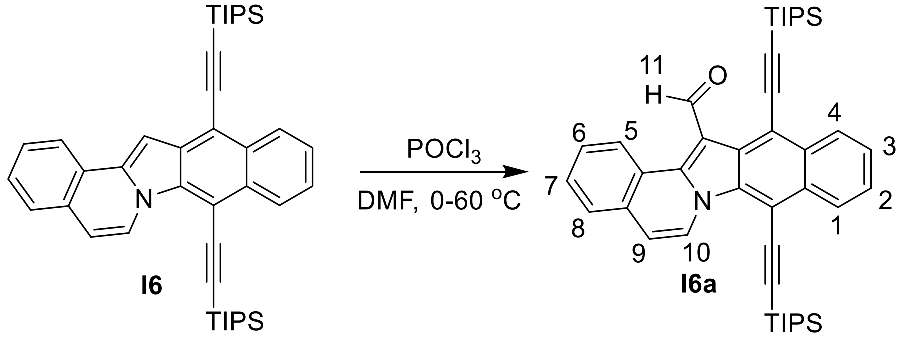

To a solution of 8,13-bis((triisopropylsilyl)ethynyl)benzo[5,6]indolo[2,1-a]isoquinoline **I6** (100 mg, 0.16 mmol) in DMF (36 µL, 0.46 mmol) at 0 °C under N_2, $POCl_3$ (44 µL, 0.46 mmol) was added dropwise. The mixture was stirred for 2 hours at 25 °C. After completion of the reaction, 1.5 mL of ice water and 1.1 mL of 25% NaOH were added. The mixture was refluxed at 60 °C for 10 minutes. Afterward, it was cooled to room temperature and crude product was extracted with chloroform (15 mL) washing with water (3 x 15 mL), drying with $MgSO_4$, then concentrated in vacuo. The concentrated mixture was purified by silica gel chromatography, yielding **I6a** (45 mg; 43%) as a red solid.

Rf: 0.90 (Hexane/EtOAc 9.5:0.5)

^{1}H NMR: (600 MHz, $CDCl_3$) δ 11.99 (s, 1H; H–C (11)), 10.42 (d, J = 7.4 Hz, 2H; H–C (1, 4)), 8.78 (dd, J = 8.0, 1.7 Hz, 1H; H–C (2)), 8.71 (dd, J = 8.0, 1.7 Hz, 1H; H–C (3)), 7.80-7.61 (m, 4H; H–C (5, 6, 7, 8)), 7.07 (d, J = 7.5 Hz, 1H; H–C (9)), 1.29 ppm (m, 42H; TIPS)

^{13}C NMR: (151 MHz, $CDCl_3$) δ 185.87, 142.30, 132.57, 132.43, 131.79, 131.48, 131.37, 131.16, 130.71, 127.70, 126.72, 126.60, 126.51, 126.37, 126.13, 125.09, 124.06, 112.60, 110.61, 110.41, 108.95, 106.77, 105.17, 104.44, 102.35, 19.00, 18.98, 11.72, 11.70 ppm.

HRMS: ($APCI^+$) Calcd for $C_{43}H_{53}NOSi_2$ $[M+H]^+$: 356.3744; found: 656.3745

mp: 222 °C

14-Bromo-8,13-bis((triisopropylsilyl)ethynyl)benzo[5,6]indolo[2,1-a]isoquinoline (I6b):

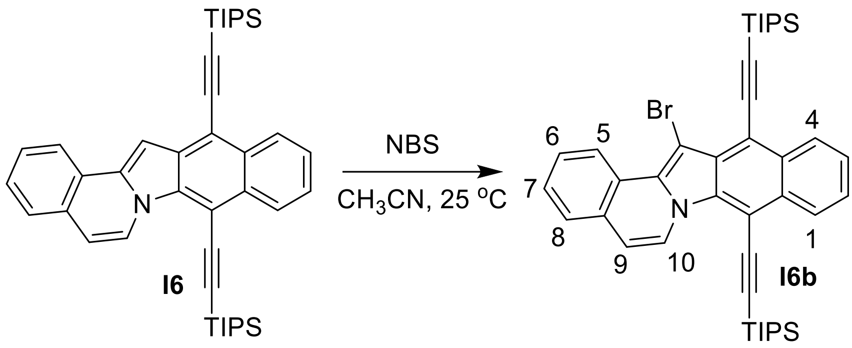

To a solution of 8,13-bis((triisopropylsilyl)ethynyl)benzo[5,6]indolo[2,1-a]isoquinoline **I6** (50 mg, 0.08 mmol) in 3 mL of CH_3CN, N-bromosuccinimide (NBS) (17 mg, 0.095 mmol) was added, and the mixture was stirred at room temperature for 2 hours. The reaction was then quenched using water, followed by extraction with hexanes (10 mL), washing with water (3 x 15 mL), drying with $MgSO_4$, and flash column chromatography (SiO_2, hexanes:CH_2Cl_2, 20:1), which yielded **I6b** (48 mg; 85%) as a dark orange solid.

Rf: 0.90 (Hexane/EtOAc 9.5:0.5)

^{1}H NMR: (600 MHz, $CDCl_3$) δ 10.07 (d, *J* = 7.6 Hz, 1H; H–C (10)), 9.62-9.49 (m, 1H; H–C (4)), 8.84-8.76 (m, 1H; H–C (1)), 8.73-8.64 (m, 1H; H–C (5)), 7.65-7.58 (m, 2H; H–C (2, 3)), 7.58-7.45 (m, 3H; H–C (6, 8)), 6.61 (d, *J* = 7.6 Hz, 1H; H–C (9)), 1.59-1.10 ppm (m, 42H; TIPS)

^{13}C NMR: (151 MHz, $CDCl_3$) δ 133.53, 131.82, 131.22, 131.13, 130.37, 129.23, 127.89, 126.85, 126.65, 126.64, 126.14, 125.81, 125.76, 125.60, 125.48, 124.47, 111.79, 108.21, 108.00, 107.51, 103.08, 102.89, 102.87, 85.02, 19.08, 18.99, 11.85, 11.74 ppm.

HRMS: ($APCI^+$) Calcd for $C_{42}H_{52}BrNSi_2$ $[M]^+$: 705.2822; found: 705.2830

mp: 193 °C

(Z)-14-((4-Bromophenyl)diazenyl)-8,13-bis((triisopropylsilyl)ethynyl)benzo[5,6]indolo[2,1-a]isoquinoline (I6c):

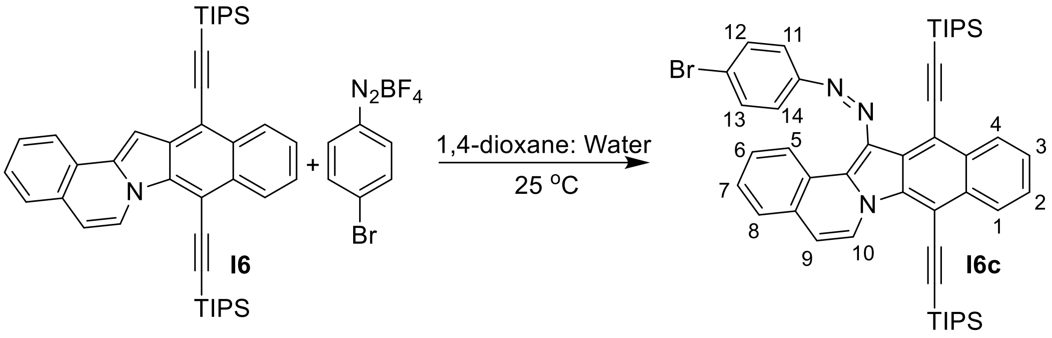

To a solution of 8,13-bis((triisopropylsilyl)ethynyl)benzo[5,6]indolo[2,1-a]isoquinoline **I6** (24 mg, 0.038 mmol) in 3 mL of 1,4-dioxane:Water (9:1), 1-(4-bromophenyl)-2-(tetrafluoro-λ^5-boraneyl)diazene (31 mg, 0.114 mmol) was added, and the mixture was stirred at room temperature for 2 hours. The reaction was then quenched using water, followed by extraction with hexanes (10 mL), washing with water (3 x 5 mL), drying with $MgSO_4$, and flash column chromatography (SiO_2, hexanes:CH_2Cl_2, 20:1), which yielded **I6c** (25 mg, 80%) as a purple solid.

Rf: 0.90 (Hexane/EtOAc 9.5:0.5)

^{1}H NMR: (600 MHz, $CDCl_3$) δ 10.25 (d, *J* = 7.5 Hz, 1H; H–C (10)), 9.46-9.37 (m, 1H; H–C (4)), 8.86-8.76 (m, 1H; H–C (1)), 8.71-8.60 (m, 1H; H–C (5)), 7.86 (d, *J* = 8.4 Hz, 2H; H–C (2, 3)), 7.73-7.69 (m, 1H; H–C (6, 8)), 7.68-7.60 (m, 5H; H–C (7, 11, 12, 13, 14)) , 6.97 (d, *J* = 7.6 Hz, 1H; H–C (7)), 1.45-1.13 (m, 21H; TIPS), 1.11-0.60 ppm (m, 21H; TIPS)

^{13}C NMR: (126 MHz, $CDCl_3$) δ 133.51, 132.69, 132.14, 131.96, 131.60, 130.84, 130.08, 129.08, 127.50, 127.05, 126.71, 126.34, 125.20, 124.42, 123.62, 122.57, 121.01, 114.31, 113.03, 111.82, 108.58, 104.30, 104.00, 102.40, 19.00, 18.92, 11.73, 11.59 ppm.

HRMS: (APCI+) Calcd for $C_{48}H_{56}BrN_3Si_2$ $[M]^+$: 809.3196; found: 809.3190

mp: 213 °C

6,11-Bis((triisopropylsilyl)ethynyl)benzo[f]pyrido[1,2-a]indole (**I8a**):

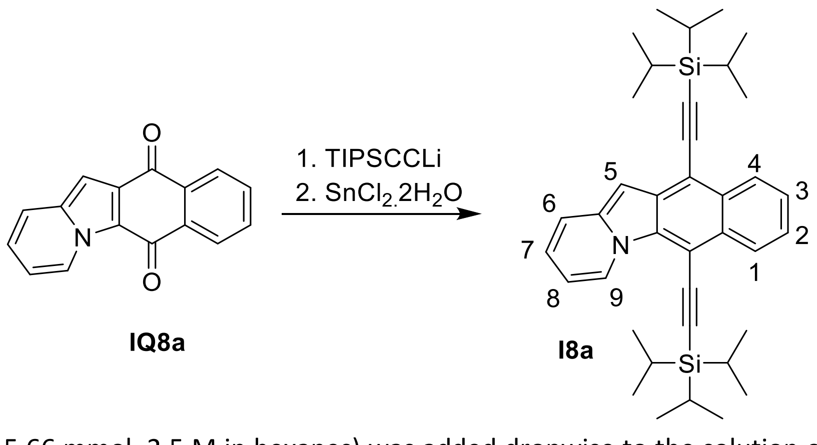

n-BuLi (2.26 mL, 5.66 mmol, 2.5 M in hexanes) was added dropwise to the solution of TIPS-acetylene (1.27 mL, 5.66 mmol) in dry THF (15 mL) at –78 °C. After 30 min. benzo[f]pyrido[1,2-a]indole-6,11-dione **IQ8a** (200 mg , 0.81 mmol) was added in one portion. The reaction mixture was allowed to warm

to 25 °C and stirred for 16 h. Afterwards, a solution of $SnCl_2.2H_2O$ (1.09 g, 4.85 mmol) in 5 mL 10% aqueous HCl was added to the flask and stirring continued for 1 hr. Extraction with hexanes (25 mL), washing with water (3 x 15 mL), drying with $MgSO_4$, and flash column chromatography (SiO_2, hexanes:CH_2Cl_2 20:1) gave **I8a** (140 mg, 30%) as a purple solid.

Rf: 0.95 (Hexane/EtOAc 9.5:0.5)

^{1}H NMR: (500 MHz, $CDCl_3$): δ 10.08 (d, J = 7.3 Hz, 1H; H–C (9)), 8.65 (d, J = 8.2 Hz, 1H; H–C (1)), 8.58 (d, J = 7.7 Hz, 1H; H–C (4)), 7.60-7.52 (m, 2H; H–C (2, 3)), 7.45 (d, J = 9.3 Hz, 1H; H–C (6)), 6.98 (dd, J = 9.3, 6.3 Hz, 1H; H–C (7)), 6.87 (s, 1H; H–C (5)), 6.41-6.35 (m, 1H; H–C (8)), 1.33-1.25 ppm (m, 42H; TIPS)

^{13}C NMR: (126 MHz, $CDCl_3$) 140.6, 132.9, 130.8, 130.0, 129.7, 128.0, 126.3, 126.3, 125.4, 125.1, 124.7, 119.2, 110.6, 106.7, 106.6, 104.5, 103.7, 103.3, 101.9, 91.6, 19.1, 19.0, 11.8, 11.7 ppm.

HRMS: (APCI$^+$) Calcd for $C_{38}H_{51}NSi_2$ $[M+H]^+$: 578.3638; found: 578.3643

mp: 160 °C

UV/Vis (CH_2Cl_2): λ_{max} (ε) = 580 (8420), 540 (12620), 506 (10070), 385 (11570), 367 (9655), 339 (16500), 300 (63010), 290 nm (59370 M^{-1} cm^{-1})

4-Bromo-6,11-bis((triisopropylsilyl)ethynyl)benzo[f]pyrido[1,2-a]indole (I8b):

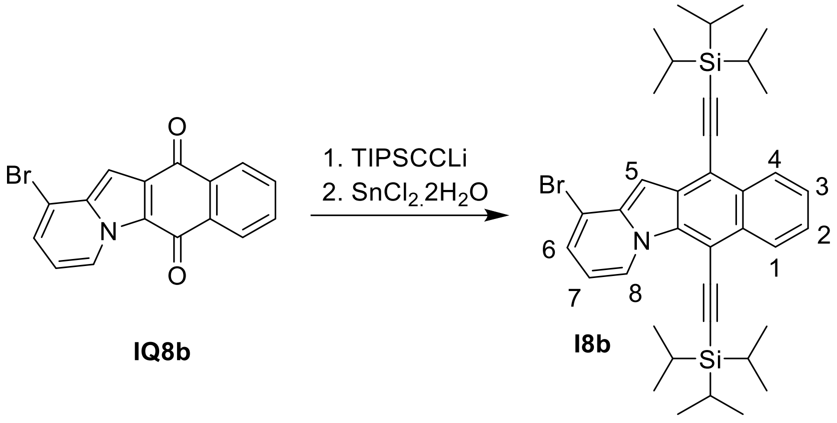

n-BuLi (1.37 mL, 3.43 mmol, 2.5 M in hexanes) was added dropwise to the solution of TIPS-acetylene (0.77 mL, 3.43 mmol) in dry THF (10 mL) at –78 °C. After 30 min. 1-bromobenzo[f]pyrido[1,2-a]indole-6,11-dione **IQ8b** (160 mg , 0.49 mmol) was added in one portion. The reaction mixture was allowed to warm to 25 °C and stirred for 16 h. Afterwards, a solution of $SnCl_2.2H_2O$ (664 mg, 2.94 mmol) in 5 mL 10% aqueous HCl was added to the flask and stirring continued for 1 hr. Extraction with hexanes (25 mL), washing with water (3 x 15 mL), drying with $MgSO_4$, and flash column chromatography (SiO_2, hexanes:CH_2Cl_2 20:1) gave **I8b** (269 mg, 83%) as a purple solid.

Rf: 0.95 (Hexane/EtOAc 9.5:0.5)

^{1}H NMR: (600 MHz, $CDCl_3$): δ 10.10 (d, J = 7.2 Hz, 1H; H–C (8)), 8.65 (d, J = 7.5 Hz, 1H; H–C (1)), 8.59 (d, J = 7.7 Hz, 1H; H–C (4)), 7.65-7.52 (m, 2H; H–C (2, 3)), 7.27 (d, J = 6.3 Hz, 1H; H–C (6)), 6.32-6.26 (m, 1H; H–C (7)), 1.46-1.20 ppm (m, 42H; TIPS)

^{13}C NMR: (151 MHz, $CDCl_3$) 139.2, 132.4, 130.9, 130.6, 130.4, 127.3, 127.0, 126.5, 126.3, 125.7, 125.2, 112.8, 111.8, 107.2, 106.4, 104.1, 104.0, 102.9, 102.8, 94.3, 19.1, 19.0, 11.8, 11.7 ppm.

HRMS: (APCI$^+$) Calcd for $C_{38}H_{50}BrNSi_2$ $[M+H]^+$: 656.2743; found: 656.2740

mp: 155 °C

UV/Vis (CH_2Cl_2): λ_{max} (ε) = 574 (9010), 534 (14120), 500 (11155), 385 (11980), 341 (15145), 300 (69825), 290 nm (67365 M^{-1} cm^{-1})

1-Fluoro-6,11-bis((triisopropylsilyl)ethynyl)benzo[f]pyrido[1,2-a]indole (I8c)

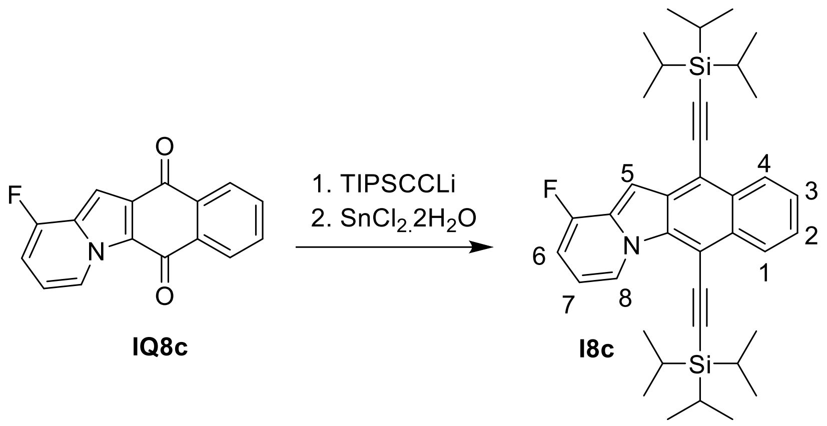

n-BuLi (1.58 mL, 3.96 mmol, 2.5 M in hexanes) was added dropwise to the solution of TIPS-acetylene (0.88 mL, 3.96 mmol) in dry THF (10 mL) at –78 °C. After 30 min. 1-fluorobenzo[f]pyrido[1,2-a]indole-6,11-dione **IQ8c** (150 mg , 0.56 mmol) was added in one portion. The reaction mixture was allowed to warm to 25 °C and stirred for 16 h. Afterwards, a solution of $SnCl_2.2H_2O$ (756 mg, 3.39 mmol) in 5 mL 10% aqueous HCl was added to the flask and stirring continued for 1 hr. Extraction with hexanes (25 mL), washing with water (3 x 15 mL), drying with $MgSO_4$, and flash column chromatography (SiO_2, hexanes:CH_2Cl_2 20:1) gave **I8c** (202 mg, 60%) as a purple solid.

Rf: 0.95 (Hexane/EtOAc 9.5:0.5)

^{1}H NMR: (500 MHz, $CDCl_3$) δ 9.91 (d, *J* = 7.2 Hz, 1H; H–C (8)), 8.65 (d, *J* = 7.7 Hz, 1H; H–C (1)), 8.59 (d, *J* = 7.6 Hz, 1H; H–C (4)), 7.65-7.53 (m, 2H; H–C (2, 3)), 7.05 (s, 1H; H–C (5)), 6.73-6.64 (m, 1H; H–C (6)), 6.37-6.28 (m, 1H; H–C (7)), 1.53-0.82 ppm (m, 42H; TIPS)

^{13}C NMR: (126 MHz, $CDCl_3$) δ 154.79, 152.81, 133.30, 133.01, 132.36, 130.95, 130.55, 130.29, 126.47, 126.31, 125.63, 125.23, 124.17, 111.88, 107.03, 105.96, 105.83, 105.24, 105.19, 103.97, 102.91, 102.66, 89.38, 19.04, 18.97, 11.73, 11.64 ppm.

HRMS: (EI$^+$) Calcd for $C_{38}H_{50}FNSi_2$ $[M+H]^+$: 595.3466; found: 595.3447

mp: 117 °C

UV/Vis (CH_2Cl_2): λ_{max} (ε) = 504 (8710), 467 (12515), 436 (9575), 333 (9625), 288 (13790), 248 (54135), 238 nm (52955 M^{-1} cm^{-1})

2-(Trifluoromethyl)-6,11-bis((triisopropylsilyl)ethynyl)benzo[f]pyrido[1,2-a]indole (I8d)

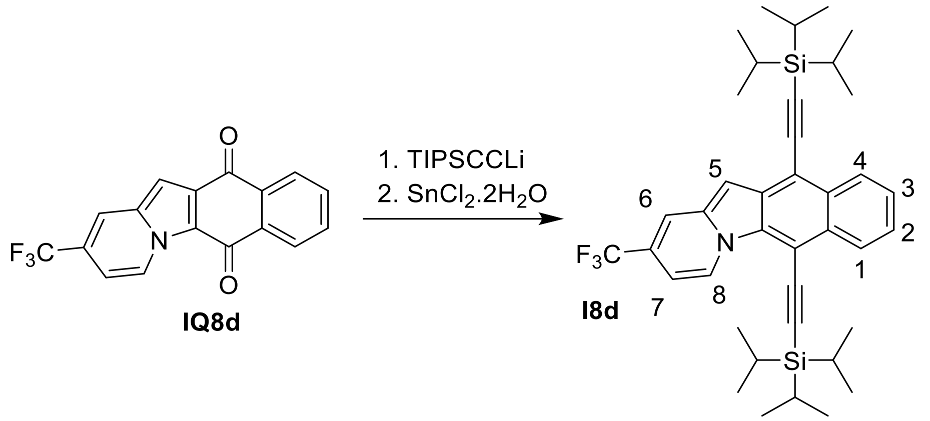

n-BuLi (1.42 mL, 3.55 mmol, 2.5 M in hexanes) was added dropwise to the solution of TIPS-acetylene (0.79 mL, 3.55 mmol) in dry THF (10 mL) at –78 °C. After 30 min. 2-(trifluoromethyl)benzo[f]pyrido[1,2-a]indole-6,11-dione **IQ8d** (150 mg , 0.47 mmol) was added in one portion. The reaction mixture was allowed to warm to 25 °C and stirred for 16 h. Afterwards, a solution of $SnCl_2.2H_2O$ (644 mg, 2.85 mmol) in 5 mL 10% aqueous HCl was added to the flask and stirring continued for 1 hr. Extraction with hexanes (25 mL), washing with water (3 x 15 mL), drying with $MgSO_4$, and flash column chromatography (SiO_2, hexanes:CH_2Cl_2 20:1) gave **I8d** (188 mg, 61%) as a purple solid.

Rf: 0.95 (Hexane/EtOAc 9.5:0.5)

^{1}H NMR: (500 MHz, $CDCl_3$) δ 10.17 (d, *J* = 7.6 Hz, 1H; H–C (8)), 8.72-8.64 (m, 1H; H–C (1)), 8.64-8.57 (m, 1H; H–C (2)), 7.83-7.77 (m, 1H; H–C (6)), 7.64-7.57 (m, 2H; H–C (2, 3)), 7.12 (s, 1H; H–C (5)), 6.51-6.40 (m, 1H; H–C (7)), 1.35-1.27 ppm (m, 42H; TIPS)

^{13}C NMR: (126 MHz, $CDCl_3$) δ 137.54, 132.73, 130.96, 129.54, 128.80, 128.77, 126.56, 126.31, 125.83, 125.54, 124.67, 122.51, 117.75, 117.71, 112.06, 107.59, 104.05, 103.89, 103.01, 102.82, 101.87, 101.84, 101.82, 101.80, 95.81, 19.05, 18.98, 11.74, 11.66 ppm.

HRMS: (EI^+) Calcd for $C_{39}H_{50}F_3NSi_2$ $[M+H]^+$ 645.3434; found: 645.3451

mp: 96 °C

UV/Vis (CH_2Cl_2): λ_{max} (ε) = 570 (7625), 432 (11950), 501 (9630), 389 (10695), 341 (12675), 302 (52425), 291 nm (63220 M^{-1} cm^{-1})

6,11-Bis((triisopropylsilyl)ethynyl)-1-((trimethylsilyl)ethynyl)benzo[f]pyrido[1,2-a]indole (I8e):

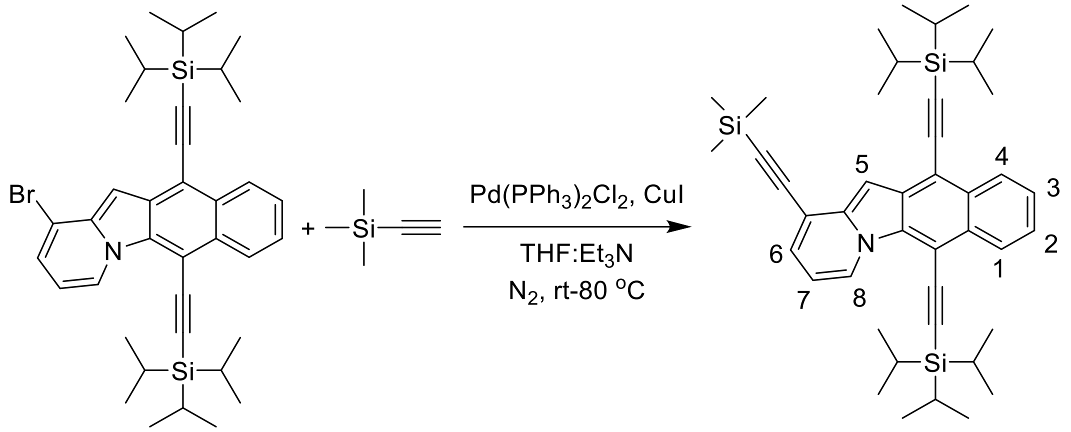

I8b **I8e**

1-Bromo-6,11-bis((triisopropylsilyl)ethynyl)benzo[f]pyrido[1,2-a]indole **I8b** (200 mg, 0.3 mmol), $Pd(PPh_3)_2Cl_2$ (10.7 mg, 0.015 mmol), and CuI (3.84 mg, 0.018 mmol) were dissolved in THF (7 mL) and triethylamine (5 mL) under inert atmosphere. Trimethylsilylacetylene (0.065 mL, 0.46 mmol) was slowly added to the mixture, and the mixture was refluxed for 18 hours. The reaction was quenched using saturated NH_4Cl, followed by extraction with hexanes (25 mL), washing with water (3 x 15 mL), drying with $MgSO_4$, and flash column chromatography (SiO_2, hexanes:CH_2Cl_2 20:1) gave **I8e** (162 mg, 79%) as a purple solid.

Rf: 0.90 (Hexane/EtOAc 9.5:0.5)

^{1}H NMR: (600 MHz, $CDCl_3$) δ 10.09 (d, *J* = 7.2 Hz, 1H; H–C (8)), 8.65 (dd, *J* = 8.4, 0.8 Hz, 1H; H–C (1)), 8.60 (dd, *J* = 8.2, 0.7 Hz, 1H; H–C (4)), 7.61-7.55 (m, 2H; H–C (2, 3)), 7.21 (dd, *J* = 6.7, 0.9 Hz, 1H; H–C (6)), 7.10 (d, *J* = 0.9 Hz, 1H; H–C (5)), 6.36 (dd, *J* = 7.3, 6.6 Hz, 1H; H–C (7)) , 1.40-1.09 (m, 42H; TIPS), 0.34 ppm (s, 9H; TMS)

^{13}C NMR: (151 MHz, $CDCl_3$) δ 140.15, 132.66, 130.93, 130.25, 130.09, 129.79, 128.22, 126.40, 126.28, 125.56, 124.95, 113.72, 111.20, 107.03, 106.24, 104.26, 103.90, 103.01, 102.11, 100.96, 100.26, 92.65, 19.08, 18.97, 11.73, 11.66, 0.12 ppm.

HRMS: (APCI+) Calcd for $C_{43}H_{59}NSi_3$ $[M+H]^+$: 674.4034; found: 674.4037

mp: 166 °C

8,15-Bis((triisopropylsilyl)ethynyl)naphtho[2',3':5,6]indolo[2,1-a]isoquinoline (I9)

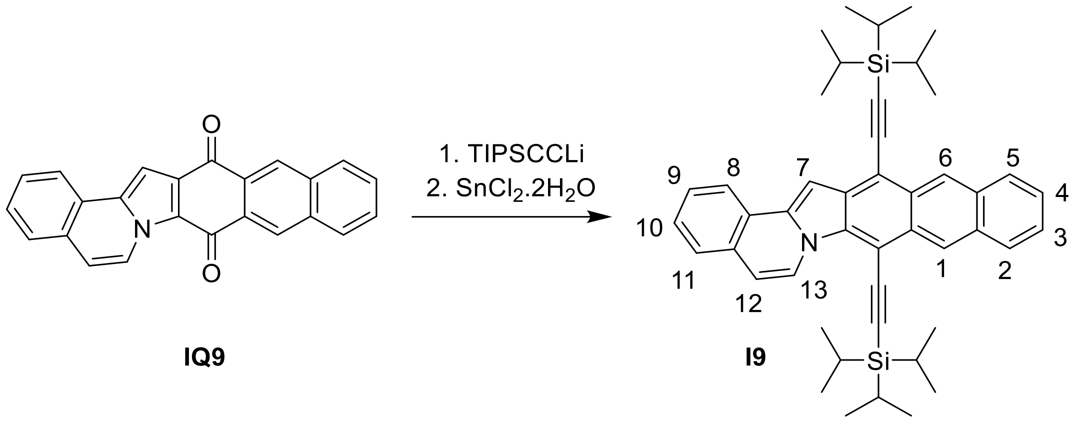

IQ9 **I9**

n-BuLi (1.04 mL, 2.59 mmol, 2.5 M in hexanes) was added dropwise to the solution of TIPS-acetylene (0.58 mL, 2.59 mmol) in dry THF (10 mL) at –78 °C. After 30 min. naphtho[2',3':5,6]indolo[2,1-a]isoquinoline-8,15-dione **IQ9** (100 mg , 0.29 mmol) was added in one portion. The reaction mixture was allowed to warm to 25 °C and stirred for 16 h. Afterwards, a solution of $SnCl_2.2H_2O$ (390 mg, 1.73 mmol) in 5 mL 10% aqueous HCl was added to the flask and stirring continued for 1 hr. Extraction with hexanes (25 mL), washing with water (3 x 15 mL), drying with $MgSO_4$, and flash column chromatography (SiO_2, hexanes:CH_2Cl_2 20:1) gave **I9** (120 mg, 61%) as a purple solid.

Rf: 0.95 (Hexane/EtOAc 9.5:0.5)

^{1}H NMR: (600 MHz, Acetone-d_6) δ 9.88 (d, *J* = 7.6 Hz, 1H; H–C (13)), 9.39 (s, 1H; H–C (1)), 9.28 (s, 1H; H–C (6)), 8.35-8.32 (m, 1H; H–C (8)), 8.14-8.08 (m, 2H; H–C (2, 10)), 7.76 (dd, *J* = 6.3, 2.7 Hz, 1H; H–C (5)), 7.67-7.61 (m, 2H; H–C (3, 4)), 7.58-7.55 (m, 2H; H–C (9, 11)), 7.55 (d, *J* = 0.8 Hz, 1H; H–C (2)), 6.88 (d, *J* = 7.6 Hz, 1H; H–C (12)), 1.40-1.0 ppm (m, 42H; TIPS)

^{13}C NMR: (126 MHz, Acetone-d_6+CS_2+DMSO-d_6) δ 142.57, 135.84, 134.35, 133.08, 133.01, 132.51, 131.79, 131.08, 130.73, 130.31, 130.26, 129.76, 129.14, 127.78, 127.60, 127.07, 127.04, 126.89, 126.81, 126.72, 112.38, 109.75, 109.15, 106.82, 105.51, 104.69, 103.65, 95.33, 21.08, 21.01, 14.04, 13.95 ppm.

HRMS: (EI^+) Calcd for $C_{46}H_{55}NSi_2$ $[M+H]^+$ 677.3873; found: 677.3862

mp: 310 °C

UV/Vis (CH_2Cl_2): λ_{max} (ε) = 594 (24925), 553 (19305), 415 (10985), 361 (37570), 318 (112110), 292 nm (69725 M^{-1} cm^{-1})

6,13-Bis((trimethylsilyl)ethynyl)naphtho[2,3-f]pyrido[1,2-a]indole (I10)

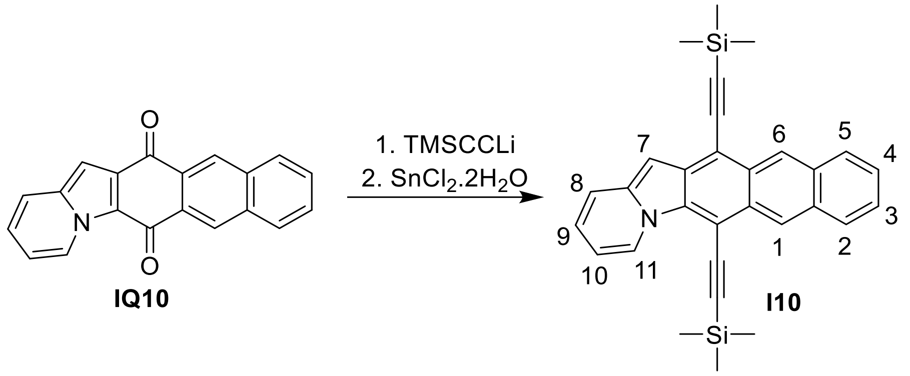

n-BuLi (0.47 mL, 1.18 mmol, 2.5 M in hexanes) was added dropwise to the solution of TMS-acetylene (0.17 mL, 1.18 mmol) in dry THF (5 mL) at –78 °C. After 30 min. naphtho[2,3-f]pyrido[1,2-a]indole-6,13-dione **IQ10** (50 mg , 0.17 mmol) was added in one portion. The reaction mixture was allowed to warm to 25 °C and stirred for 16 h. Afterwards, a solution of $SnCl_2.2H_2O$ (228 mg, 1.01 mmol) in 5 mL 10% aqueous HCl was added to the flask and stirring continued for 10 minutes. Extraction with hexanes (10 mL), washing with water (3 x 10 mL), drying with $MgSO_4$, and flash column chromatography (basic alumina, hexanes:CH_2Cl_2 20:1) gave **I10** (30 mg, 38%) as a greenish blue solid.

Rf: 0.90 (Hexane/EtOAc 9.5:0.5)

^{1}H NMR: (600 MHz, Acetone-*d*$_6$) δ 9.95 (dd, *J* = 7.3, 1.0 Hz, 1H; H–C (11)), 9.26 (d, *J* = 1.0 Hz, 1H; H–C (1)), 9.14 (d, *J* = 1.0 Hz, 1H; H–C (6)), 8.16-8.13 (m, 2H; H–C (2, 5)), 7.58 (d, *J* = 9.3 Hz, 1H; H–C (8)), 7.56-7.49 (m, 2H; H–C (3, 4)), 7.18-7.14 (m, 1H; H–C (10)), 6.87 (d, *J* = 0.9 Hz, 1H; H–C (7)), 6.61-6.58 (m, 1H; H–C (9)), 0.53 (s, 9H; TMS), 0.46 (s, 9H; TMS) ppm. The **^{13}C NMR** could not be recorded due to the instability of the compound.

HRMS: ($APCI^+$) Calcd for $C_{30}H_{29}NSi_2$ $[M+H]^+$ 460.1917; found: 460.1915

mp: 162 °C

UV/Vis (CH_2Cl_2): λ_{max} (ε) = 620 (5535), 569 (6815), 527 (5300), 376 (4900), 264 (47285) 257 nm (49440 M^{-1} cm^{-1})

S3. Photophysical Measurements and Stability Studies

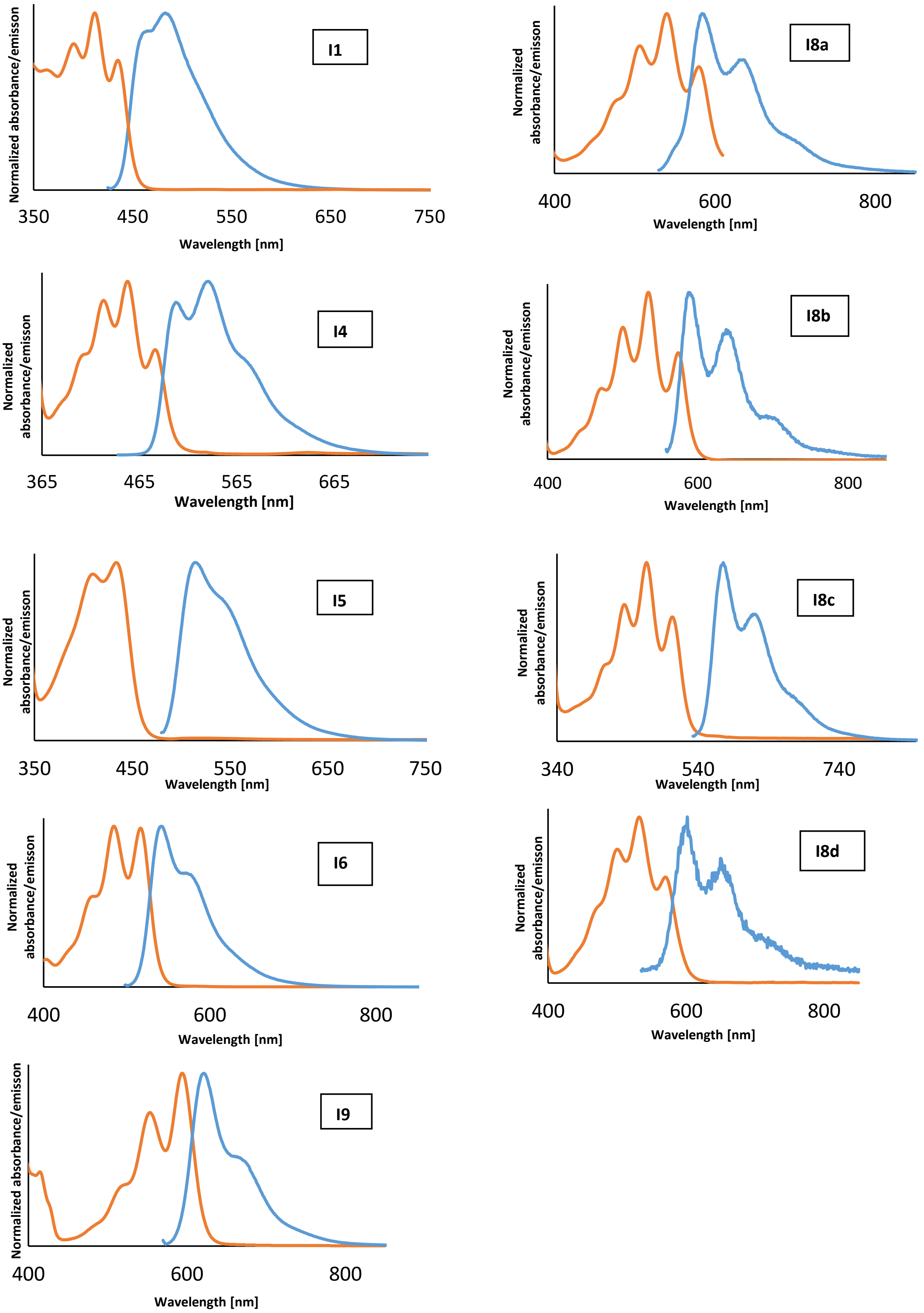

Figure S 1. The normalized absorption and emission spectra of compounds **I1; I4; I5; I6; I8a; I8b; I8c; I8d,** and **I9.**

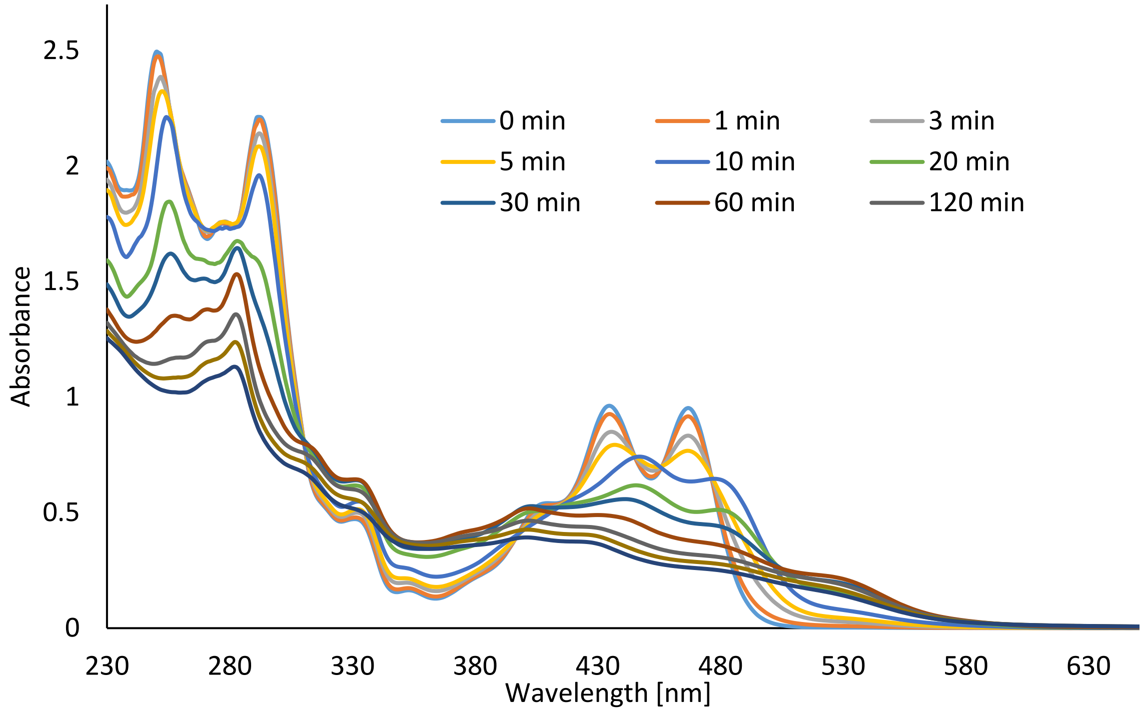

Figure S 2. Spectral changes upon irradiation of **I6** with 254 nm light (4W lamp) in aerated CH_2Cl_2.

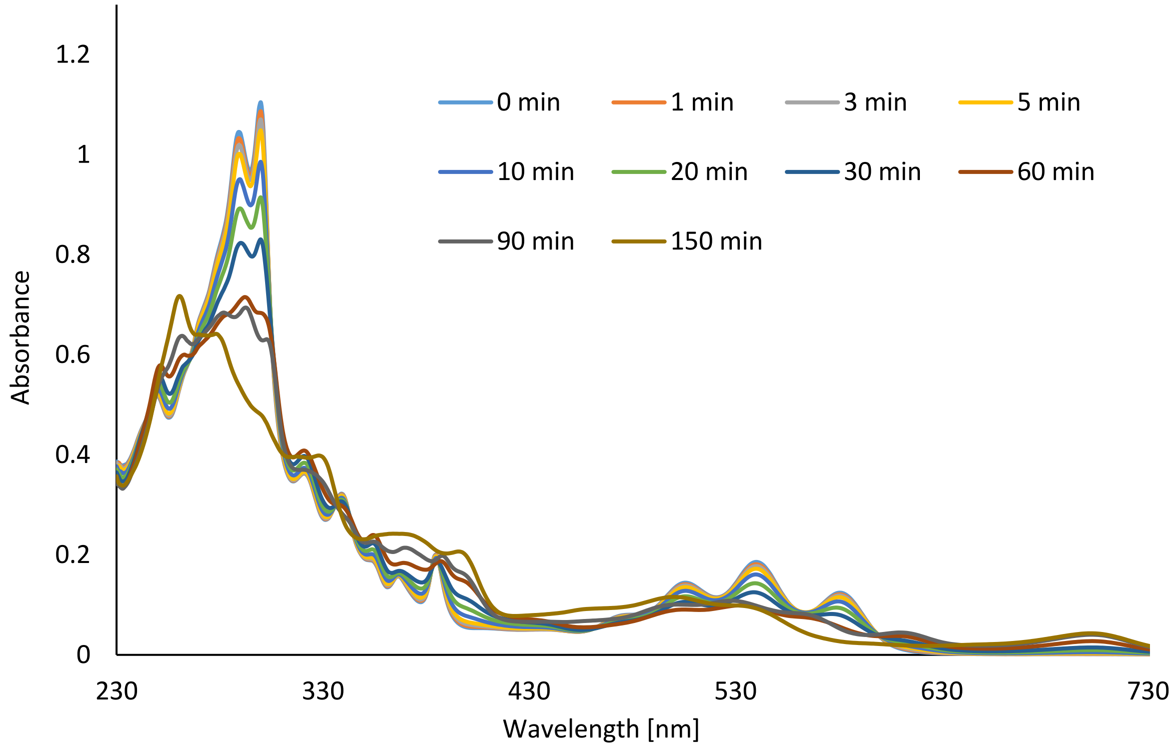

Figure S 3. Spectral changes upon irradiation of **I8a** with 254 nm light (4W lamp) in aerated CH_2Cl_2.

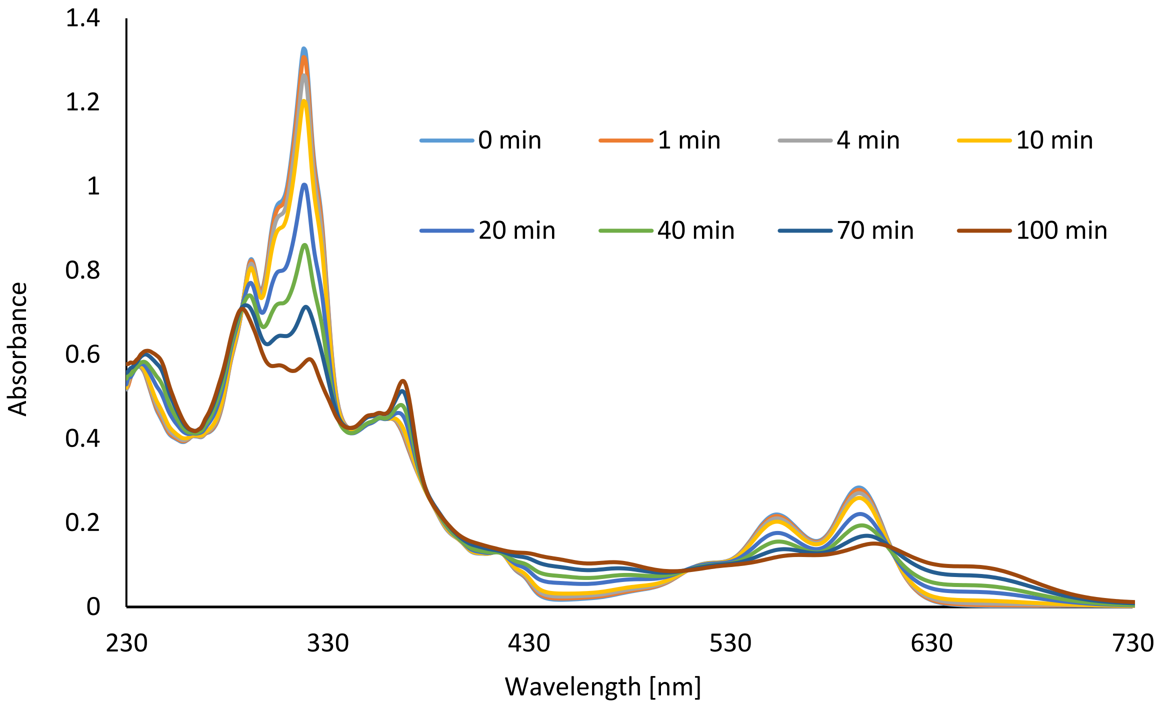

Figure S 4. Spectral changes upon irradiation of **I9** with 254 nm light (4W lamp) in aerated CH_2Cl_2.

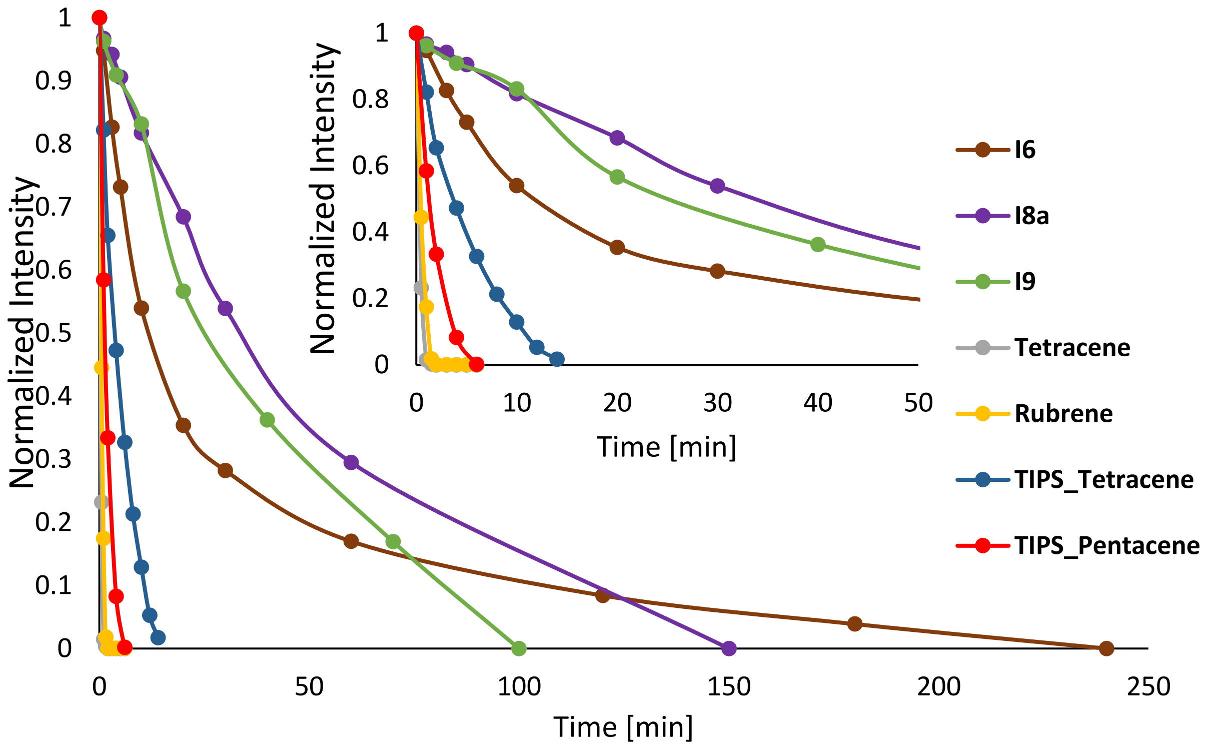

Figure S 5. Relative intensity of λ_{max} upon irradiation with 254 nm light (4W lamp) in aerated CH_2Cl_2 (corrected for absorption of forming new species). TIPS_Tetracene (CAS: 628316-50-7); TIPS_Pentacene (CAS: 373596-08-8).

S4. Electrochemistry

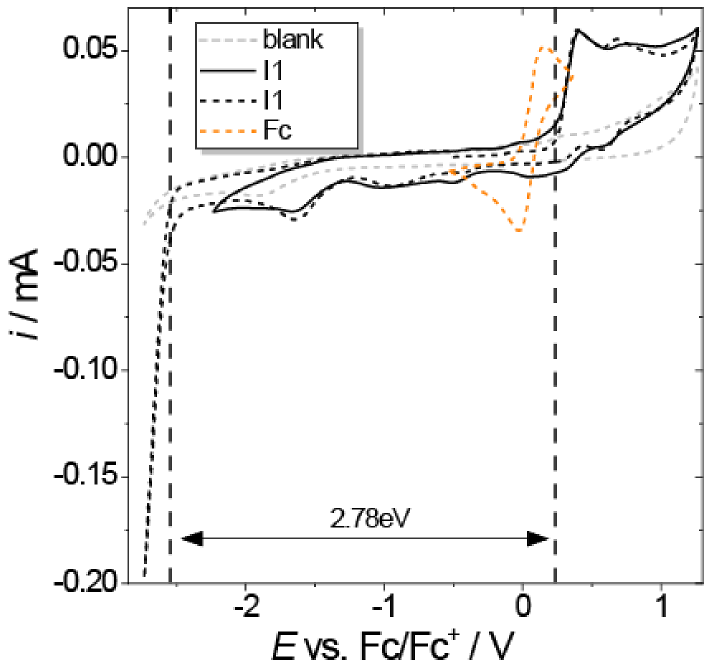

Figure S 6. Cyclic voltammograms recorded for **I1** (black solid line) and ferrocenium (orange dotted line) in 50 mM TBAP in DCM on Pt electrode; T = 268 K; sweep rate v = 100 mV s^{-1}.

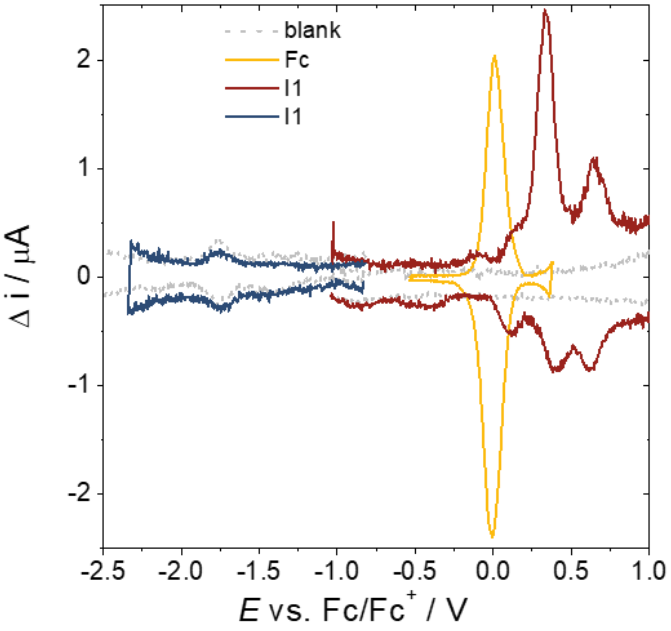

Figure S 7. Differential pulse voltammograms recorded for **I1** (anodic scan – red line, cathodic scan – blue line) and ferrocenium (orange line) in 50 mM TBAP in DCM on Pt electrode; T = 268 K; P_H = ±2.5 mV, P_W = 100 ms, S_t = 500 ms, S_H = ±5 mV. From this DPV curve it is not possible to determine the HOMO-LUMO gap as redox pair at low potentials is not visible.

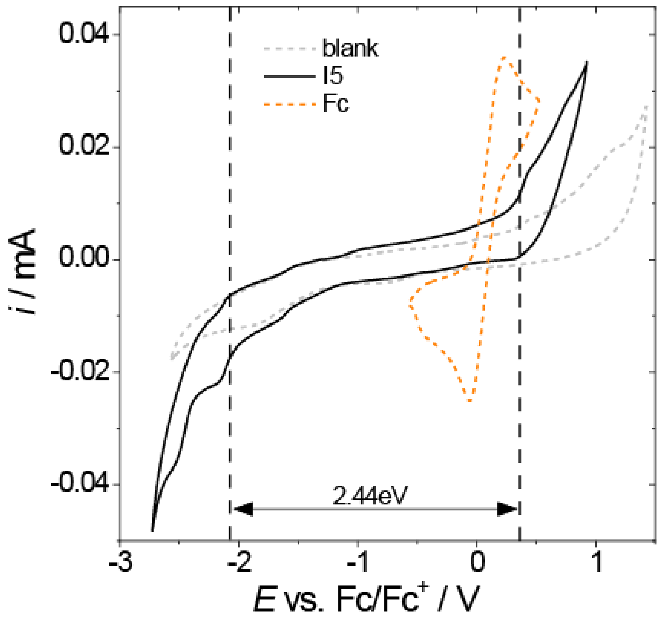

Figure S 8. Cyclic voltammograms recorded for **I5** (black solid line) and ferrocenium (orange dotted line) in 50mM TBAP in DCM on Pt electrode; T = 268K; sweep rate v = 100mV s^{-1}.

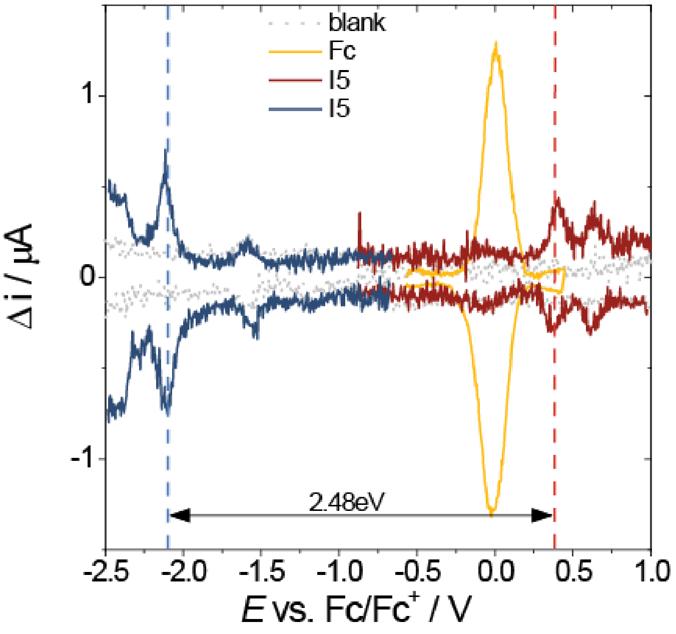

Figure S 9. Differential pulse voltammograms recorded for **I5** (anodic scan – red line, cathodic scan – blue line) and ferrocenium (orange line) in 50 mM TBAP in DCM on Pt electrode; T = 268K; P_H = ±2.5 mV, P_W = 100 ms, S_t = 500 ms, S_H = ±5 mV.

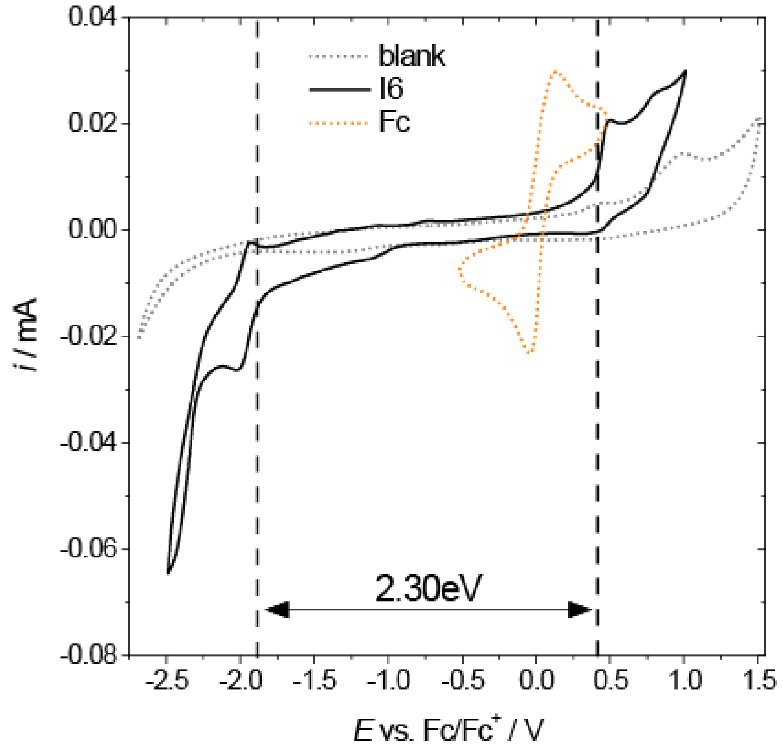

Figure S 10. Cyclic voltammograms recorded for **I6** (black solid line) and ferrocenium (orange dotted line) in 50mM TBAP in DCM on Pt electrode; T = 268K; sweep rate v = 100mV s^{-1}.

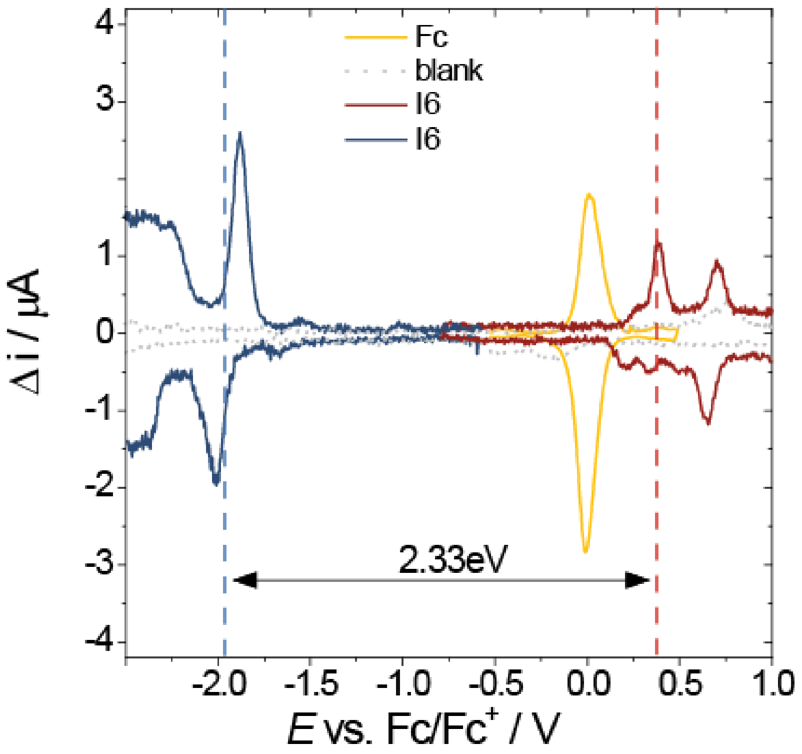

Figure S 11. Differential pulse voltammograms recorded for **I6** (anodic scan – red line, cathodic scan – blue line) and ferrocenium (orange line) in 50 mM TBAP in DCM on Pt electrode; T = 268 K; P_H = ±2.5 mV, P_W = 100 ms, S_t = 500 ms, S_H = ±5 mV.

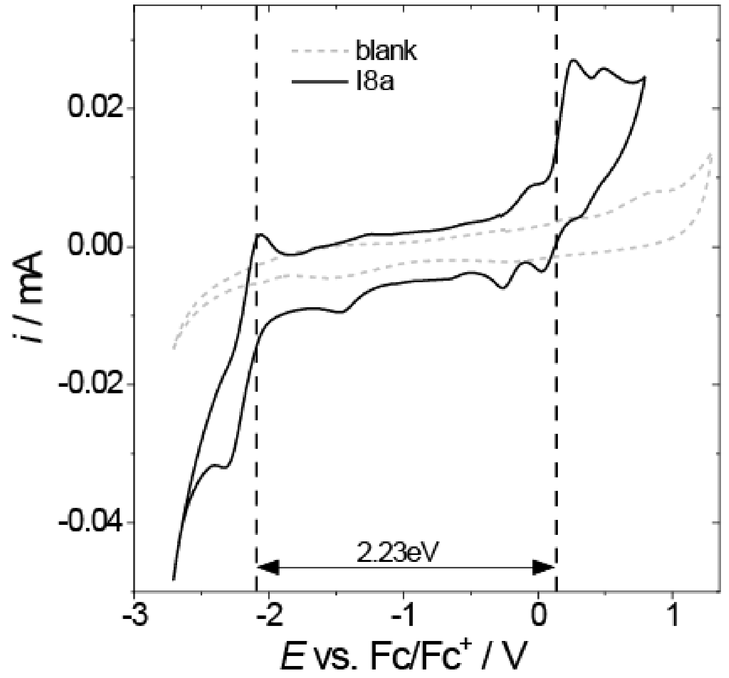

Figure S 12. Cyclic voltammograms recorded for **I8a** (black solid line) in 50mM TBAP in DCM on Pt electrode; T = 268K; sweep rate v = 100mV s^{-1}.

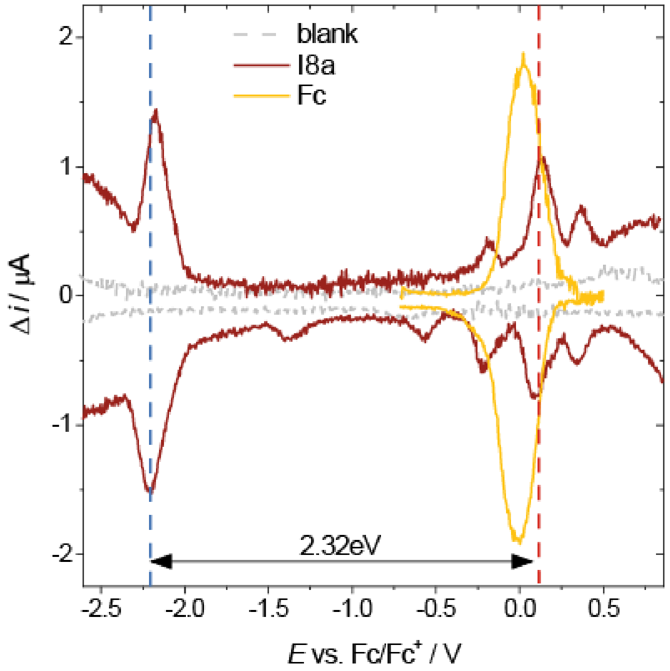

Figure S 13. Differential pulse voltammograms recorded for **I8a** (red solid line) and ferrocenium (orange line) in 50 mM TBAP in DCM on Pt electrode; T = 268 K; P_H = ±2.5 mV, P_W = 100 ms, S_t = 500 ms, S_H = ±5 mV.

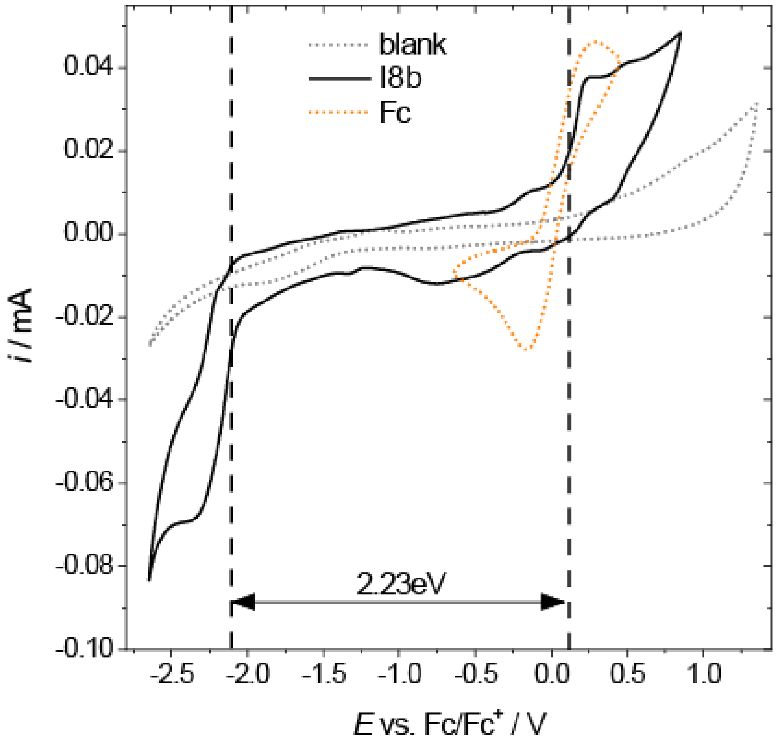

Figure S 14. Cyclic voltammograms recorded for **I8b** (black solid line) and ferrocenium (orange dotted line) in 50mM TBAP in DCM on Pt electrode; T = 268K; sweep rate v = 100mV s^{-1}.

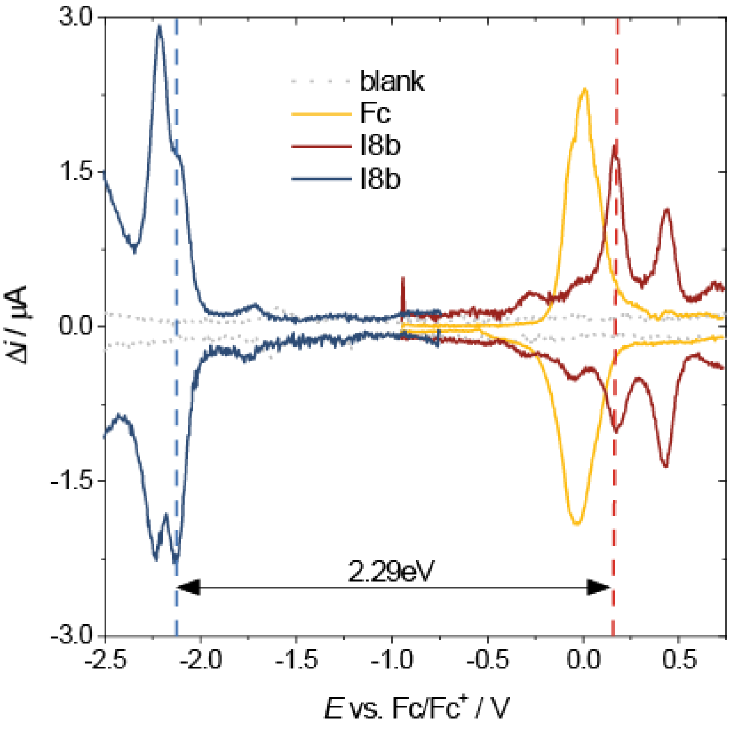

Figure S 15. Differential pulse voltammograms recorded for **I8b** (anodic scan – red line, cathodic scan – blue line) and ferrocenium (orange line) in 50 mM TBAP in DCM on Pt electrode; T = 268 K;P_H = ±2.5 mV, P_W = 100 ms, S_t = 500 ms, S_H = ±5 mV.

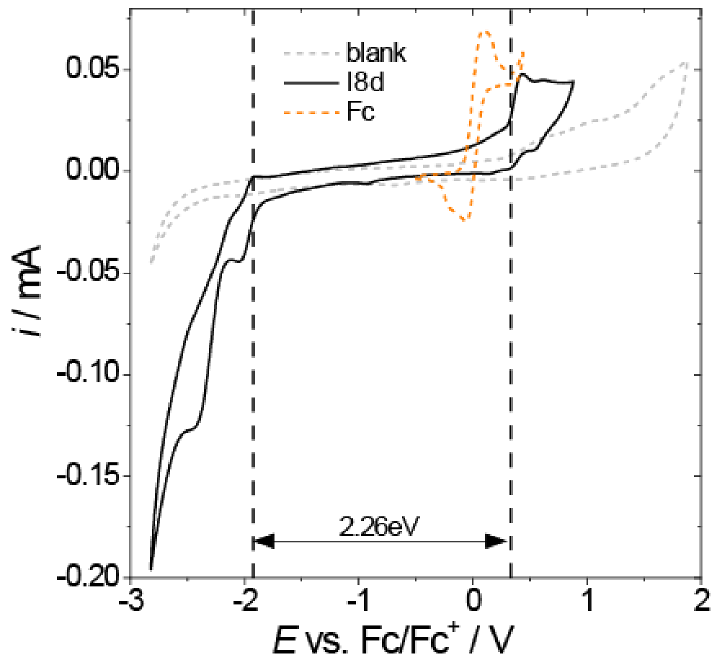

Figure S 16. Cyclic voltammograms recorded for **I8d** (black solid line) and ferrocenium (orange dotted line) in 50mM TBAP in DCM on Pt electrode; T = 268K; sweep rate v = 100mV s^{-1}.

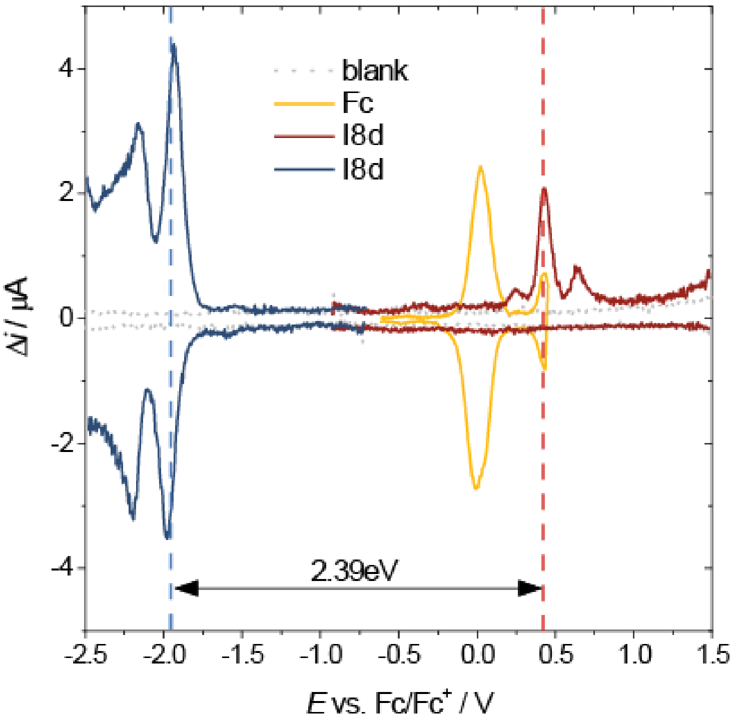

Figure S 17. Differential pulse voltammograms recorded for **I8d** (anodic scan – red line, cathodic scan – blue line) and ferrocenium (orange line) in 50 mM TBAP in DCM on Pt electrode; T = 268 K; P_H = ±2.5 mV, P_W = 100 ms, S_t = 500 ms, S_H = ±5 mV.

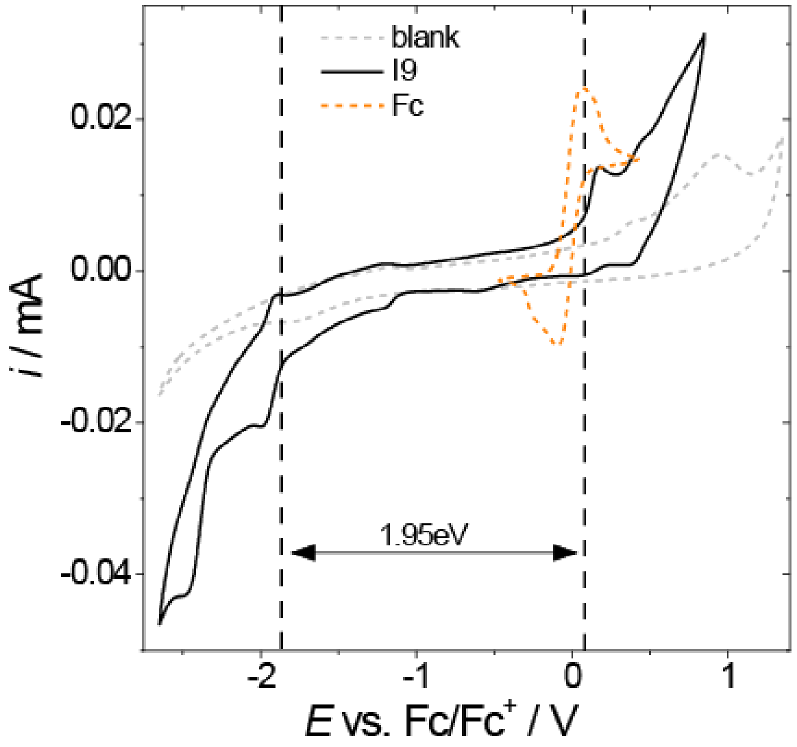

Figure S 18. Cyclic voltammograms recorded for **I9** (black solid line) and ferrocenium (orange dotted line) in 50mM TBAP in DCM on Pt electrode; T = 268K; sweep rate v = 100mV s^{-1}.

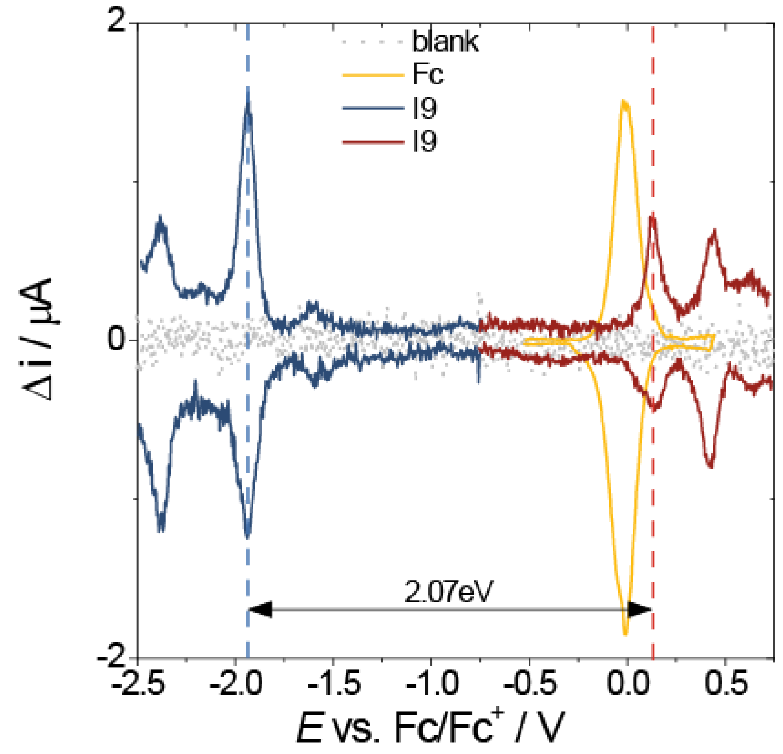

Figure S 19. Differential pulse voltammograms recorded for **I9** (anodic scan – red line, cathodic scan – blue line) and ferrocenium (orange line) in 50 mM TBAP in DCM on Pt electrode; T = 268 K; P_H = ±2.5 mV, P_W = 100 ms, S_t = 500 ms, S_H = ±5 mV.

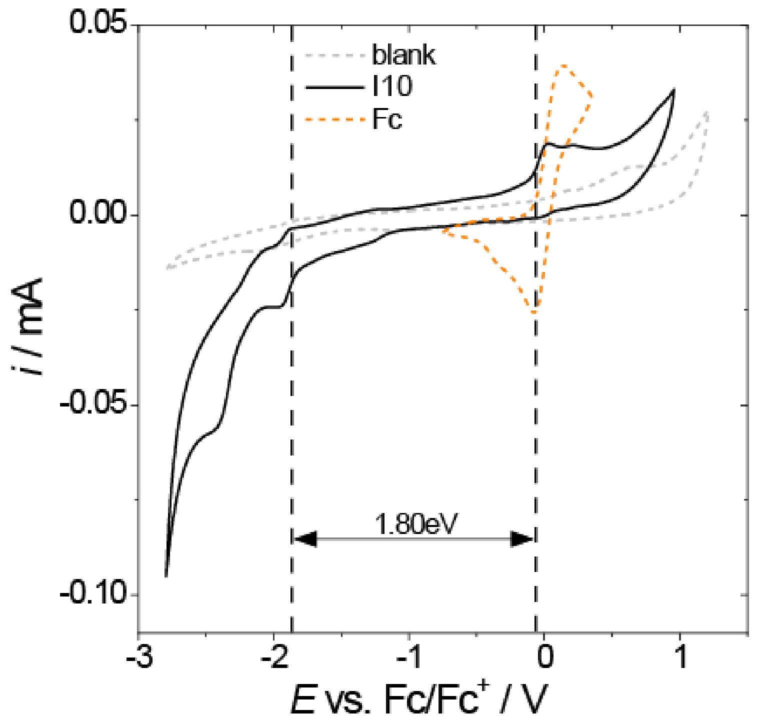

Figure S 20. Cyclic voltammograms recorded for **I10** (black solid line) and ferrocenium (orange dotted line) in 50mM TBAP in DCM on Pt electrode; T = 268K; sweep rate v = 100mV s^{-1}.

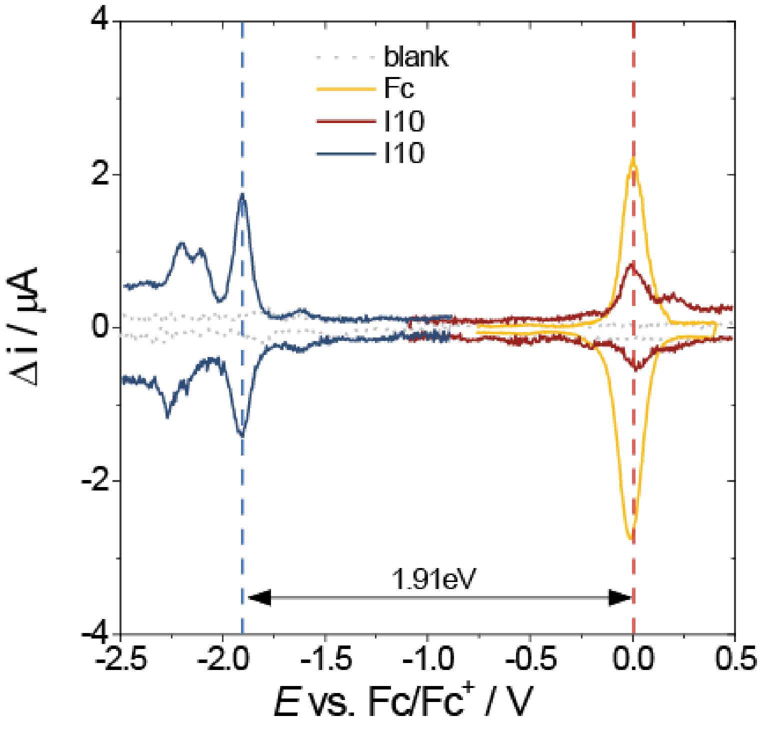

Figure S 21. Differential pulse voltammograms recorded for **I10** (anodic scan – red line, cathodic scan – blue line) and ferrocenium (orange line) in 50 mM TBAP in DCM on Pt electrode; T = 268 K; P_H = ±2.5 mV, P_W = 100 ms, S_t = 500 ms, S_H = ±5 mV.

Table S 1. Summary of electrochemical data obtained with Cyclic Voltammetry (CV)

Cpd	E'$_{cat}$ (V vs. Fc/Fc^{+})	E'$_{an}$ (V vs. Fc/Fc^{+})	ΔE$_{peak}$ (V)	ΔE$_{onset}$ (V)	ΔE$_{cat}$ (V)	ΔE$_{an}$ (V)
**I1**	−1.634	0.242	1.711	1.678		0.33
**I5**	−2.086	0.3795	2.495	2.438	0.168	0.109
**I6**	−1.957	0.477	2.502	2.299	0.086	0.05
**I8a**	−2.181	−0.158	2.041	1.938	0.25	0.214
**I8b**	−2.193	0.0935	2.294	2.219	0.294	0.279
**I8d**	−1.976	0.3745	2.356	2.258	0.122	0.111
**I9**	−1.9405	0.102	2.01	1.946	0.071	0.136
**I10**	−1.9065	−0.0175	1.901	1.8	0.077	0.053
Rubrene	−2.2205	0.1905	2.416	2.195	0.123	0.113

Table S 2. Summary of electrochemical data obtained with differential pulse voltammetry and comparison of bandgap measures with different electrochemical methods.

Cpd	E'$_{cat}$ (V vs. Fc/Fc^{+})	E'$_{an}$ (V vs. Fc/Fc^{+})	ΔE$_{peak}$ (V)	ΔE$_{onset}$ (V)	ΔE$_{cat}$ (V)	ΔE$_{an}$ (V)	ΔE$_{peak}$(CV) – E$_{peak}$(DPV) (V)
**I1**	-	0.350	-	-	-	0.074	-
**I5**	−2.111	0.376	2.488	2.335	0.029	0.111	0.008
**I6**	−1.942	0.366	2.308	2.232	0.046	0.026	0.194
**I8a**	−2.185	−0.212	1.973	1.680	0.034	0.164	0.068
**I8b**	−2.118	0.175	2.293	2.094	0.010	0.086	0.001
**I8d**	−1.968	0.426	2.394	2.209		0.085	−0.038
**I9**	−1.938	0.133	2.070	2.275	0	0.106	−0.060
**I10**	−1.910	0.044	1.954	1.730	0.108	0.090	−0.053
Rubrene	−2.333	0.135	2.468	2.140	0.004	0.191	−0.052

S5. X-Ray Crystallographic Analysis

Structure of I6

A pink cube-like crystals of **I6** were grown by slow vapor diffusion of hexanes to CH_2Cl_2 solution. Crystal of approximate dimensions 0.334 mm x 0.365 mm x 0.508 mm, was used for the X-ray crystallographic analysis. A total of 4043 frames were collected. The total exposure time was 38.18 hours. CCDC number for **I6:** 2332189

The frames were integrated with the Bruker SAINT software package using a narrow-frame algorithm. The integration of the data using a monoclinic unit cell yielded a total of 78224 reflections to a maximum θ angle of 68.51° (0.83 Å resolution), of which 13451 were independent (average redundancy 5.815, completeness = 95.4%, R_{int} = 6.97%, R_{sig} = 4.09%) and 10178 (75.67%) were greater than 2σ(F^2). The final cell constants of a = 13.857(2) Å, b = 19.166(3) Å, c = 29.076(4) Å, β = 97.384(10)°, volume = 7658.(2) Å^3, are based upon the refinement of the XYZ-centroids of 9756 reflections above 20 σ(I) with 5.536° < 2θ < 136.5°. Data were corrected for absorption effects using the multi-scan method (SADABS). The ratio of minimum to maximum apparent transmission was 0.802. The calculated minimum and maximum transmission coefficients (based on crystal size) are 0.6210 and 0.7230.

The structure was solved and refined using the Bruker SHELXTL Software Package, using the space group P 1 21/n 1, with Z = 8 for the formula unit, $C_{42}H_{53}NSi_2$. The final anisotropic full-matrix least-squares refinement on F^2 with 841 variables converged at R1 = 8.34%, for the observed data and wR2 = 27.36% for all data. The goodness-of-fit was 0.981. The largest peak in the final difference electron density synthesis was 0.757 e^-/Å^3 and the largest hole was –0.403 e^-/Å^3 with an RMS deviation of 0.086 e^-/Å^3. On the basis of the final model, the calculated density was 1.089 g/cm^3 and F(000), 2720 e^-.

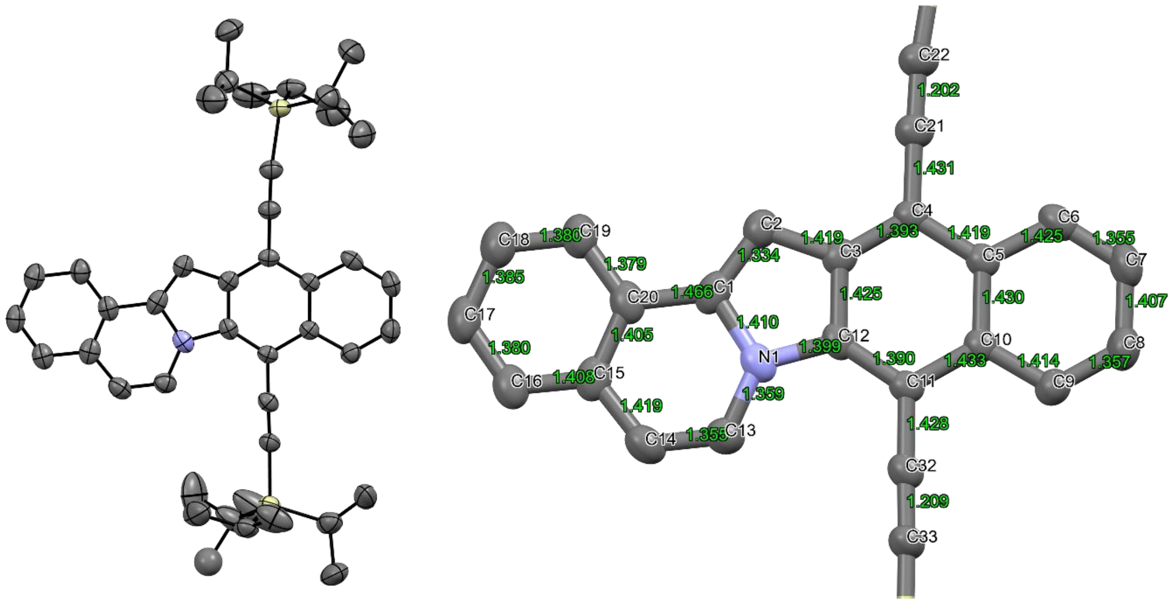

Figure S 22. (left) ORTEP plot for single crystal structure of X. Atomic displacement parameters at 160 K are drawn at 50 % probability level. H atoms are omitted for clarity. (right) Bond lengths of the polycyclic aromatic core of **I6**.

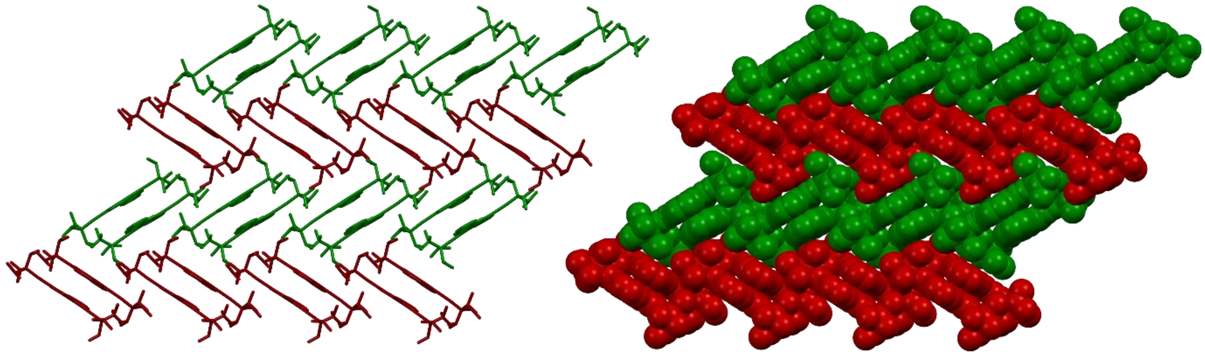

Figure S 23. Crystal packing of **I6** represented as a stick model (left) and space fill model (right).

S6. Quantum Chemical Calculations

The molecular structures of compounds were optimized using the given level of theory with Gaussian 16, Revision C.01.[7] Solvation was considered using the polarizable continuum model (PCM). The structures, optimized at DFT B3LYP[8, 9]/def2-TZVPP[10, 11] computational level, were confirmed as ground-state minima by analyzing their analytical frequencies, which indicated the absence of any imaginary frequencies. The vertical transition energies were computed using time-dependent density functional theory (TD-DFT). Triisopropylsilyl groups were substituted with trimethylsilyl groups, which does not significantly affect the electronic properties but substantially reduces computational time and costs.

Calculations of optoelectronic properties

Frontier molecular orbitals of representative examples.

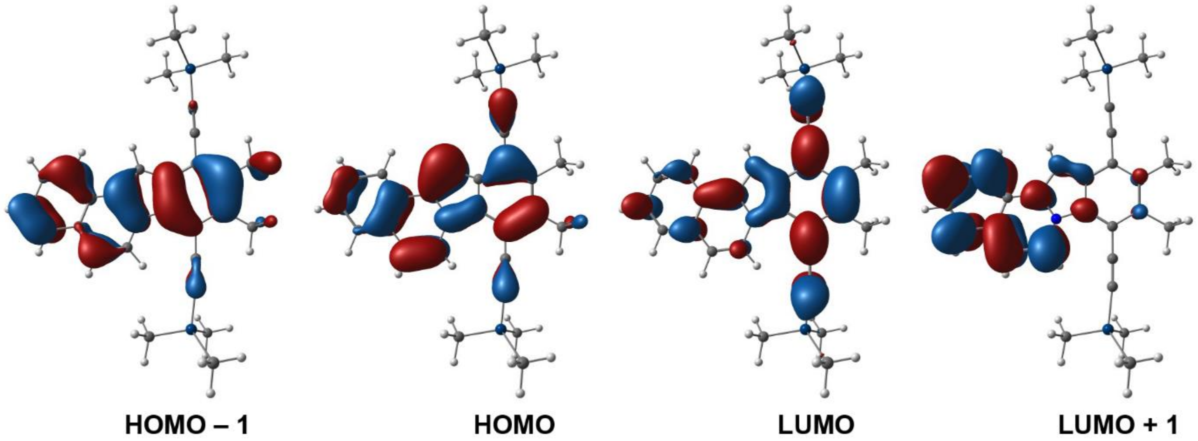

Figure S 24. Frontier molecular orbitals of **I1**

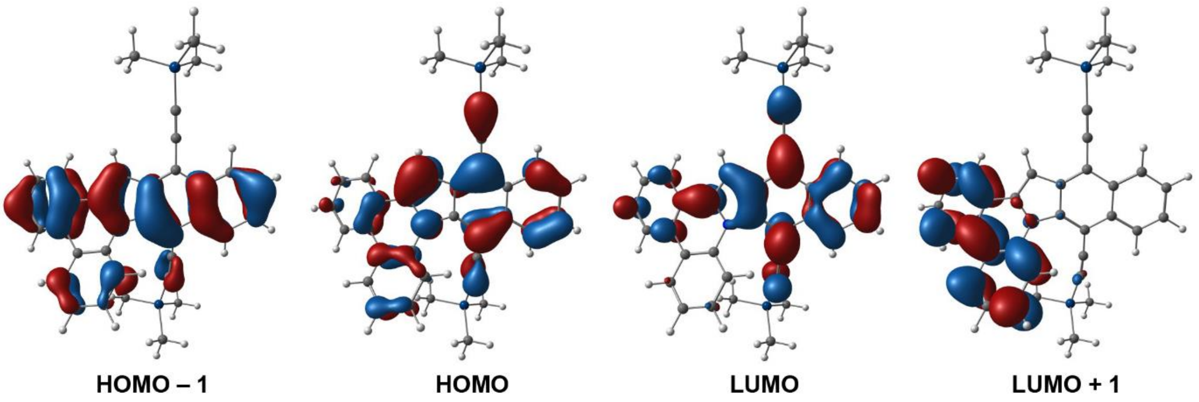

Figure S 25. Frontier molecular orbitals of **I5.**

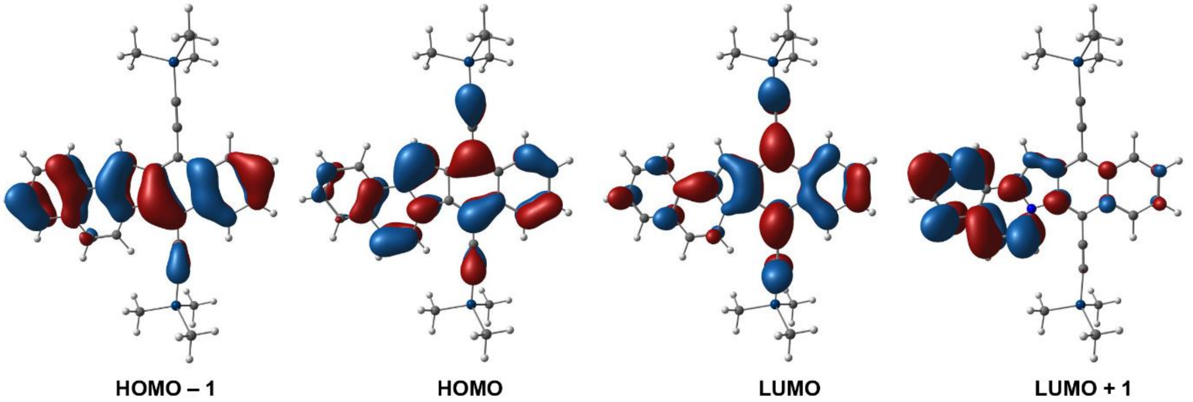

Figure S 26. Frontier molecular orbitals of **I6.**

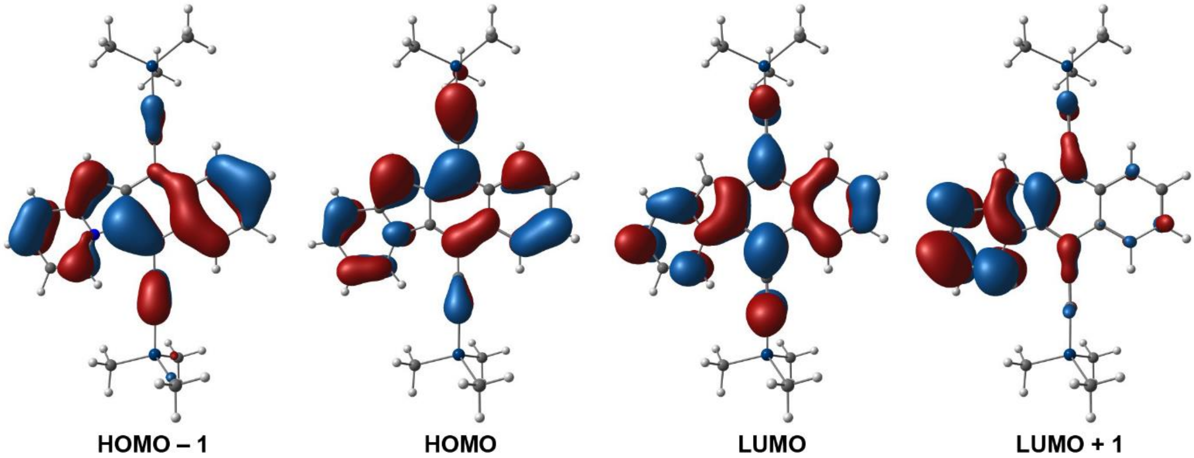

Figure S 27. Frontier molecular orbitals of **I8a.**

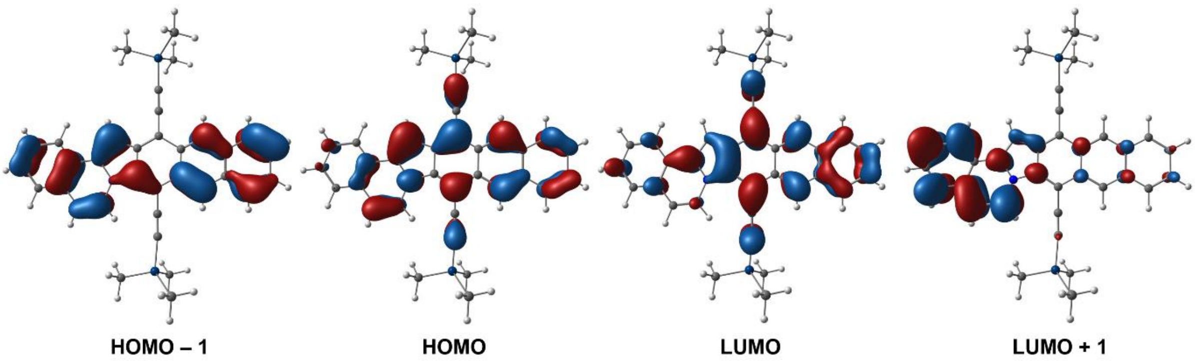

Figure S 28. Frontier molecular orbitals of **I9.**

Table S 3. Calculated parameters of HOMO-to-LUMO transitions of selected compounds **I.**

Compound	Transition energy		Oscillator strength	Transition FMOs
	nm	eV		
**I1**	366.60	3.3820	0.4454	117 ->118
**I5**	424.02	2.9240	0.5452	135 ->136
**I6**	448.34	2.7654	0.4419	122 ->123
**I8a**	480.07	2.5827	0.3940	109 ->110
**I9**	522.05	2.3749	0.3761	135 ->136

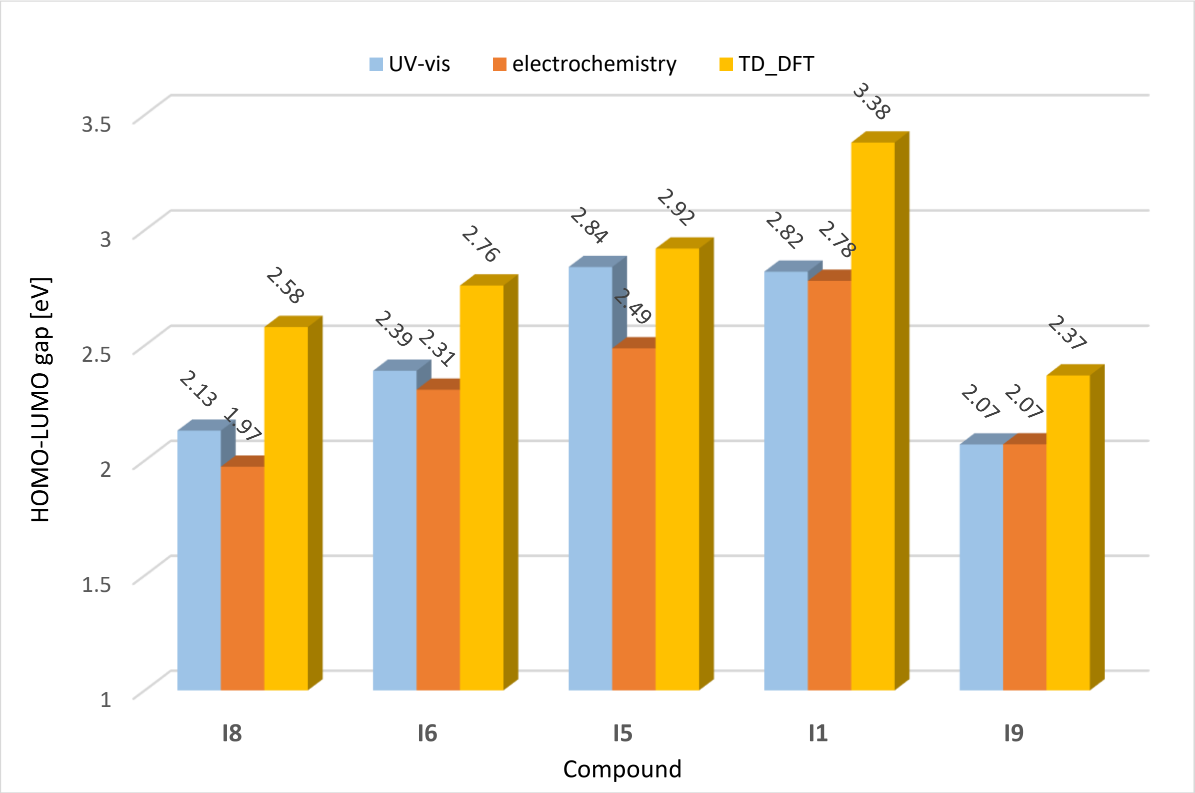

Figure S 29. Comparison of optical (blue) electrochemical (DPV ΔE_{onset}, orange) and computed optical (TD-DFT, yellow) band gaps of representative compounds.

Calculations of Aromatic Ring Currents and Nucleus Independent Chemical Shifts (NICS)

Aromatic ring currents and nucleus-independent chemical shifts (NICS) were computed to investigate the electronic properties and aromaticity of the studied indoloindolizines. These calculations provide insights into the magnetic shielding effects associated with aromatic systems, which are critical for understanding the properties of these compounds. Aromatic ring currents and NICS values were initially calculated at a distance of 1 bohr (0.53 Å) above the plane of the polycyclic aromatic core, as this position maximizes the detection of ring currents due to the close proximity to the π-electron cloud.[12] However, NICS values were also calculated at a greater distance of 1.7 Å above the aromatic plane because, at 0.53 Å, the influence of σ-electrons and localized bonding interactions can become significant, potentially leading to misleading interpretations of aromaticity. According to Stanger and Gershoni-Poranne, at the 1.7 Å height, these σ-bond effects are minimized, allowing for a more accurate assessment of the π-electron contributions to the aromatic character.[13]

All model structures were optimized at the B3LYP/def2-TZVPP level of theory. Aromatic ring current representations were generated using the AIMAll software, which utilized GIAO (Gauge-Independent Atomic Orbital) NMR calculations conducted with Gaussian at the B3LYP/def2-TZVPP level of theory.[7, 14] NICS calculations were also performed using Gaussian software at the B3LYP/def2-TZVPP level. A grid of ghost atoms (Bq) was placed 0.2 Å apart at a specified height above the molecular plane, where GIAO NMR calculations were performed to capture the aromaticity effects. The extracted isovalues were then visualized using a custom-built Python script, which employs the following libraries:

- **Pandas**: For reading and manipulating data from Excel files (*pd.read_excel()*).
- **Matplotlib**: For creating and displaying 2D grid plots (*plt.contourf(), plt.colorbar()*, etc.).
- **NumPy**: For numerical operations, including creating grids for plotting (*np.mgrid()*).
- **SciPy**: Specifically, the *griddata* function from the *scipy.interpolate* module is used to interpolate data points onto a grid for contour plotting.

Below, graphical representations of these results for the unsubstituted model derivatives **M1**–**M10** of the studied indoloindolizines are presented. In current density profiles clockwise arrows represent diatropic ring currents, while counterclockwise arrows represent paratropic ring currents. Scales for NICS plots are shown in Figures S30 and S31.

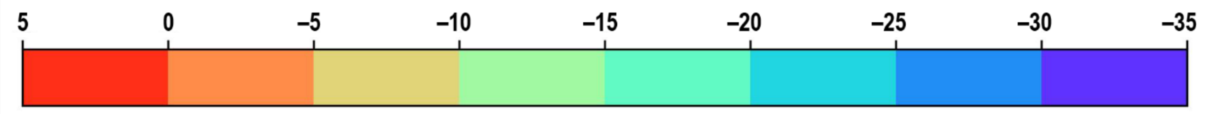

Figure S 30. Isovalue scale for NICS plots at the height 0,53 Å above the molecular plane.

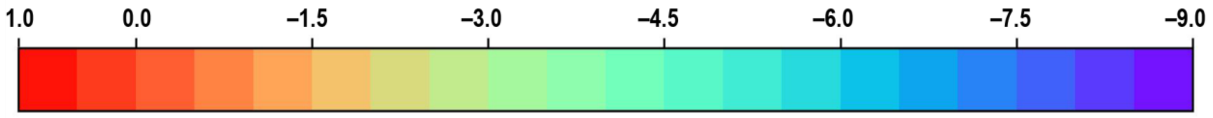

Figure S 31. Isovalue scale for NICS plots at the height 1.7 Å above the molecular plane.

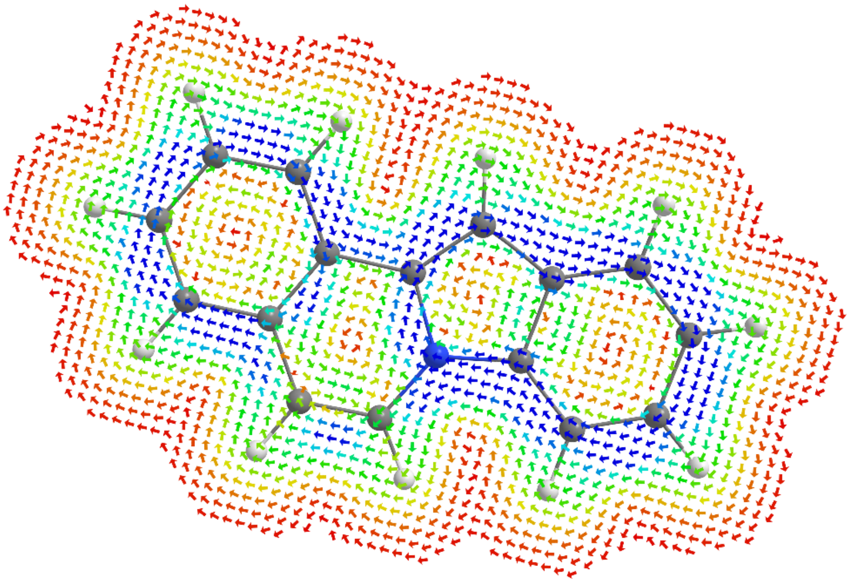

Figure S 32. Current density profile of **M1**.

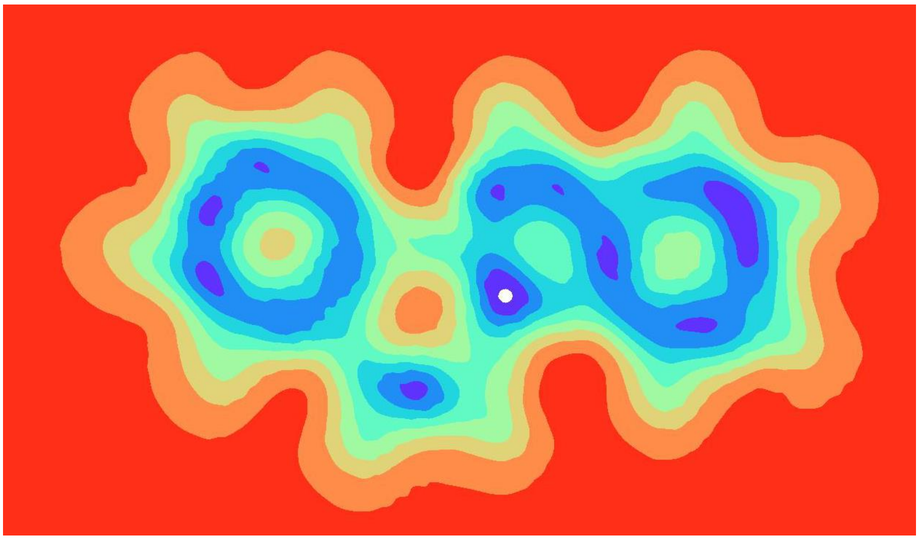

Figure S 33. NICS grid plot 0.53 Å above the molecular plane of **M1**.

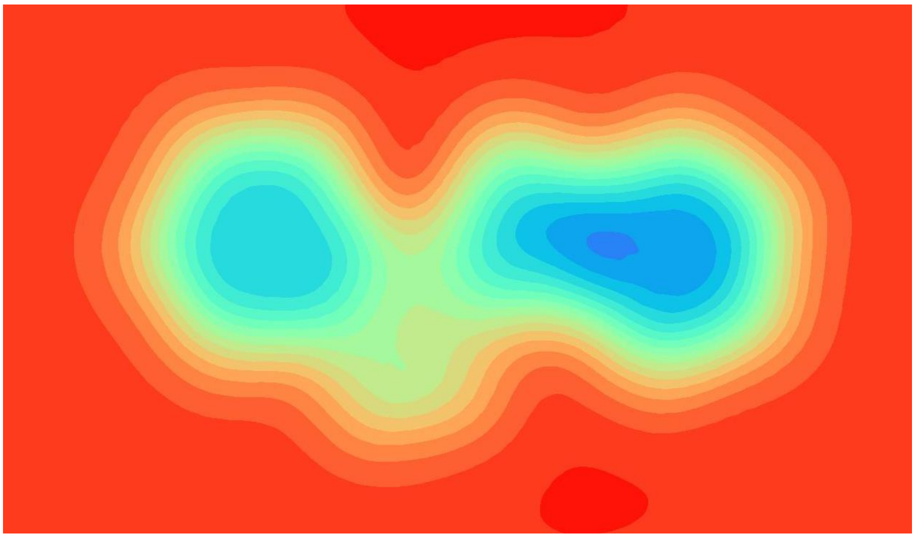

Figure S 34. NICS grid plot 1.7 Å above the molecular plane of **M1**.

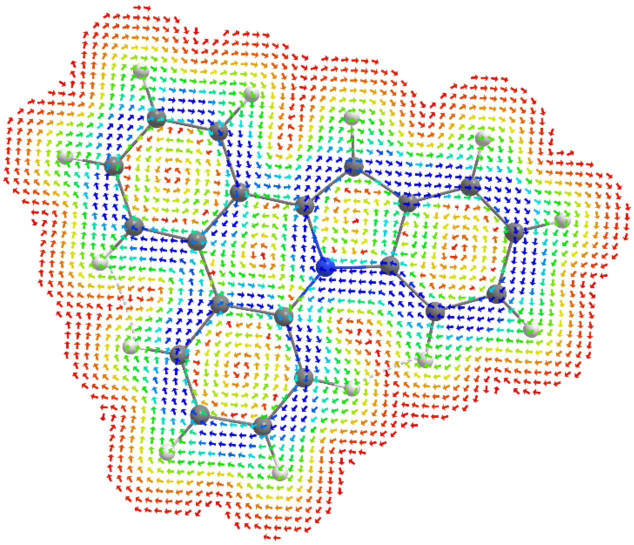

Figure S 35. Current density profile of **M2**.

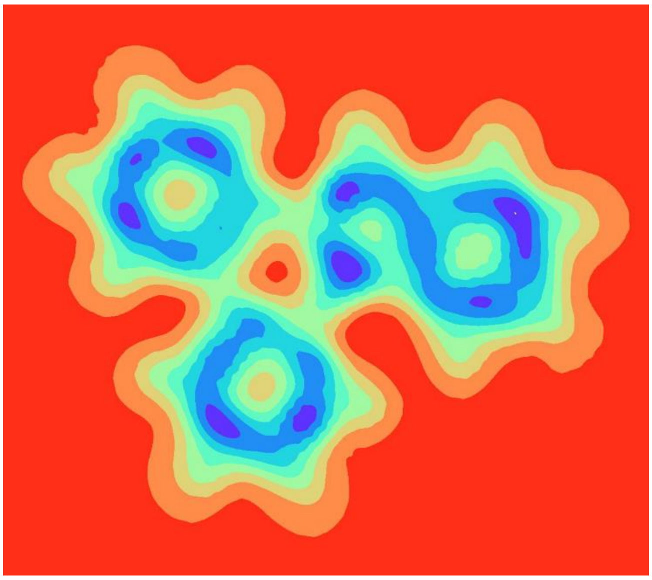

Figure S 36. NICS grid plot 0.53 Å above the molecular plane of **M2**.

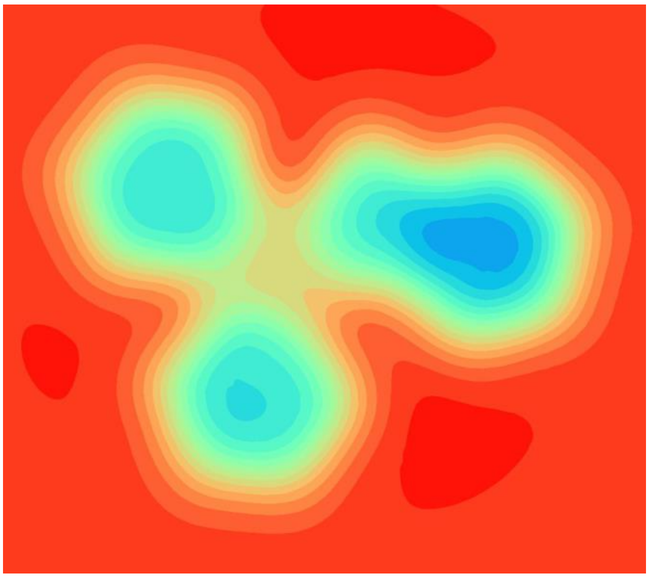

Figure S 37. NICS grid plot 1.7 Å above the molecular plane of **M2**.

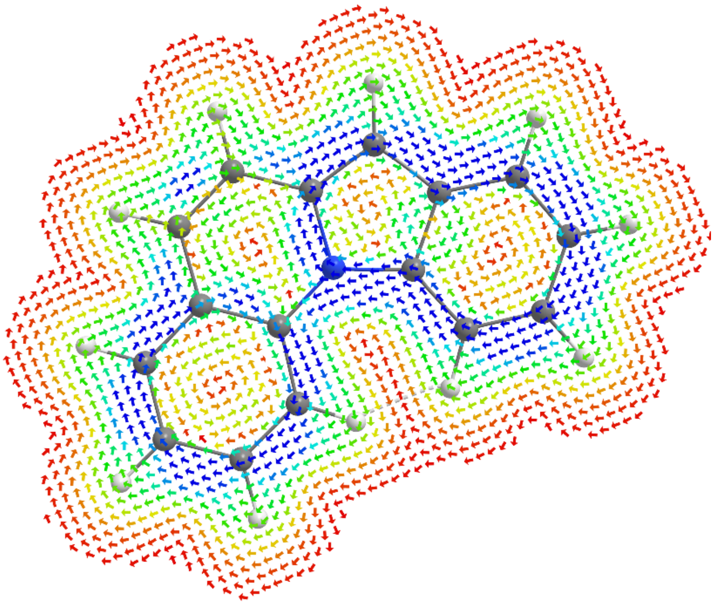

Figure S 38. Current density profile of **M3**.

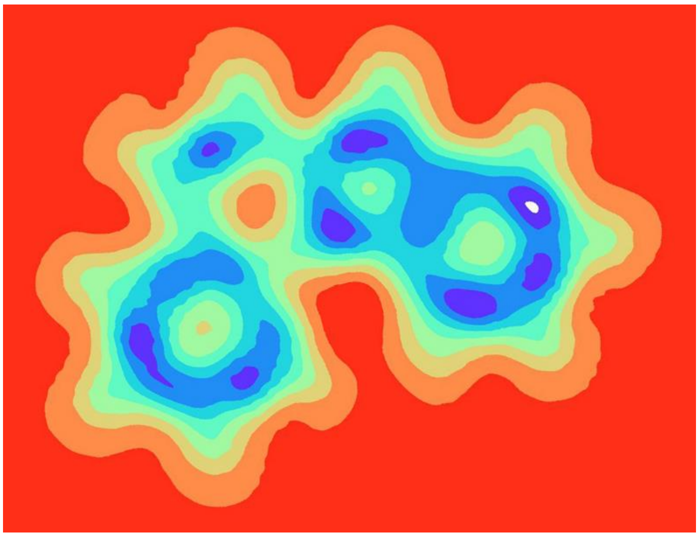

Figure S 39. NICS grid plot 0.53 Å above the molecular plane of **M3**.

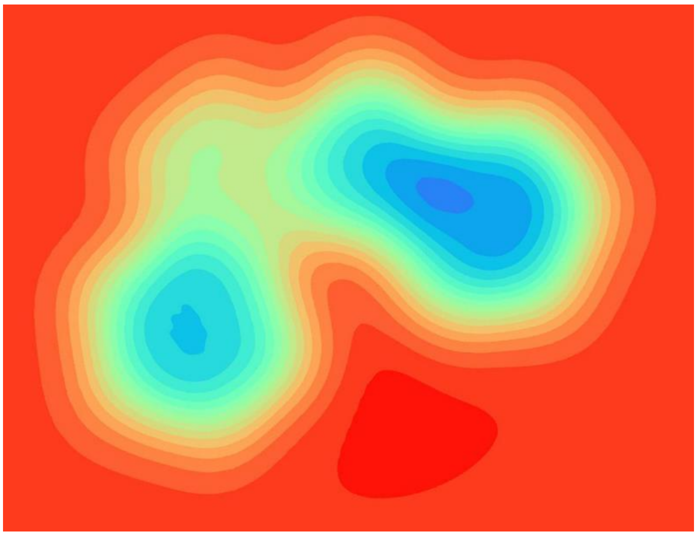

Figure S 40. NICS grid plot 1.7 Å above the molecular plane of **M3**.

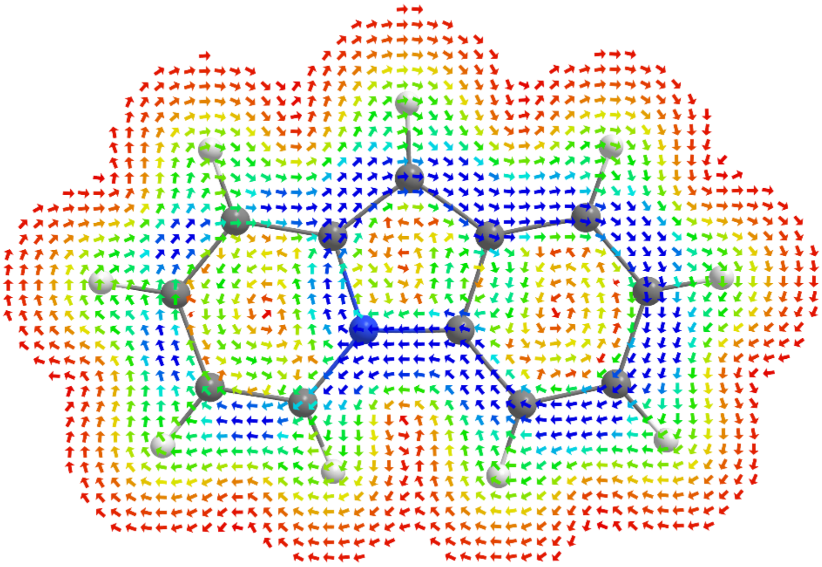

Figure S 41. Current density profile of **M4**.

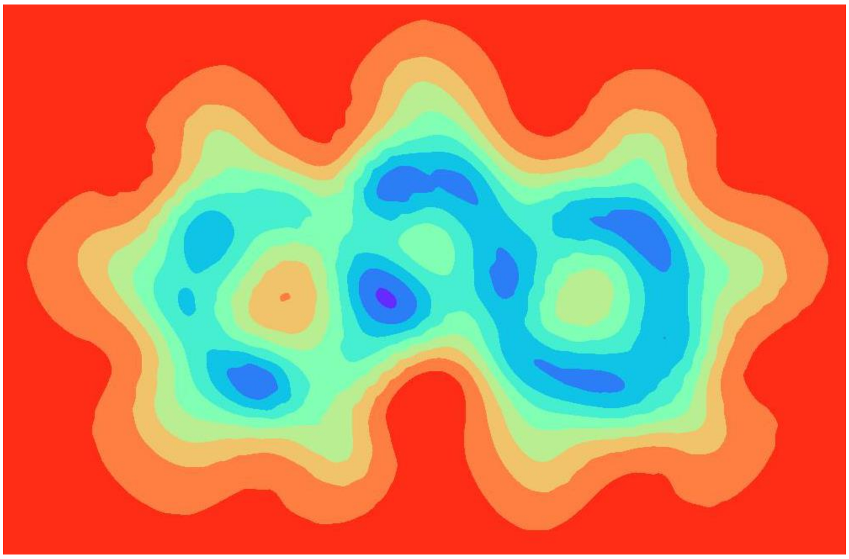

Figure S 42. NICS grid plot 0.53 Å above the molecular plane of **M4**.

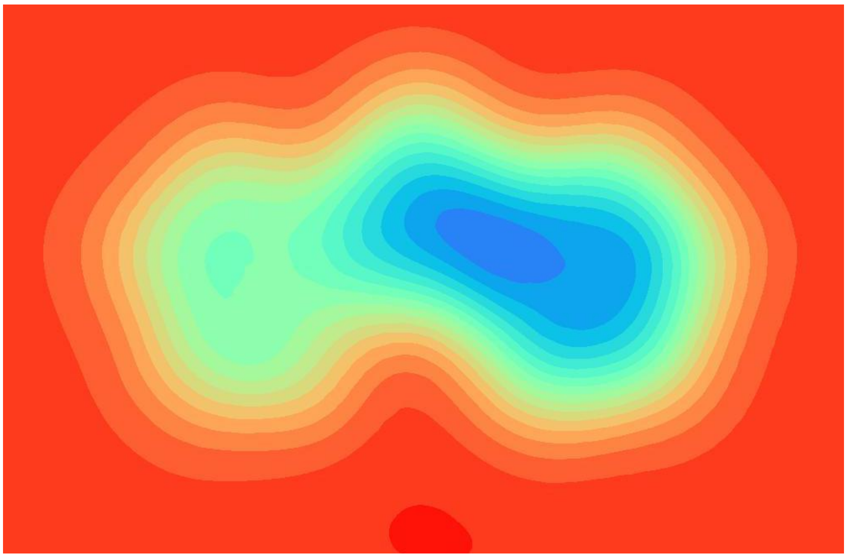

Figure S 43. NICS grid plot 1.7 Å above the molecular plane of **M4**.

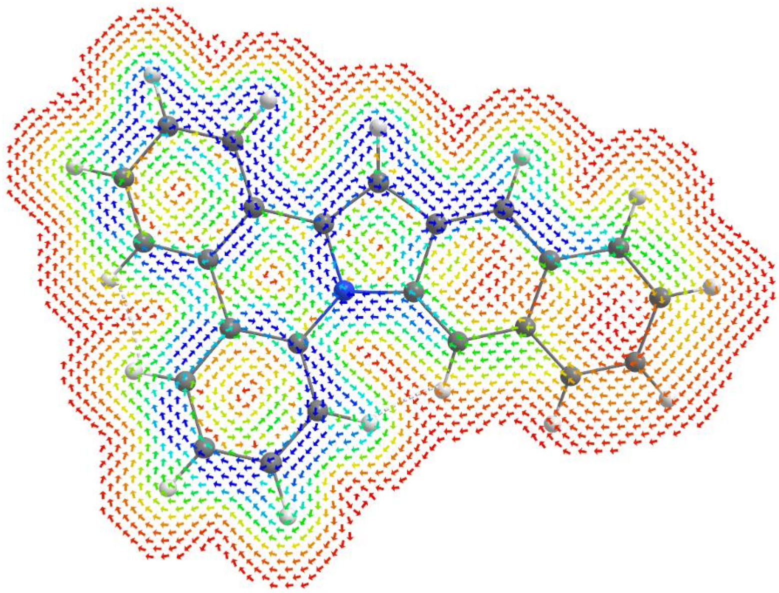

Figure S 44. Current density profile of **M5**.

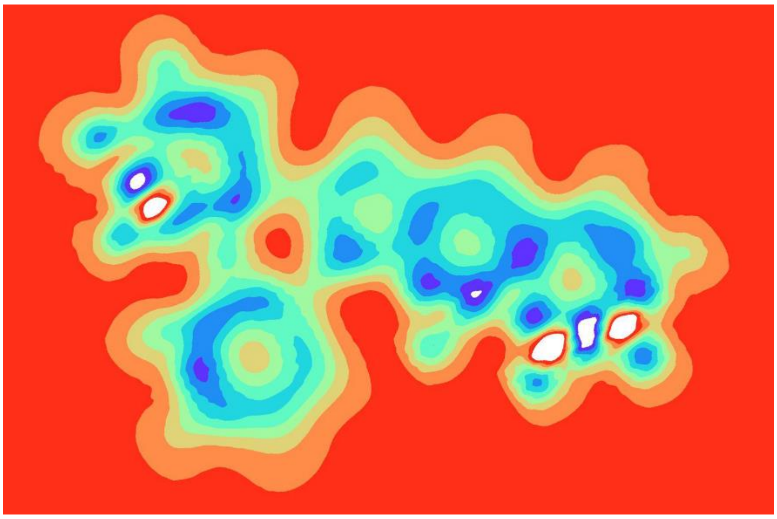

Figure S 45. NICS grid plot 0.53 Å above the molecular plane of **M5**. Irregularities are caused the curvature of the π-system.

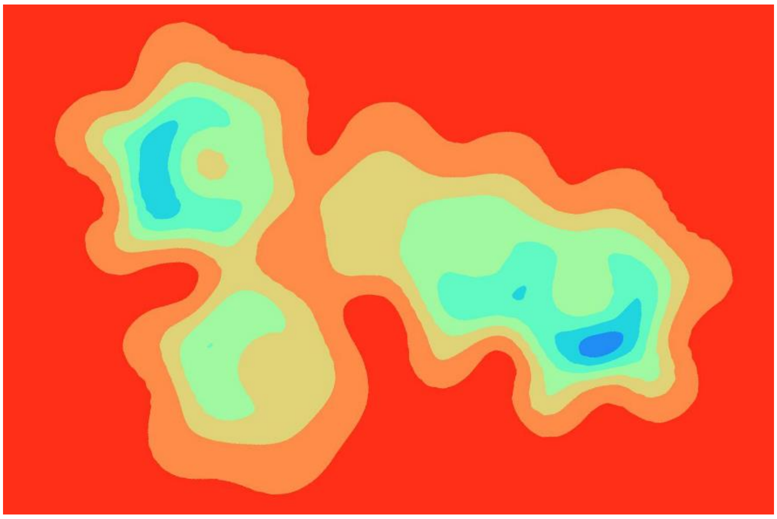

Figure S 46. Figure S 47. NICS grid plot 1.7 Å above the molecular plane of **M5**. The same scale as in 0.53 Å used because of the curvature of the π-system.

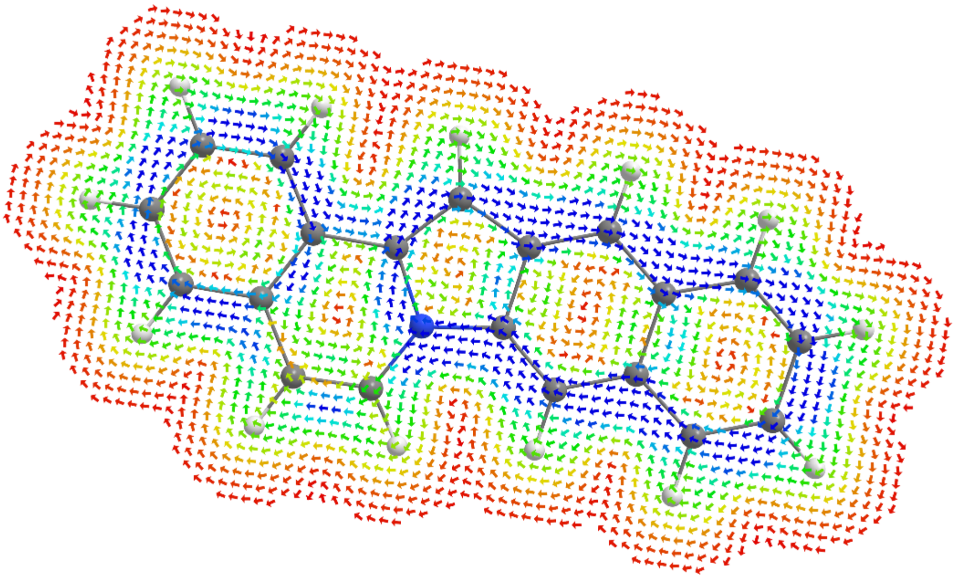

Figure S 48. Current density profile of **M6**.

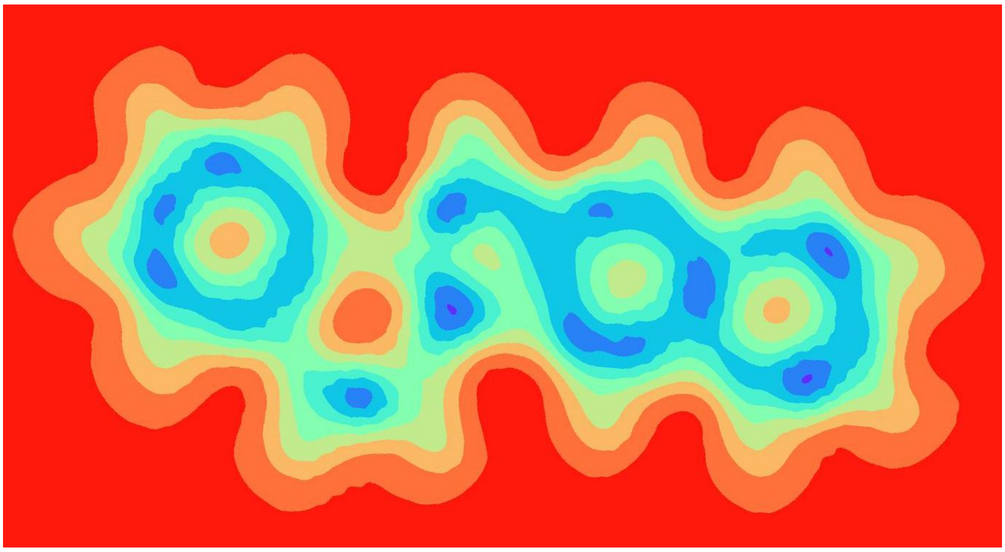

Figure S 49. NICS grid plot 0.53 Å above the molecular plane of **M6**.

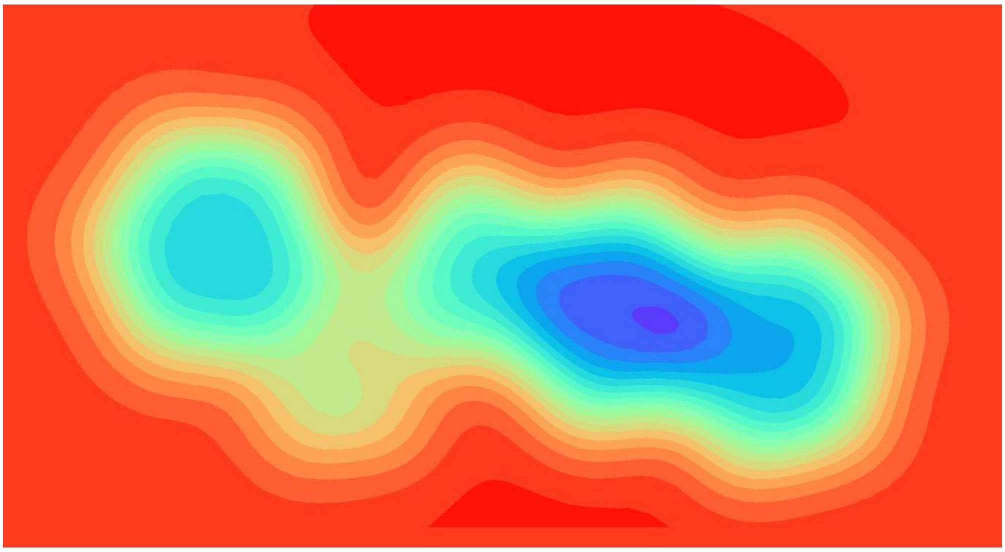

Figure S 50. NICS grid plot 1.7 Å above the molecular plane of **M6**.

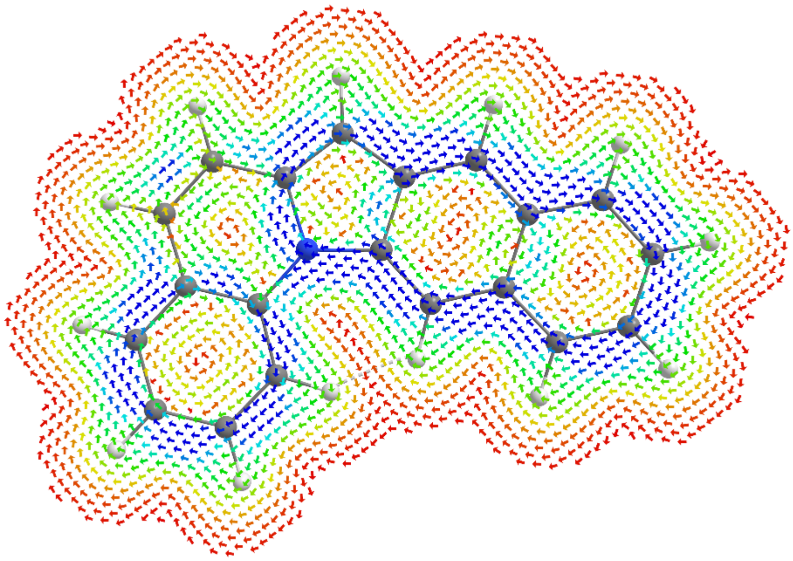

Figure S 51. Current density profile of **M7**.

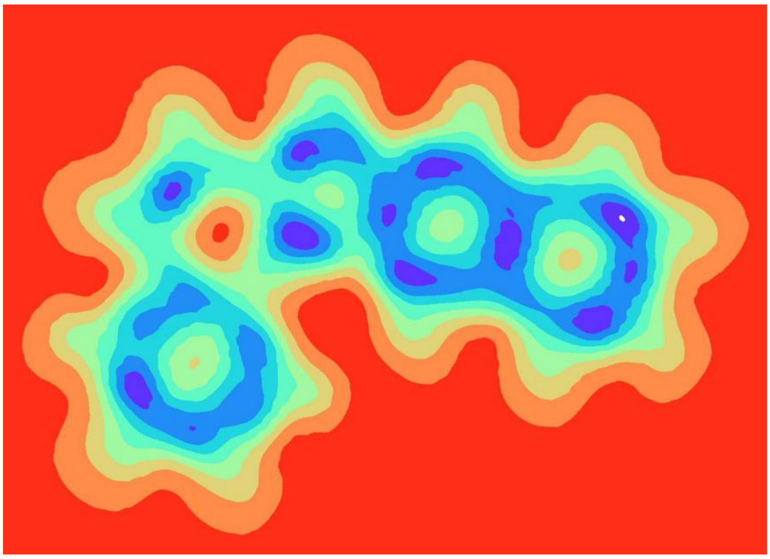

Figure S 52. NICS grid plot 0.53 Å above the molecular plane of **M7**.

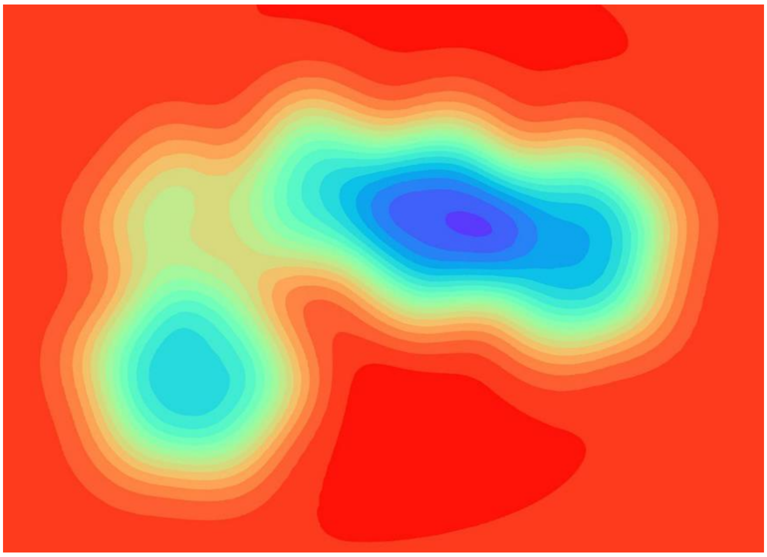

Figure S 53. NICS grid plot 1.7 Å above the molecular plane of **M7**.

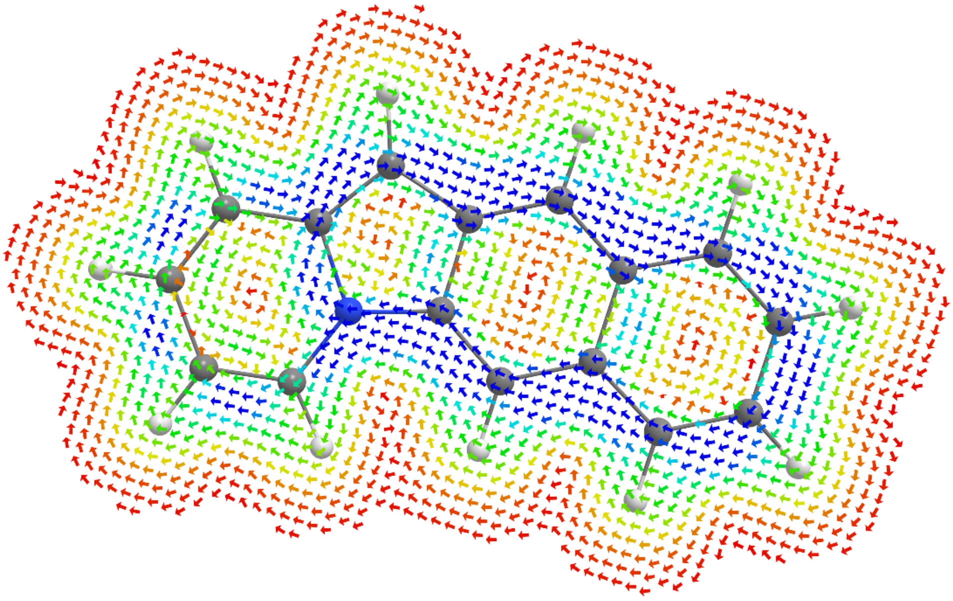

Figure S 54. Current density profile of **M8a**.

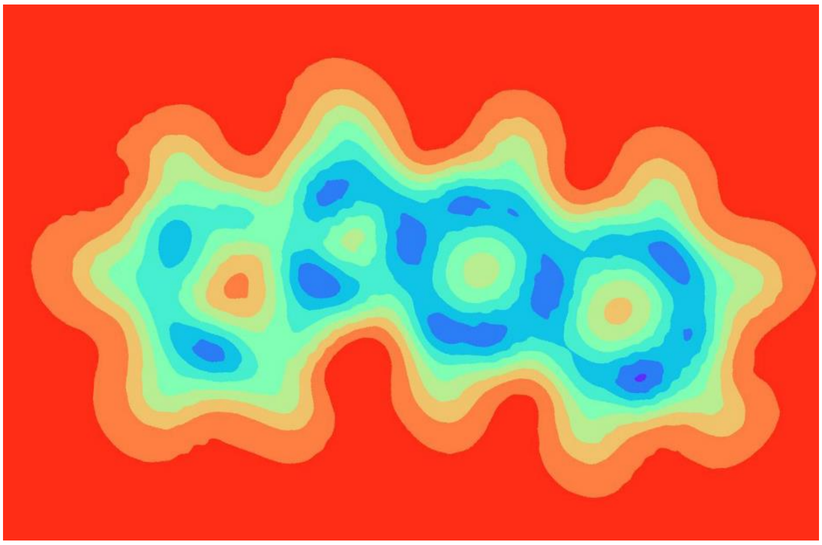

Figure S 55. NICS grid plot 0.53 Å above the molecular plane of **M8a**.

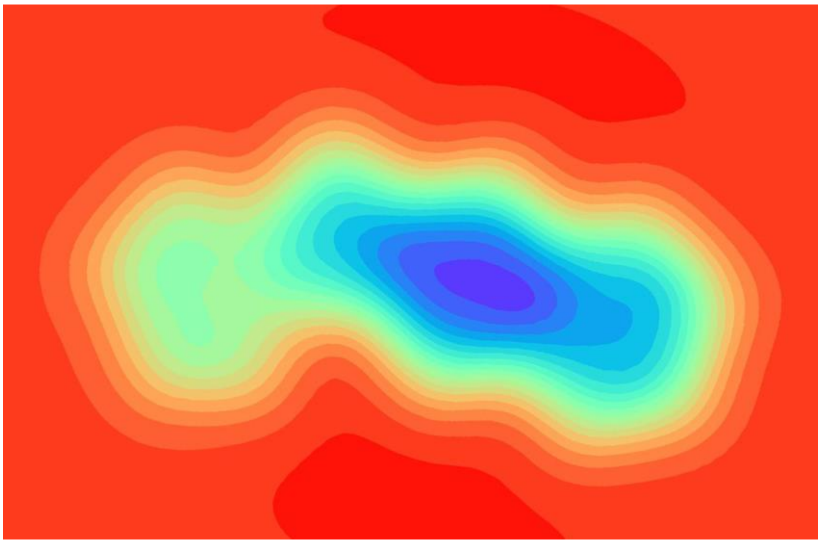

Figure S 56. NICS grid plot 1.7 Å above the molecular plane of **M8a**.

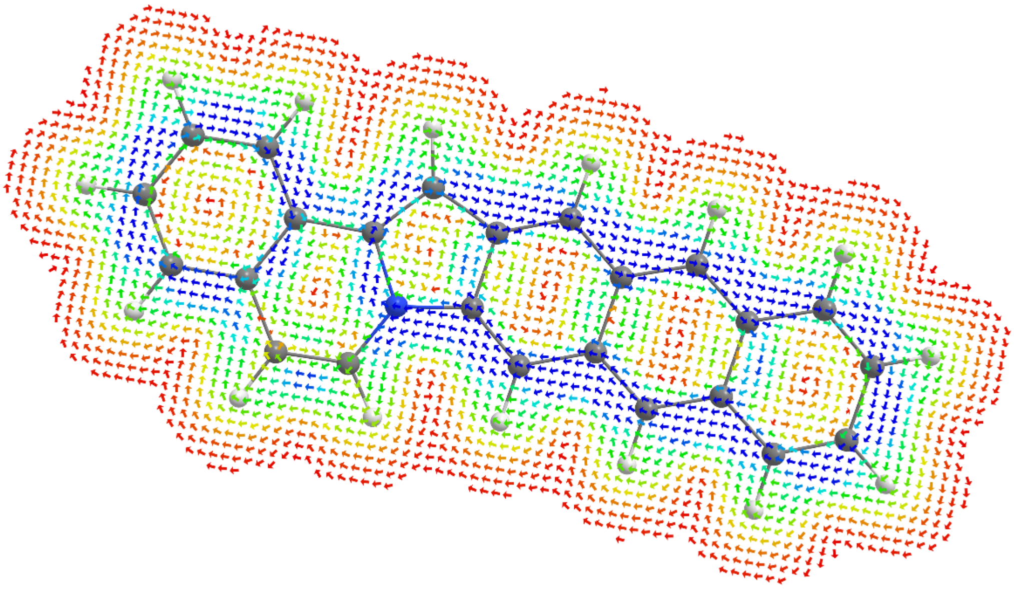

Figure S 57. Current density profile of **M9**.

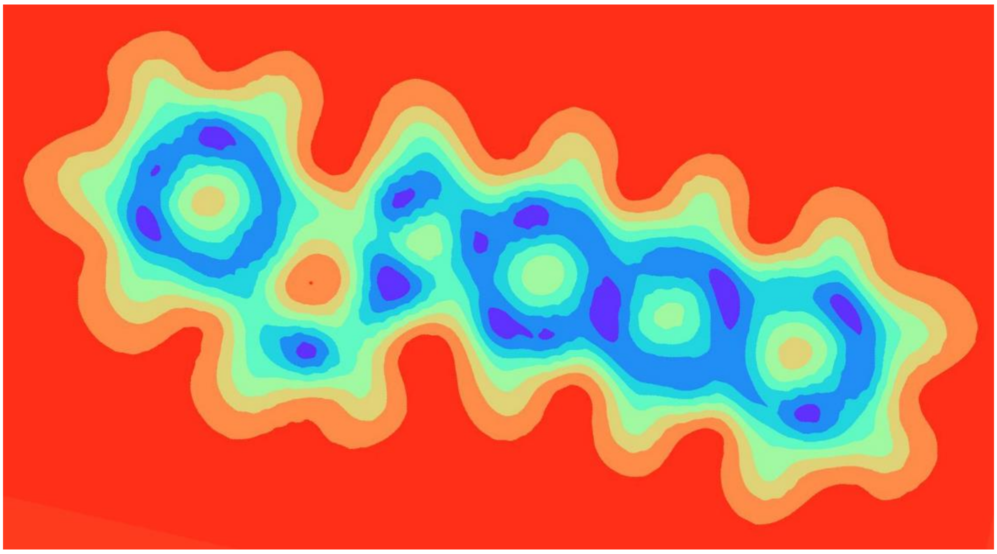

Figure S 58. NICS grid plot 0.53 Å above the molecular plane of **M9**.

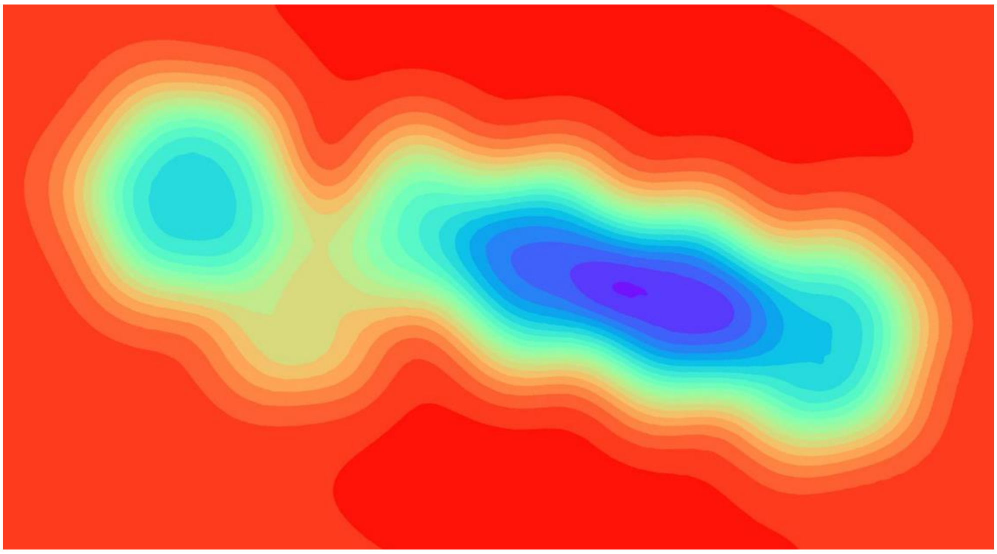

Figure S 59. NICS grid plot 1.7 Å above the molecular plane of **M9**.

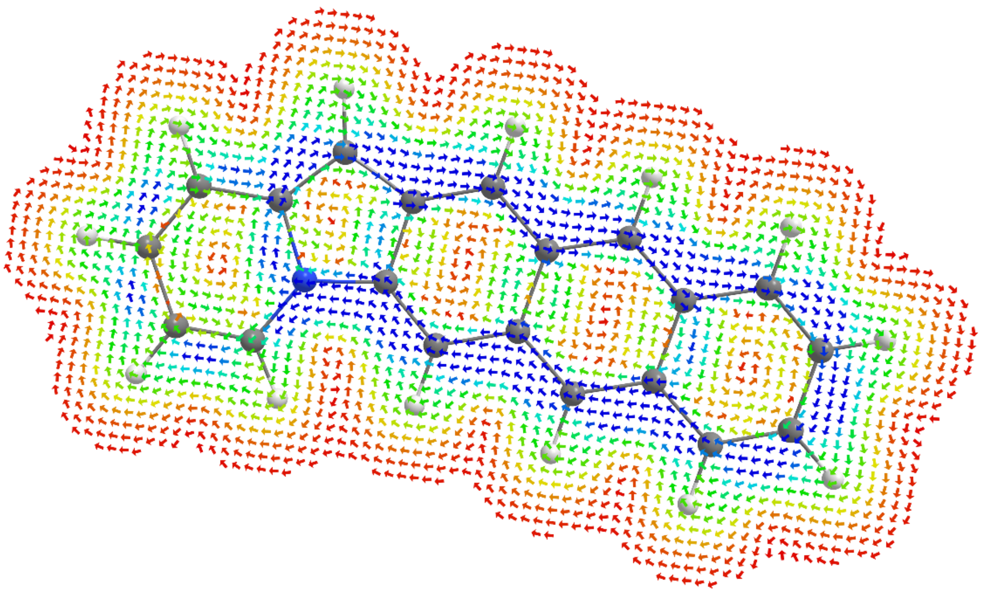

Figure S 60. Current density profile of **M10**.

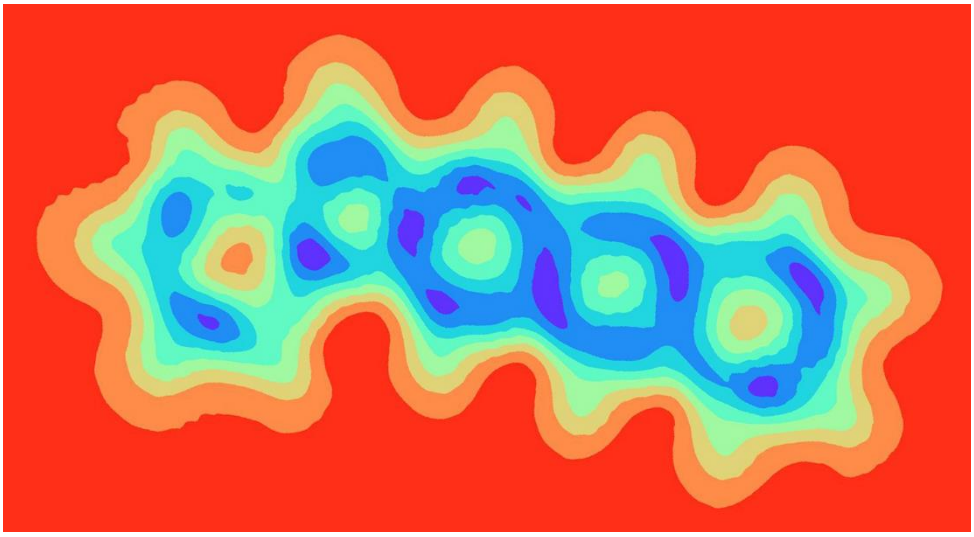

Figure S 61. NICS grid plot 0.53 Å above the molecular plane of **M10**.

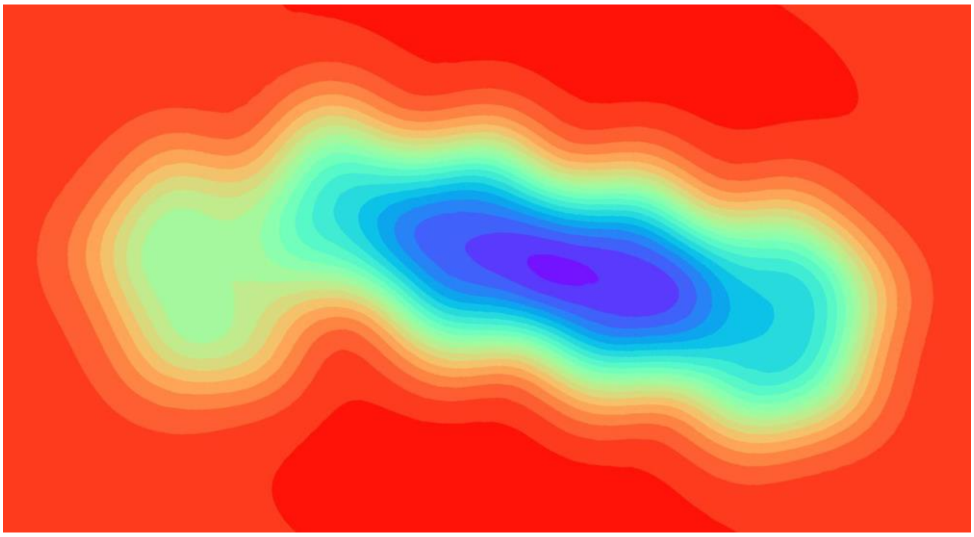

Figure S 62. NICS grid plot 1.7 Å above the molecular plane of **M10**.

Calculations of aromatic stabilization energy

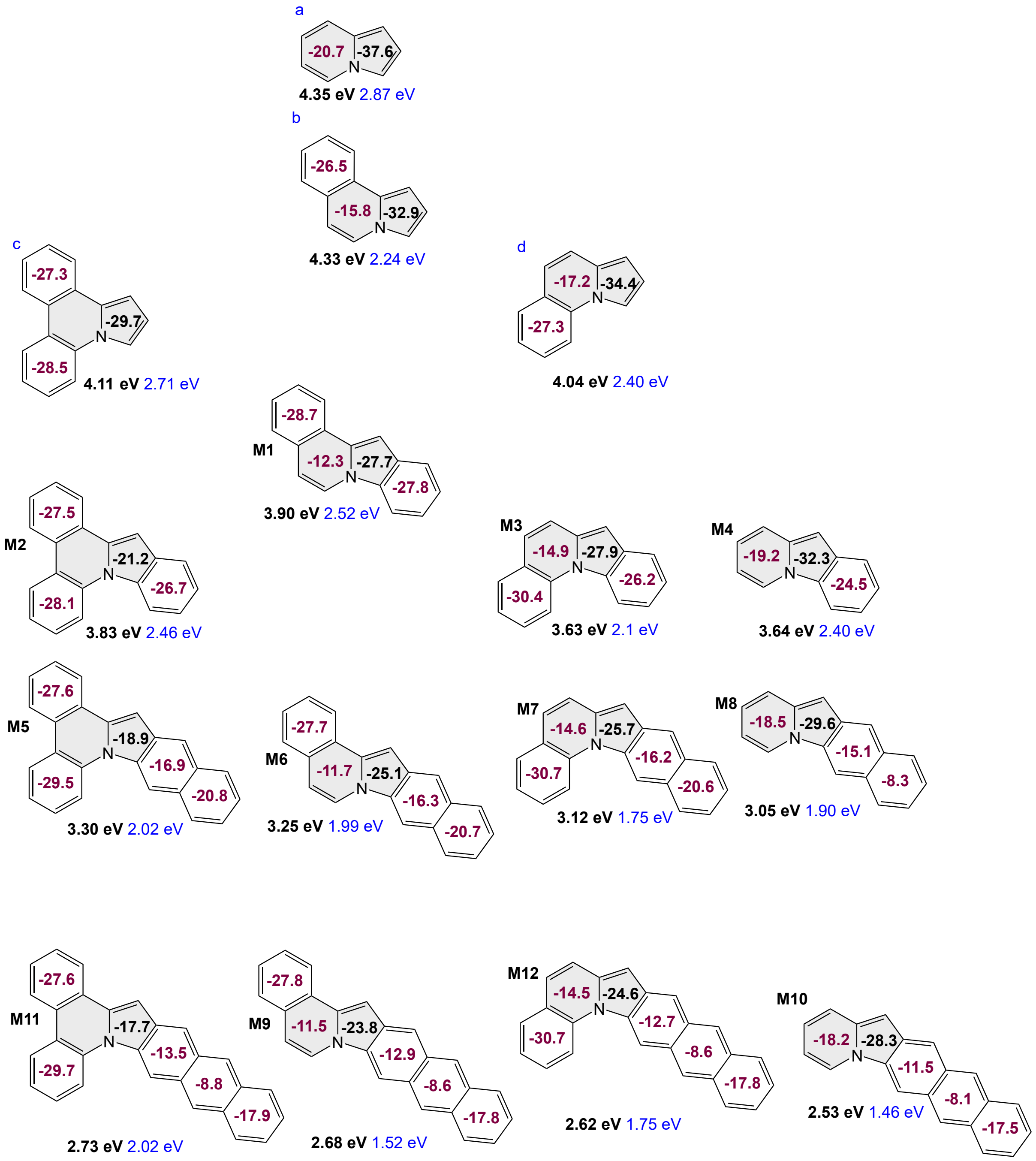

Figure S 63. Extended periodic table of π-expanded indoloindolizines. The purple numbers in each ring denote the ISE of the ring. The black number in the 5-membered ring provides ISE for the indolizine moiety. Below each molecule, their band gaps computed at the DFT (black bold) and extended Huckel (blue regular) levels are presented.

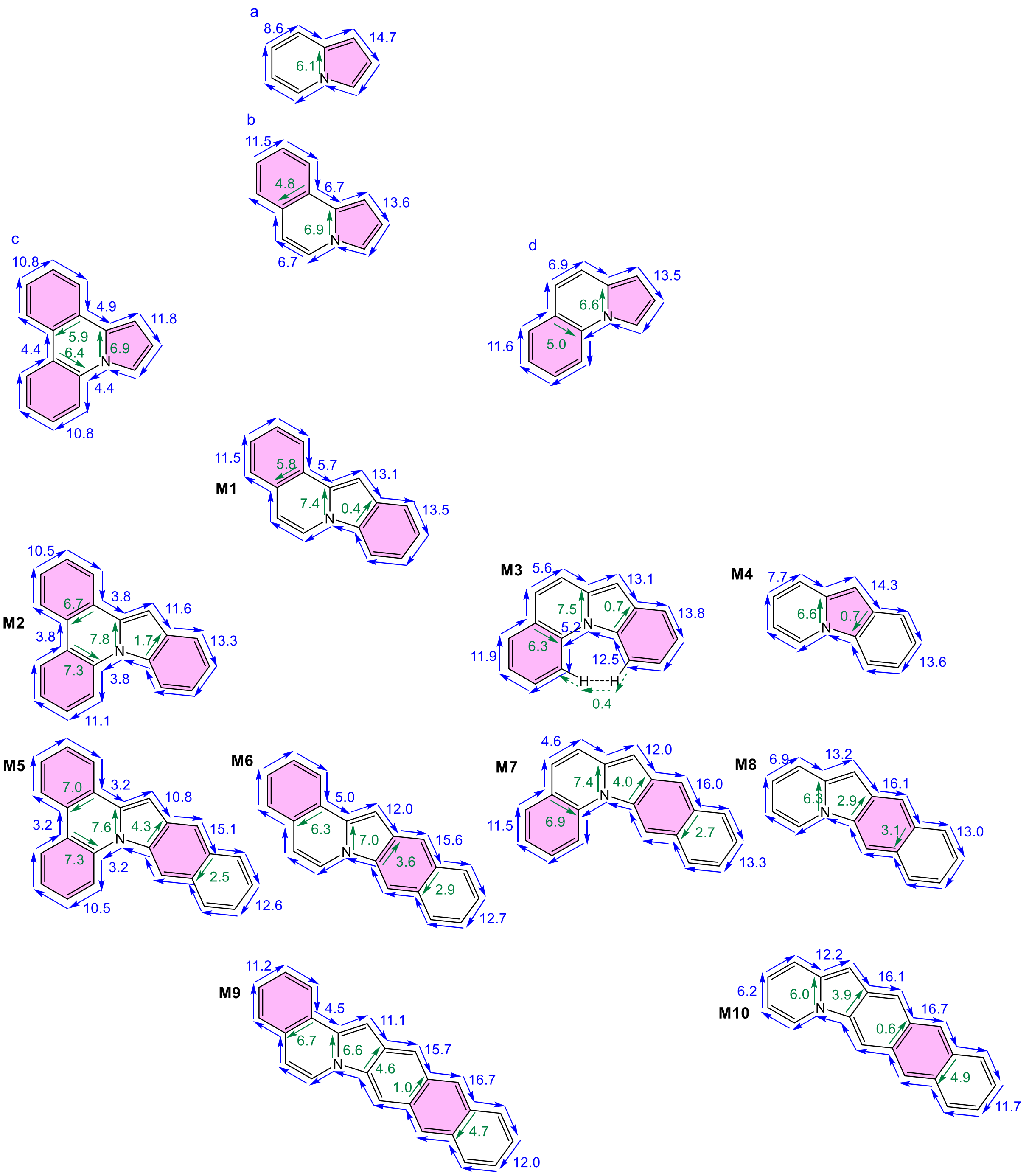

Figure S 64. The current density values for of π-expanded indoloindolizines.

Details of aromatic stabilization energy calculations

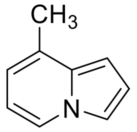	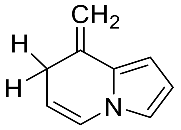
Structure-1 (A)	
Difference of energy = -20.674 kcal/mol	
Energy: -403.2724747 (B3LYP/def2-TZVPP)<br><br>Sum of electronic and zero-point Energies= -403.115815	Energy: -403.2391032<br><br>Sum of electronic and zero-point Energies= -403.082869
Frequency: 143.6127	Frequency: 74.6381
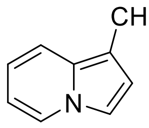	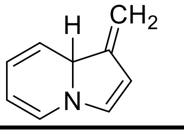
Structure-1 (B)	
Difference of energy = -37.579 kcal/mol	
Energy: -403.2701164 (B3LYP/def2-TZVPP)<br><br>Sum of electronic and zero-point Energies= -403.113724	Energy: -403.2093326 (B3LYP/def2-TZVPP)<br><br>Sum of electronic and zero-point Energies= -403.053838
Frequency: 43.1758	Frequency: 100.6927
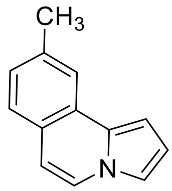	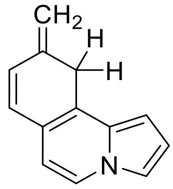
Structure-2 (A)	
Difference of energy = -26.951 kcal/mol	
Energy: =-556.9748381 (B3LYP/def2-TZVPP)<br><br>Sum of electronic and zero-point Energies= -556.771522	Energy: -556.9316992 (B3LYP/def2-TZVPP)<br><br>Sum of electronic and zero-point Energies= -556.728873
Frequency: 59.5978	Frequency: 55.0259
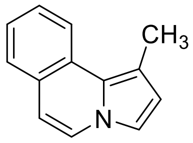	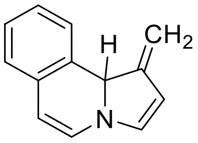
Structure-2 (B)	
Difference of energy = -32.978 kcal/mol	
Energy: =-556.972503 (B3LYP/def2-TZVPP)<br><br>Sum of electronic and zero-point Energies= -556.768574	Energy: -556.9190591 (B3LYP/def2-TZVPP)<br><br>Sum of electronic and zero-point Energies= -556.716020
Frequency: 82.4239	Frequency: 72.707
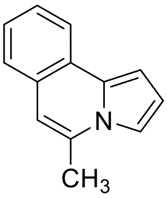	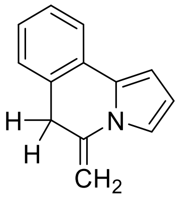
Structure-2 (C)	
Difference of energy = -15.806 kcal/mol	

Energy: -556.9775723 (B3LYP/def2-TZVPP)<br><br>Sum of electronic and zero-point Energies= -556.773714	Energy: -556.9522;569 (B3LYP/def2-TZVPP)<br><br>Sum of electronic and zero-point Energies= -556.748526
Frequency: 100.8152	Frequency: 80.661
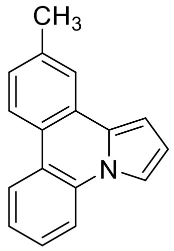	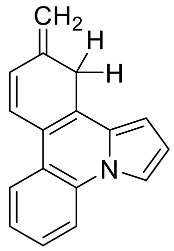
Structure-4c (A)	
Difference of energy = -27.315 kcal/mol	
Energy: -710.6762575 (B3LYP/def2-TZVPP)<br><br>Sum of electronic and zero-point Energies= -710.426024	Energy: -710.6323372 (B3LYP/def2-TZVPP)<br><br>Sum of electronic and zero-point Energies= -710.382494
Frequency: 55.7849	Frequency: 49.0475
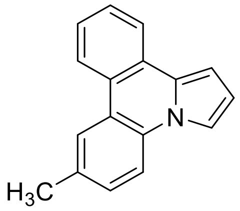	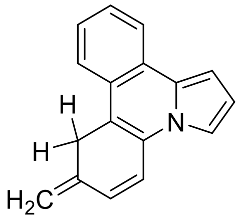
Structure-4c (B)	
Difference of energy = -28.539 kcal/mol	
Energy: -710.6756624 (B3LYP/def2-TZVPP)<br><br>Sum of electronic and zero-point Energies= -710.425434	Energy: -710.629786 (B3LYP/def2-TZVPP)<br><br>Sum of electronic and zero-point Energies= -710.379954
Frequency: 48.6122	Frequency: 40.7353
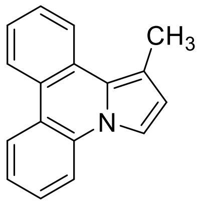	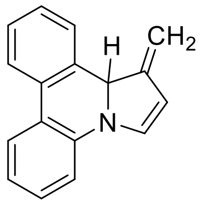
Structure-4c (C)	
Difference of energy = -29.718 kcal/mol	
Energy: -710.6696111 (B3LYP/def2-TZVPP)<br><br>Sum of electronic and zero-point Energies= -710.419495	Energy: -710.6253525 (B3LYP/def2-TZVPP)<br><br>Sum of electronic and zero-point Energies= -710.375409
Frequency: 53.779	Frequency: 69.8589
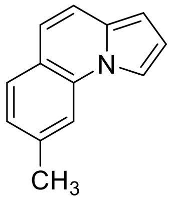	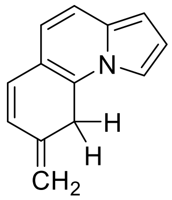
Structure-2a (A)	
Difference of energy = -27.273 kcal/mol	

Energy: -556.975474 (B3LYP/def2-TZVPP)<br><br>Sum of electronic and zero-point Energies= -556.772221	Energy: -556.9314789 (B3LYP/def2-TZVPP)<br><br>Sum of electronic and zero-point Energies= -556.728759
Frequency: 49.6465	Frequency: 19.2749
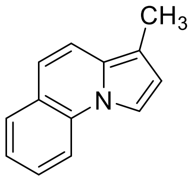	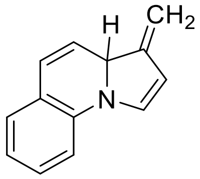
Structure-2a (B)	
Difference of energy = -34.406 kcal/mol	
Energy: -556.97463 (B3LYP/def2-TZVPP)<br><br>Sum of electronic and zero-point Energies= -556.771311	Energy: -556.9191061 (B3LYP/def2-TZVPP)<br><br>Sum of electronic and zero-point Energies= -556.716482
Frequency: 36.6623	Frequency: 66.4044
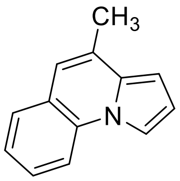	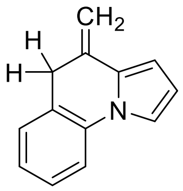
Structure-2a (C)	
Difference of energy = -17.214 kcal/mol	
Energy: -556.9769014 (B3LYP/def2-TZVPP)<br><br>Sum of electronic and zero-point Energies= -556.773275	Energy: -556.9494792 (B3LYP/def2-TZVPP)<br><br>Sum of electronic and zero-point Energies= -556.745842
Frequency: 100.8152	Frequency: 76.1206
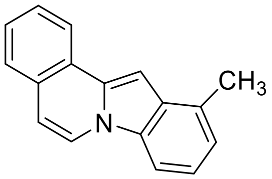	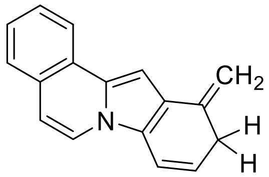
Structure-4 (A)	
Difference of energy = -27.826 kcal/mol	
Energy: -710.6765752 (B3LYP/def2-TZVPP)<br><br>Sum of electronic and zero-point Energies= -710.426171	Energy: -710.6315293 (B3LYP/def2-TZVPP)<br><br>Sum of electronic and zero-point Energies= -710.381828
Frequency: 65.2086	Frequency: 58.298
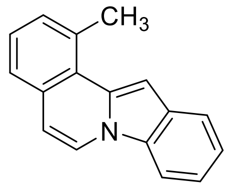	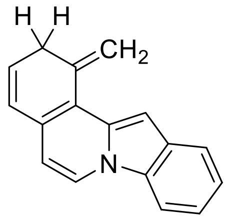
Structure-4 (B)	
Difference of energy = -28.699 kcal/mol	
Energy: -710.6715175 (B3LYP/def2-TZVPP)<br><br>Sum of electronic and zero-point Energies= -710.420455	Energy: -710.6246427 (B3LYP/def2-TZVPP)<br><br>Sum of electronic and zero-point Energies= -710.374720
Frequency: 56.9516	Frequency: 62.3149

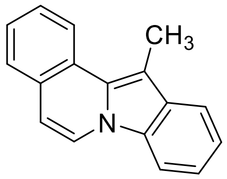	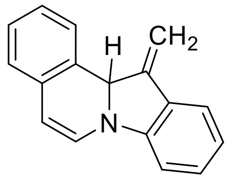
Structure-4 (C)	
Difference of energy = -27.659 kcal/mol	
Energy: =-710.6762575 (B3LYP/def2-TZVPP)<br><br>Sum of electronic and zero-point Energies= -710.426024	Energy: -710.6282806 (B3LYP/def2-TZVPP)<br><br>Sum of electronic and zero-point Energies= -710.378044
Frequency: 53.4875	Frequency: 36.7808
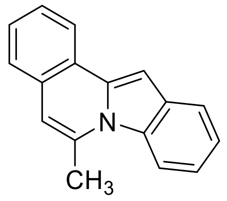	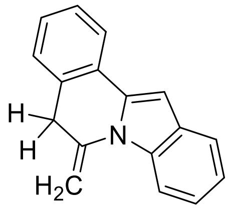
Structure-4 (D)	
Difference of energy = -12.289 kcal/mol	
Energy: -710.6732558 (B3LYP/def2-TZVPP)<br><br>Sum of electronic and zero-point Energies= -710.422164	Energy: -710.6534482 (B3LYP/def2-TZVPP)<br><br>Sum of electronic and zero-point Energies= -710.402581
Frequency: 56.7966	Frequency: 61.2979
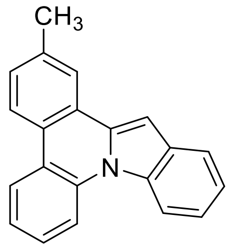	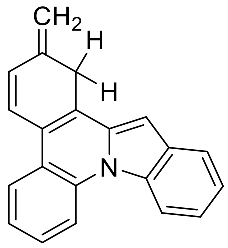
Structure-6c (A)	
Difference of energy = -27.5 kcal/mol	
Energy: -864.3715513 (B3LYP/def2-TZVPP)<br><br>Sum of electronic and zero-point Energies= - 864.074149	Energy: -864.3273968 (B3LYP/def2-TZVPP),<br><br>Sum of electronic and zero-point Energies= -864.030401
Frequency: 48.6049	Frequency: 44.0705
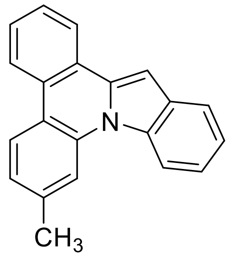	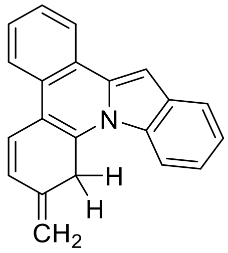
Structure-6c (B)	
Difference of energy = -28.21 kcal/mol	
Energy: -826.1838584 (B3LYP/def2-TZVPP)<br><br>Sum of electronic and zero-point Energies= -825.895014	Energy: -826.1477904 (B3LYP/def2-TZVPP)<br><br>Sum of electronic and zero-point Energies= -825.858442
Frequency: 29.4294	Frequency: 44.9067

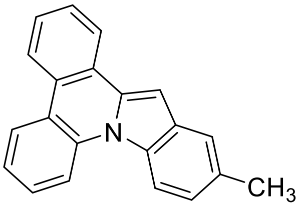	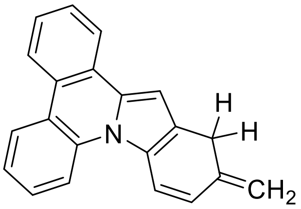
Structure-6c (C)	
Difference of energy = -26.7 kcal/mol	
Energy: -864.3707237 (B3LYP/def2-TZVPP)<br><br>Sum of electronic and zero-point Energies= - 864.073304	Energy: -864.3276751 (B3LYP/def2-TZVPP)<br><br>Sum of electronic and zero-point Energies= -864.030686
Frequency: 46.3801	Frequency: 41.224
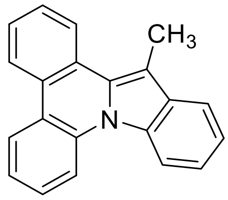	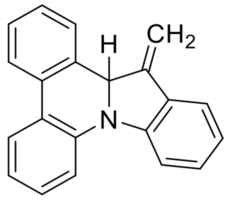
Structure-6c (D)	
Difference of energy = -21.215 kcal/mol	
Energy: -864.3670781 (B3LYP/def2-TZVPP), -542398.1208 Kcal/mol<br><br>Sum of electronic and zero-point Energies= -864.068867	Energy: -864.33251 (B3LYP/def2-TZVPP), -542376.429 Kcal/mol<br><br>Sum of electronic and zero-point Energies= -864.035059
Frequency: 34.3911	Frequency: 34.6179
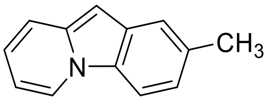	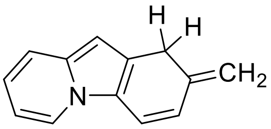
Structure-3 (A)	
Difference of energy = -24.459 kcal/mol	
Energy: -556.970224 (B3LYP/def2-TZVPP)<br><br>Sum of electronic and zero-point Energies= -556.767001	Energy: -556.9308918 (B3LYP/def2-TZVPP)<br><br>Sum of electronic and zero-point Energies= -556.728023
Frequency: 72.8885	Frequency: 46.595
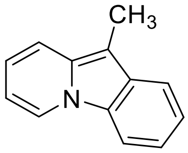	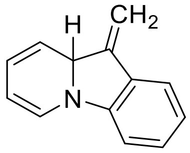
Structure-3 (B)	
Difference of energy = -32.282 kcal/mol	
Energy: -556.9702553 (B3LYP/def2-TZVPP),<br><br>Sum of electronic and zero-point Energies= -556.766838	Energy: -556.9180749 (B3LYP/def2-TZVPP)<br><br>Sum of electronic and zero-point Energies= -556.715394
Frequency: 76.2822	Frequency: 63.705
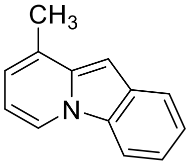	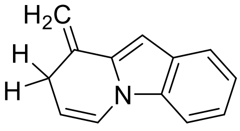
Structure-3 (C)	

Difference of energy = -19.161 kcal/mol	
Energy: -556.9726009 (B3LYP/def2-TZVPP), -349505.3198 Kcal/mol<br><br>Sum of electronic and zero-point Energies= -556.769029	Energy: -556.941741 (B3LYP/def2-TZVPP)<br><br>Sum of electronic and zero-point Energies= -556.738494
Frequency: 95.0024	Frequency: 71.2883
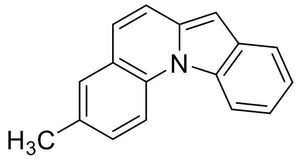	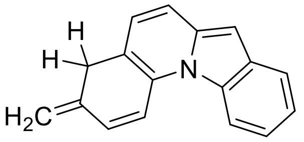
Structure-4a (A)	
Difference of energy = -30.402 kcal/mol	
Energy: -710.6706368 (B3LYP/def2-TZVPP)<br><br>Sum of electronic and zero-point Energies= -710.420345	Energy: -710.6215934 (B3LYP/def2-TZVPP)<br><br>Sum of electronic and zero-point Energies= -710.371896
Frequency: 43.6417	Frequency: 47.5301
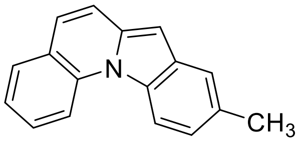	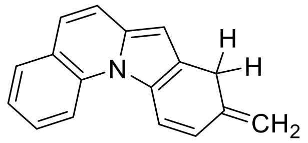
Structure-4a (B)	
Difference of energy = -26.236 kcal/mol	
Energy: -710.6703763 (B3LYP/def2-TZVPP)<br><br>Sum of electronic and zero-point Energies= -710.420045	Energy: -710.6282043 (B3LYP/def2-TZVPP)<br><br>Sum of electronic and zero-point Energies= -710.378236
Frequency: 44.3476	Frequency: 62.3149
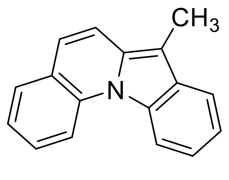	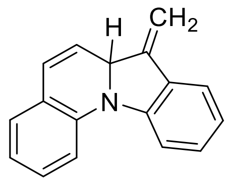
Structure-4a (C)	
Difference of energy = -27.98 kcal/mol	
Energy: -710.6706118 (B3LYP/def2-TZVPP)<br><br>Sum of electronic and zero-point Energies= -710.419998	Energy: -710.6253525 (B3LYP/def2-TZVPP), -445923.8043 Kcal/mol<br><br>Sum of electronic and zero-point Energies= -710.375409
Frequency: 46.8111	Frequency: 41.4387
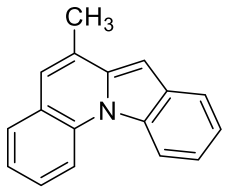	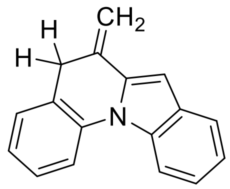
Structure-4a (D)	
Difference of energy = -14.91 kcal/mol	
Energy: -710.672384 (B3LYP/def2-TZVPP)<br><br>Sum of electronic and zero-point Energies= -710.421629	Energy: -710.6486755 (B3LYP/def2-TZVPP)<br><br>Sum of electronic and zero-point Energies= -710.397868
Frequency: 48.8972	Frequency: 67.7431

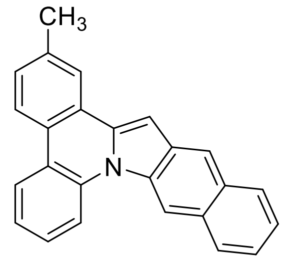	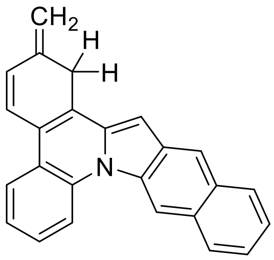
Structure-8c (A)	
Difference of energy = -27.578 kcal/mol	
Energy: -1018.0650638 (B3LYP/def2-TZVPP)<br>Sum of electronic and zero-point Energies= -1017.721179	Energy: -1018.0207063 (B3LYP/def2-TZVPP)<br>Sum of electronic and zero-point Energies= -1017.677231
Frequency: 34.6756	Frequency: 32.3956
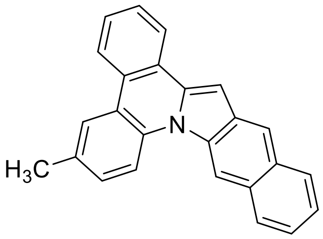	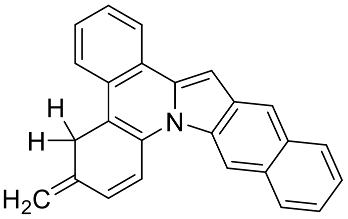
Structure-8c (B)	
Difference of energy = -29.554 kcal/mol	
Energy: -1018.0644359 (B3LYP/def2-TZVPP)<br>Sum of electronic and zero-point Energies= -1017.720560	Energy: -1018.0168456 (B3LYP/def2-TZVPP)<br>Sum of electronic and zero-point Energies= -1017.673463
Frequency: 35.1854	Frequency: 39.4194
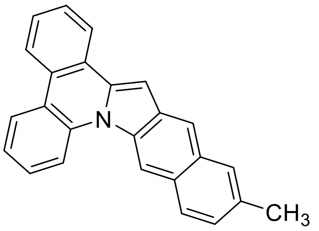	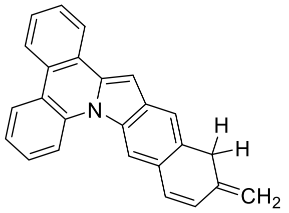
Structure-8c (C)	
Difference of energy = -20.781 kcal/mol	
Energy: -1018.0649748 (B3LYP/def2-TZVPP)<br>Sum of electronic and zero-point Energies= -1017.720990	Energy: -1018.0317293 (B3LYP/def2-TZVPP)<br>Sum of electronic and zero-point Energies= -1017.687874
Frequency: 34.2816	Frequency: 32.8885
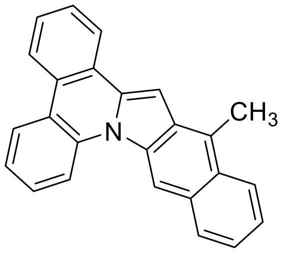	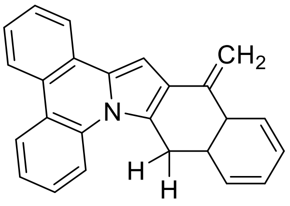
Structure-8c (D)	
Difference of energy = -16.9 kcal/mol	
Energy= -1018.0623376 (B3LYP/def2-TZVPP)	Energy= -1018.0355906 (B3LYP/def2-TZVPP)

Sum of electronic and zero-point Energies= -1017.718020	Sum of electronic and zero-point Energies= -<br>1017.691121
Frequency: 32.6199	Frequency: 15.783
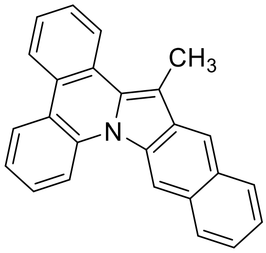	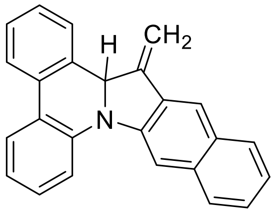
Structure-8c (E)	
Difference of energy = -18.918 kcal/mol	
Energy= -1018.0605807 (B3LYP/def2-TZVPP)<br><br>Sum of electronic and zero-point Energies= -1017.715889	Energy= -1018.0298832 (B3LYP/def2-TZVPP)<br><br>Sum of electronic and zero-point Energies= -1017.685741
Frequency: 31.5428	Frequency: 25.1821
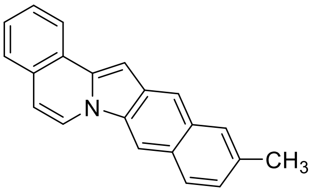	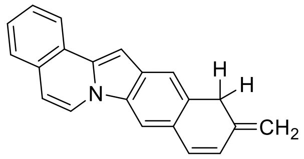
Structure-6 (A)	
Difference of energy = -20.68 kcal/mol	
Energy= -864.3692882 (B3LYP/def2-TZVPP)<br><br>Sum of electronic and zero-point Energies= -864.072545	Energy= -864.3362423 (B3LYP/def2-TZVPP)<br><br>Sum of electronic and zero-point Energies= -864.039589
Frequency: 38.1845	Frequency: 36.3196
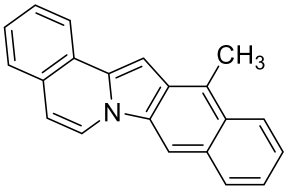	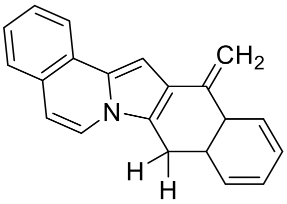
Structure-6 (B)	
Difference of energy = -16.3 kcal/mol	
Energy= -864.3667869 (B3LYP/def2-TZVPP)<br><br>Sum of electronic and zero-point Energies= -864.069741	Energy= -864.3408203 (B3LYP/def2-TZVPP)<br><br>Sum of electronic and zero-point Energies= - 864.043680
Frequency: 41.6346	Frequency: 1.0391
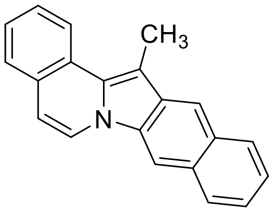	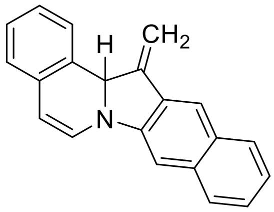
Structure-6 (C)	
Difference of energy = -25.141 kcal/mol	
Energy= -864.3660238 (B3LYP/def2-TZVPP)<br><br>Sum of electronic and zero-point Energies= -864.068733	Energy= -864.3256115 (B3LYP/def2-TZVPP)<br><br>Sum of electronic and zero-point Energies= -864.028669

Frequency: 39.0328	Frequency: 26.9436
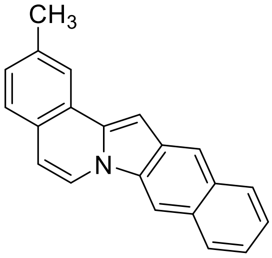	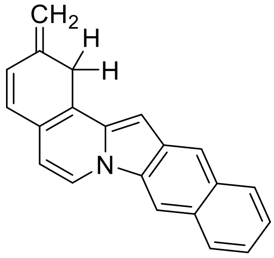
Structure-6 (D)	
Difference of energy = -27.7 kcal/mol	
Energy= -864.3691722 (B3LYP/def2-TZVPP)<br><br>Sum of electronic and zero-point Energies= -864.072521	Energy = -864.3244497 (B3LYP/def2-TZVPP)<br><br>Sum of electronic and zero-point Energies= - 864.028336
Frequency: 39.2977	Frequency: 33.5202
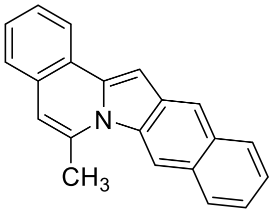	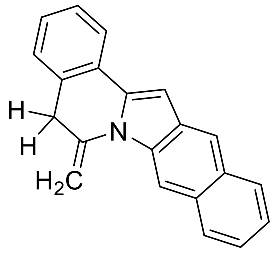
Structure-6 (E)	
Difference of energy = -11.674 kcal/mol	
Energy= -864.3663138 (B3LYP/def2-TZVPP)<br><br>Sum of electronic and zero-point Energies= -864.068739	Energy = -864.3474293 (B3LYP/def2-TZVPP)<br><br>Sum of electronic and zero-point Energies= -864.050045
Frequency: 38.0728	Frequency: 41.3759
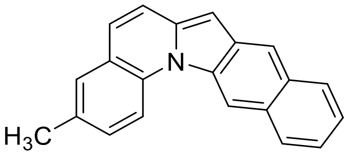	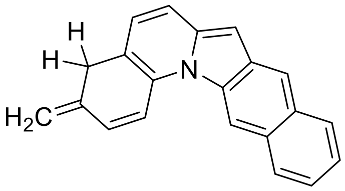
Structure-6a (A)	
Difference of energy = -30.7 kcal/mol	
Energy= -864.3638386 (B3LYP/def2-TZVPP)<br><br>Sum of electronic and zero-point Energies= - 864.067058	Energy = - 864.3147027 (B3LYP/def2-TZVPP)<br><br>Sum of electronic and zero-point Energies= - 864.018488
Frequency: 37.2506	Frequency: 47.4332
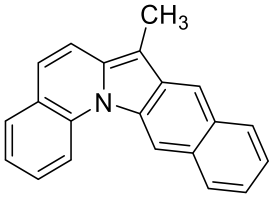	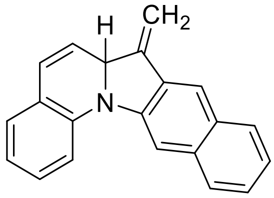
Structure-6a (B)	
Difference of energy = -25.7 kcal/mol	
Energy= -864.3640869 (B3LYP/def2-TZVPP)<br><br>Sum of electronic and zero-point Energies= - 864.066951	Energy = -864.3225487 (B3LYP/def2-TZVPP)<br><br>Sum of electronic and zero-point Energies= -864.025903

Frequency: 39.2056	Frequency: 33.331
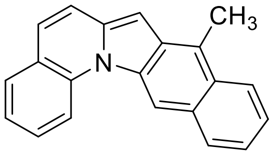	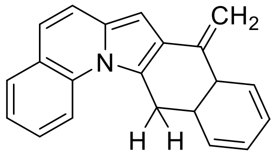
Structure-6a (C)	
Difference of energy = -16.2 kcal/mol	
Energy= -864.3616993 (B3LYP/def2-TZVPP)<br><br>Sum of electronic and zero-point Energies= -864.064447	Energy = -864.3361466 (B3LYP/def2-TZVPP)<br><br>Sum of electronic and zero-point Energies= - 864.038695
Frequency: 37.0021	Frequency: 15.7135
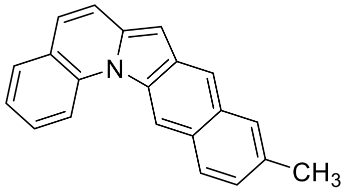	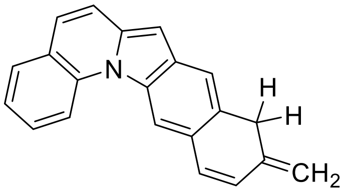
Structure-6a (D)	
Difference of energy = -20.6 kcal/mol	
Energy= -864.3643447 (B3LYP/def2-TZVPP)<br><br>Sum of electronic and zero-point Energies= -864.067433	Energy = -864.331445 (B3LYP/def2-TZVPP)<br><br>Sum of electronic and zero-point Energies= - 864.034643
Frequency: 37.2644	Frequency: 39.2056
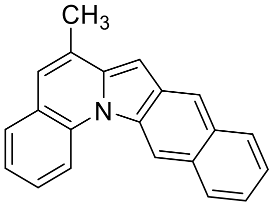	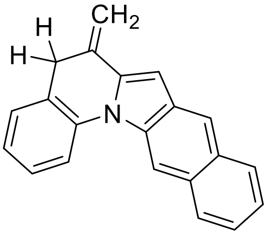
Structure-6a (E)	
Difference of energy = -14.6 kcal/mol	
Energy= -864.3656724 (B3LYP/def2-TZVPP)<br><br>Sum of electronic and zero-point Energies= - 864.068418	Energy = -864.3424615 (B3LYP/def2-TZVPP)<br><br>Sum of electronic and zero-point Energies= -864.045157
Frequency: 37.9917	Frequency: 52.7085
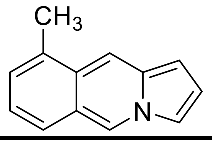	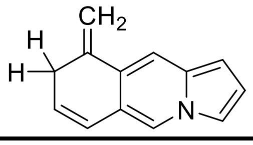
Structure-2b (A)	
Difference of energy = -18.687 kcal/mol	
Energy= -556.9569003 (B3LYP/def2-TZVPP)<br><br>Sum of electronic and zero-point Energies= -556.753718	Energy = -556.9268451 (B3LYP/def2-TZVPP)<br><br>Sum of electronic and zero-point Energies= -556.723939
Frequency: 104.3734	Frequency: 87.6267
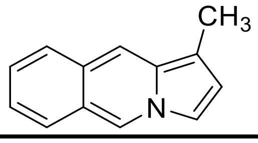	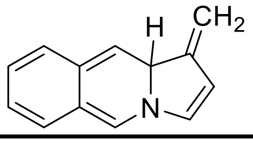
Structure-2b (B)	

Difference of energy = -41.444 kcal/mol	
Energy= -556.9566692 (B3LYP/def2-TZVPP)<br><br>Sum of electronic and zero-point Energies= -556.753858	Energy = -556.8897025 (B3LYP/def2-TZVPP)<br><br>Sum of electronic and zero-point Energies= -556.687813
Frequency: 75.5176	Frequency: 74.3257
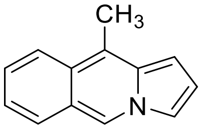	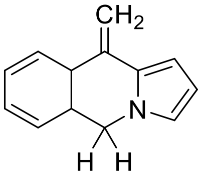
Structure-2b (C)	
Difference of energy = -5.7 kcal/mol	
Energy= -556.9554084 (B3LYP/def2-TZVPP)<br><br>Sum of electronic and zero-point Energies= -556.752437	Energy = -556.9473841 (B3LYP/def2-TZVPP)<br><br>Sum of electronic and zero-point Energies= - 556.743404
Frequency: 54.9472	Frequency: 52.4888
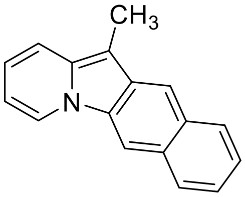	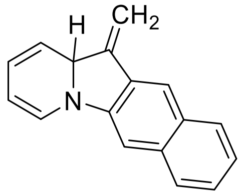
Structure-5 (A)	
Difference of energy = -29.674 kcal/mol	
Energy= -710.6630229 (B3LYP/def2-TZVPP)<br><br>Sum of electronic and zero-point Energies= -710.413087	Energy = -710.6151821 (B3LYP/def2-TZVPP)<br><br>Sum of electronic and zero-point Energies= -710.365799
Frequency: 67.2283	Frequency: 48.8235
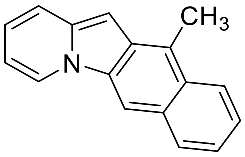	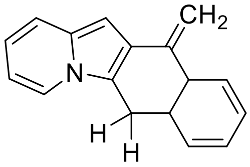
Structure-5 (B)	
Difference of energy = -15.1 kcal/mol	
Energy= -710.6610465 (B3LYP/def2-TZVPP)<br><br>Sum of electronic and zero-point Energies= -710.410990	Energy = -710.6371688 (B3LYP/def2-TZVPP)<br><br>Sum of electronic and zero-point Energies= - 710.386946
Frequency: 65.7526	Frequency: 12.3523
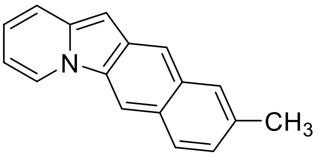	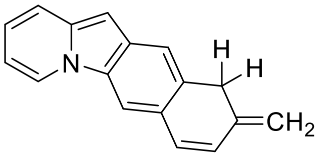
Structure-5 (C)	
Difference of energy = -8.3164 kcal/mol	
Energy= -710.6636329 (B3LYP/def2-TZVPP)<br><br>Sum of electronic and zero-point Energies= -710.413861	Energy = -710.6314096 (B3LYP/def2-TZVPP)<br><br>Sum of electronic and zero-point Energies= -710.381708
Frequency: 53.3532	Frequency: 48.1267

<table>
<tr><td>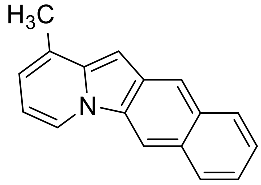</td><td>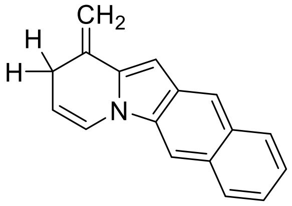</td></tr>
<tr><td>Structure-5 (D)</td><td></td></tr>
<tr><td colspan="2">Difference of energy = -18.521 kcal/mol</td></tr>
<tr><td>Energy= -710.6652651 (B3LYP/def2-TZVPP)

Sum of electronic and zero-point Energies= -710.415201</td><td>Energy = -710.635457 (B3LYP/def2-TZVPP)

Sum of electronic and zero-point Energies= -710.385686</td></tr>
<tr><td>Frequency: 60.0627</td><td>Frequency: 59.062</td></tr>
<tr><td>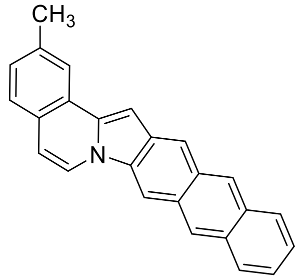</td><td>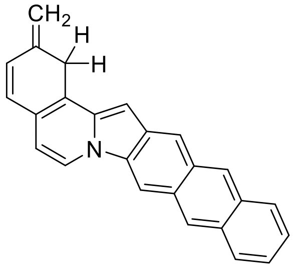</td></tr>
<tr><td>Structure-8 (A)</td><td></td></tr>
<tr><td colspan="2">Difference of energy = -27.823 kcal/mol</td></tr>
<tr><td>Energy= -1018.0583879 (B3LYP/def2-TZVPP)

Sum of electronic and zero-point Energies= -1017.715351</td><td>Energy = -1018.0134951 (B3LYP/def2-TZVPP)

Sum of electronic and zero-point Energies= -1017.671012</td></tr>
<tr><td>Frequency: 28.3207</td><td>Frequency: 25.1099</td></tr>
<tr><td>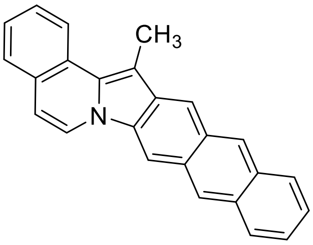</td><td>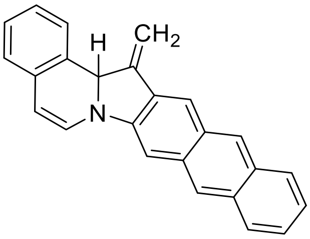</td></tr>
<tr><td>Structure-8 (B)</td><td></td></tr>
<tr><td colspan="2">Difference of energy = -23.822 kcal/mol</td></tr>
<tr><td>Energy= -1018.0552111 (B3LYP/def2-TZVPP)

Sum of electronic and zero-point Energies= -1017.711534</td><td>Energy = -1018.0170399 (B3LYP/def2-TZVPP)

Sum of electronic and zero-point Energies= -1017.673571</td></tr>
<tr><td>Frequency: 29.48</td><td>Frequency: 22.0711</td></tr>
<tr><td>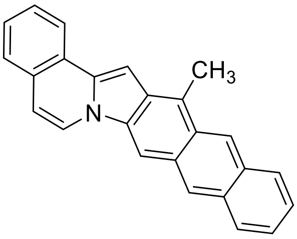</td><td>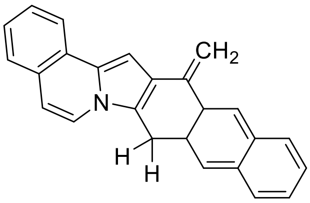</td></tr>
<tr><td>Structure-8 (C)</td><td></td></tr>
<tr><td colspan="2">Difference of energy = -12.9 kcal/mol</td></tr>
<tr><td>Energy= -1018.056383 (B3LYP/def2-TZVPP)

Sum of electronic and zero-point Energies= -1017.712899</td><td>Energy = -1018.0361595 (B3LYP/def2-TZVPP)</td></tr>
</table>

	Sum of electronic and zero-point Energies= - 1017.692320
Frequency: 29.8124	Frequency: 15.0652
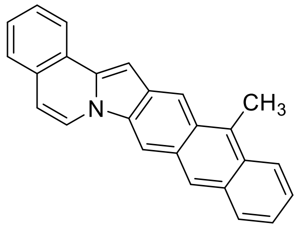	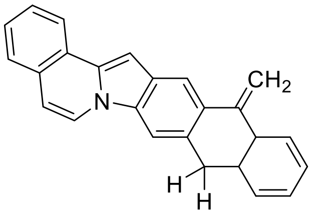
Structure-8 (D)	
Difference of energy = -8.6 kcal/mol	
Energy= -1018.0519111 (B3LYP/def2-TZVPP)<br><br>Sum of electronic and zero-point Energies= -1017.708319	Energy = -1018.0386888 (B3LYP/def2-TZVPP)<br><br>Sum of electronic and zero-point Energies= -1017.694756
Frequency: 28.4886	Frequency: 27.6391
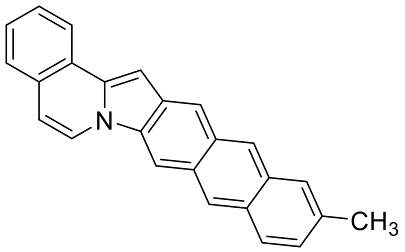	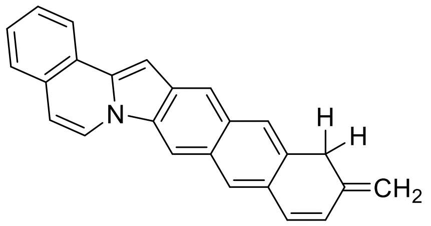
Structure-8 (E)	
Difference of energy = -17.775 kcal/mol	
Energy= -1018.0588201 (B3LYP/def2-TZVPP)<br><br>Sum of electronic and zero-point Energies= -1017.715657	Energy = -1018.0305137 (B3LYP/def2-TZVPP)<br><br>Sum of electronic and zero-point Energies= -1017.687331
Frequency: 27.6981	Frequency: 27.2581
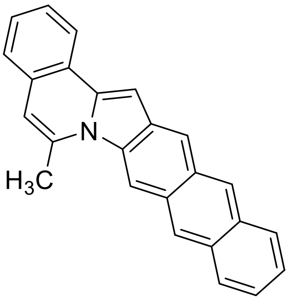	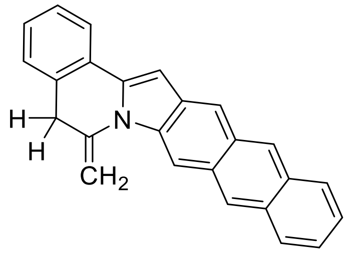
Structure-8 (F)	
Difference of energy = -11.467 kcal/mol	
Energy= -1018.0555084 (B3LYP/def2-TZVPP)<br><br>Sum of electronic and zero-point Energies= -1017.711537	Energy = -1018.0370636 (B3LYP/def2-TZVPP)<br><br>Sum of electronic and zero-point Energies= -1017.693263
Frequency: 28.3036	Frequency: 29.9411
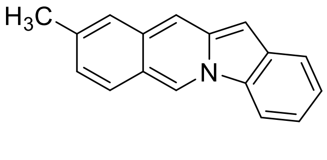	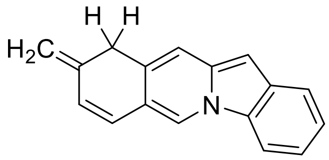
Structure-4b (A)	
Difference of energy = -15.848 kcal/mol	

Energy= -710.6572502 (B3LYP/def2-TZVPP)<br><br>Sum of electronic and zero-point Energies= -710.407632	Energy = -710.6321822 (B3LYP/def2-TZVPP)<br><br>Sum of electronic and zero-point Energies= -710.382377
Frequency: 52.3671	Frequency: 47.6768
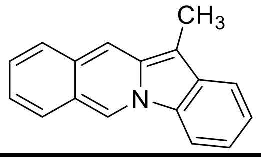	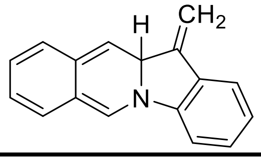
Structure-4b (B)	
Difference of energy = -35.69 kcal/mol	
Energy= -710.6554073 (B3LYP/def2-TZVPP)<br><br>Sum of electronic and zero-point Energies= -710.405781	Energy = -710.5980775 (B3LYP/def2-TZVPP)<br><br>Sum of electronic and zero-point Energies= -710.349064
Frequency: 45.196	Frequency: 48.3231
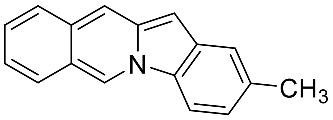	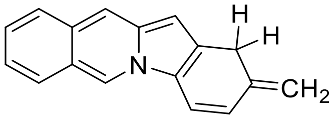
Structure-4b (C)	
Difference of energy = -22.952 kcal/mol	
Energy= -710.6558642 (B3LYP/def2-TZVPP)<br><br>Sum of electronic and zero-point Energies= -710.406360	Energy = -710.6189531 (B3LYP/def2-TZVPP)<br><br>Sum of electronic and zero-point Energies= -710.369784
Frequency: 53.5383	Frequency: 39.8193
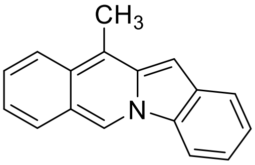	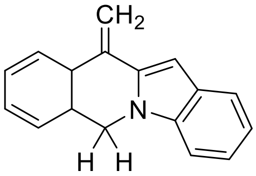
Structure-4b (D)	
Difference of energy = -3.6 kcal/mol	
Energy= -710.6543303 (B3LYP/def2-TZVPP)<br><br>Sum of electronic and zero-point Energies= -710.404534	Energy = -710.6495758 (B3LYP/def2-TZVPP)<br><br>Sum of electronic and zero-point Energies= - 710.398720
Frequency: 39.4773	Frequency: 39.5865
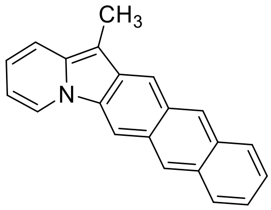	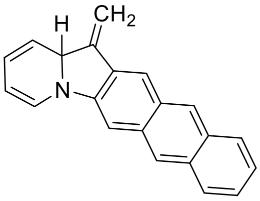
Structure-7 (A)	
Difference of energy = -28.32 kcal/mol	
Energy= -826.1862076 (B3LYP/def2-TZVPP)<br><br>Sum of electronic and zero-point Energies= -864.055725	Energy = -864.3065037 (B3LYP/def2-TZVPP)<br><br>Sum of electronic and zero-point Energies= -864.010594
Frequency: 44.3608	Frequency: 38.2681

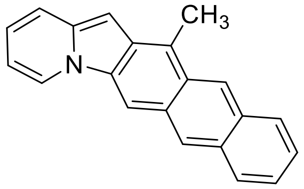	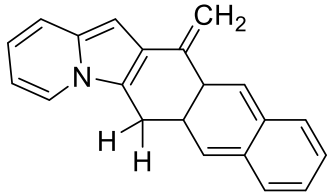
Structure-7 (B)	
Difference of energy = -11.5 kcal/mol	
Energy= -864.332536 (B3LYP/def2-TZVPP)<br><br>Sum of electronic and zero-point Energies= -864.053887	Energy = -865.4751997 (B3LYP/def2-TZVPP)<br><br>Sum of electronic and zero-point Energies= - 864.035623
Frequency: 46.3801	Frequency: 32.4073
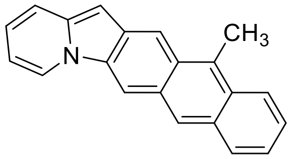	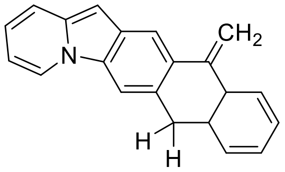
Structure-7 (C)	
Difference of energy = -8.08 kcal/mol	
Energy= -864.3459836 (B3LYP/def2-TZVPP)<br><br>Sum of electronic and zero-point Energies= -864.049356	Energy = -864.3334578 (B3LYP/def2-TZVPP)<br><br>Sum of electronic and zero-point Energies= - 864.036476
Frequency: 39.6632	Frequency: 36.4944
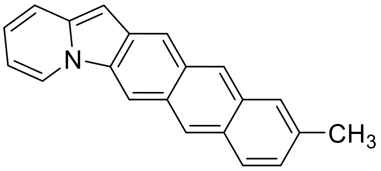	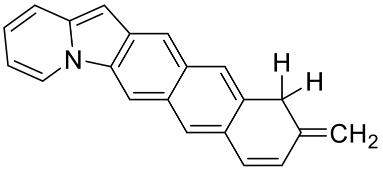
Structure-7 (D)	
Difference of energy = -17.5 kcal/mol	
Energy= -864.3528677 (B3LYP/def2-TZVPP)<br><br>Sum of electronic and zero-point Energies= -864.056677	Energy = -864.3249466 (B3LYP/def2-TZVPP)<br><br>Sum of electronic and zero-point Energies= -864.028721
Frequency: 37.2079	Frequency: 35.9
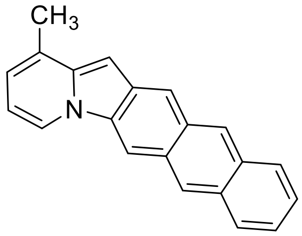	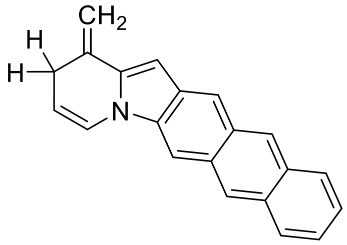
Structure-7 (E)	
Difference of energy = -18.217 kcal/mol	
Energy= -864.354259 (B3LYP/def2-TZVPP)<br><br>Sum of electronic and zero-point Energies= -864.057803	Energy = -864.324958 (B3LYP/def2-TZVPP)<br><br>Sum of electronic and zero-point Energies= -864.028772
Frequency: 40.4441	Frequency: 40.1376
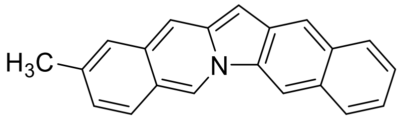	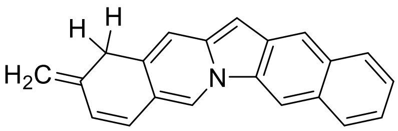

Structure-6b (A)	
Difference of energy = -15.54 kcal/mol	
Energy= -864.349858 (B3LYP/def2-TZVPP)<br><br>Sum of electronic and zero-point Energies= -864.053764	Energy = -864.3252848 (B3LYP/def2-TZVPP)<br><br>Sum of electronic and zero-point Energies= -864.029001
Frequency: 38.58	Frequency: 36.9465
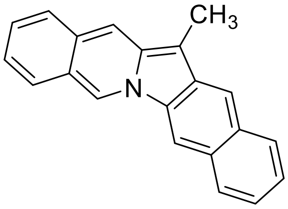	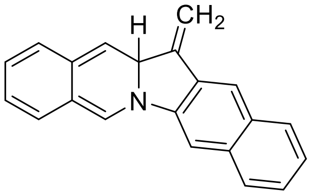
Structure-6b (B)	
Difference of energy = -32.951 kcal/mol	
Energy= -864.3480242 (B3LYP/def2-TZVPP)<br><br>Sum of electronic and zero-point Energies= -864.051879	Energy = -864.2950499 (B3LYP/def2-TZVPP)<br><br>Sum of electronic and zero-point Energies= -863.999368
Frequency: 46.8898	Frequency: 35.2513
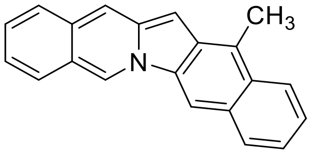	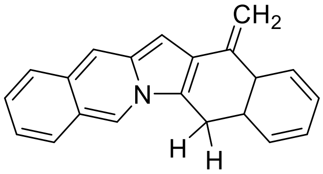
Structure-6b (C)	
Difference of energy = -14.2 kcal/mol	
Energy= -864.3462514 (B3LYP/def2-TZVPP)<br><br>Sum of electronic and zero-point Energies= -864.049951	Energy = -865. 3237945 (B3LYP/def2-TZVPP)<br><br>Sum of electronic and zero-point Energies= - 864.027296
Frequency: 46.1778	Frequency: 15.9412
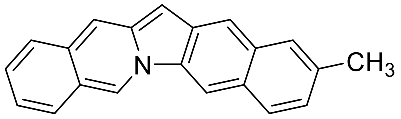	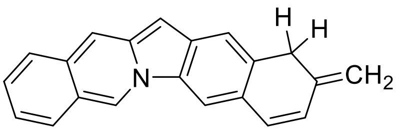
Structure-6b (D)	
Difference of energy = -19.781 kcal/mol	
Energy= -864.3489772 (B3LYP/def2-TZVPP)<br><br>Sum of electronic and zero-point Energies= -864.052941	Energy = -864.3173786 (B3LYP/def2-TZVPP)<br><br>Sum of electronic and zero-point Energies= -864.021418
Frequency: 39.0126	Frequency: 37.49
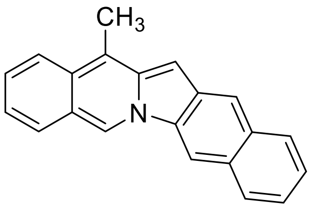	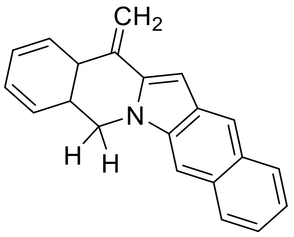
Structure-6b (E)	
Difference of energy = -3.1 kcal/mol	
Energy= -864.3468676 (B3LYP/def2-TZVPP)	Energy = -864.3429247 (B3LYP/def2-TZVPP)

Sum of electronic and zero-point Energies= - 864.050564	Sum of electronic and zero-point Energies= - 864.045605
Frequency: 41.496	Frequency: 33.1222

<table>
<tr><td>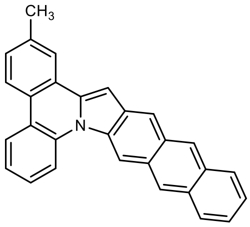
</td><td>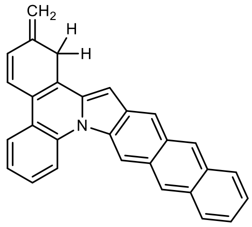
</td></tr>
<tr><td>Structure-m11 (A)
Sum of electronic and zero-point Energies=-1171.364200</td><td>Sum of electronic and zero-point Energies=-1171.320191</td></tr>
<tr><td colspan="2">Energy difference= -27.61 kcal/mol</td></tr>
<tr><td>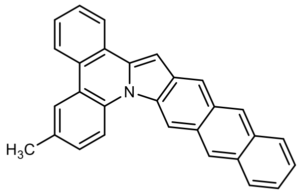
</td><td>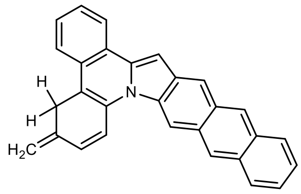
</td></tr>
<tr><td>Structure-m11 (B)
Sum of electronic and zero-point Energies=-1171.363551</td><td>Sum of electronic and zero-point Energies=-1171.316267</td></tr>
<tr><td colspan="2">Energy difference= -29.67 kcal/mol</td></tr>
<tr><td>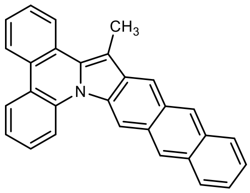
</td><td>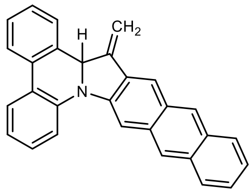
</td></tr>
<tr><td>Structure-m11 (C)
Sum of electronic and zero-point Energies=-1171.358917</td><td>Sum of electronic and zero-point Energies=-1171.330652</td></tr>
<tr><td colspan="2">Energy difference = -17.74 kcal/mol</td></tr>
<tr><td>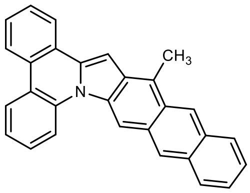
</td><td>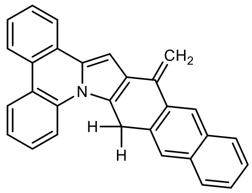
</td></tr>
<tr><td>Structure-m11 (D)
Sum of electronic and zero-point Energies=-1171.361353</td><td>Sum of electronic and zero-point Energies=-1171.339870</td></tr>
<tr><td colspan="2">Energy difference = -13.48 kcal/mol</td></tr>
<tr><td>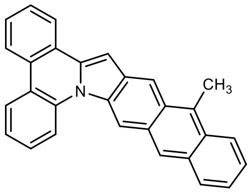
</td><td>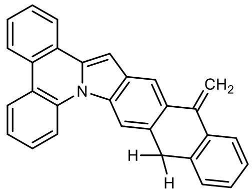
</td></tr>
<tr><td>Structure-m11 (E)
Sum of electronic and zero-point Energies=-1171.357196</td><td>Sum of electronic and zero-point Energies=-1171.343160</td></tr>
<tr><td colspan="2">Energy difference= -8.81 kcal/mol</td></tr>
</table>

<table>
<tr><td>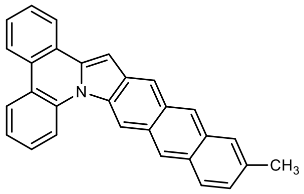
</td><td>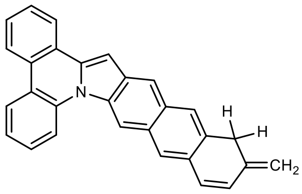
</td></tr>
<tr><td>Structure-m11 (F)
Sum of electronic and zero-point Energies=-1171.364278</td><td>Sum of electronic and zero-point Energies=-1171.335758</td></tr>
<tr><td colspan="2">Energy difference= -17.90 kcal/mol</td></tr>
<tr><td>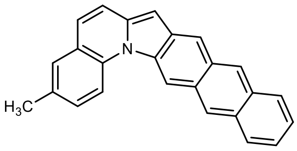
</td><td>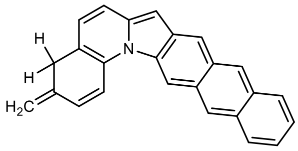
</td></tr>
<tr><td>Structure-m12 (A)
Sum of electronic and zero-point Energies=-1017.709935</td><td>Sum of electronic and zero-point Energies=-1017.660991</td></tr>
<tr><td colspan="2">Energy difference= -30.71 kcal/mol</td></tr>
<tr><td>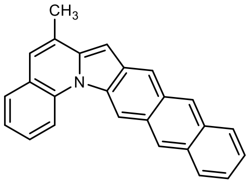
</td><td>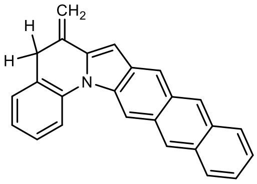
</td></tr>
<tr><td>Structure-m12 (B)
Sum of electronic and zero-point Energies=-1017.711324</td><td>Sum of electronic and zero-point Energies=-1017.688284</td></tr>
<tr><td colspan="2">Energy difference= -14.46 kcal/mol</td></tr>
<tr><td>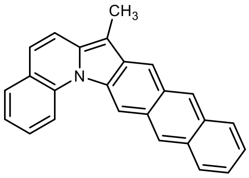
</td><td>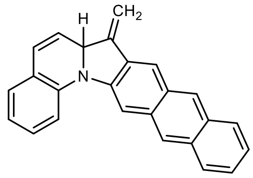
</td></tr>
<tr><td>Structure-m12 (C)
Sum of electronic and zero-point Energies=-1017.709925</td><td>Sum of electronic and zero-point Energies=-1017.670734</td></tr>
<tr><td colspan="2">Energy difference = -24.59 kcal/mol</td></tr>
<tr><td>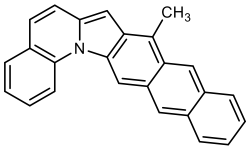
</td><td>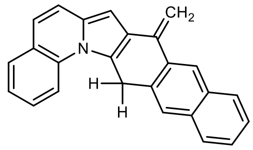
</td></tr>
<tr><td>Structure-m12 (D)
Sum of electronic and zero-point Energies=-1017.707662</td><td>Sum of electronic and zero-point Energies=-1017.687351</td></tr>
<tr><td colspan="2">Energy difference = -12.74 kcal/mol</td></tr>
<tr><td>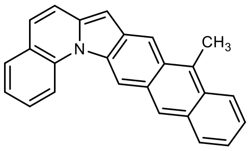
</td><td>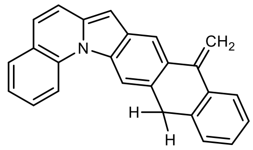
</td></tr>
<tr><td>Structure-m12 (E)
Sum of electronic and zero-point Energies=-1017.703520</td><td>Sum of electronic and zero-point Energies=-1017.689745</td></tr>
<tr><td colspan="2">Energy difference= -8.64 kcal/mol</td></tr>
<tr><td>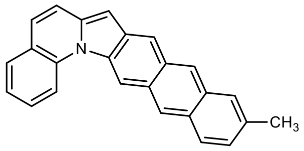
</td><td>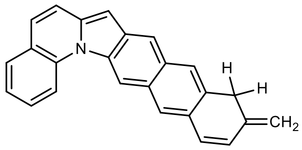
</td></tr>
</table>

Structure-m12 (F)<br>Sum of electronic and zero-point Energies=-1017.710587	Sum of electronic and zero-point Energies=-1017.682229
Energy difference= -17.79 kcal/mol	

Compounds with methyl groups on the beta-position to the nitrogen.

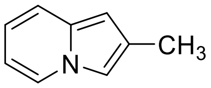	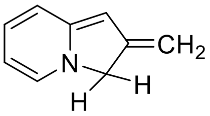
Structure-1 (B)	
Energy difference= -23.026 kcal/mol	
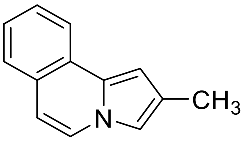	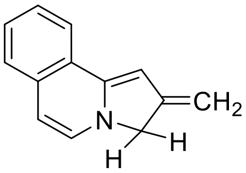
Structure-2 (B)	
Energy difference= -21.200 kcal/mol	
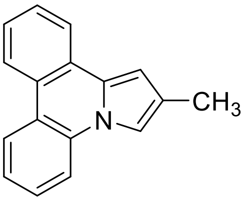	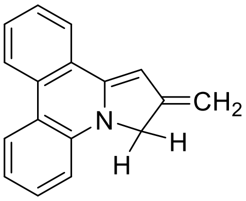
Structure-4c (C)	
Energy difference = -19.025 kcal/mol	
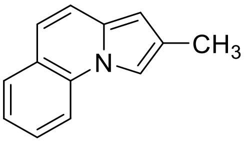	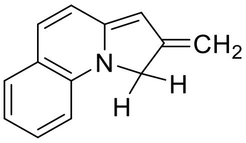
Structure-2a (B)	
Energy difference = -20.0157 kcal/mol	
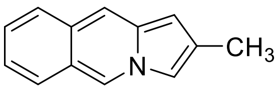	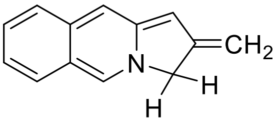
Structure-2b (B)	
Energy difference= -23.1080 kcal/mol	

S7. Organic Field Effect Transistor (OFET) device fabrication and characterization

S7.1 Equipment

OFET characteristics were recorded using A Keithley 2636A dual-channel source-meter instrument by Tektronix UK Ltd. (UK), operated with KickStart Software from the same company. It was connected to OFET Test Board for High Density OFETs provided by Ossila Ltd. (UK).

OFET devices were fabricated using Platinum OFET Test Chip (High Density) by Ossila (Ltd) (Figure S 65). One side of the chip, containing gate contacts, was covered with silver paste.

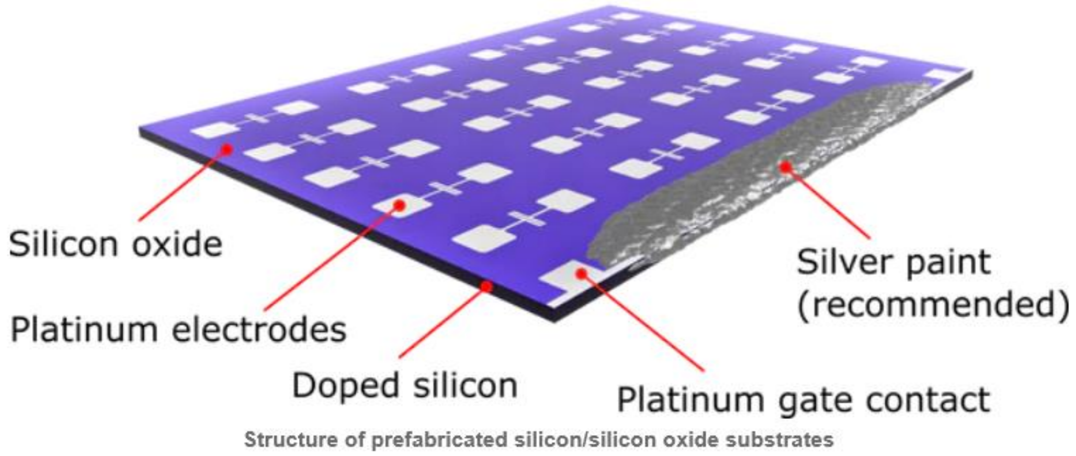

Figure S 65. Platinum OFET Test Chips adapted from Ossila.[15]

Parameters of the test chip were as follows:

Substrate/Gate	-	Silicon (p-doped)
Gate dielectric	-	300 nm thermally grown silicon dioxide
Source-Drain electrodes	-	Platinum (100 nm) / Titanium adhesion layer (5 nm)
Deposition method	-	Plasma sputtering
Patterning method	-	Photolithography
Channel width	-	1 mm
Channel lengths	-	2, 4, 6, 8, and 10 µm
No. of devices on plate	-	20

Electrodeposition of Au film was performed using SP-300 potentiostat/galvanostat of BioLogic company, controlled by EC-Lab software provided by the same manufacturer. ECF 60 Gold Plating Solution (10 g/L) from Melator® (Melator Technologies, UK) was used for that purpose.

S7.2. Procedures

S7.2.1 Electrode surface modification

To promote deposition of pentafluorothiophenol (FTP) monolayer, electrode surface was coated electrochemically with thin film of gold. For that purpose, gold was deposited on platinum electrodes using chronoamperometry in single droplet mode. That is, each Pt electrode of the FET devise (chip) served as working electrode (WE). Electrical contact was provided by touching contact area of FET device with Pt wire electrode. Coiled Pt electrode was immobilized parallel to FET chip surface on 3-5 mm height. It was used as a counter electrode (CE). Silver wire, wrapped with parafilm to prevent direct contact to counter electrode, was put in CE coil and served as a pseudoreference electrode (RE). To deposit Au film 50-100 µL droplet of gold plating solution was introduced into empty space between electrodes with automatic pipette. Then following potential program was applied. Firstly, 10 s of waiting allowed to start the program and to touch contact of WE with Pt wire. Subsequently, potential of –0.7 V vs. Ag/Ag^+ pseudo-reference was applied for 2 minutes. If no change in WE colour was observed, above potential program was repeated. Current recorded during gold plating is shown below on Figure S 66. This results in deposition of 100 nm thick Au film (estimation based on our previous experience - unpublished).

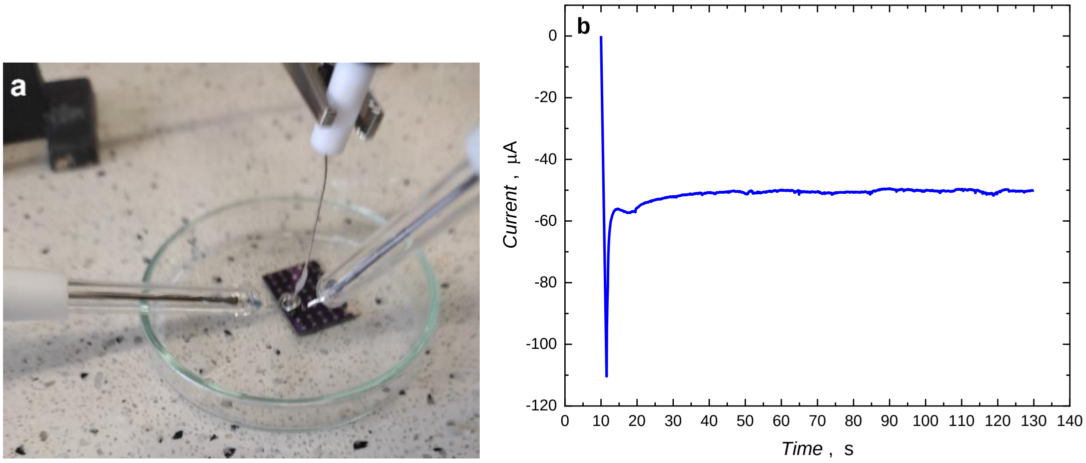

Figure S 66. Electrochemical deposition of gold film. (a) Photography of the electrochemical setup. (b) Current recorded during gold deposition from Melator® Gold Plating Solution by applying constant potential of -0.7 V vs. Ag/Ag^+ pseudo-reference.

After Au deposition chip was washed with deionized water twice and left to dry on air. Then, chip was immersed in 30 mM solution of FTP in anhydrous EtOH for 1h.[16, 17] Finaly, it was washed with EtOH three time and left to dry on air.

S7.2.2 Fabrication of the OFET devices

I6, **I8a**, **I9** were dissolved in anhydrous toluene under N_2 in glove box. Concentration of these solutions was 2 mg/mL. The solutions were drop-casted on each individual FET device in such a way to ensure grow of crystallites along channel width. For that purpose, Petri dish was tilted by 5-10°. Ossila test chip was put on the upper part of these Petri dishes. Lower part was filled with toluene. On each individual FET device the solution of organic compound (2 mg/mL) was drop-cast on the chip. Volume of drop was 0.25-0.5 µL. Petri dish was covered until solvent from dropped solutions evaporated. Then, drop-casting was repeated. After drying test chip was mounted in test board.

S7.2.3 Characterization of the OFET devices

To characterize OFET devices a constant gate voltage (V_{GS}) was applied and drain-source voltage (V_{DS}) was swept and series of measurements were performed at different values of V_{GS}. Value of drain-source current (I_{DS}) was recorded. The maximum possible voltage that could be applied as V_{GS} and V_{DS} was ±50 V.

S7.3. Scanning electron microscopy of the fabricated OFET devices

S7.3.1 Equipment and procedures

OFET samples prepared on the platinum OFET test chips and Au-coated OFET test chips were mounted in the SEM holder using conductive carbon tape. Electrical contact to the fabricated structures has been assured by using carbon tape link between the SEM support and OFET structures contact.

All SEM images and energy dispersive X-ray (EDX) spectra has been recorded using Nova NanoSEM microscope of FEI company (Hillsboro, OR, USA). For all images secondary electron mode has been used. In the low- resolution mode low-energy electron detector, an Everhart–Thornley detector (ETD) has been used, while, in the and high-resolution mode a through-the-lens (TLD) detector was employed. All images were taken using 3 kV beam. For EDX measurements 15 kV beam has been used to obtain higher intensity spectra.

S7.3.2 Results

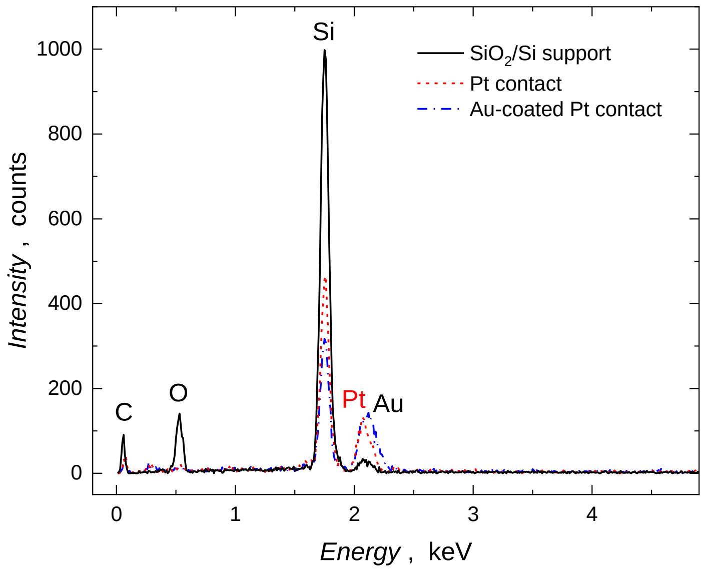

Figure S 67. EDX spectra recorded at different regions of the sample.

SEM images and EDX spectra of the as prepared Pt OFET test chip, Au-coated Pt OFET test chip and gate region with deposited indolizine derivatives are shown in Figure S 67 and Figure S 68.

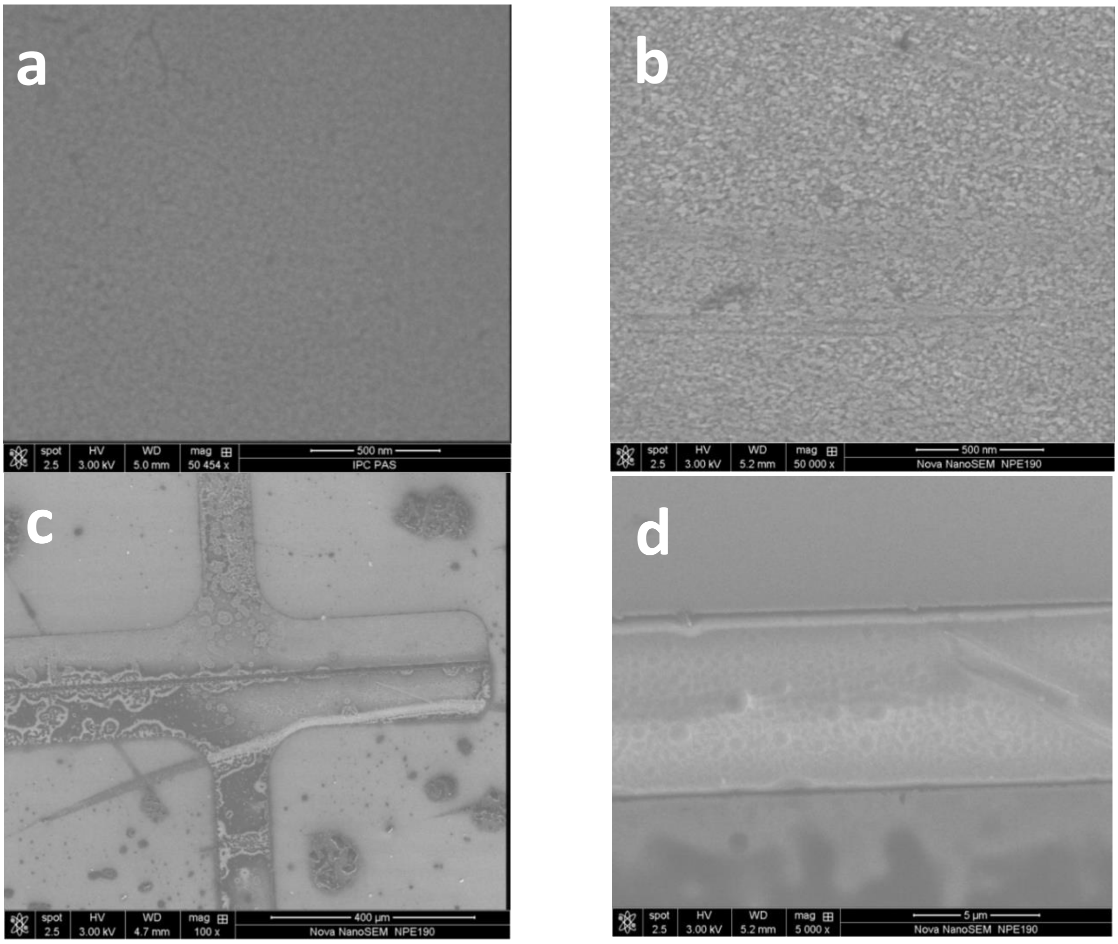

Figure S 68. Scanning electron microscopy images of (**a**) surface of Pt contact of the transistor structure, (**b**) surface of Pt contact of the transistor structure electrochemically coated by the gold layer, (**c**) overview of the transistor structure channel, and (**d**) channel filled by **I8a** organic material.

7.4. Mobility of the charge carriers calculations

The charge carrier mobility has been calculated from the Figure 7d and 7f showing dependence of the $I_{DS}^{1/2}$ of the studied compounds on the applied gate voltage. Taking into account ambipolar characteristic of the studied indolizine derivatives of both holes and electrons can be calculated from the linear parts of the curves depicted in the abovementioned figures. The slope of the fitted line is related with the charge carrier mobility by following equation:

$$\mu = \frac{2LA^2}{WC_i}$$

Where A is the slope of respective $I_{DS}^{1/2}$ vs. V_G curves, W and L are channel width and length, respectively, while C_i is dielectric capacitance per unit area. The calculated values of mobility are shown in the Table S 4.

Table S 4. Electron and hole mobilities calculated for the three studied indoloindolizine compounds

**Compound**	**Hole mobility, cm^2 V^{-1} s^{-1} at V_g = –30 V**	**Electron mobility, cm^2 V^{-1} s^{-1} at V_g =10 V**
I6	0.49	0.11
I8a	0.21	0.29
I9	0.26	0.11

S8. ^{1}H and ^{13}C NMR Spectra

IQ1

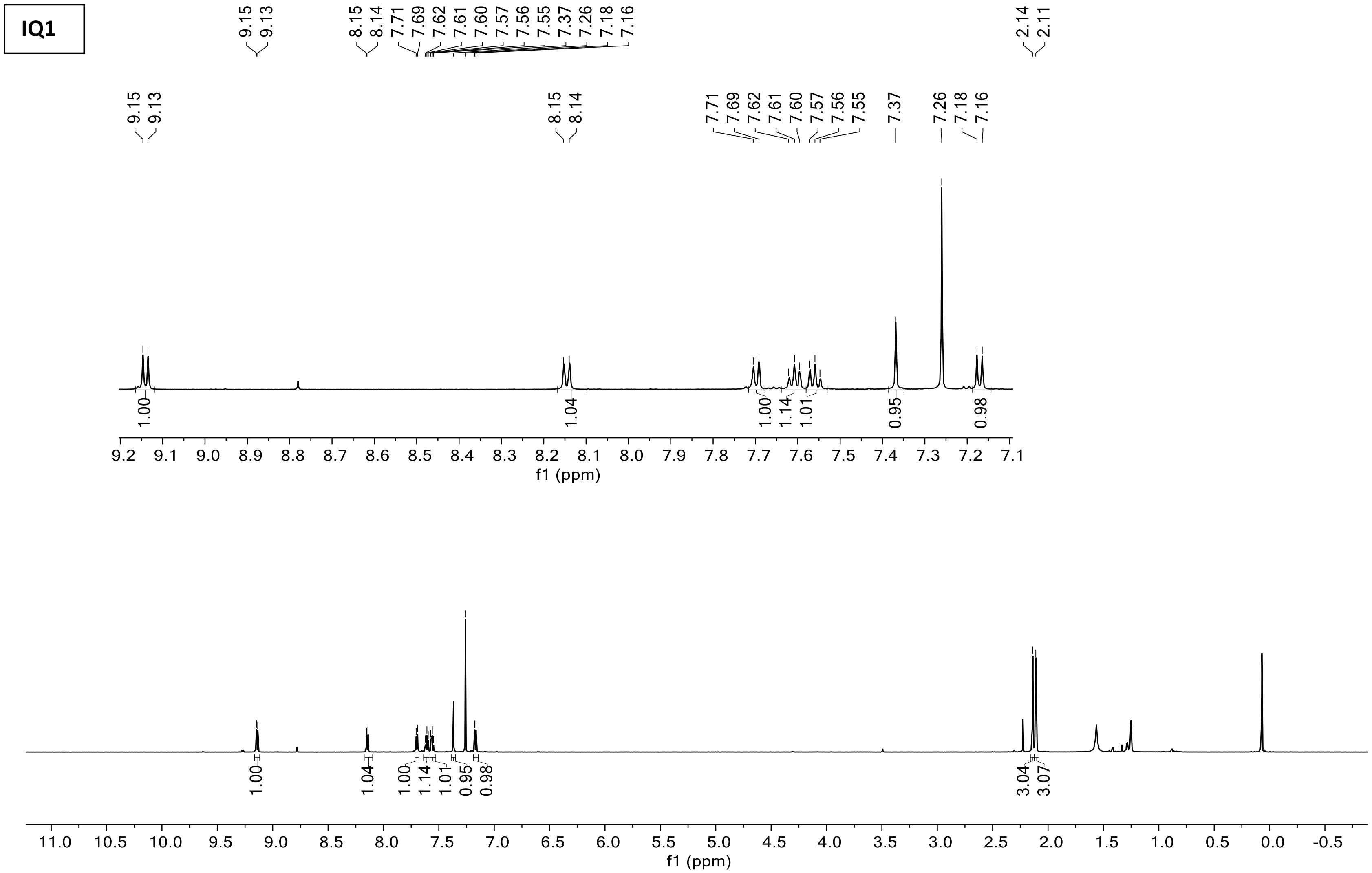

Figure S 69. ^{1}H NMR (top) spectra of **IQ1** in $CDCl_3$.

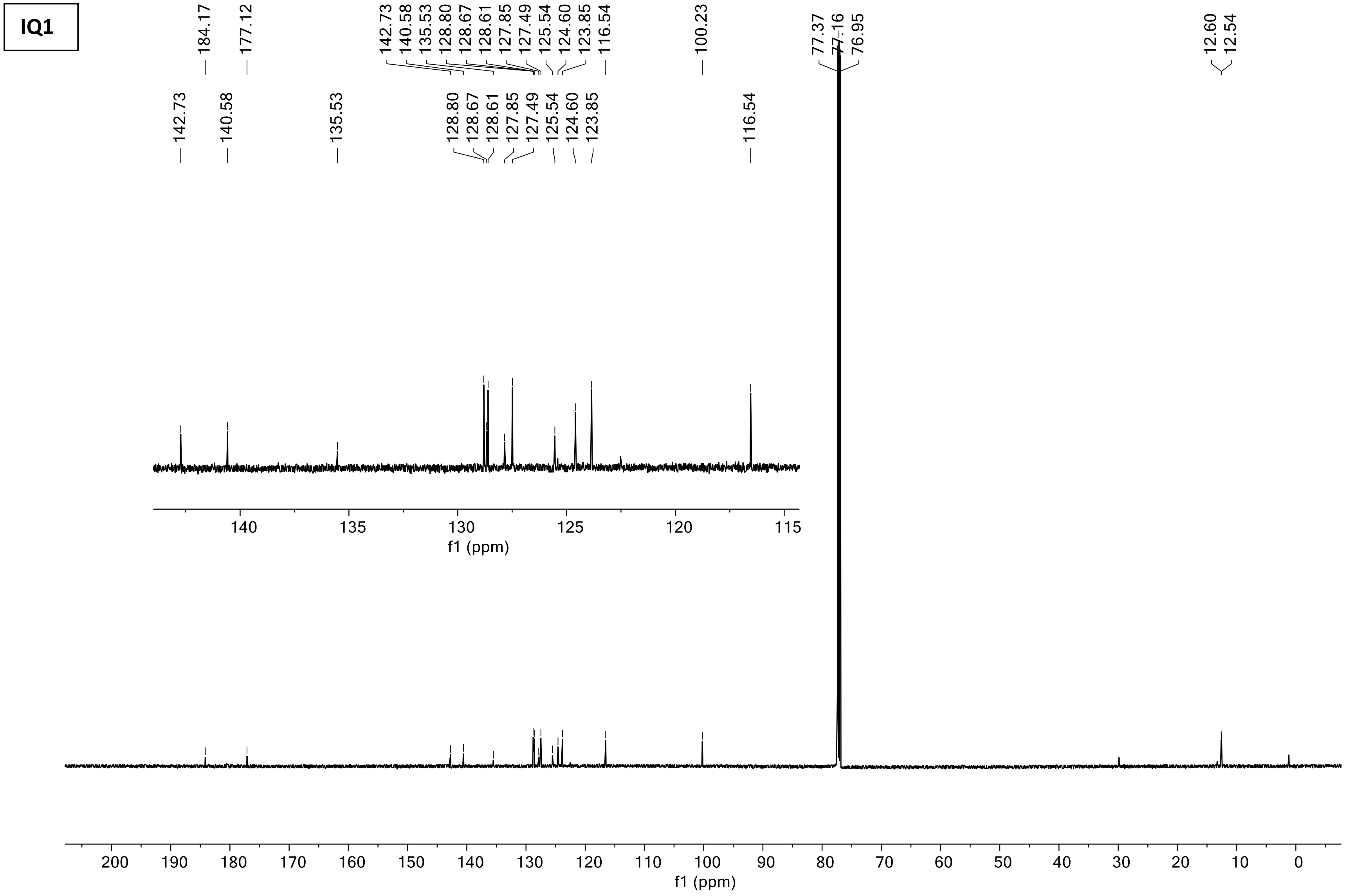

Figure S 70. ^{13}C NMR spectra of **IQ1** in $CDCl_3$.

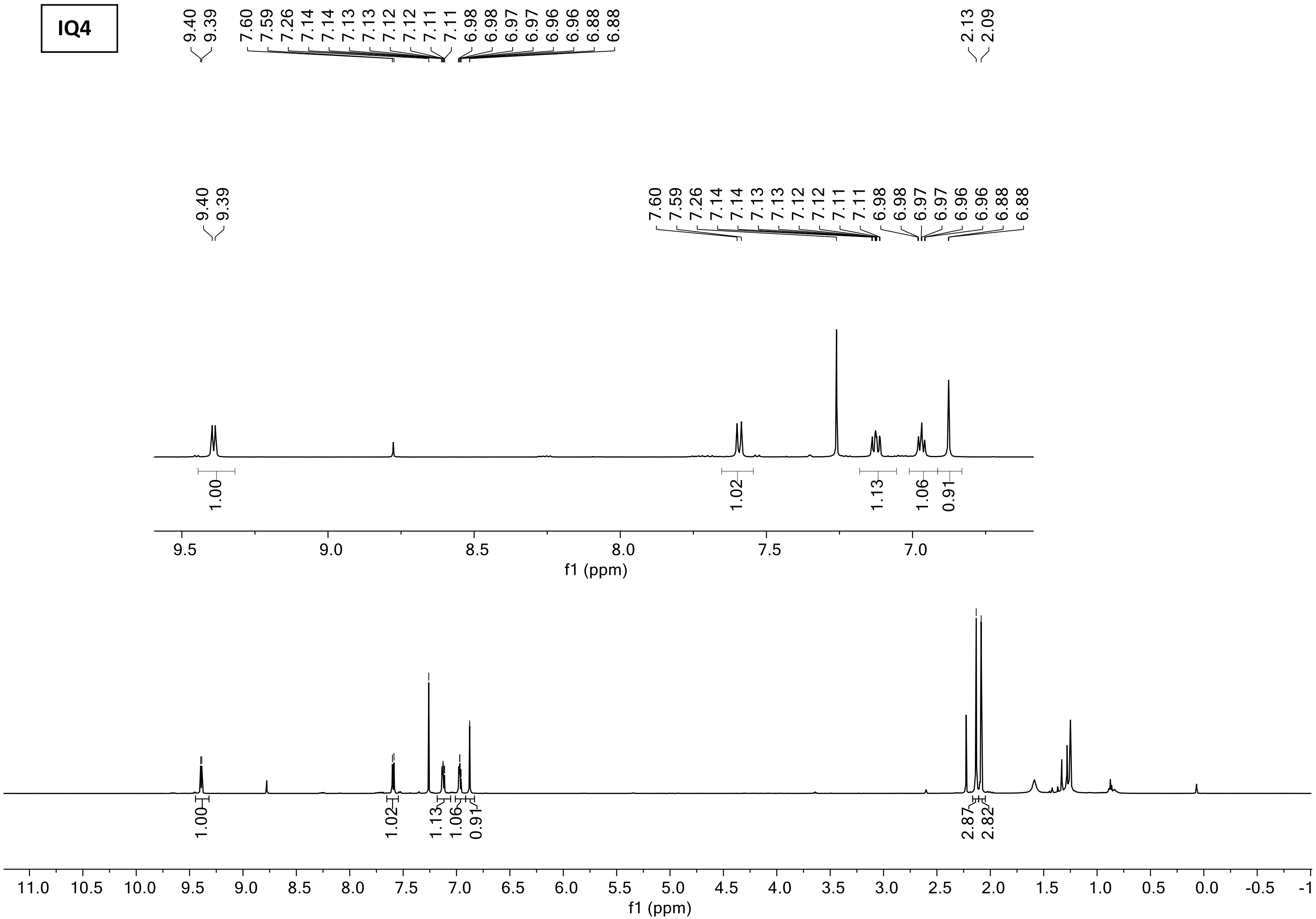

Figure S 71. ^{1}H NMR (top) spectra of **IQ4** in $CDCl_3$.

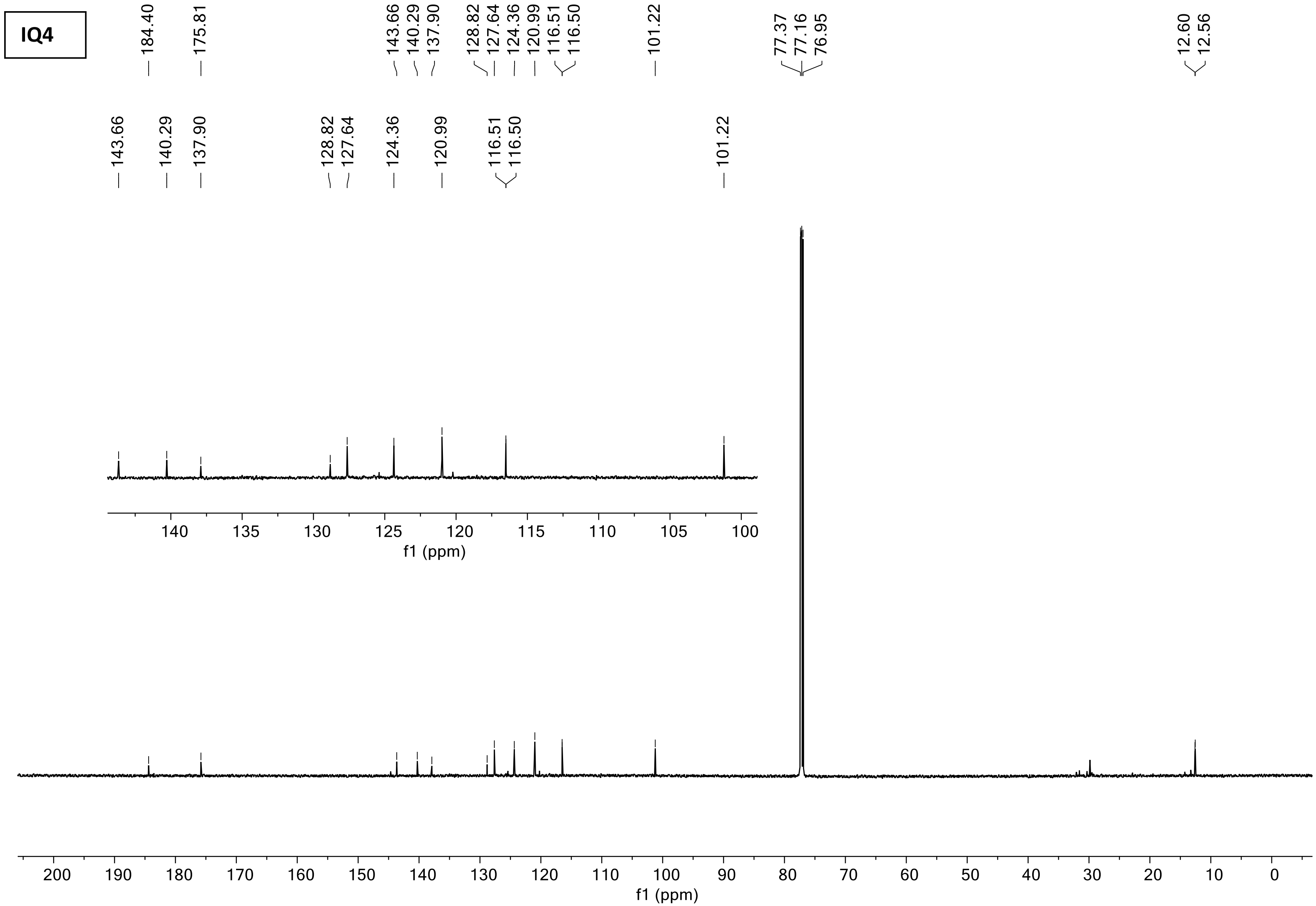

Figure S 72. ^{13}C NMR spectra of **IQ4** in $CDCl_3$.

IQ5

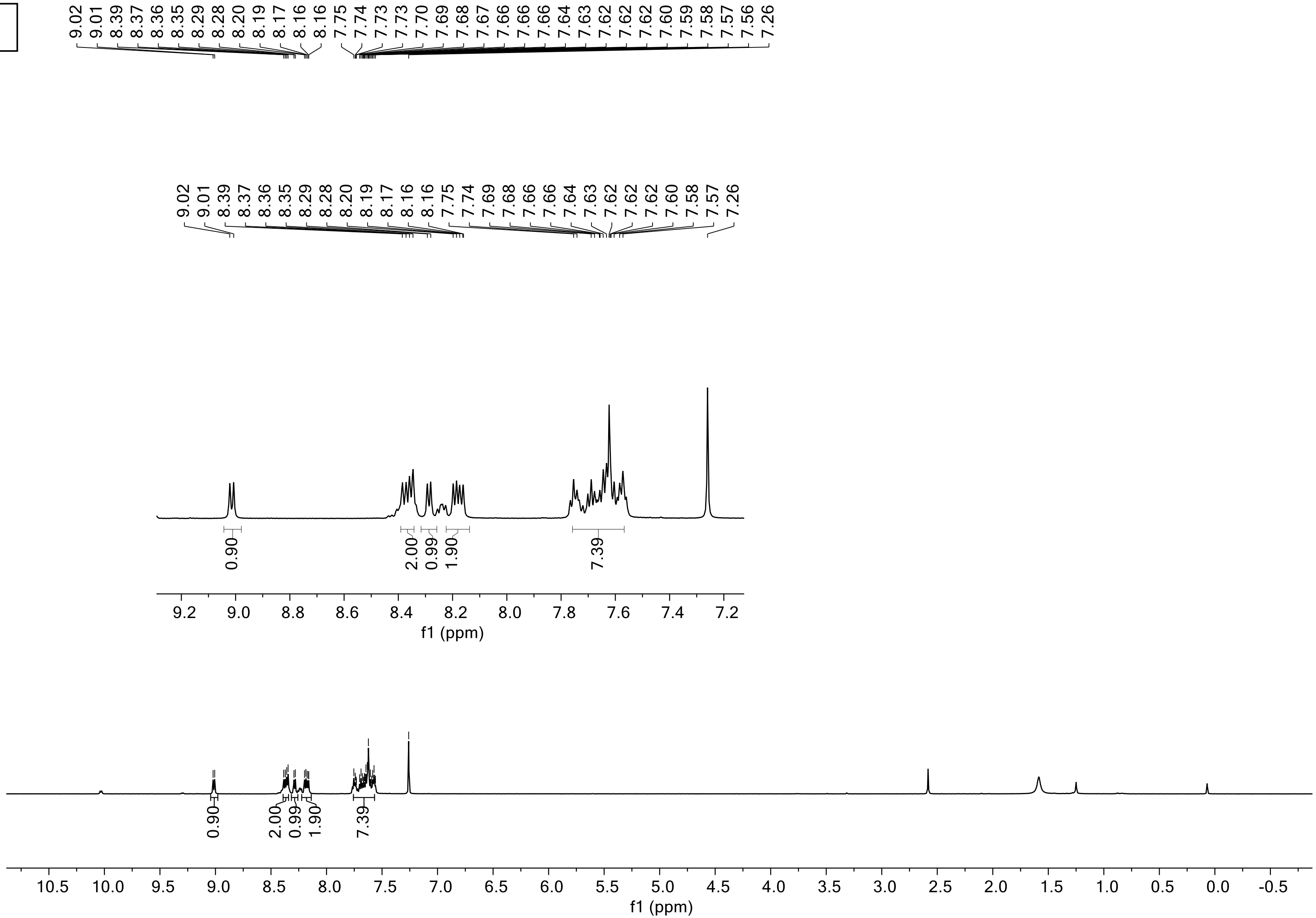

Figure S 73. ^{1}H NMR (top) spectra of **IQ5** in $CDCl_3$.

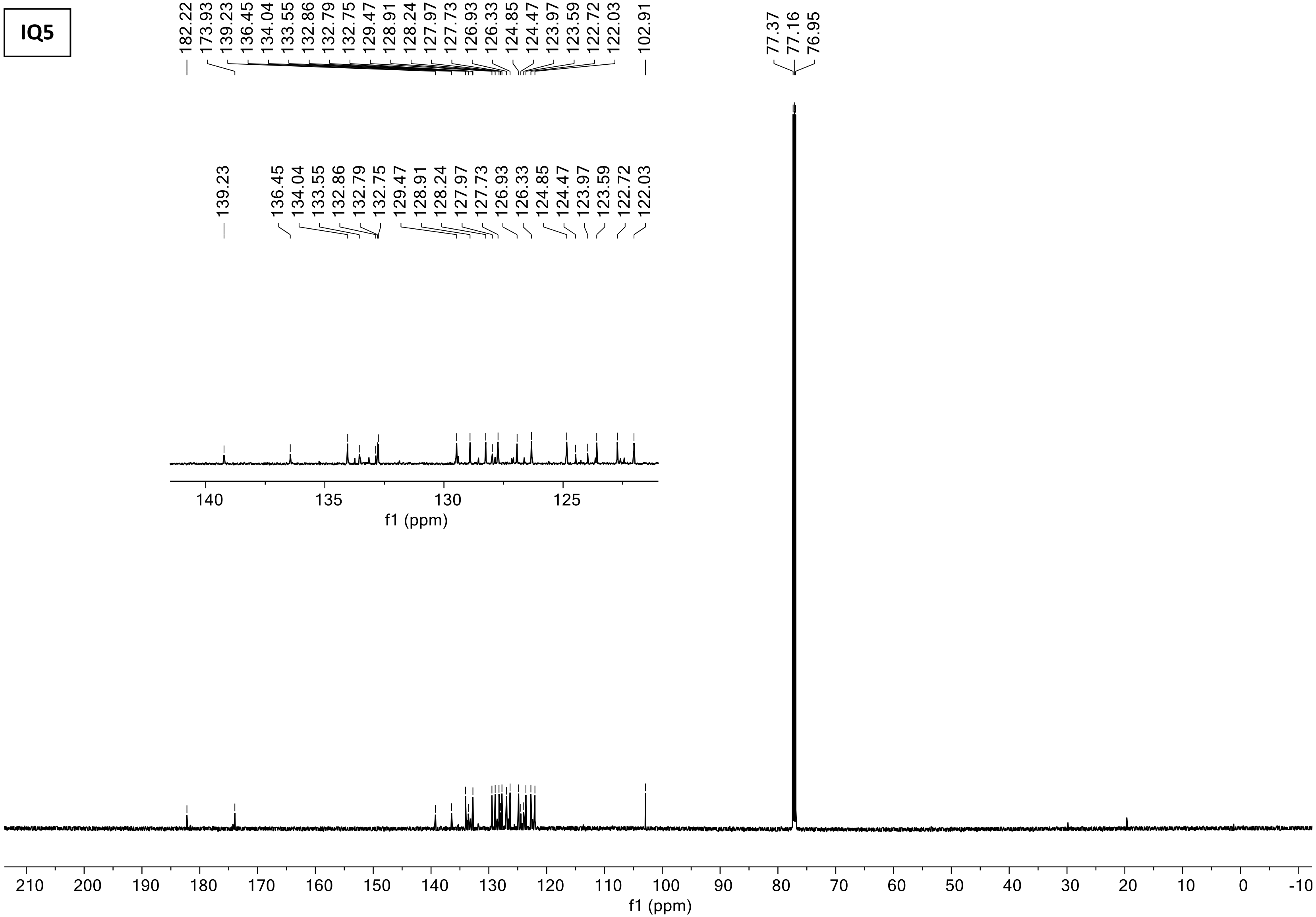

Figure S 74. ^{13}C NMR spectra of **IQ5** in $CDCl_3$.

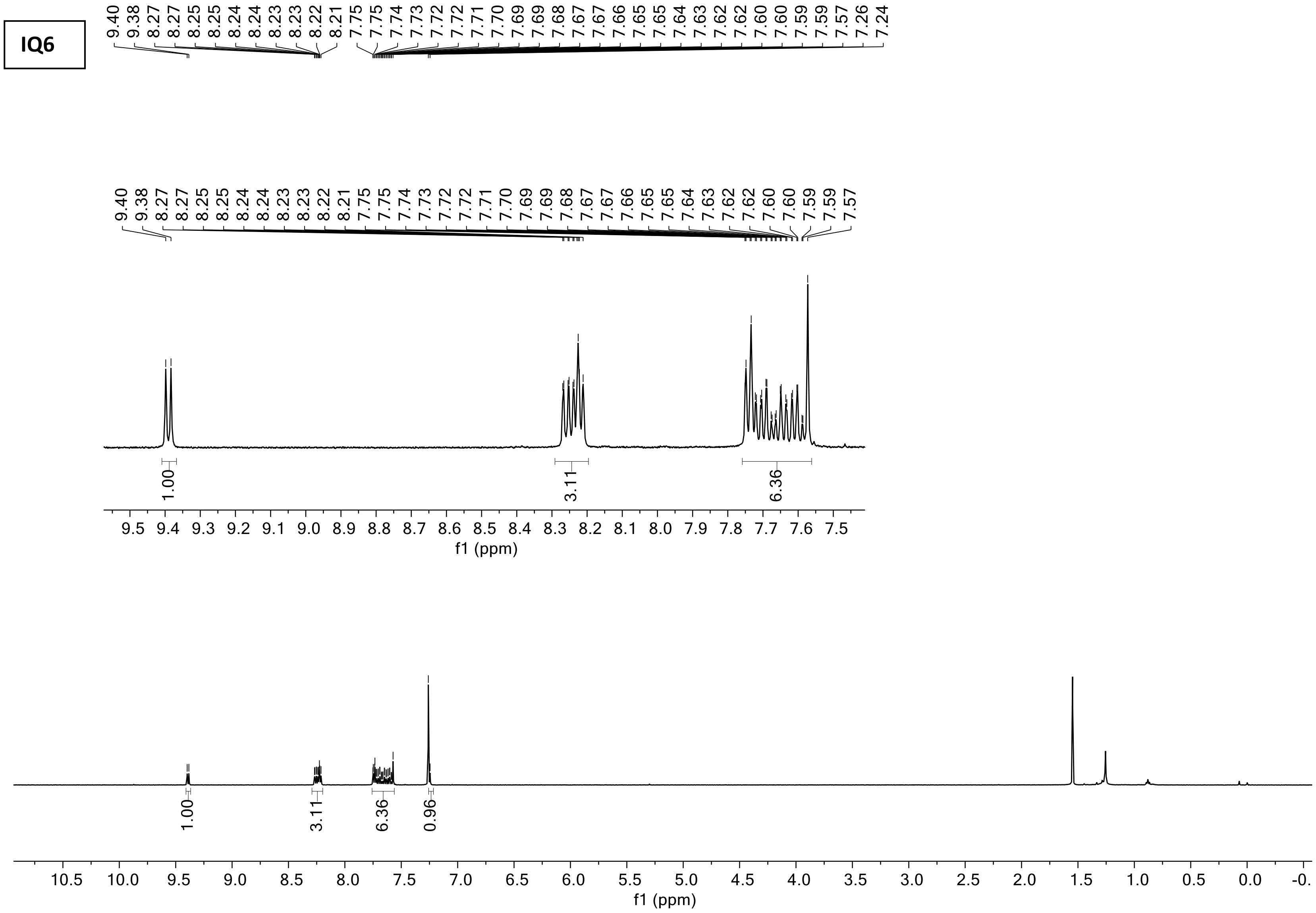

Figure S 75. ^{1}H NMR (top) spectra of **IQ6** in DMSO-d_6.

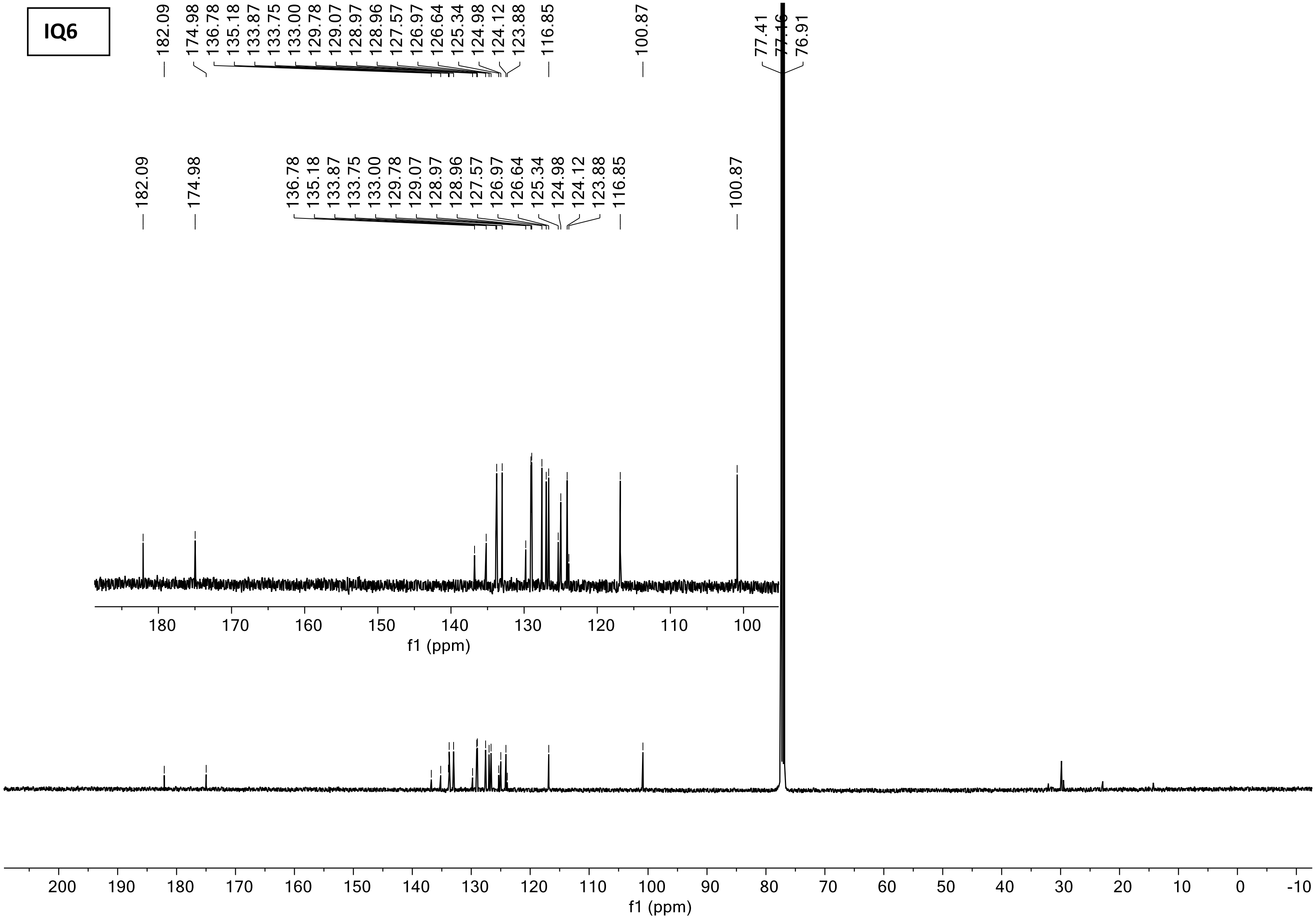

Figure S 76. ^{13}C NMR spectra of **IQ6** in DMSO-d_6.

IQ8a

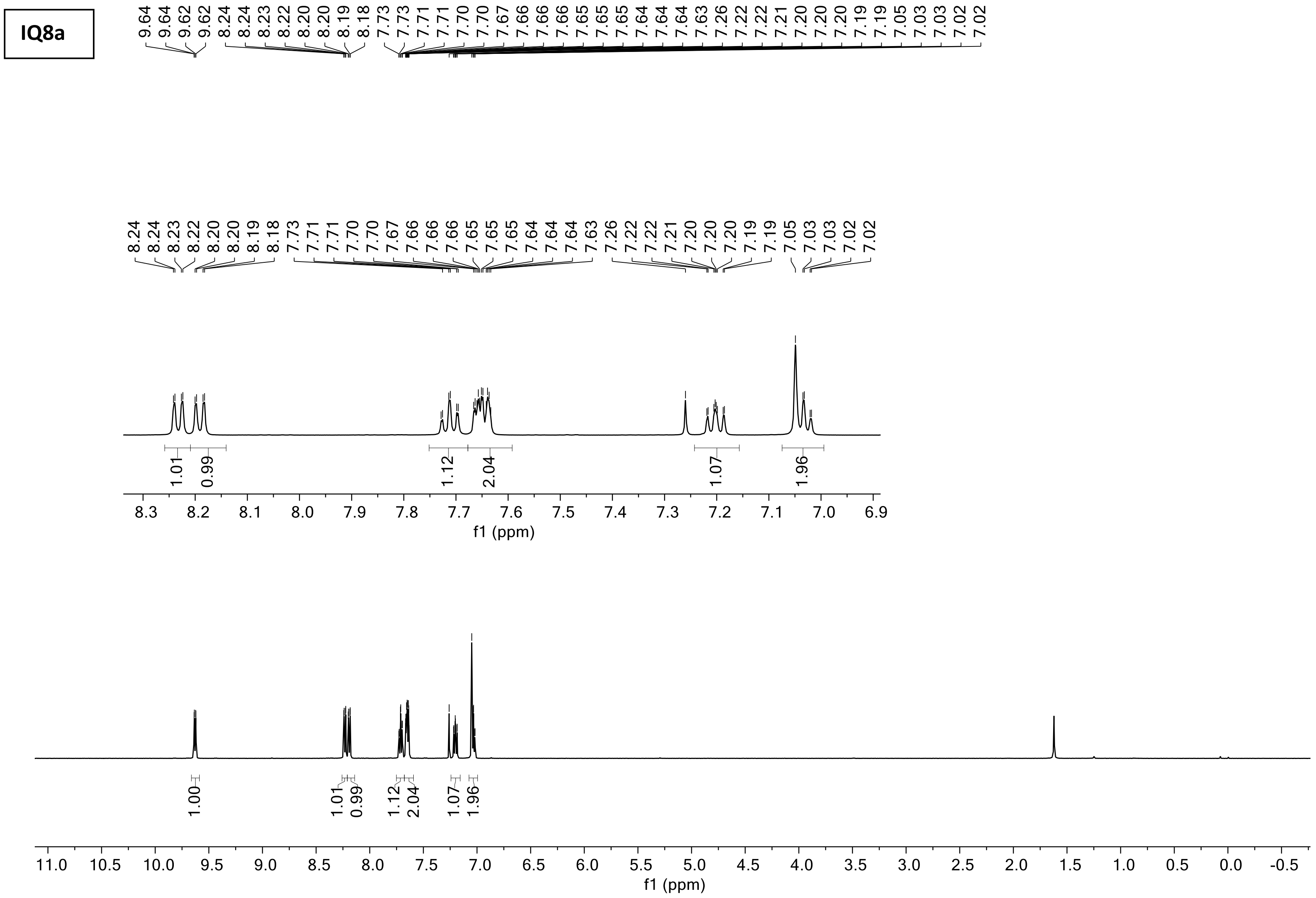

Figure S 77. ^{1}H NMR (top) spectra of **IQ8a** in $CDCl_3$.

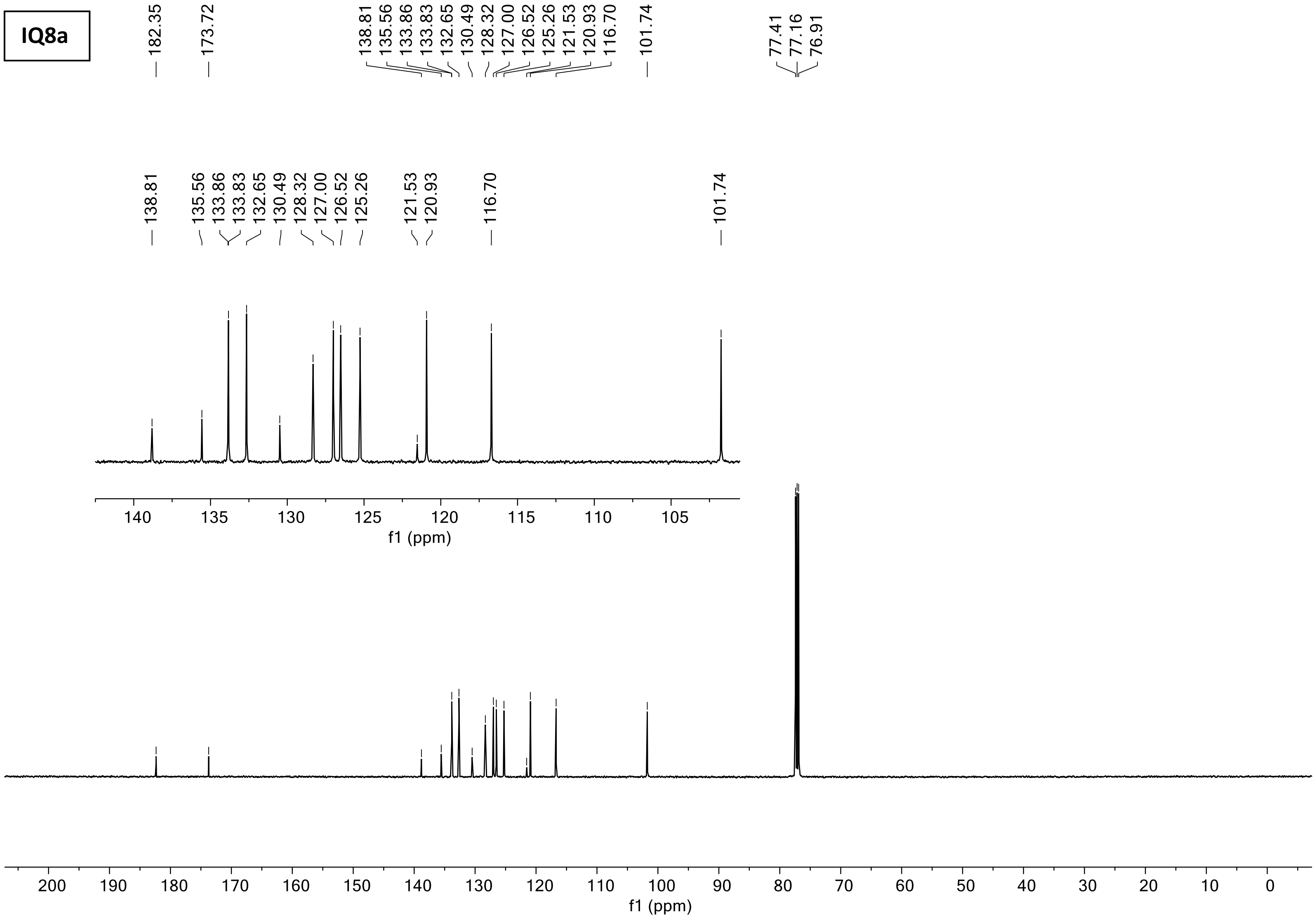

Figure S 78. ^{13}C NMR spectra of **IQ8a** in $CDCl_3$.

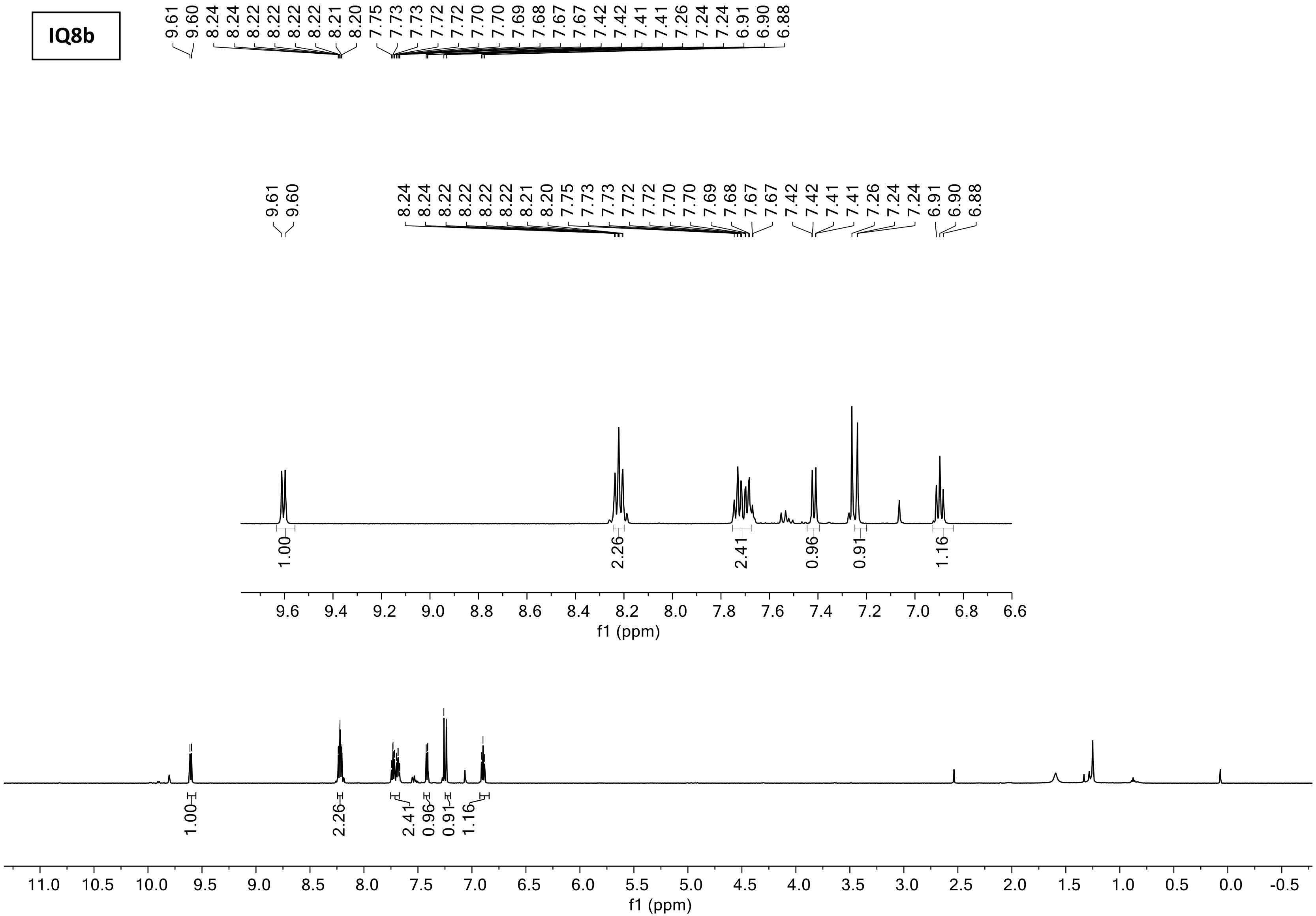

Figure S 79. ^{1}H NMR (top) spectra of **IQ8b** in $CDCl_3$.

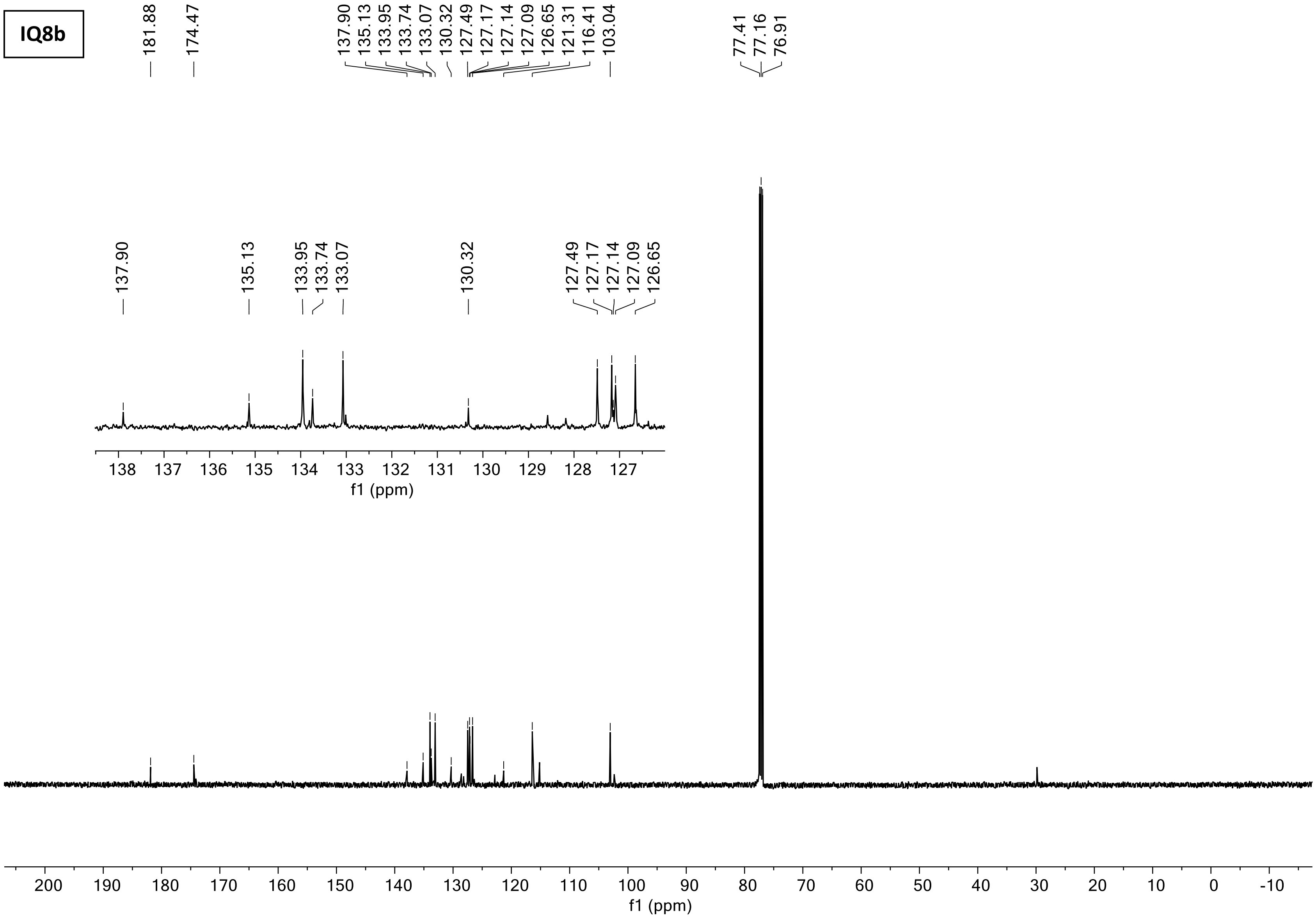

Figure S 80. ^{13}C NMR spectra of **IQ8b** in $CDCl_3$.

IQ8c

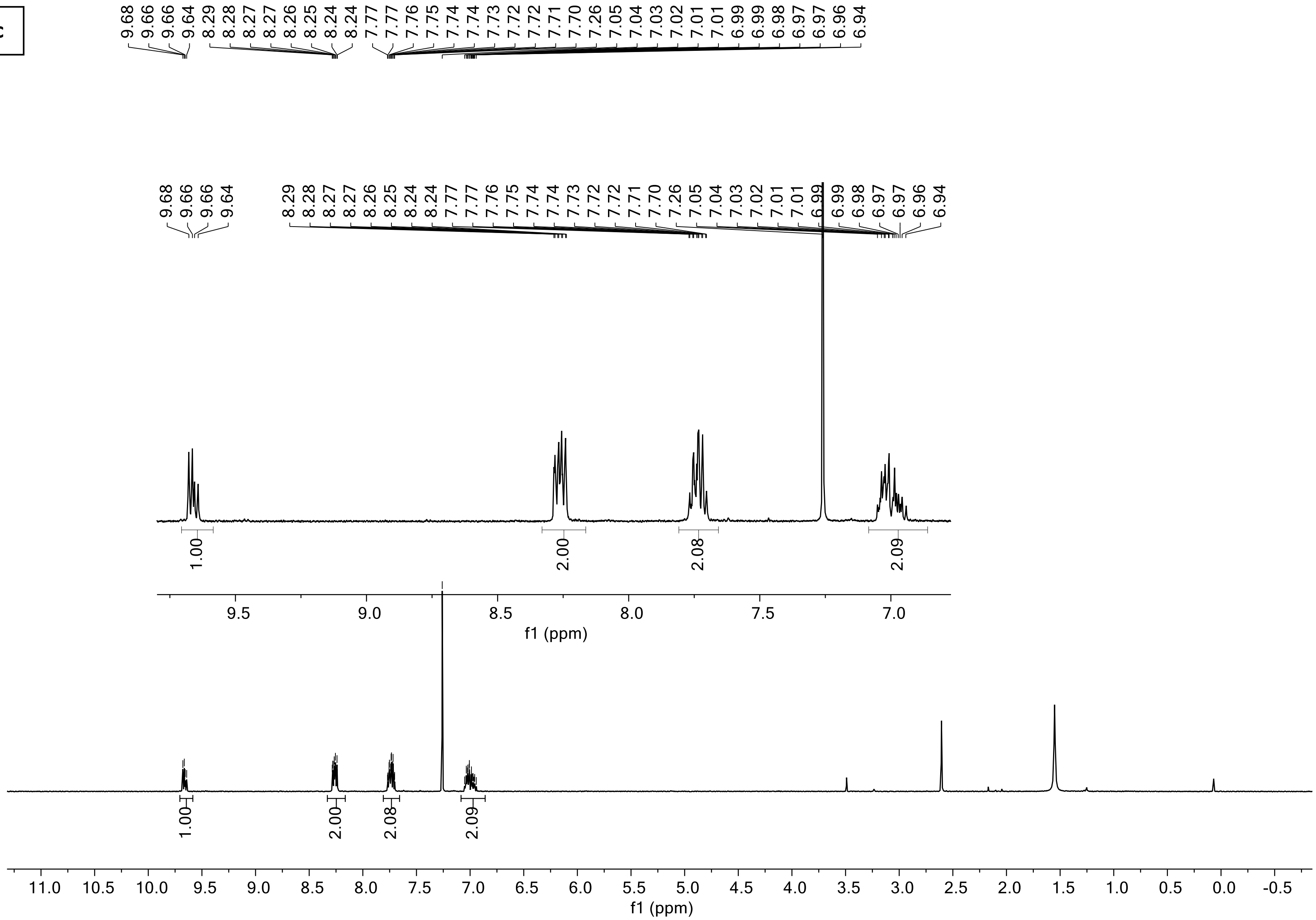

Figure S 81. ^{1}H NMR (top) spectra of **IQ8c** in $CDCl_3$.

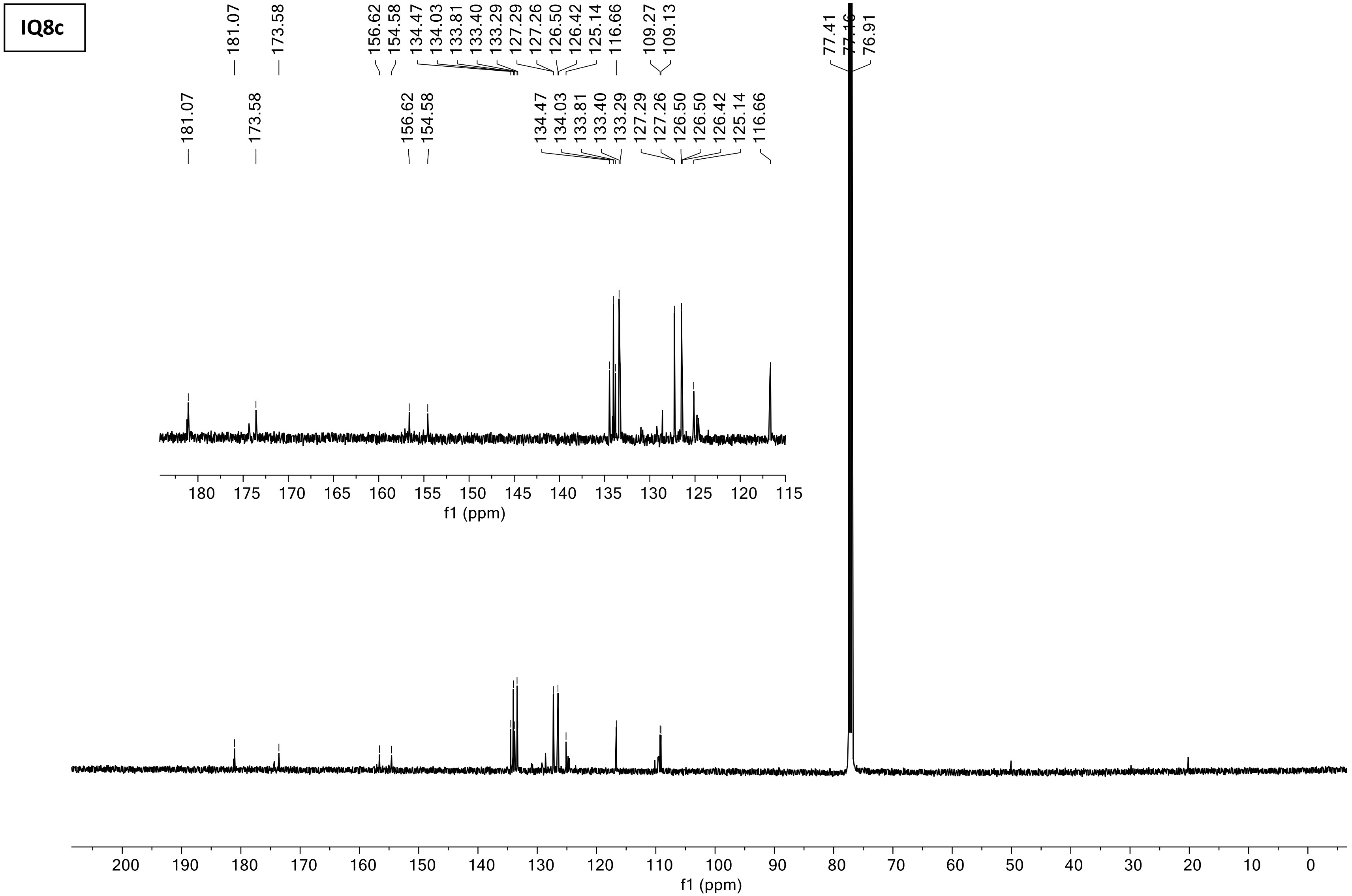

Figure S 82. ^{13}C NMR spectra of **IQ8c** in $CDCl_3$.

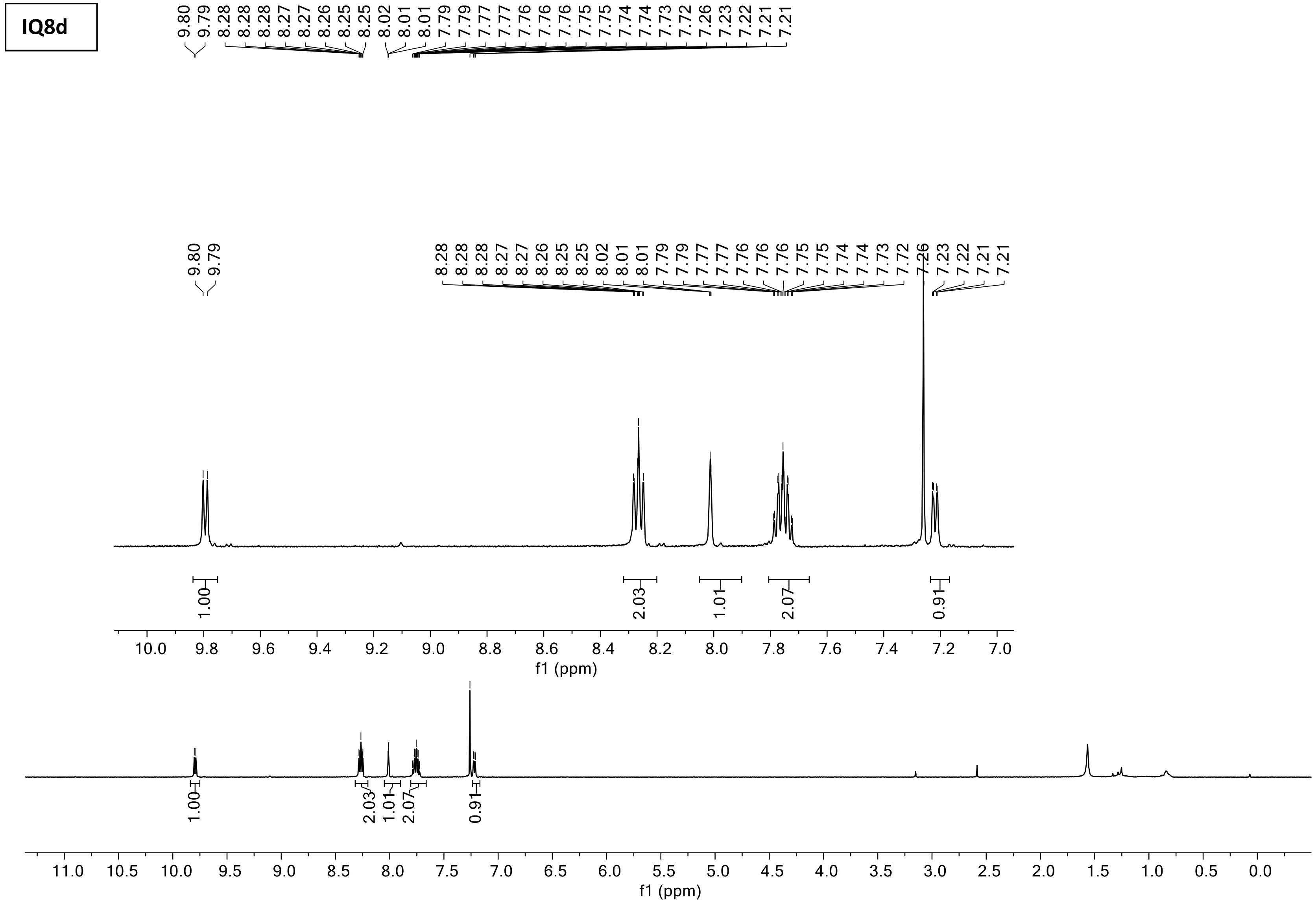

Figure S 83. ^{1}H NMR (top) spectra of **IQ8d** in $CDCl_3$.

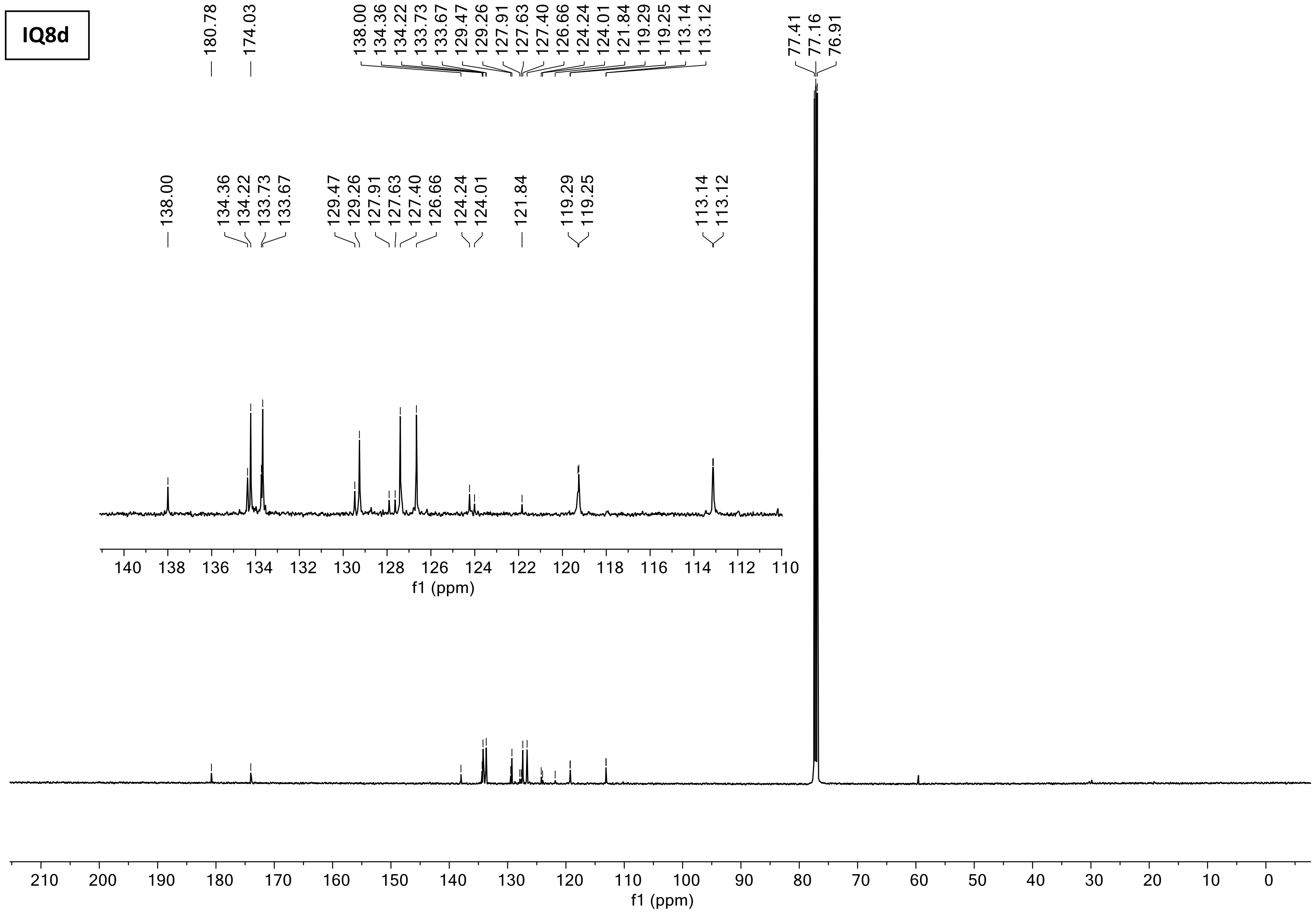

Figure S 84. ^{13}C NMR spectra of **IQ8d** in $CDCl_3$.

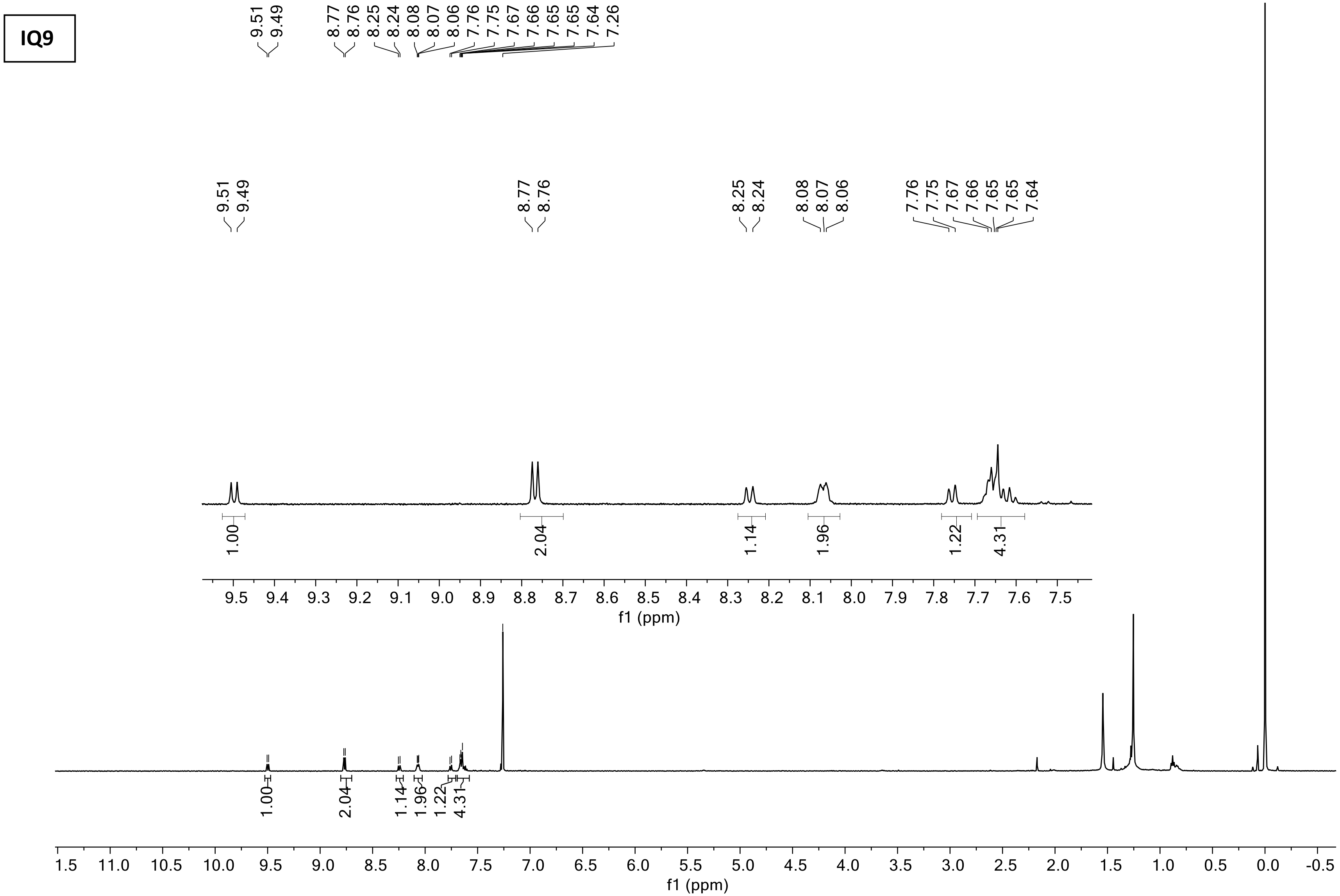

Figure S 85. ^{13}C NMR spectra of **IQ9** in $CDCl_3$.

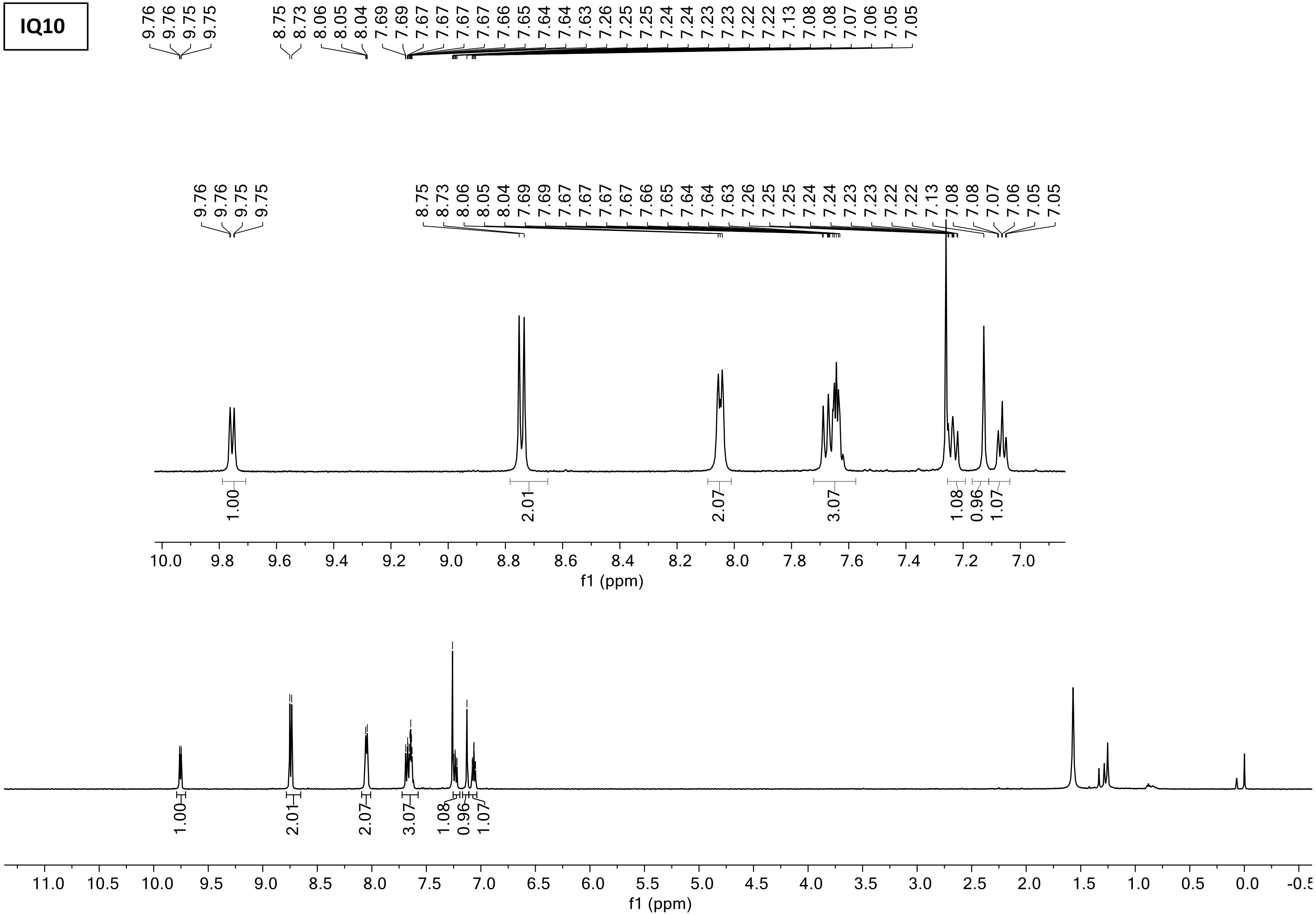

Figure S 86. ^{1}H NMR (top) spectra of **IQ10** in $CDCl_3$.

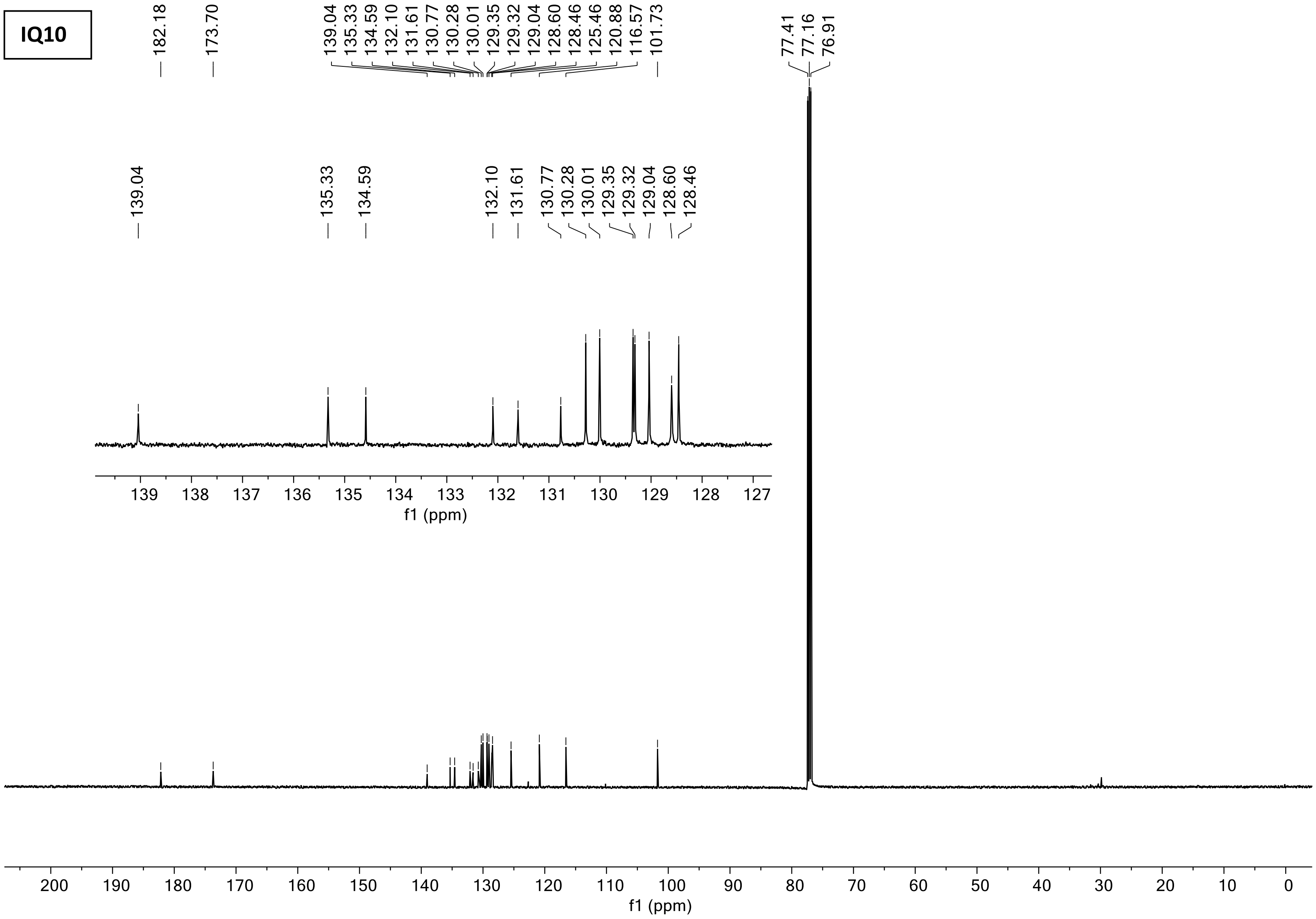

Figure S 87. ^{13}C NMR spectra of **IQ10** in $CDCl_3$.

I1

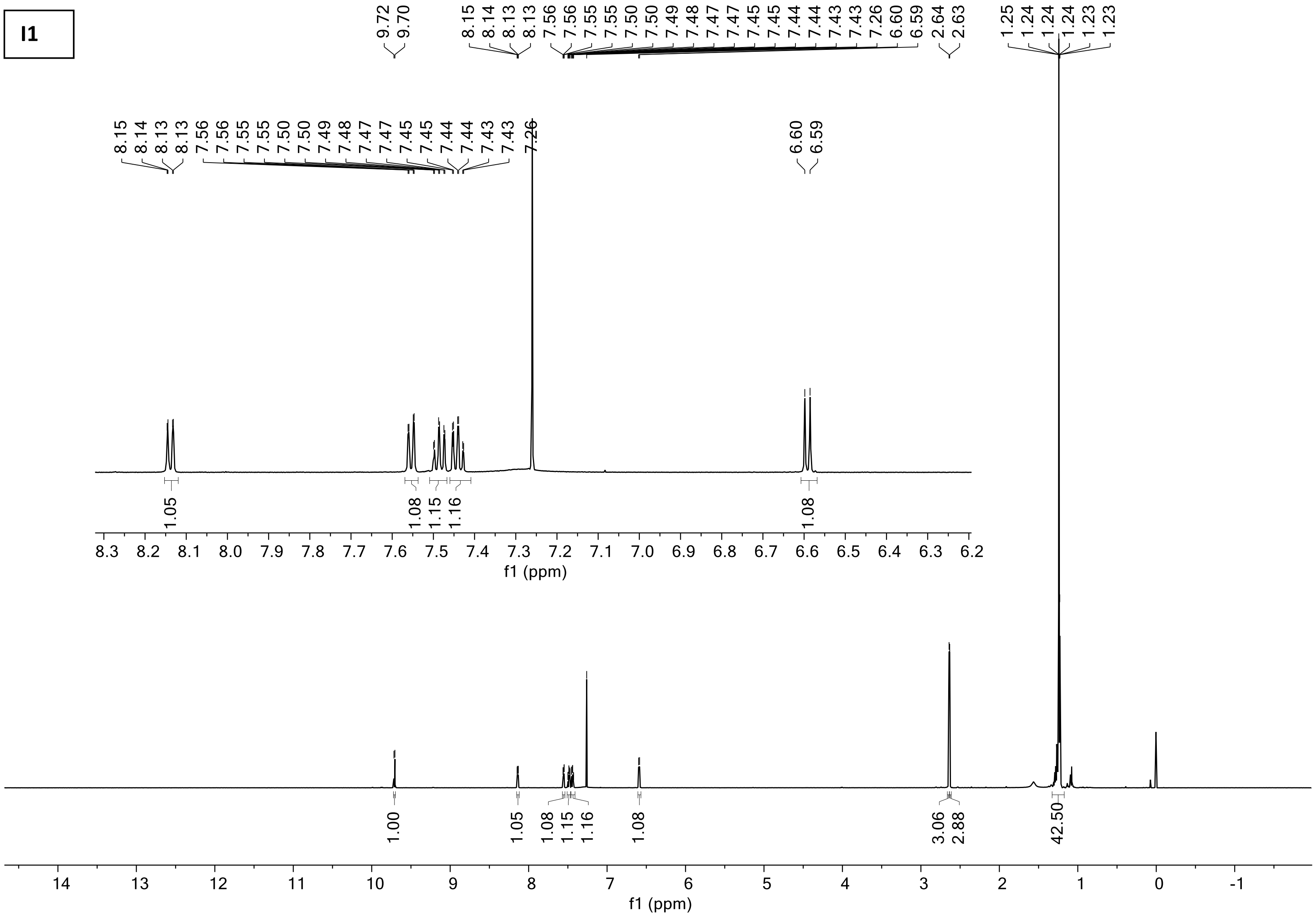

Figure S 88. ^{1}H NMR (top) spectra of **I1** in $CDCl_3$.

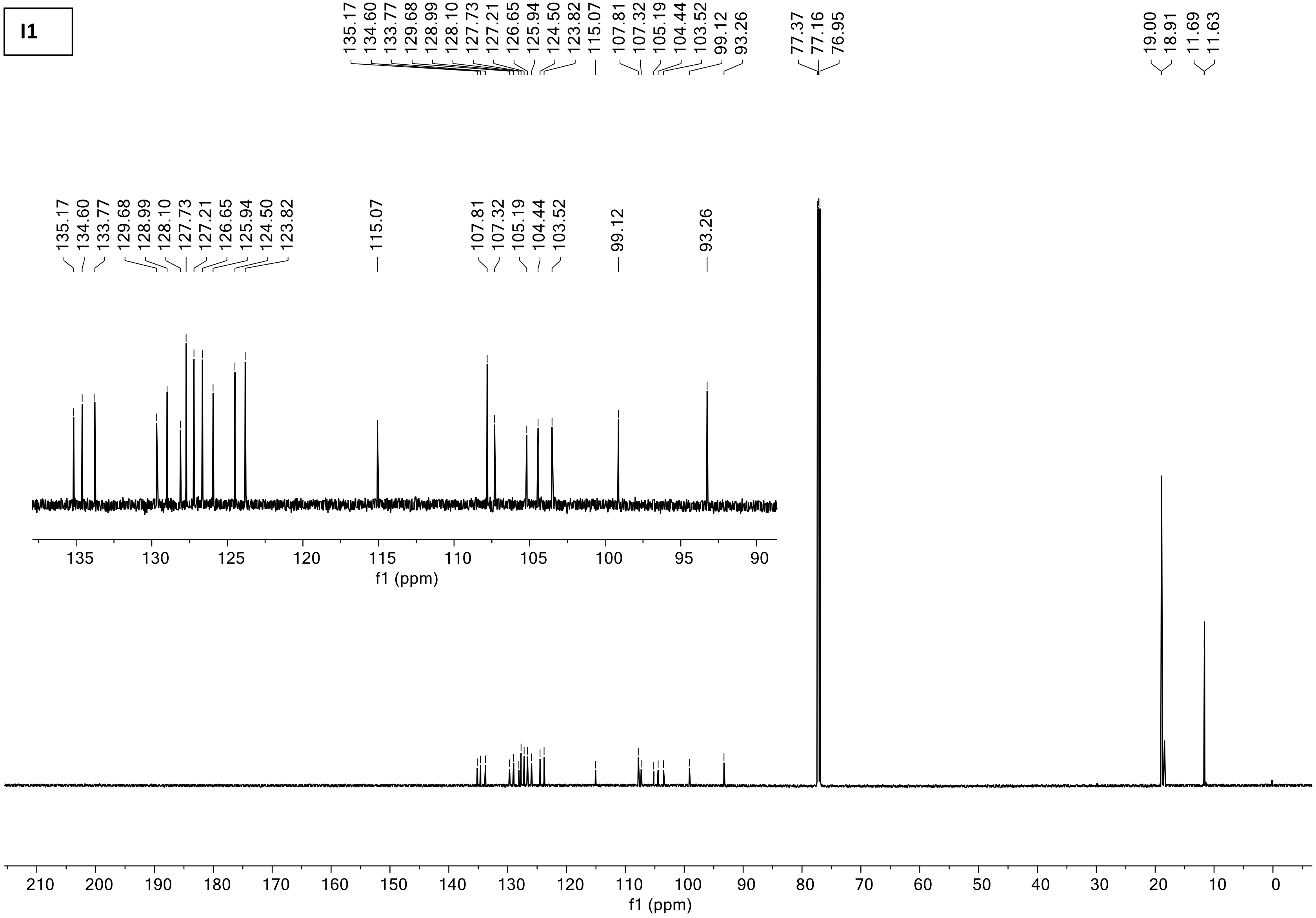

Figure S 89. ^{13}C NMR spectra of **I1** in $CDCl_3$.

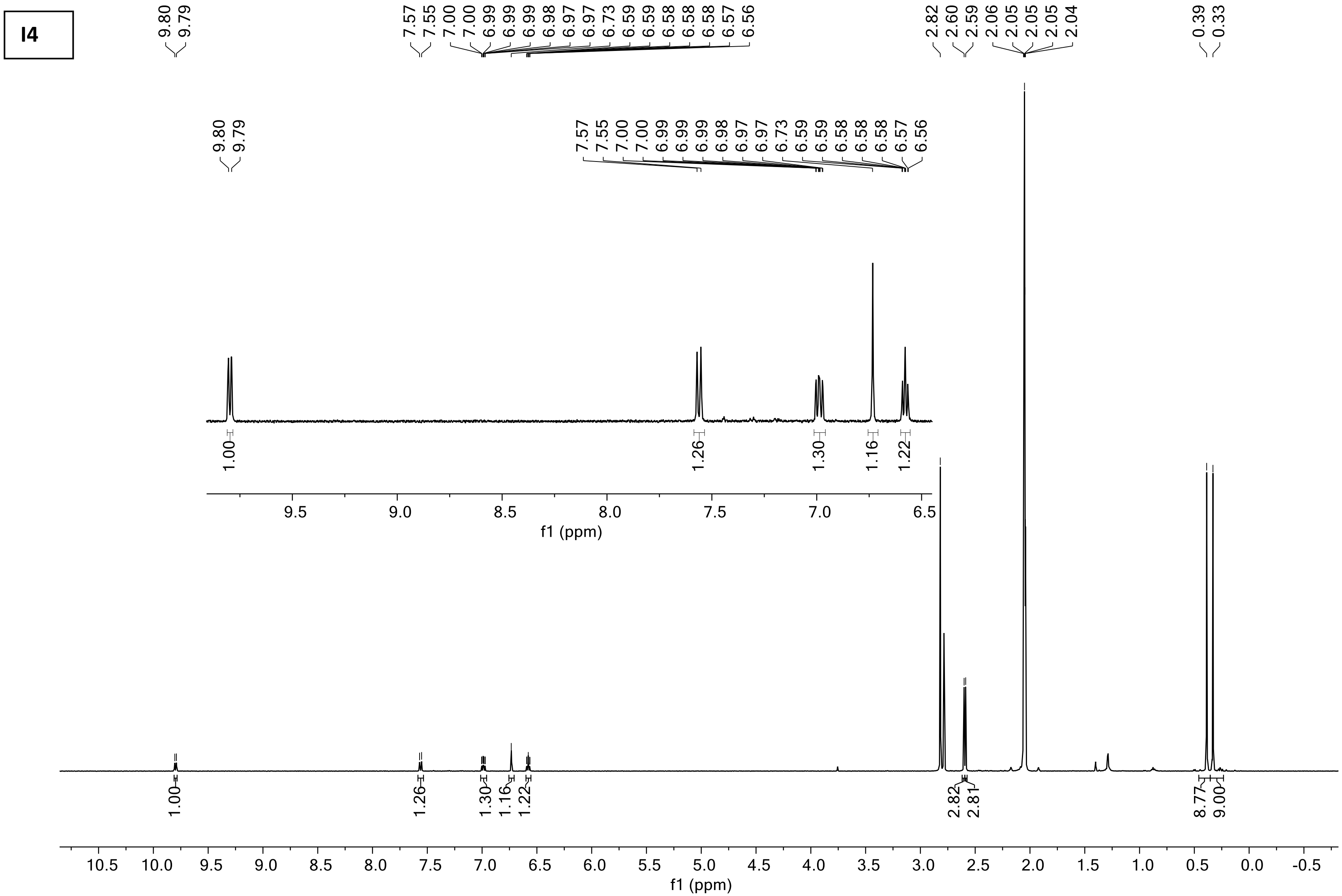

Figure S 90. ^{1}H NMR (top) spectra of **I4** in Acetone-d_6.

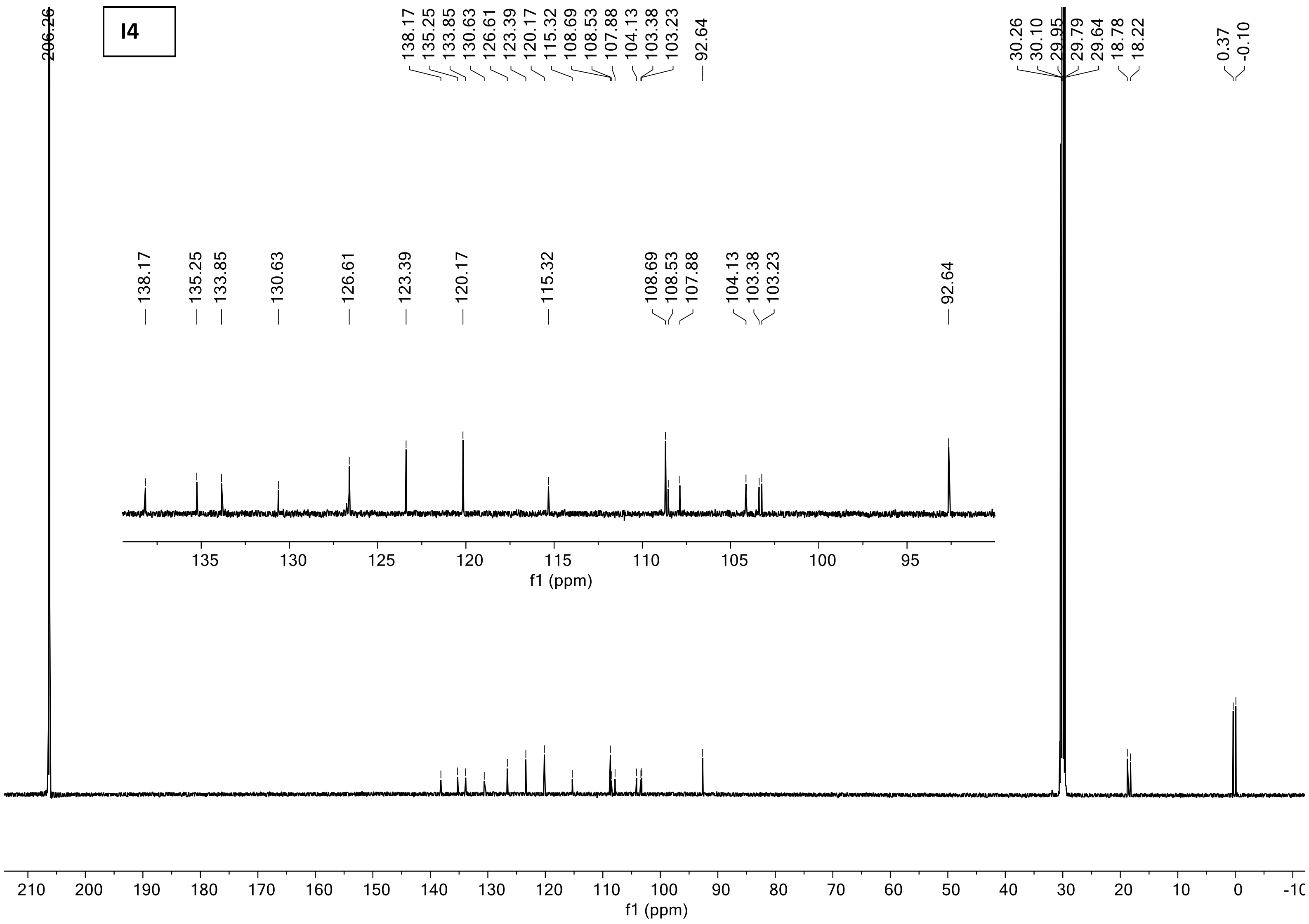

Figure S 91. ^{13}C NMR spectra of **I4** in Acetone-d_6.

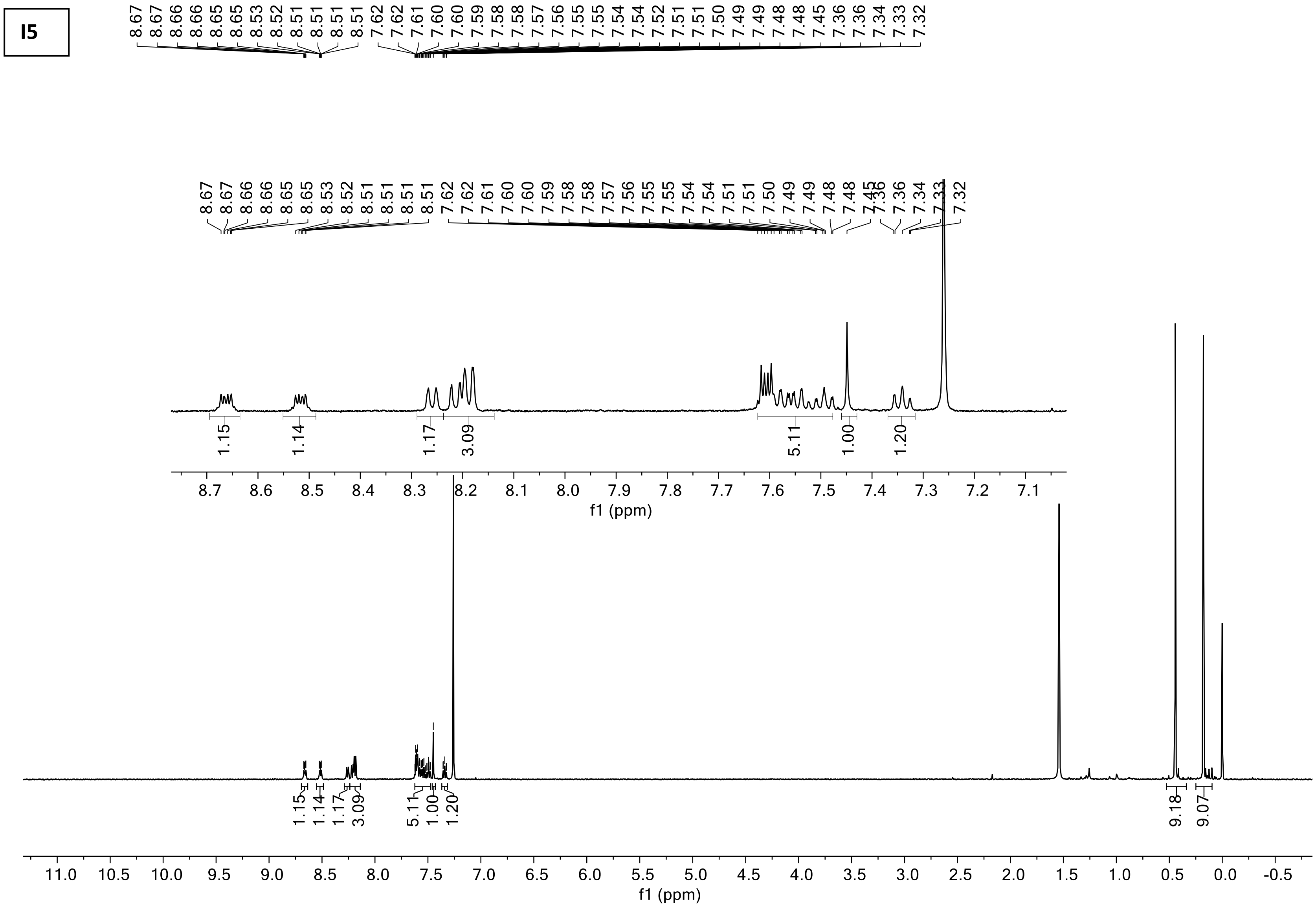

Figure S 92. ^{1}H NMR (top) spectra of **I5** in $CDCl_3$.

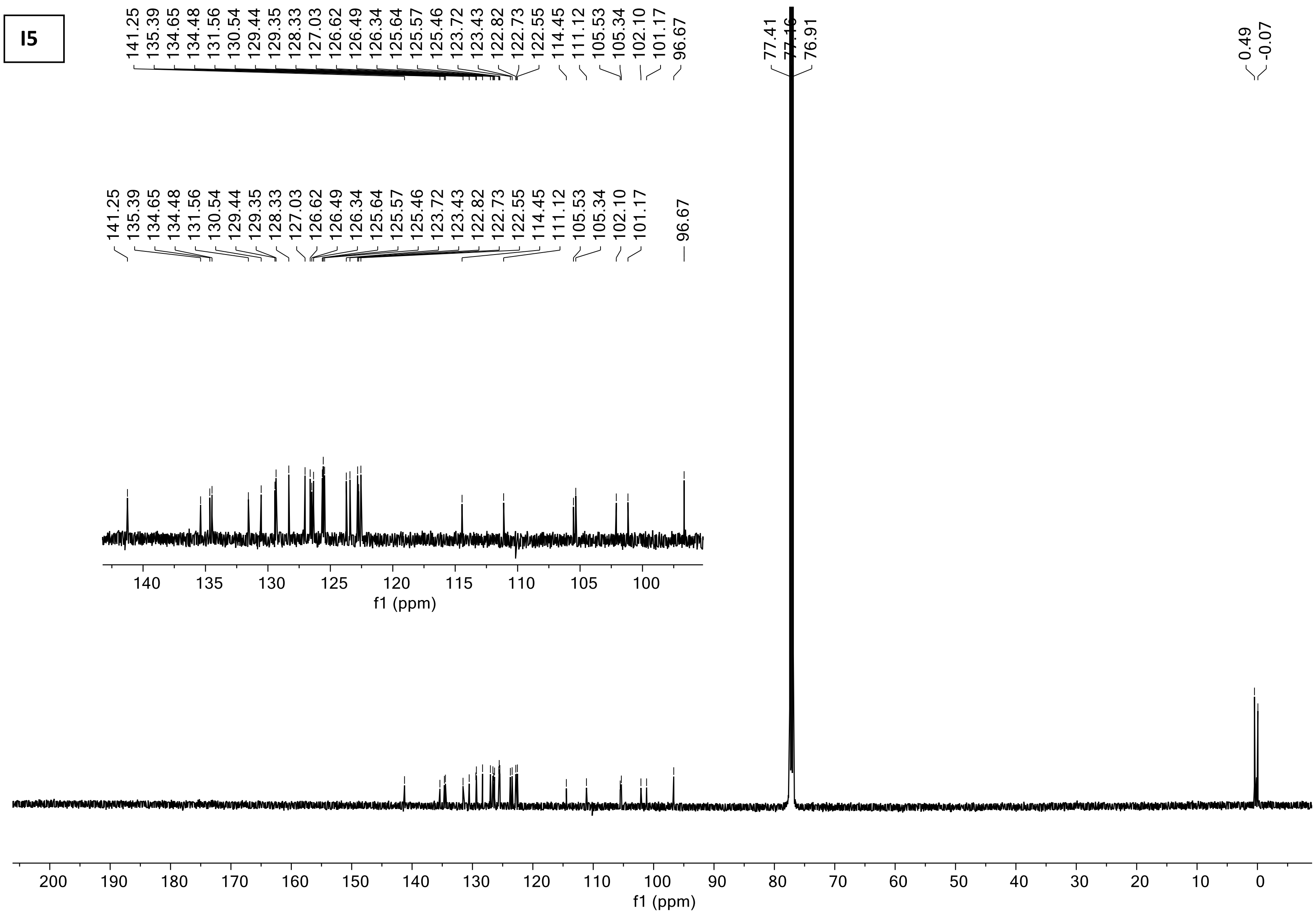

Figure S 93. ^{13}C NMR spectra of **I5** in $CDCl_3$.

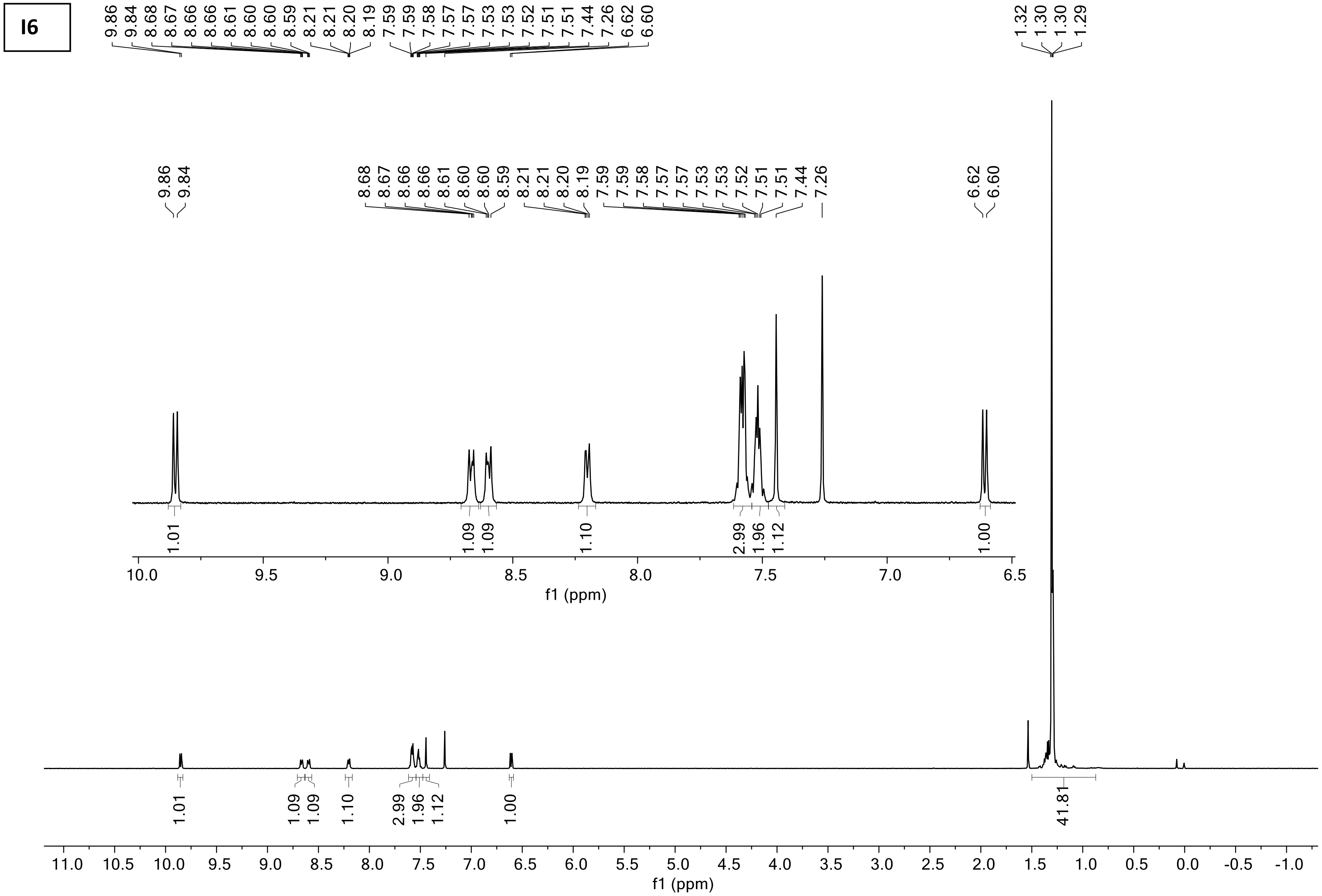

Figure S 94. ^{1}H NMR (top) spectra of **I6** in $CDCl_3$.

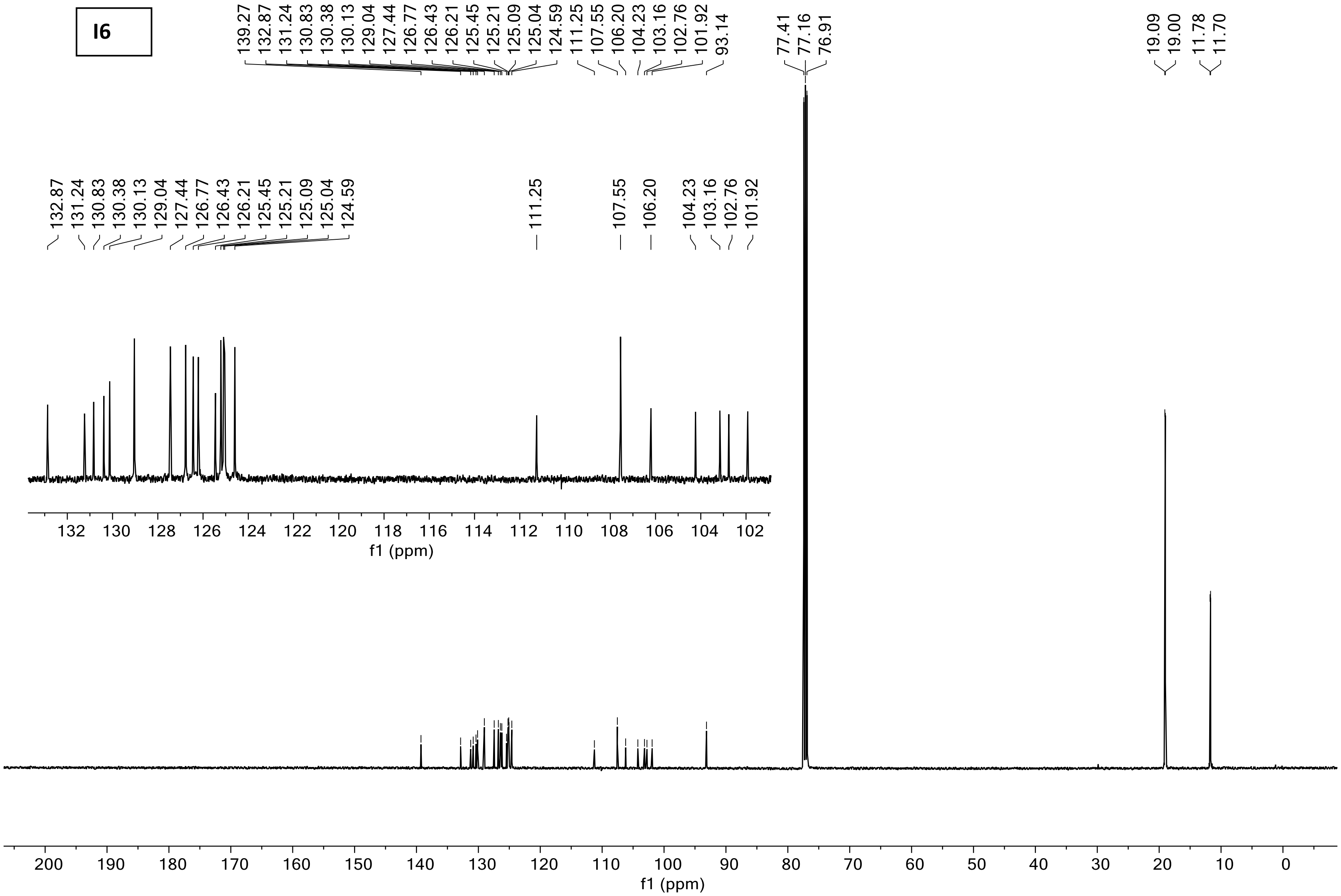

Figure S 95. ^{13}C NMR spectra of **I6** in $CDCl_3$.

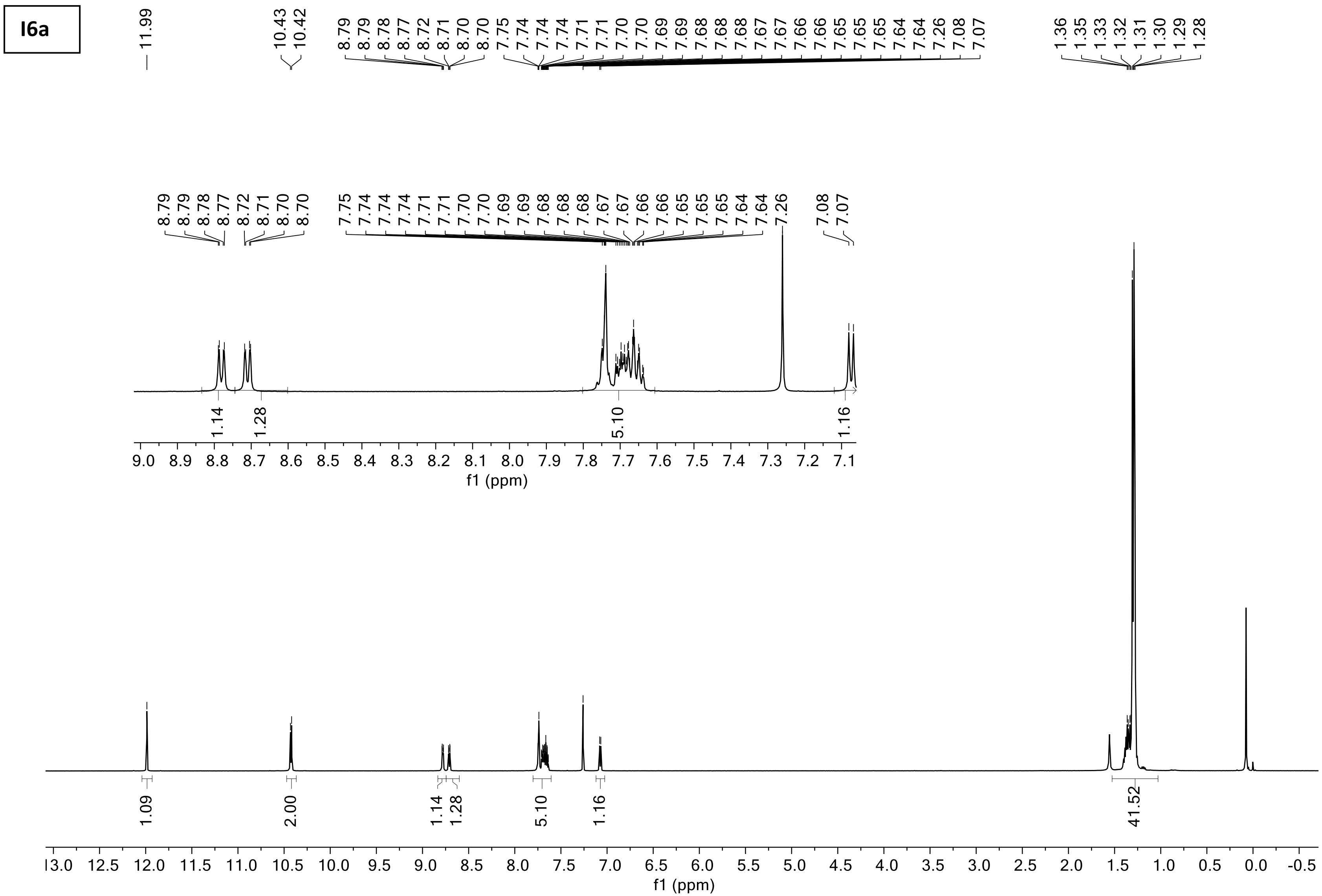

Figure S 96. ^{1}H NMR (top) spectra of **I6a** in $CDCl_3$.

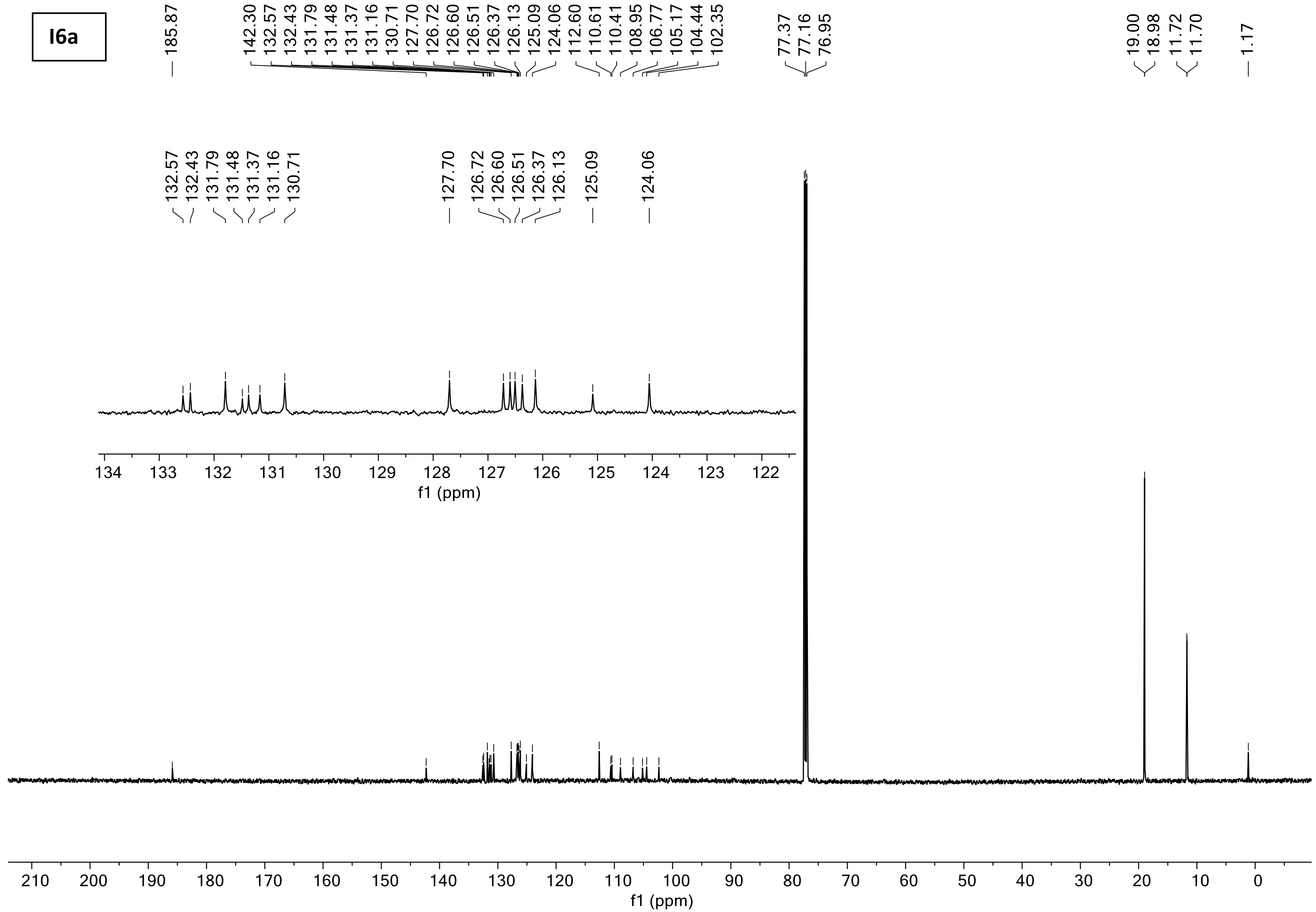

Figure S 97. ^{13}C NMR spectra of **I6a** in $CDCl_3$.

I6b

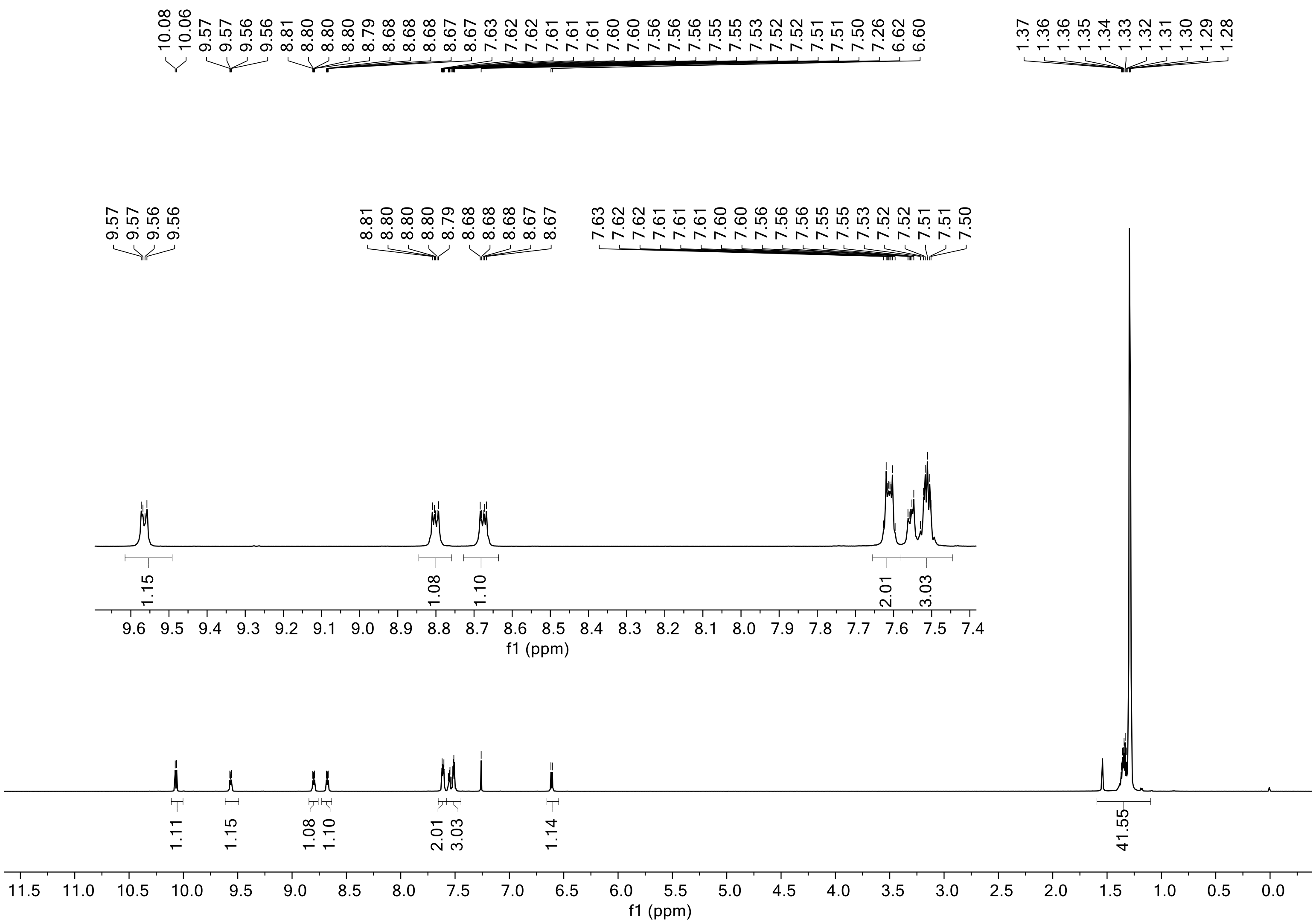

Figure S 98. ^{1}H NMR (top) spectra of **I6b** in $CDCl_3$.

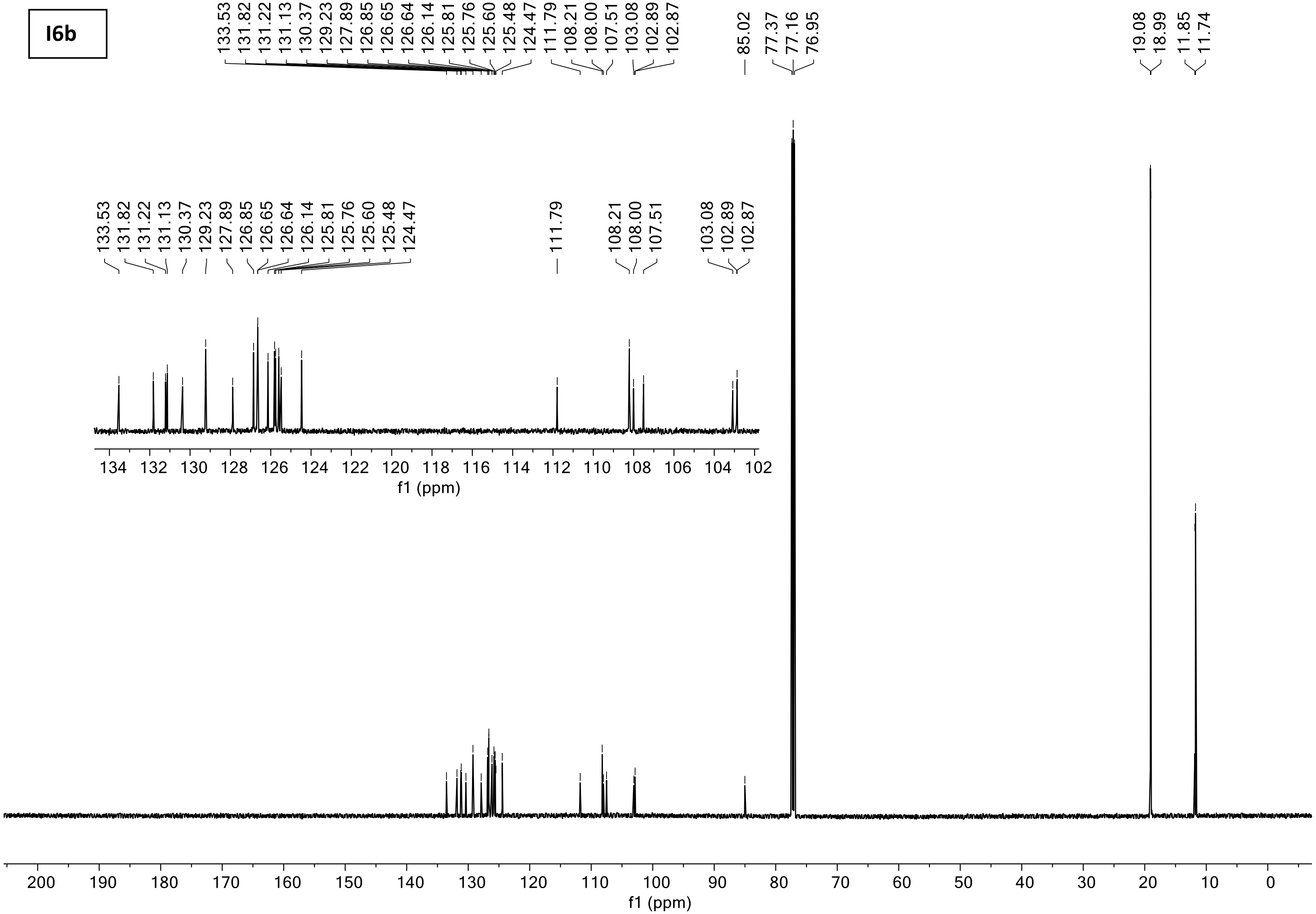

Figure S 99. ^{13}C NMR spectra of **I6b** in $CDCl_3$.

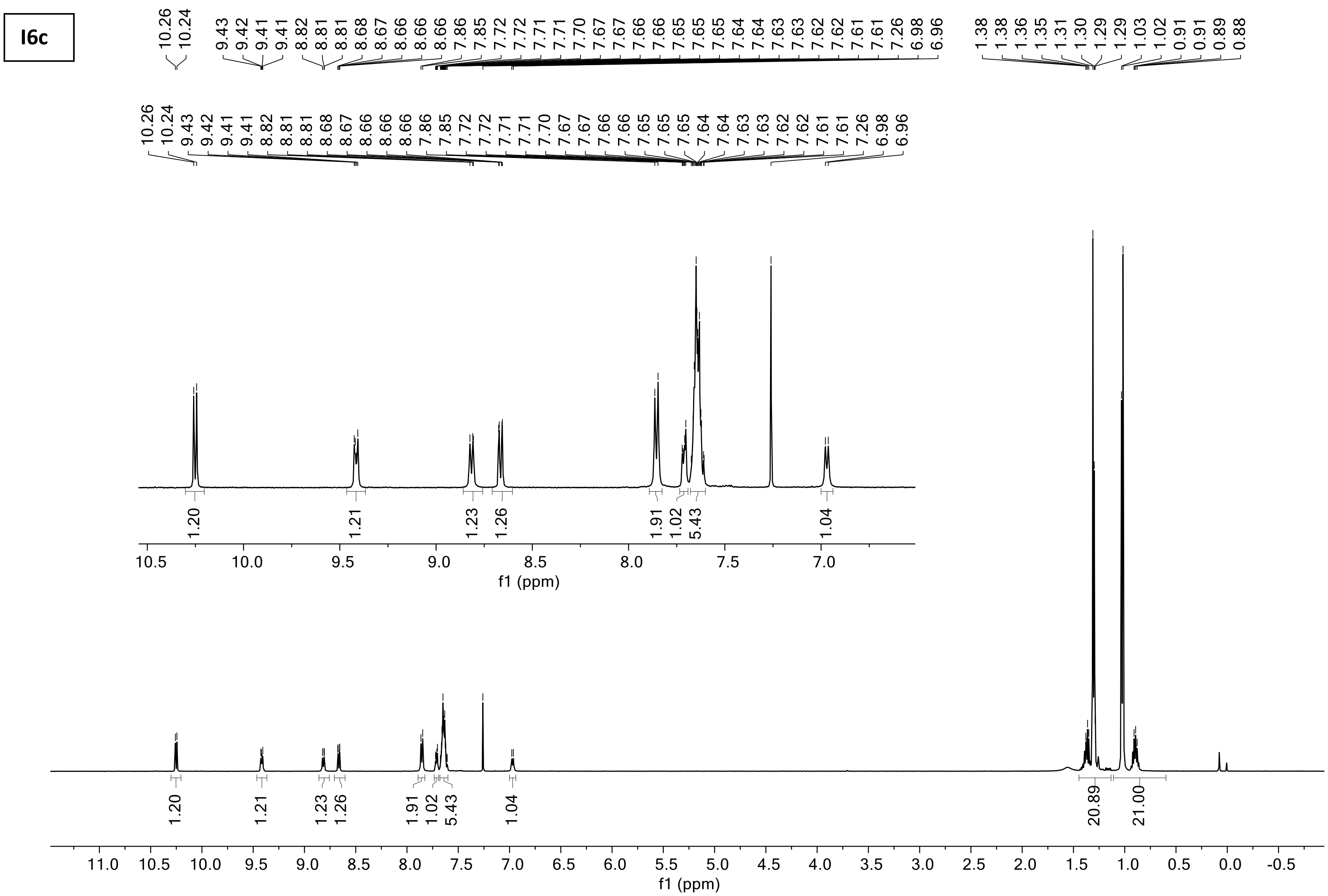

Figure S 100. ^{1}H NMR (top) spectra of **I6c** in $CDCl_3$.

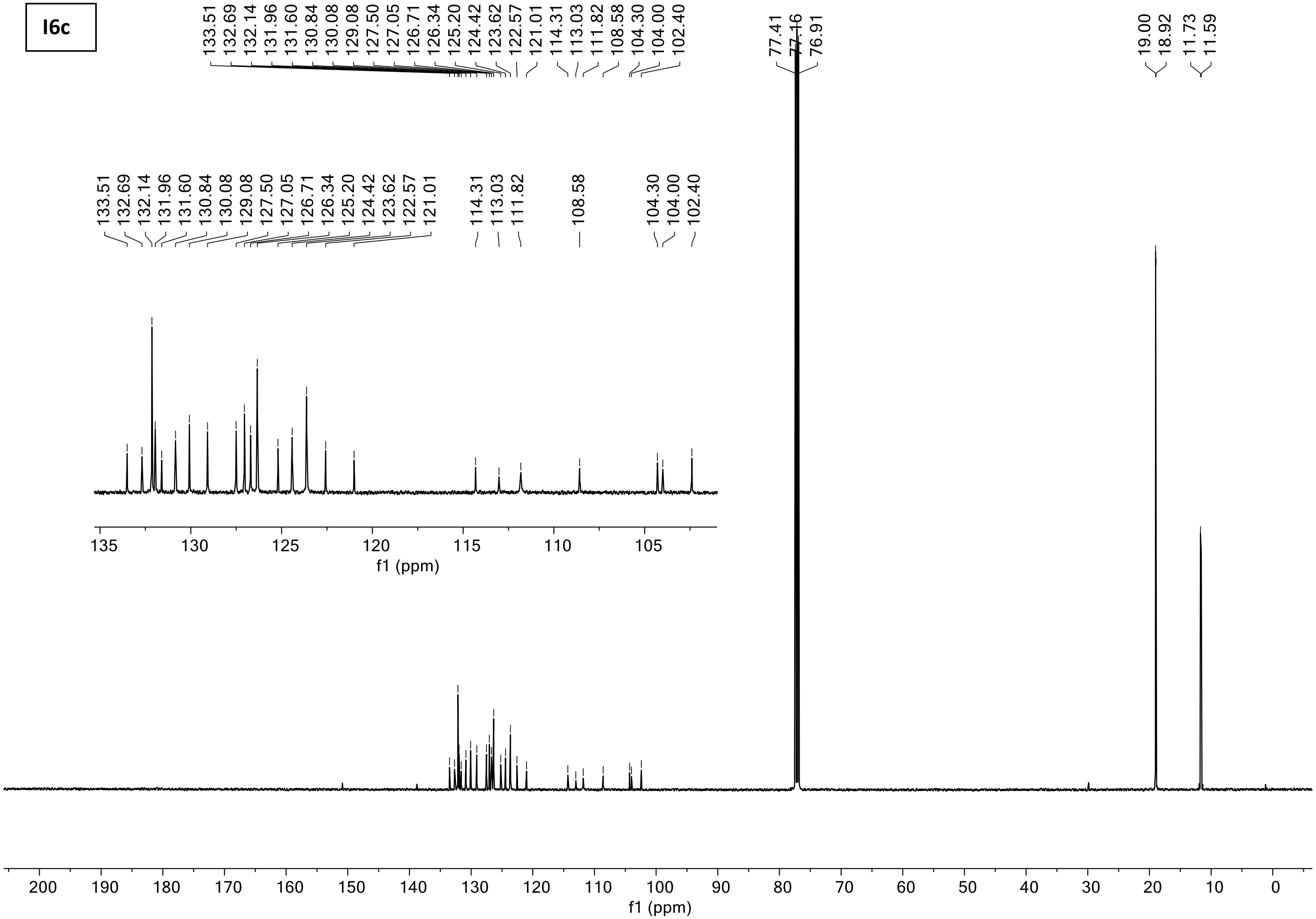

Figure S 101. ^{13}C NMR spectra of **I6c** in $CDCl_3$.

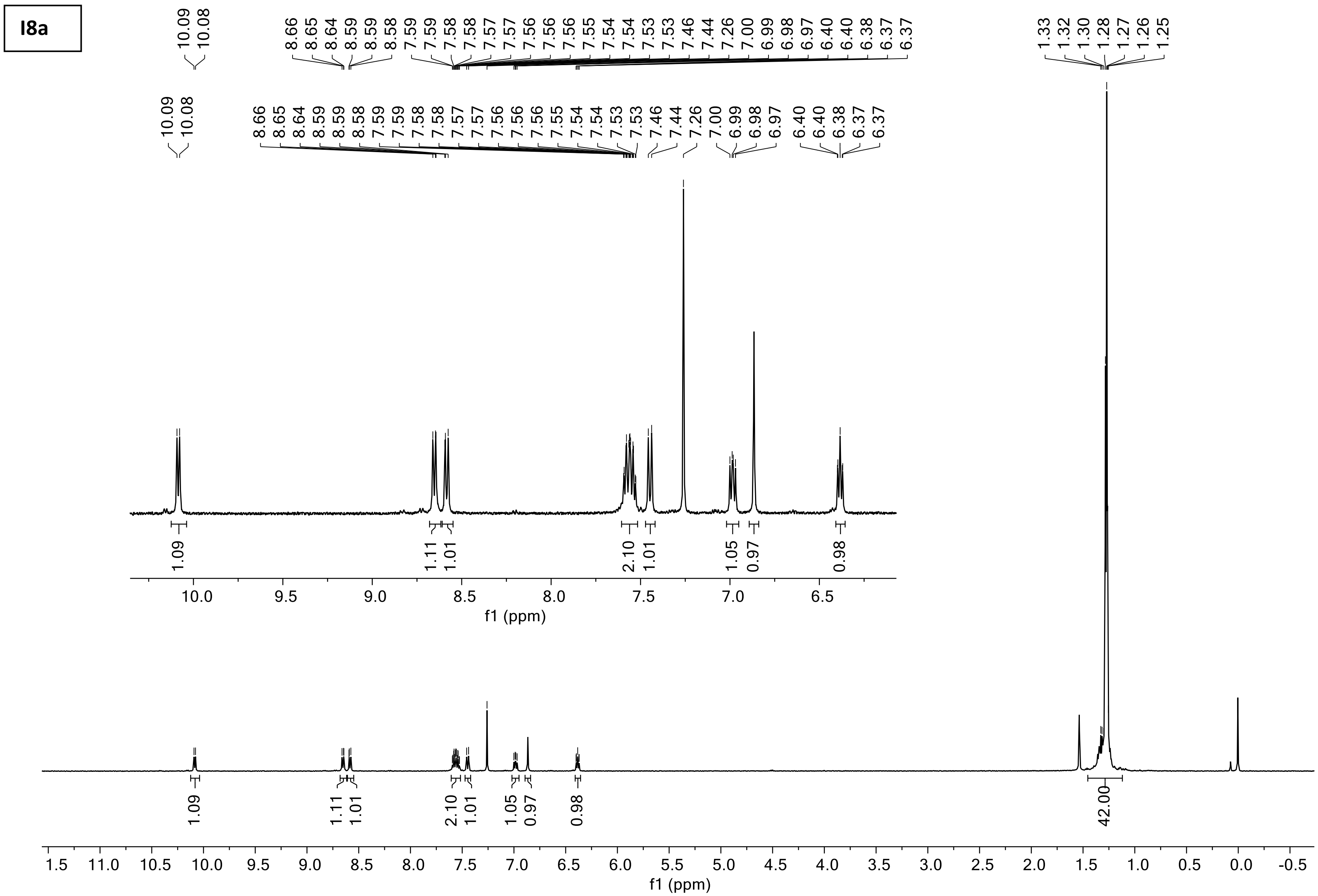

Figure S 102. ^{1}H NMR (top) spectra of **I8a** in $CDCl_3$.

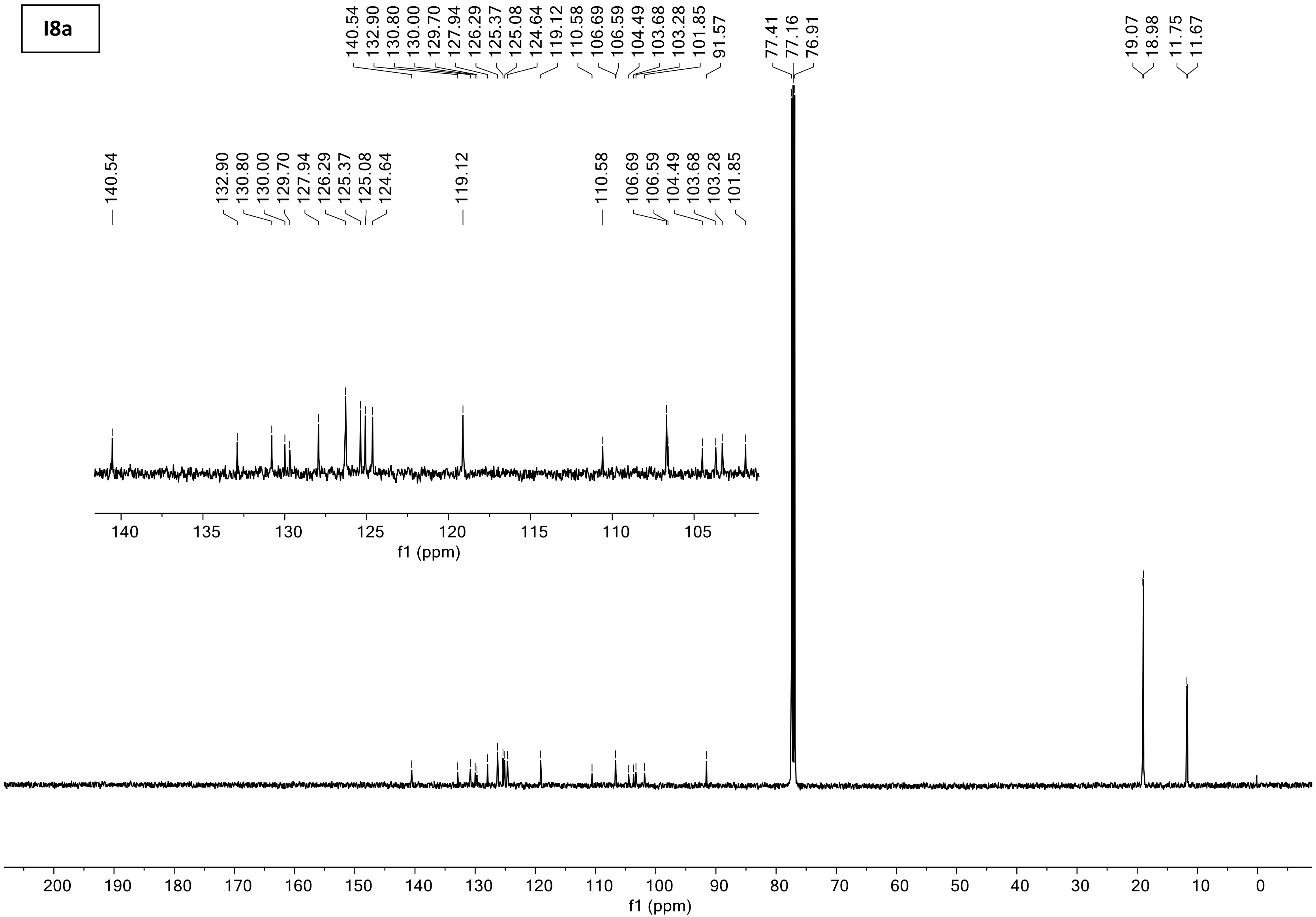

Figure S 103. ^{13}C NMR spectra of **I8a** in $CDCl_3$.

I8b

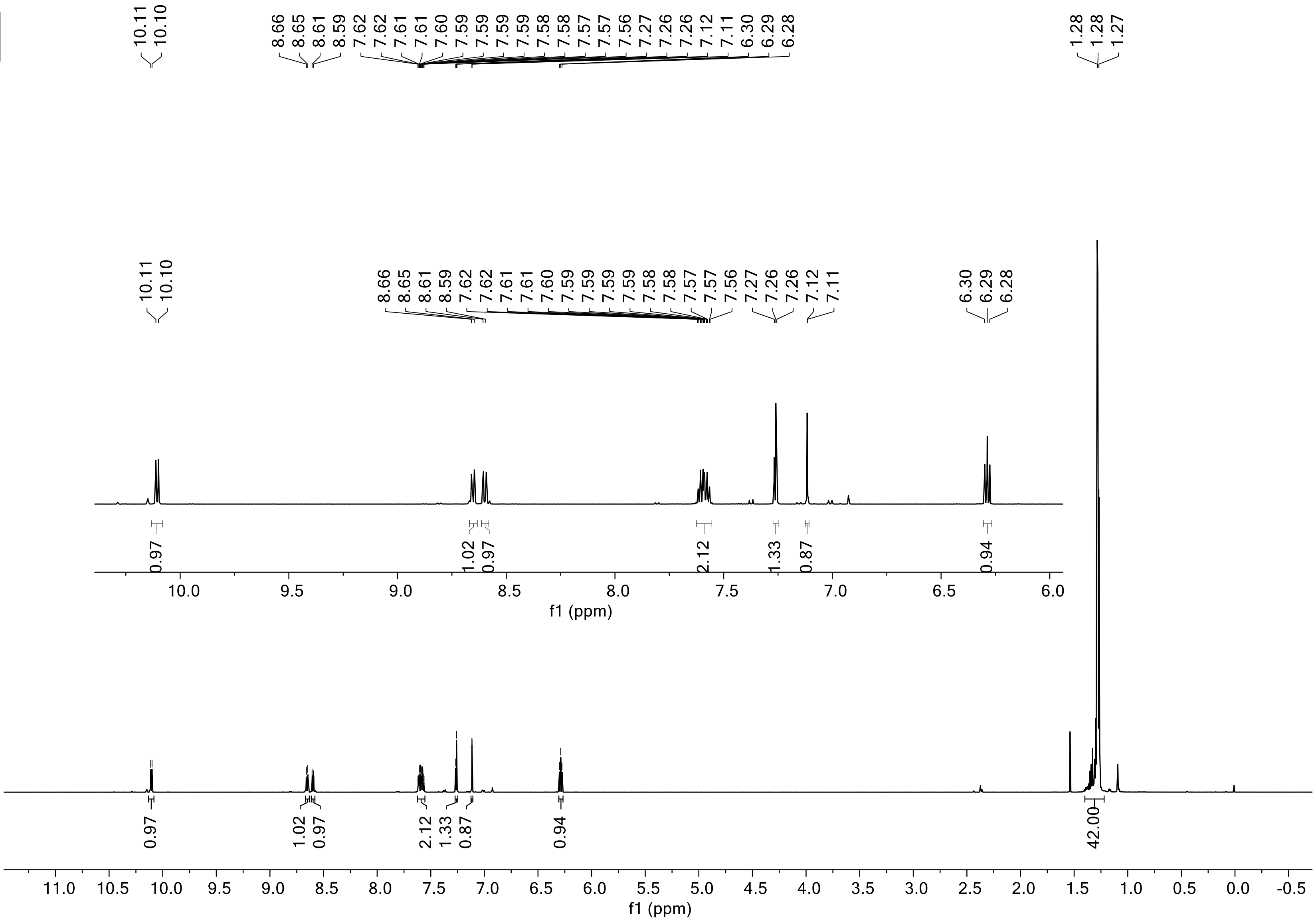

Figure S 104. ^{1}H NMR (top) spectra of **I8b** in $CDCl_3$.

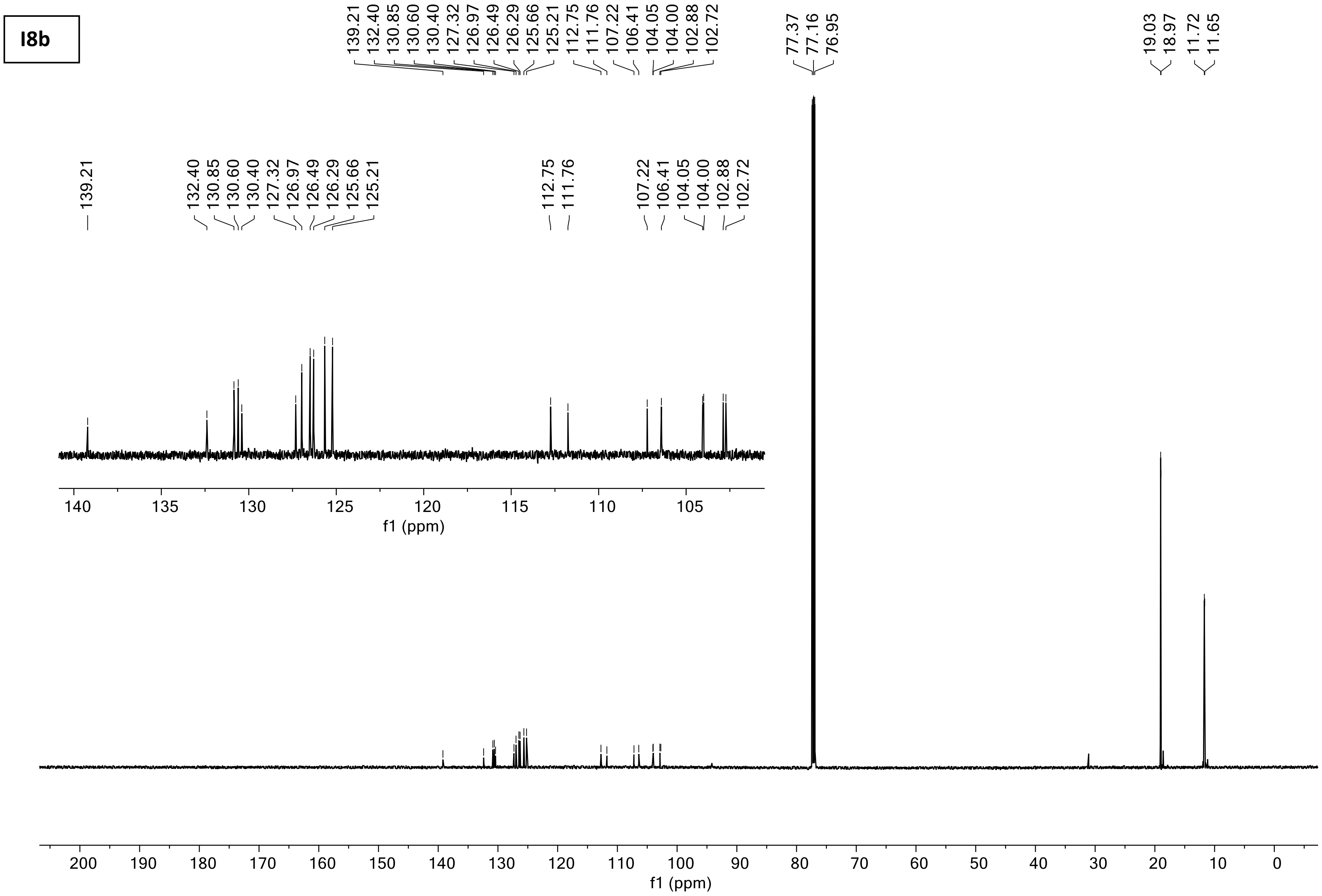

Figure S 105. ^{13}C NMR spectra of **I8b** in $CDCl_3$

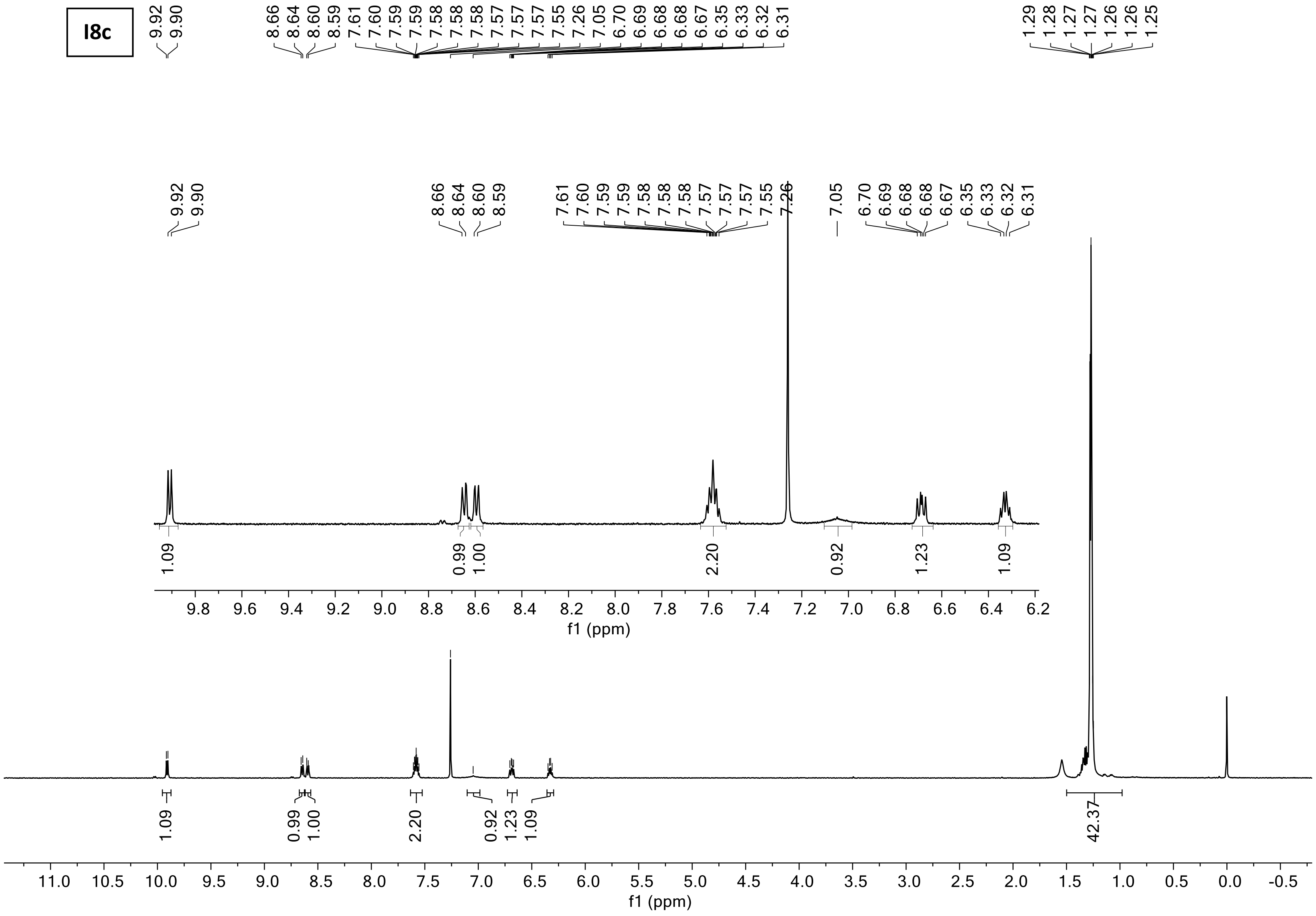

Figure S 106. ^{1}H NMR (top) spectra of **I8c** in $CDCl_3$.

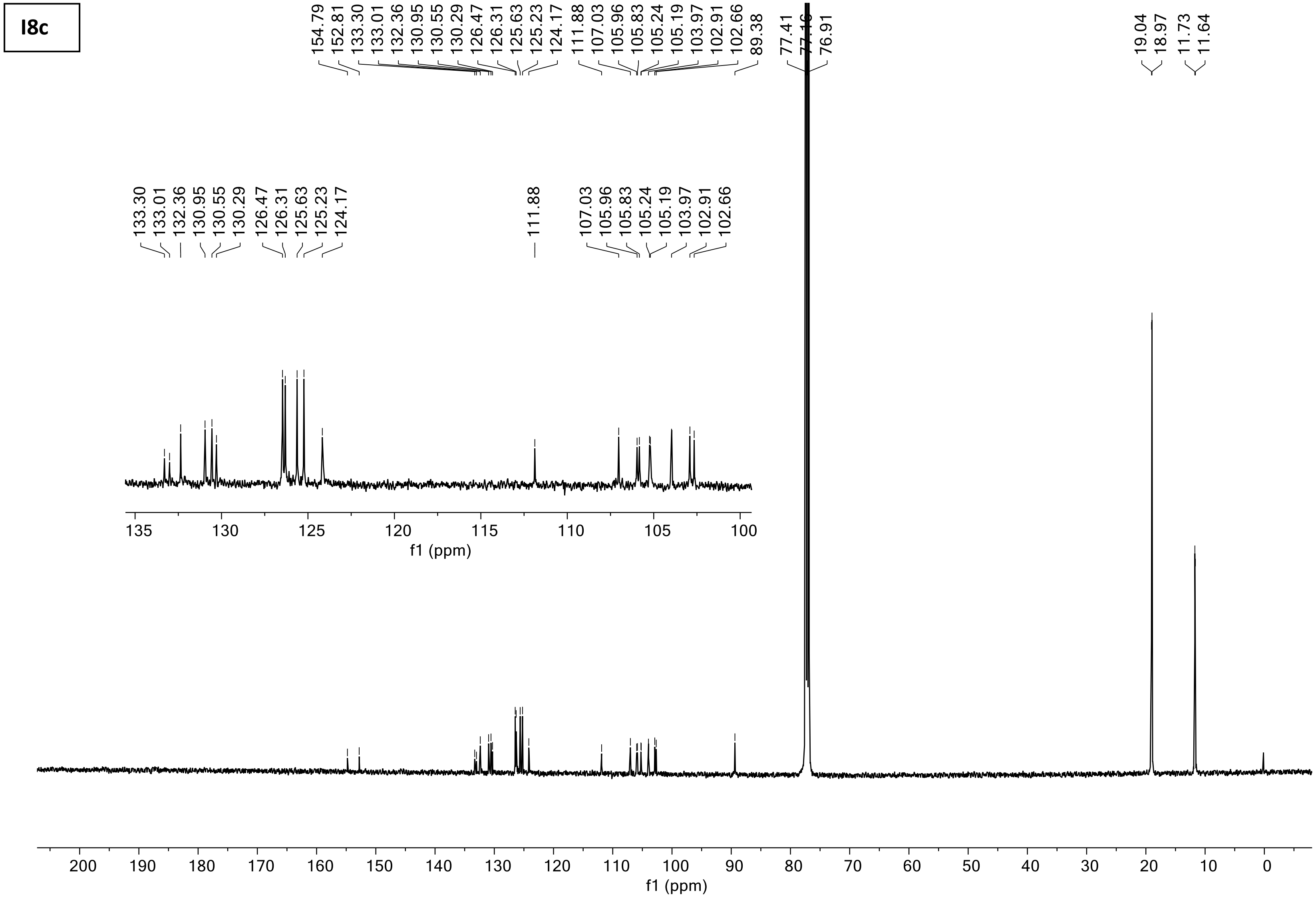

Figure S 107. ^{13}C NMR spectra of **I8c** in $CDCl_3$.

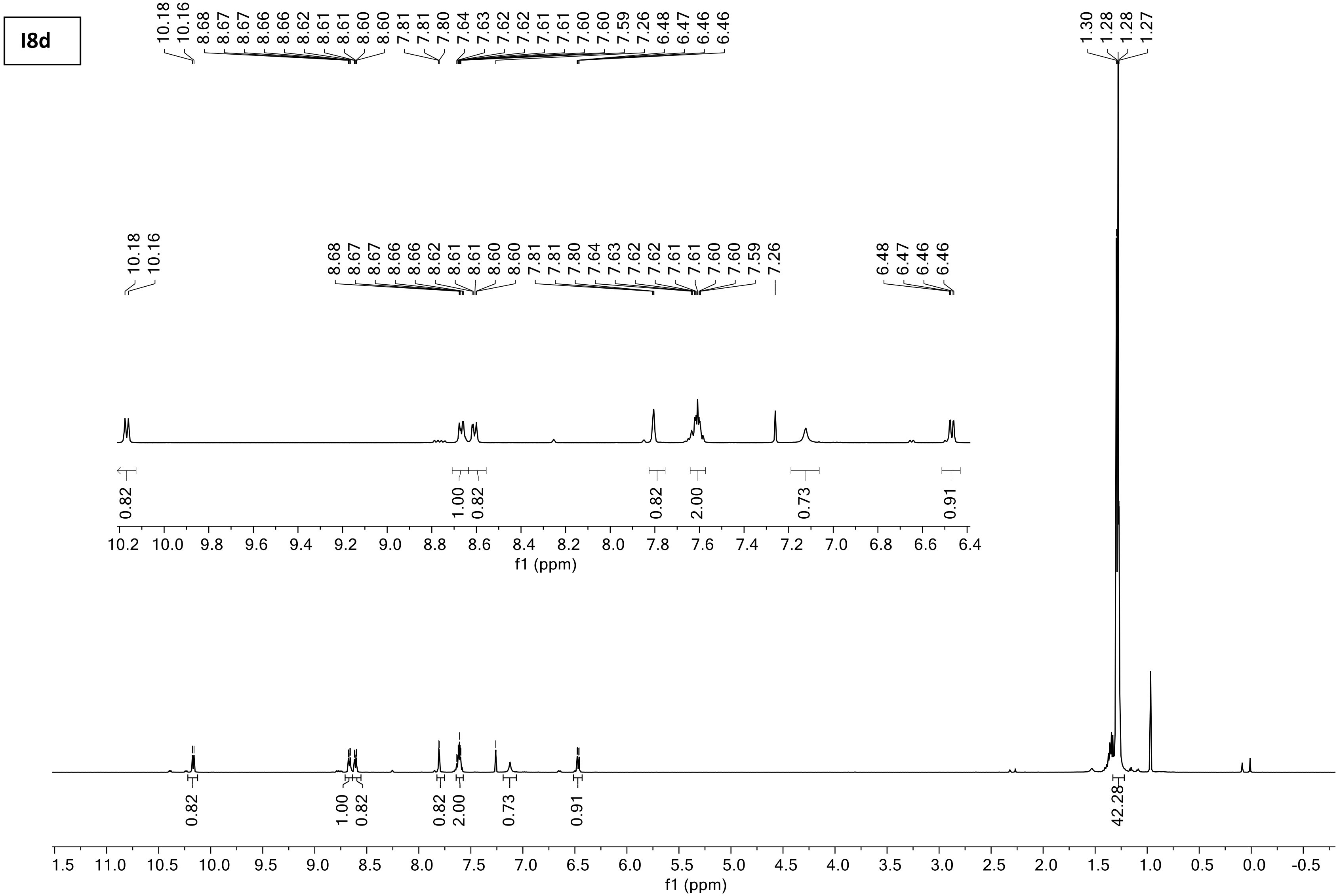

Figure S 108. ^{1}H NMR (top) spectra of **I8d** in $CDCl_3$.

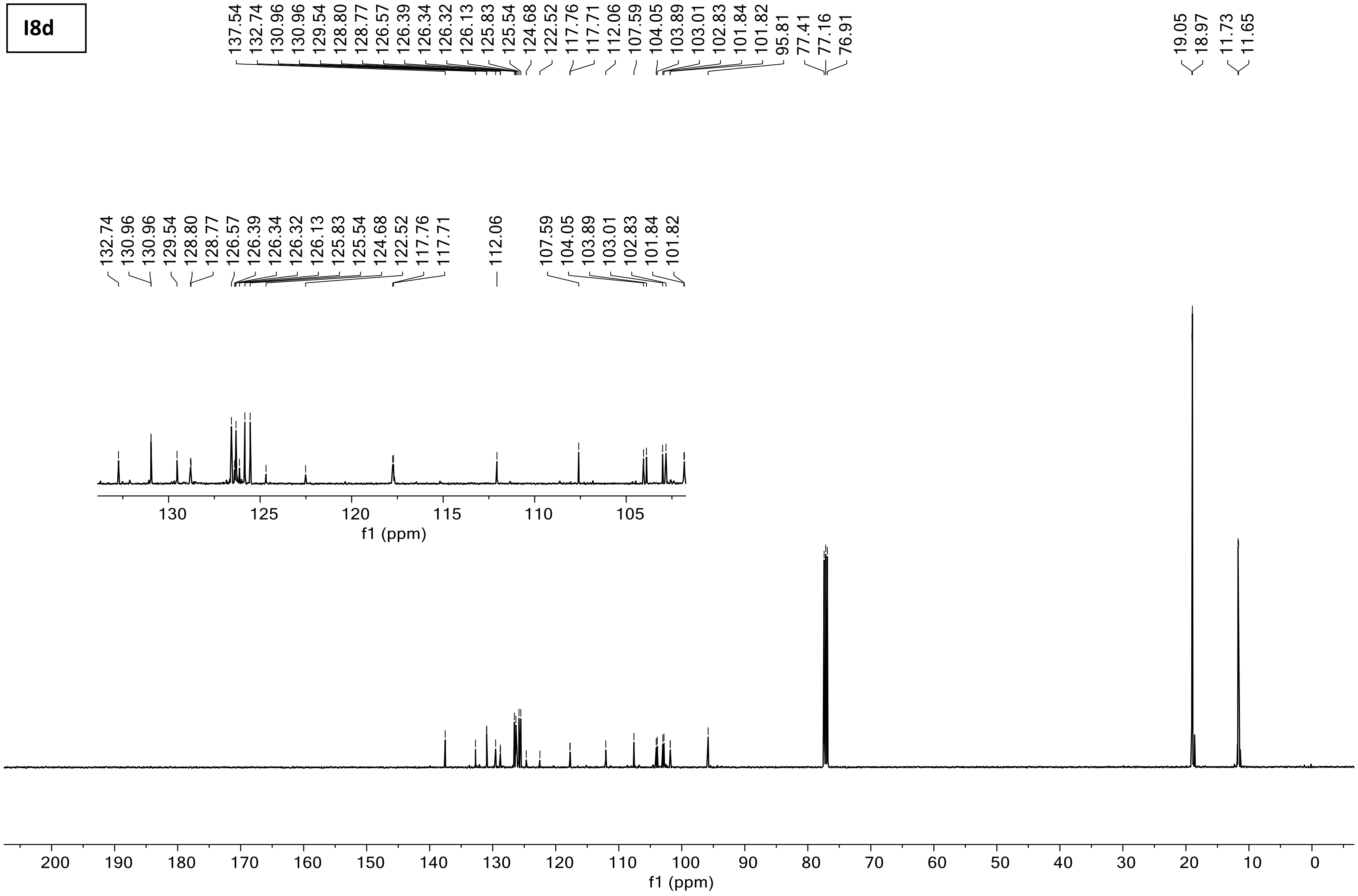

Figure S 109. ^{13}C NMR spectra of **I8d** in $CDCl_3$.

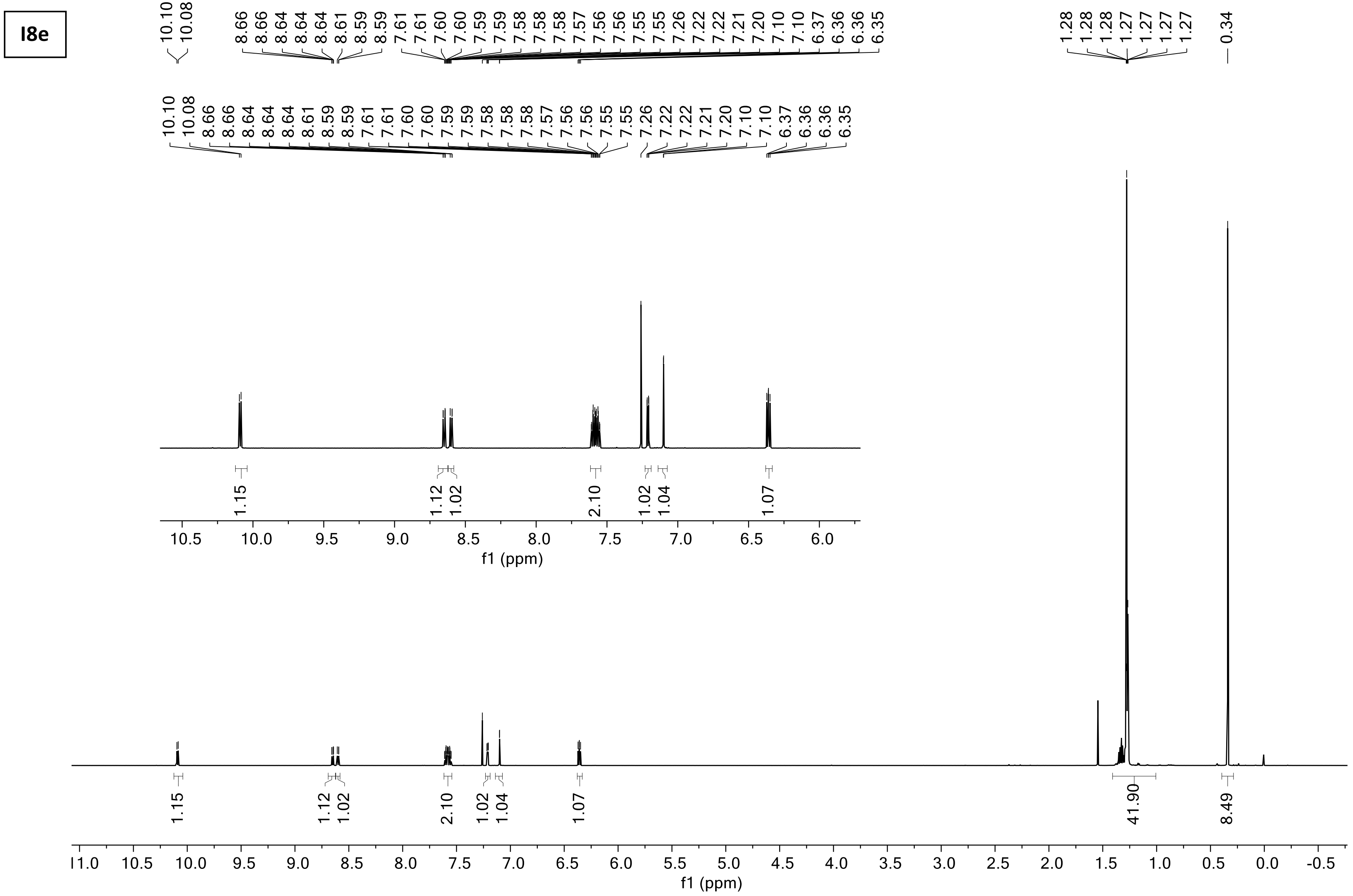

Figure S 110. ^{1}H NMR (top) spectra of **I8e** in $CDCl_3$.

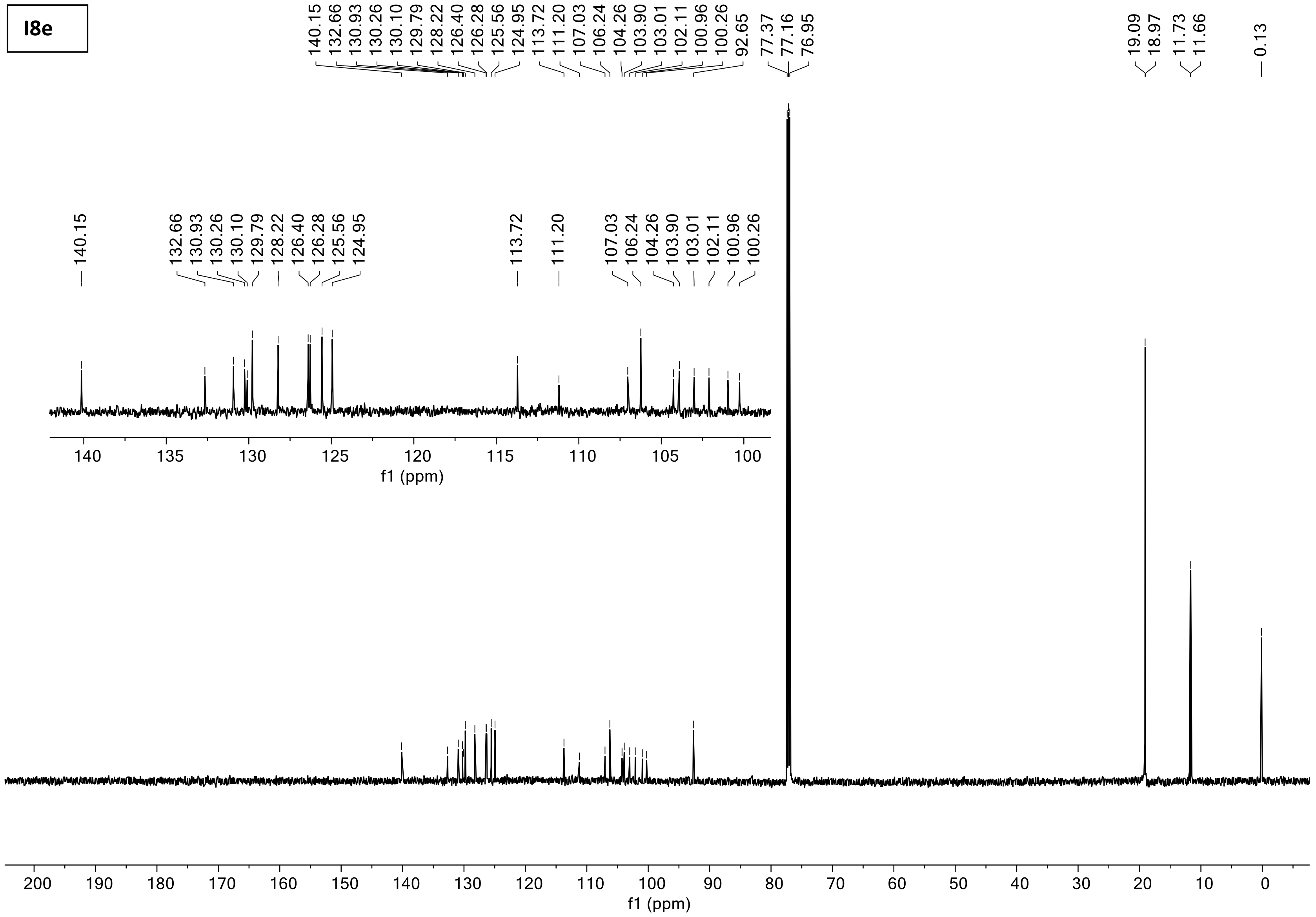

Figure S 111. ^{13}C NMR spectra of **I8e** in $CDCl_3$.

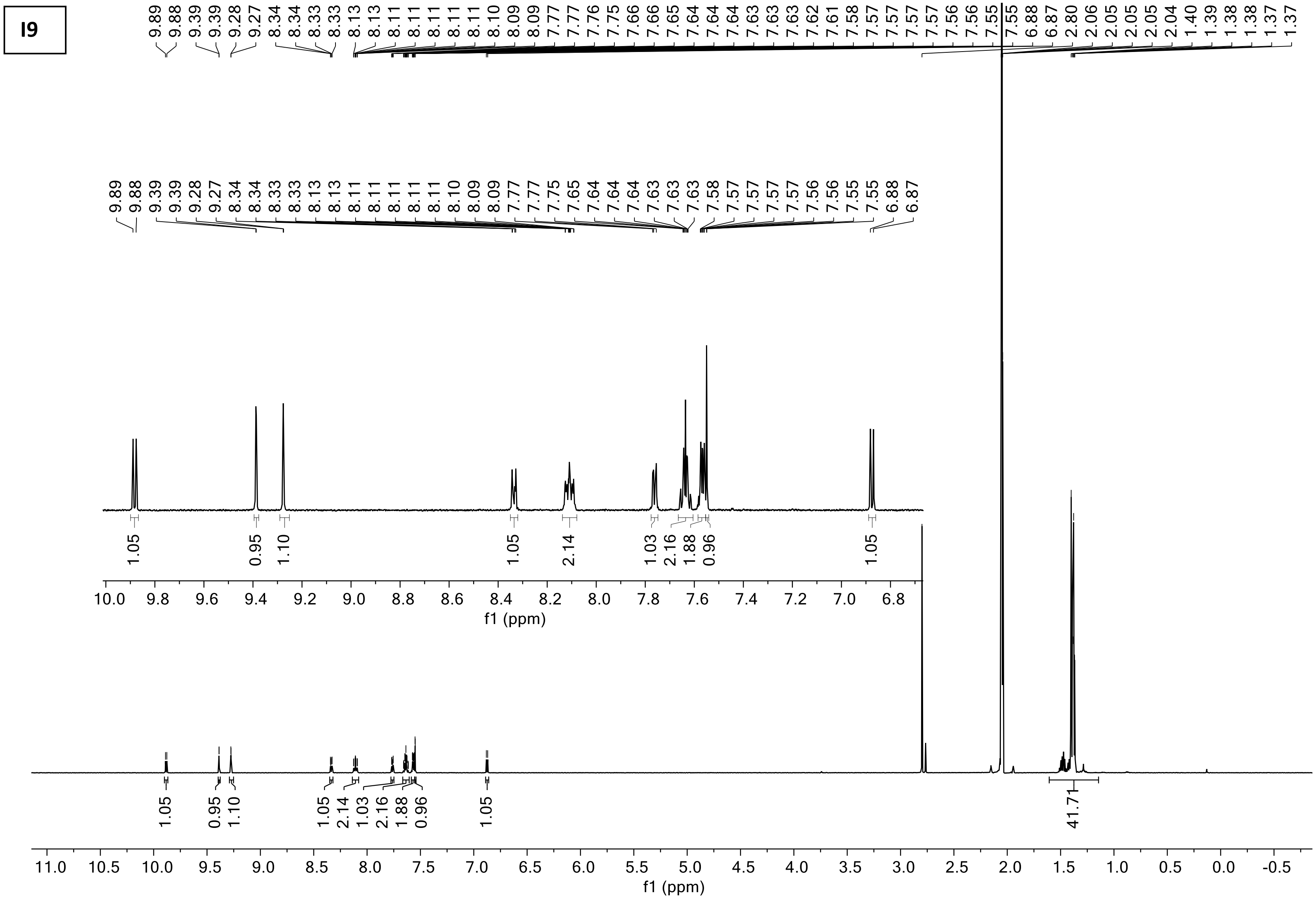

Figure S 112. ^{1}H NMR (top) spectra of **I9** in Acetone-d_6 + CS_2 + DMSO-d_6.

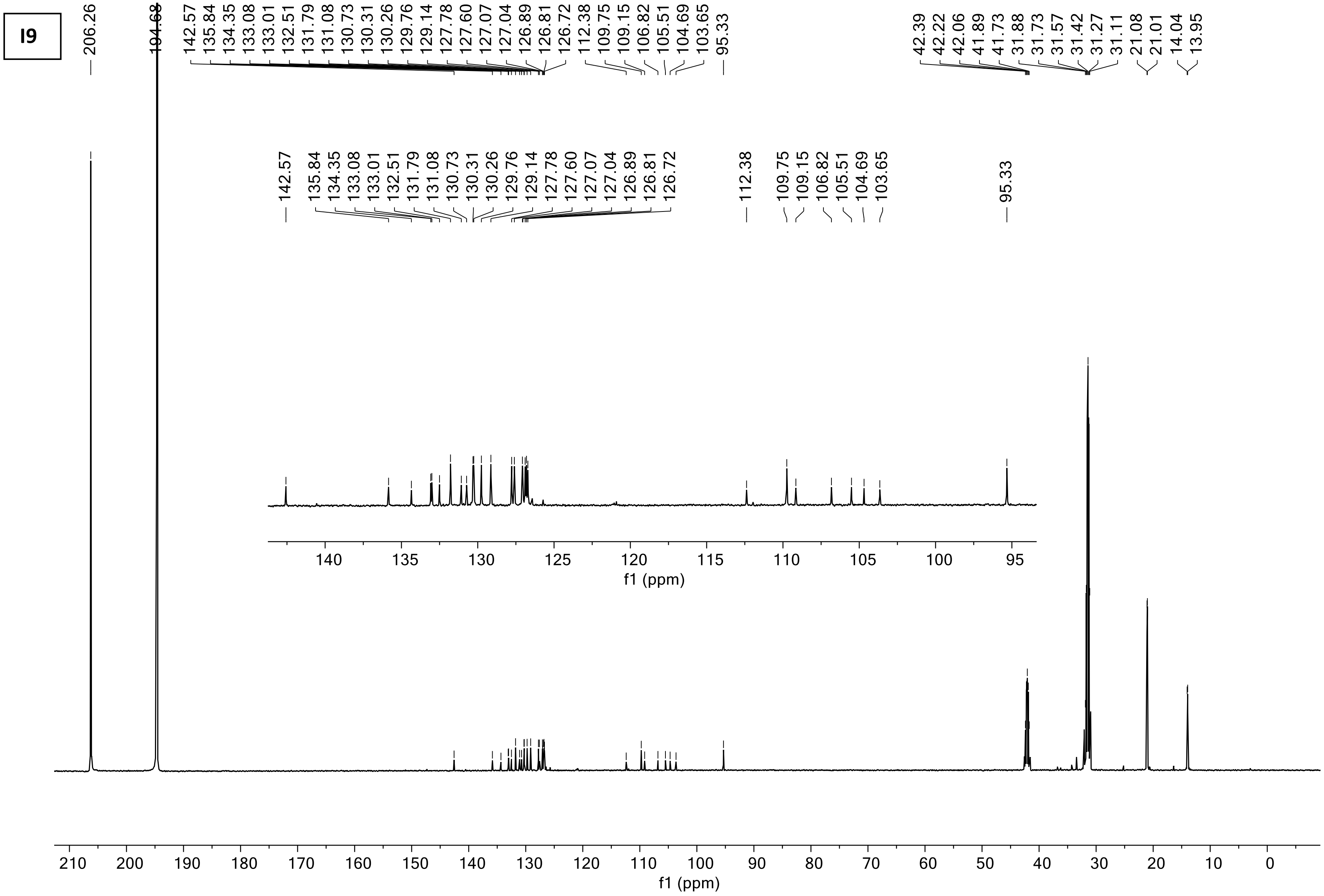

Figure S 113. ^{13}C NMR spectra of I9 in Acetone-d_6+ CS_2 + DMSO-d_6.

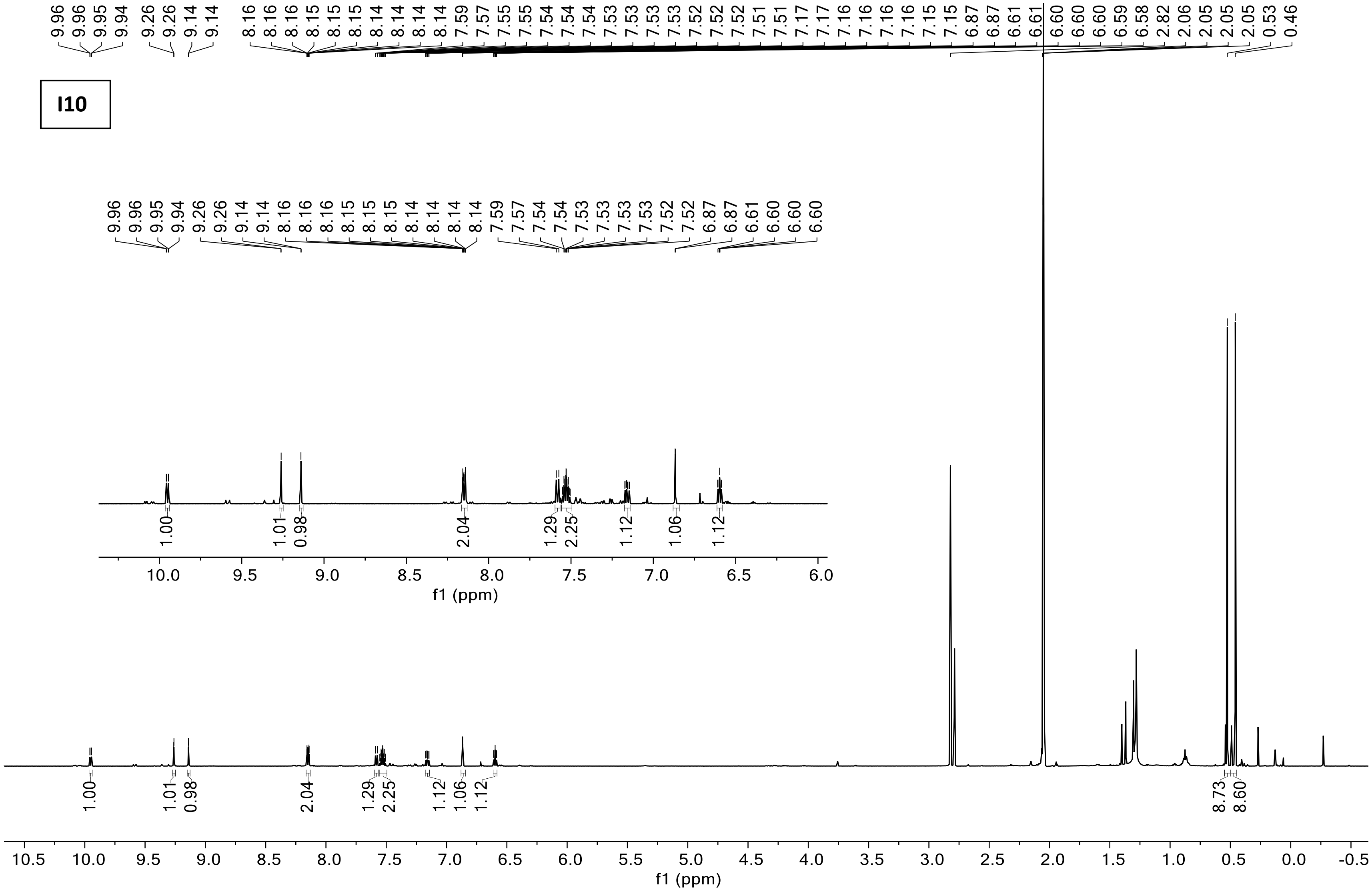

Figure S 114. ^{1}H NMR (top) spectra of **I10** in Acetone-d_6.

S9. References

1. Williams, A. T. R.; Winfield, S. A.; Miller, J. N., Relative fluorescence quantum yields using a computer-controlled luminescence spectrometer. *Analyst* **1983,** *108* (1290), 1067-1071.
2. Morris, J. V.; Mahaney, M. A.; Huber, J. R., Fluorescence quantum yield determinations. 9,10-Diphenylanthracene as a reference standard in different solvents. *The Journal of Physical Chemistry* **1976,** *80* (9), 969-974.
3. Bruker Bruker AXS Inc. : Madison, Wisconsin, USA, 2004.
4. Sheldrick, G., A short history of SHELX. *Acta Crystallographica Section A* **2008,** *64* (1), 112-122.
5. Sheldrick, G. M., *SADABS. Program for Empirical Absorption Correction of Area Detector Data.* Univ. of Göttingen: Göttingen, Germany, 1996.
6. Sheldrick, G. M. *SHELXL-2014. Program for the Refinement of Crystal Structures from Diffraction Data*, University of Göttingen: Germany, 2014.
7. Frisch, M. J.; Trucks, G. W.; Schlegel, H. B.; Scuseria, G. E.; Robb, M. A.; Cheeseman, J. R.; Scalmani, G.; Barone, V.; Petersson, G. A.; Nakatsuji, H.; Li, X.; Caricato, M.; Marenich, A. V.; Bloino, J.; Janesko, B. G.; Gomperts, R.; Mennucci, B.; Hratchian, H. P.; Ortiz, J. V.; Izmaylov, A. F.; Sonnenberg, J. L.; Williams; Ding, F.; Lipparini, F.; Egidi, F.; Goings, J.; Peng, B.; Petrone, A.; Henderson, T.; Ranasinghe, D.; Zakrzewski, V. G.; Gao, J.; Rega, N.; Zheng, G.; Liang, W.; Hada, M.; Ehara, M.; Toyota, K.; Fukuda, R.; Hasegawa, J.; Ishida, M.; Nakajima, T.; Honda, Y.; Kitao, O.; Nakai, H.; Vreven, T.; Throssell, K.; Montgomery Jr., J. A.; Peralta, J. E.; Ogliaro, F.; Bearpark, M. J.; Heyd, J. J.; Brothers, E. N.; Kudin, K. N.; Staroverov, V. N.; Keith, T. A.; Kobayashi, R.; Normand, J.; Raghavachari, K.; Rendell, A. P.; Burant, J. C.; Iyengar, S. S.; Tomasi, J.; Cossi, M.; Millam, J. M.; Klene, M.; Adamo, C.; Cammi, R.; Ochterski, J. W.; Martin, R. L.; Morokuma, K.; Farkas, O.; Foresman, J. B.; Fox, D. J. *Gaussian 16 Rev. C.01*, Wallingford, CT, 2016.
8. Lee, C.; Yang, W.; Parr, R. G., Development of the Colle-Salvetti correlation-energy formula into a functional of the electron density. *Physical Review B* **1988,** *37* (2), 785-789.
9. Becke, A. D., Density-functional thermochemistry. III. The role of exact exchange. *The Journal of Chemical Physics* **1993,** *98* (7), 5648-5652.
10. Weigend, F., Accurate Coulomb-fitting basis sets for H to Rn. *Physical Chemistry Chemical Physics* **2006,** *8* (9), 1057-1065.
11. Weigend, F.; Ahlrichs, R., Balanced basis sets of split valence, triple zeta valence and quadruple zeta valence quality for H to Rn: Design and assessment of accuracy. *Physical Chemistry Chemical Physics* **2005,** *7* (18), 3297-3305.
12. Foroutan-Nejad, C.; Shahbazian, S.; Feixas, F.; Rashidi-Ranjbar, P.; Solà, M., A dissected ring current model for assessing magnetic aromaticity: A general approach for both organic and inorganic rings. *Journal of Computational Chemistry* **2011,** *32* (11), 2422-2431.
13. Gershoni-Poranne, R.; Stanger, A., The NICS-XY-Scan: Identification of Local and Global Ring Currents in Multi-Ring Systems. *Chemistry – A European Journal* **2014,** *20* (19), 5673-5688.
14. Keith, T. A. *AIMAll (Version 19.10.12),* , TK Gristmill Software: Overland Park KS, USA, 2019
15. Ossila https://www.ossila.com/products/tips-pentacene?_pos=2&_sid=18b391a57&_ss=r.
16. Mei, Y.; Fogel, D.; Chen, J.; Ward, J. W.; Payne, M. M.; Anthony, J. E.; Jurchescu, O. D., Interface engineering to enhance charge injection and transport in solution-deposited organic transistors. *Organic Electronics* **2017,** *50*, 100-105.
17. Torsi, L.; Magliulo, M.; Manoli, K.; Palazzo, G., Organic field-effect transistor sensors: a tutorial review. *Chemical Society Reviews* **2013,** *42* (22), 8612-8628.
